# Glucose-dependent insulinotropic polypeptide regulates body weight and food intake via GABAergic neurons in mice

**DOI:** 10.1038/s42255-023-00931-7

**Published:** 2023-11-09

**Authors:** Arkadiusz Liskiewicz, Ahmed Khalil, Daniela Liskiewicz, Aaron Novikoff, Gerald Grandl, Gandhari Maity-Kumar, Robert M. Gutgesell, Mostafa Bakhti, Aimée Bastidas-Ponce, Oliver Czarnecki, Konstantinos Makris, Heiko Lickert, Annette Feuchtinger, Monica Tost, Callum Coupland, Lisa Ständer, Seun Akindehin, Sneha Prakash, Faiyaz Abrar, Russell L. Castelino, Yantao He, Patrick J. Knerr, Bin Yang, Wouter F. J. Hogendorf, Shiqi Zhang, Susanna M. Hofmann, Brian Finan, Richard D. DiMarchi, Matthias H. Tschöp, Jonathan D. Douros, Timo D. Müller

**Affiliations:** 1 Institute for Diabetes and Obesity, Helmholtz Diabetes Center, Helmholtz Munich, Neuherberg, Germany; 2 German Center for Diabetes Research (DZD), Neuherberg, Germany; 3 Department of Physiology, Faculty of Medical Sciences in Katowice, Medical University of Silesia, Katowice, Poland; 4 Institute of Physiotherapy and Health Sciences, Academy of Physical Education, Katowice, Poland; 5 Institute of Diabetes and Regeneration Research, Helmholtz Diabetes Center, Helmholtz Zentrum München, Neuherberg, Germany; 6 TUM School of Medicine, Technical University of Munich, Munich, Germany; 7 Core Facility Pathology & Tissue Analytics, Helmholtz Munich, Neuherberg, Germany; 8 Novo Nordisk Research Center Indianapolis, Indianapolis, IN USA; 9 Department of Medicine IV, University Hospital, LMU Munich, Munich, Germany; 10 Department of Chemistry, Indiana University, Bloomington, IN USA; 11 Helmholtz Munich, Neuherberg, Germany; 12 Division of Metabolic Diseases, Department of Medicine, Technical University of Munich, Munich, Germany

**Keywords:** Pharmacodynamics, Neuroendocrinology, Obesity

## Abstract

The development of single-molecule co-agonists for the glucagon-like peptide-1 (GLP-1) receptor (GLP-1R) and glucose-dependent insulinotropic polypeptide (GIP) receptor (GIPR) is considered a breakthrough in the treatment of obesity and type 2 diabetes. But although GIPR–GLP-1R co-agonism decreases body weight with superior efficacy relative to GLP-1R agonism alone in preclinical^1–3^ and clinical studies^4,5^, the role of GIP in regulating energy metabolism remains enigmatic. Increasing evidence suggests that long-acting GIPR agonists act in the brain to decrease body weight through the inhibition of food intake^3,6–8^; however, the mechanisms and neuronal populations through which GIP affects metabolism remain to be identified. Here, we report that long-acting GIPR agonists and GIPR–GLP-1R co-agonists decrease body weight and food intake via inhibitory GABAergic neurons. We show that acyl-GIP decreases body weight and food intake in male diet-induced obese wild-type mice, but not in mice with deletion of *Gipr* in *Vgat*(also known as *Slc32a1*)-expressing GABAergic neurons (*Vgat-Gipr* knockout). Whereas the GIPR–GLP-1R co-agonist MAR709 leads, in male diet-induced obese wild-type mice, to greater weight loss and further inhibition of food intake relative to a pharmacokinetically matched acyl-GLP-1 control, this superiority over GLP-1 vanishes in *Vgat-Gipr* knockout mice. Our data demonstrate that long-acting GIPR agonists crucially depend on GIPR signaling in inhibitory GABAergic neurons to decrease body weight and food intake.

## Abstract (web summary)

Long-acting GIPR agonists, individually or as part of GIPR–GLP-1R co-agonists, are shown to require GIPR signaling in inhibitory GABAergic neurons to decrease body weight and food intake in mice.

## Main

The development of GIPR–GLP-1R co-agonists have been a major advancement in the treatment of obesity and diabetes^9^, but the mechanisms through which GIP affects systemic energy metabolism remain largely unknown. Accumulating evidence indicates that long-acting GIPR agonists act in the brain to decrease body weight through inhibition of food intake^3,6–8^. Chemogenetic activation of hypothalamic and hindbrain GIPR neurons decreases food intake in mice^6,7^ and long-acting GIPR agonists decrease body weight and food intake in obese wild-type mice^3,8^, but not in mice with *Nes-cre*-mediated neuronal loss of *Gipr*^3^. Accumulating evidence indicates that GIPR agonism is also a vital constituent to GIPR–GLP-1R co-agonism. The GIPR–GLP-1R co-agonist MAR709 leads relative to a pharmacokinetically matched acyl-GLP-1 to greater weight loss and further inhibition of food intake, but this superiority vanishes in mice with neuronal loss of *Gipr*^3^. And while the GIPR–GLP-1R co-agonist tirzepatide promotes insulin secretion in isolated human islets primarily via the GIP receptor^10^, long-acting GIPR agonists attenuate the emetic effect of GLP-1R agonism in experimental animals^11,12^ and hence likely contribute to greater tolerability of GIPR–GLP-1R co-agonism relative to GLP-1R agonism at higher doses. Although the mechanisms and neuronal populations through which GIP affects body weight and food intake have yet to be identified, these data collectively indicate that GIPR agonism is a vital constituent to the metabolic efficacy and tolerability of GIPR–GLP-1R co-agonism.

Several studies have recently assessed the expression profile of *Gipr* in the brain using single-cell RNA-sequencing (scRNA-seq) analysis, revealing that *Gipr* is expressed in a variety of different cells types within the hypothalamus and the hindbrain, including neurons, mesenchymal cells, mural cells and oligodendrocytes^7,13–15^. In both of these brain areas, expression of *Gipr* is found in cells or neurons that express *Slc32a1* (also known as *Vgat*), a marker indicative of inhibitory GABAergic neurons^6,14,16,17^. GABAergic *Gipr* neurons seem crucial for the anti-emetic effect of GIPR agonism^11,13^, but their role in energy metabolism remains unknown. Emphasizing their potential role in energy balance regulation, *Vgat*-expressing GABA neurons are implicated in the control of eating behavior^18–20^, and while optogenetic stimulation of VGAT neurons in the lateral hypothalamus promotes food intake, genetic ablation of these neurons has the opposite effect^18^. Although being expressed in only 14–18% of all *Gipr* cells in the hypothalamus^15^ and the hindbrain^14^, *Vgat* it is found in around 32% of hypothalamic *Gipr* neurons^15^ and, depending on the study, in up to 55% of hindbrain *Gipr* neurons^14,16,17,21^ (Extended Data Table 1). Based on the expression of *Gipr* in GABAergic neurons^7,11,14,15^, and the demonstration that selective activation of hypothalamic and hindbrain GIPR neurons decreases food intake in mice^6,7^, we here assessed whether the metabolic effects of GIP and GIPR–GLP-1R co-agonism depend on GIPR signaling in inhibitory GABAergic neurons.

**Fig. 1:**
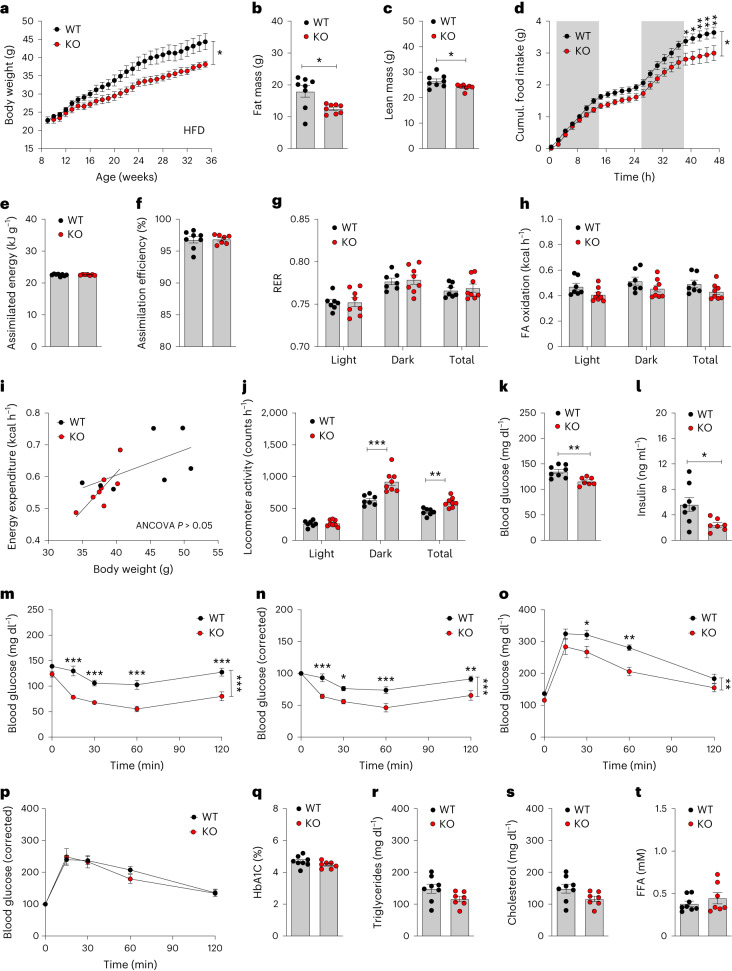
Metabolic characterization of HFD-fed *Vgat-Gipr* KO mice. **a**–**c**, Body weight development (**a**) and body composition of 35-week-old male C57BL/6J wild-type (WT) and *Vgat-Gipr* knockout (KO) mice (*n* = 7–8 each group) (**b**,**c**). **d**–**f**, Food intake in 35-week-old male C57BL/6J mice (*n* = 7 each group) (**d**) as well as assimilated energy and assimilation efficiency in 35-week-old male C57BL/6J mice (*n* = 7–8 each group) (**e**,**f**). **g**–**j**, Respiratory exchange ratio (RER) (**g**), fatty acid (FA) oxidation (**h**), energy expenditure (**i**) and locomotor activity (**j**) in 35-week-old male C57BL/6J mice (*n* = 7–8 each group). **k**,**l**, Fasting levels of blood glucose (**k**) and insulin (**l**) in 37-week-old male C57BL/6J mice (*n* = 7–8 each group). **m**–**p**, Intraperitoneal insulin tolerance in 38-week-old male C57BL/6J mice (*n* = 7–8 each group) (**m**,**n**) and glucose tolerance in 34-week-old male C57BL/6J mice (**o**,**p**). **q**–**t**, HbA1c (**q**) and plasma levels of triglycerides (**r**), cholesterol (**s**) and free fatty acids (FFA) (**t**) in 40-week-old male C57BL/6J mice (*n* = 7–8 each group). Data in **a**,**d**,**m**–**p** were analyzed by repeated measures two-way analysis of variance (ANOVA) with Bonferroni’s post hoc test for comparison of individual time points. Data in **b**,**c**,**e**–**h**,**j**,**k**,**l**,**q**–**t** were analyzed using a Student’s two-sided, two-tailed *t*-test. Data in **i** were analyzed using ANCOVA with body weight as covariate. Date are mean ± s.e.m.; *P < 0.05; **P < 0.01 and ***P < 0.001. Individual *P* values are shown in the Source Data file, unless *P* < 0.0001. Source data

**Extended Data Fig. 1:**
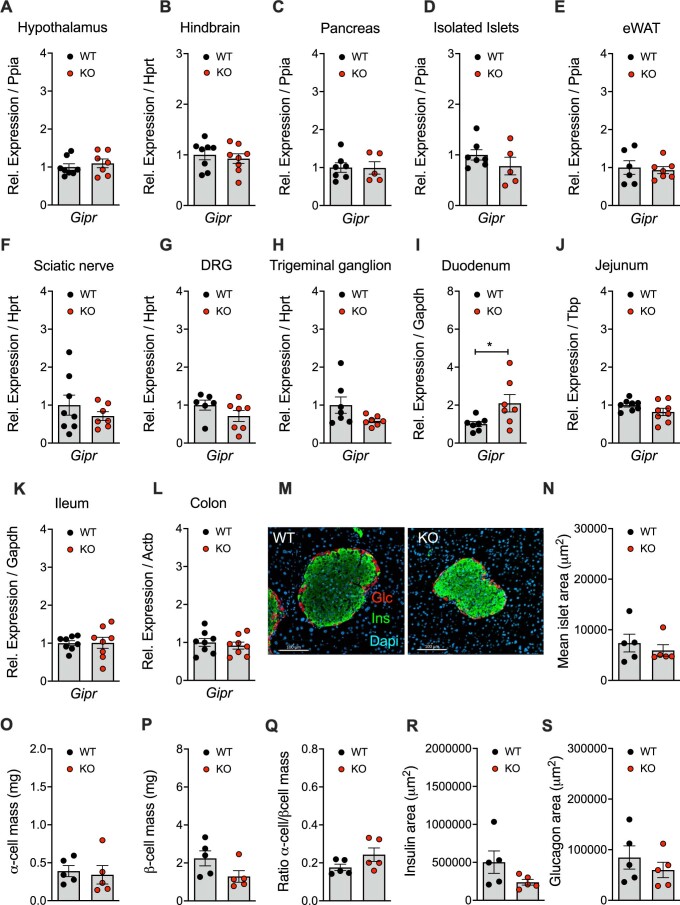
Tissue-selective expression of *Gipr* in wild-type and *Vgat-Gipr* Ko mice. Expression of *Gipr* in hypothalamus (n = 7-8 each group) **(a)**, hindbrain (n = 8 each group) **(b)**, and pancreas (5-7 each group) of 40-wk old HFD-fed DIO male C57BL/6J wild-type and *Vgat-Gipr* KO mice **(c)**. Expression of *Gipr* in pancreatic islets isolated from 12-wk old male C57BL/6J wild-type and *Vgat-Gipr* KO mice **(d)** and Gipr expression in epididymal white adipose tissue (eWAT) (n = 6-7 each group) of 40-wk old HFD-fed DIO male C57BL/6J wild-type and *Vgat-Gipr* KO mice **(e)**. Expression of *Gipr* in sciatic nerve (n = 7-8 each group) **(f)**, dorsal root ganglia (DRG) (n = 6-7 each group) **(g)**, trigeminal ganglion (n = 7 each group) **(h)**, as well as in duodenum (n = 7 each group) **(i)**, jejunum (n = 8 each group) **(j)**, ileum (n = 8 each group) **(k)**, and colon (n = 8 each group) **(l)** of 12-wk old HFD-fed male C57BL/6J wild-type and *Vgat-Gipr* KO mice. Representative image of of insulin and glucagon immunostaining in pancreata of 38-wk old HFD-fed male C57BL/6J wild-type and *Vgat-Gipr* KO mice (n = 5 each group) **(m)**, and quantification of pancreatic islet area (n = 5 each group) **(n)**, *α*-cell mass (n = 5 each group) **(o)**, β-cell mass (n = 5 each group) **(p)**, *α*-cell/β-cell mass ratio (n = 5 each group) **(q)**, as well insulin area (n = 5 each group) **(r)** and glucagon area (n = 5 each group) **(s)** in 38-wk old male HFD-fed C57BL/6J wild-type and *Vgat-Gipr* KO mice. Data were analyzed using student’s 2-sided, two-tailed t-test. Data represent mean ± SEM. Asterisk indicates * p < 0.05. Individual p values are shown in the Data Source file. Source data

**Extended Data Fig. 2:**
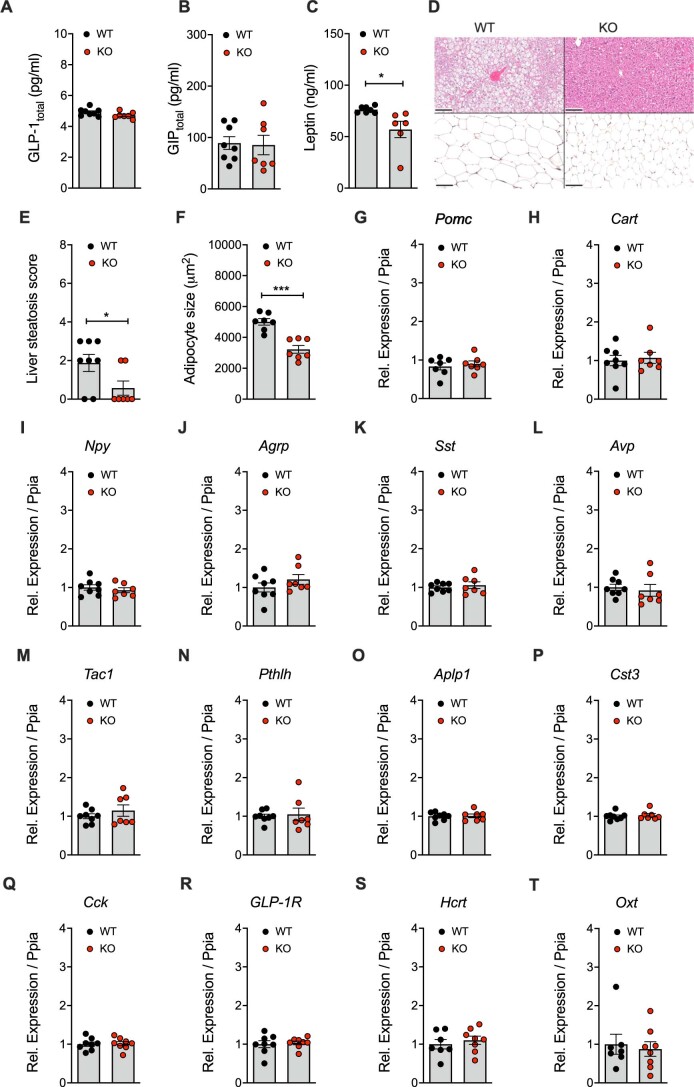
Tissue histology and hypothalamic target gene expression in DIO wild-type and *Vgat-Gipr* KO mice. Ad libitum plasma levels of GLP-1_total_ **(a)** and GIP_total_ **(b)** in 40-wk old male C57BL/6J DIO wild-type and Vgat-Gipr KO mice mice (n = 7-8 each group). Serum levels of leptin in 40-wk old male C57BL/6J DIO wild-type and Vgat-Gipr KO mice (n = 6-7 each group) **(c)**, representative H&E staining of liver and inguinal white adipose tissue (iWAT) in 40-wk old C57BL/6J mice (n = 7-8 each group, scale bar: 100μm) **(d)**, corresponding steatosis score (n = 7-8 each group) **(e)** and mean iWAT adipocyte size (n = 7 each group) **(f)**, as well as hypothalamic expression of proopiomelanocortin; *Pomc* (n = 7 each group) **(g)**, cocaine and amphetamine regulated transcript; *Cart* (n = 7-8 each group) **(h)**, neuropeptide y; *Npy* (n = 7 each group) **(i)**, agouti-related peptide; *Agrp* (n = 7 each group) **(j)**, somatostatin; *Sst* (n = 7 each group) **(k)**, arginine vasopressin; *Avp* (n = 7 each group) **(l)**, tachykinin precursor 1; *Tac1* (n = 7 each group) **(m)**, parathyroid hormone like hormone; *Pthlh* (n = 7 each group) **(n)**, amyloid beta precursor like protein 1; *Aplp1* (n = 7 each group) **(o)** and cystacin c; *Cst3* (n = 7 each group) **(p)** in 40-wk old male C57BL/6J DIO wild-type and *Vgat-Gipr* KO mice. Expression of cholescystokinin; *Cck* **(q)**, *GLP-1R* **(r)**, hypocretin neuropeptide precursor; *Hcrt* **(s)** and oxytocin; *Oxt* **(t)** in the hindbrain of 40-wk old male C57BL/6J DIO wild-type and *Vgat-Gipr* KO mice. Data were analyzed using student’s 2-sided, two-tailed t-test. Date represent mean ± SEM. Asterisks indicate * p < 0.05; ** p < 0.01 and *** p < 0.001. Individual p values are shown in the Data Source file. Source data

High-fat diet (HFD)-fed male *Vgat-Gipr* knockout (KO) mice show decreased body weight and improved glucose metabolism. Mice with deletion of *Gipr* in inhibitory GABAergic neurons were generated by crossing C57BL/6J *Gipr*^flx/flx^ mice^22,23^ with C57BL/6J *Vgat-ires-cre* knock-in mice (Jackson Laboratories; 028862), which express Cre recombinase under control of the *Vgat* promoter. Consistent with scRNA-seq data showing that *Vgat* is only expressed in 14–18% of all *Gipr*-expressing cells in the hypothalamus^15^ and hindbrain^14^ (Extended Data Table 1)*, Vgat-cre*^+/−^*Gipr*^flx/flx^ mice (*Vgat-Gipr* KO) show relative to *Vgat-cre*^+/−^*Gipr*^wt/wt^ controls (wild-type) no overt changes in *Gipr* expression in either the hypothalamus or the hindbrain (Extended Data Fig. 1a,b). *Vgat-Gipr* KO mice show further no decreased expression of *Gipr* in the pancreas, isolated pancreatic islets, epididymal white adipose tissue (eWAT), peripheral nervous system (sciatic nerve, dorsal root ganglia and trigeminal ganglion) and the gut (duodenum, jejunum, ileum and colon) (Extended Data Fig. 1c–l). But consistent with the phenotype seen in mice with *Nes-cre*-mediated neuronal loss of *Gipr*^3^, male *Vgat-Gipr* KO mice show, relative to wild-type controls, decreased body weight when chronically fed HFD (Fig. 1a). The decreased body weight in *Vgat-Gipr* KO mice is accompanied by decreased fat and lean tissue mass (Fig. 1b,c) and is mediated by decreased food intake (Fig. 1d) without alterations in nutrient absorption (Fig. 1e,f), substrate utilization (Fig. 1g) or fatty acid oxidation (Fig. 1h). Male *Vgat-Gipr* KO mice show no difference in energy expenditure, although locomotor activity is increased (Fig. 1i,j). Similar to mice with global^24^ or neuronal^3^ loss of *Gipr*, HFD-fed male *Vgat-Gipr* KO mice exhibit decreased fasting levels of blood glucose and insulin (Fig. 1k,l), improved insulin sensitivity (Fig. 1m,n) and improved glucose control that is, however, lost after normalizing to baseline glucose levels (Fig. 1o,p). Male *Vgat-Gipr* KO mice show no differences in pancreatic islet size, α- and β-cell mass and insulin and glucagon immunoreactivity (Extended Data Fig. 1m–s). No differences are observed in glycated hemoglobin (HbA1c) or plasma levels of triglycerides, cholesterol and free fatty acids (Fig. 1q–t). Also, ad libitum levels of plasma GLP-1_total_ and GIP_total_ are unchanged between *Vgat-Gipr* KO mice and wild-type controls (Extended Data Fig. 1a,b). But consistent with the lower body fat mass (Fig. 1b), HFD-fed male *Vgat-Gipr* KO mice show decreased plasma levels of leptin, reduced hepatosteatosis and reduced adipocyte size in the inguinal white adipose tissue (Extended Data Fig. 2c–f). No differences are observed in hypothalamic expression of proopiomelanocortin (*Pomc*), cocaine and amphetamine-regulated transcript (*Cart*), neuropeptide y (*Npy*), agouti-related peptide (*Agrp*), somatostatin (*Sst*), arginine vasopressin (*Avp*), tachykinin precursor 1 (*Tac1*), parathyroid hormone-like hormone (*Pthlh*), amyloid-β precursor like protein 1 (*Aplp1*) or cystatin c (*Cst3*) (Extended Data Fig. 2g–p), or in hindbrain expression of cholecystokinin (*Cck*), GLP-1R (*Glp1r*), hypocretin neuropeptide precursor (*Hcrt*) or oxytocin (*Oxt)* (Extended Data Fig. 2q–t). Collectively, male *Vgat-Gipr* KO mice largely resemble the phenotype of *Nes-Gipr* KO mice^3^ as reflected by decreased body weight and food intake, and improved glucose control when fed a HFD.

**Fig. 2:**
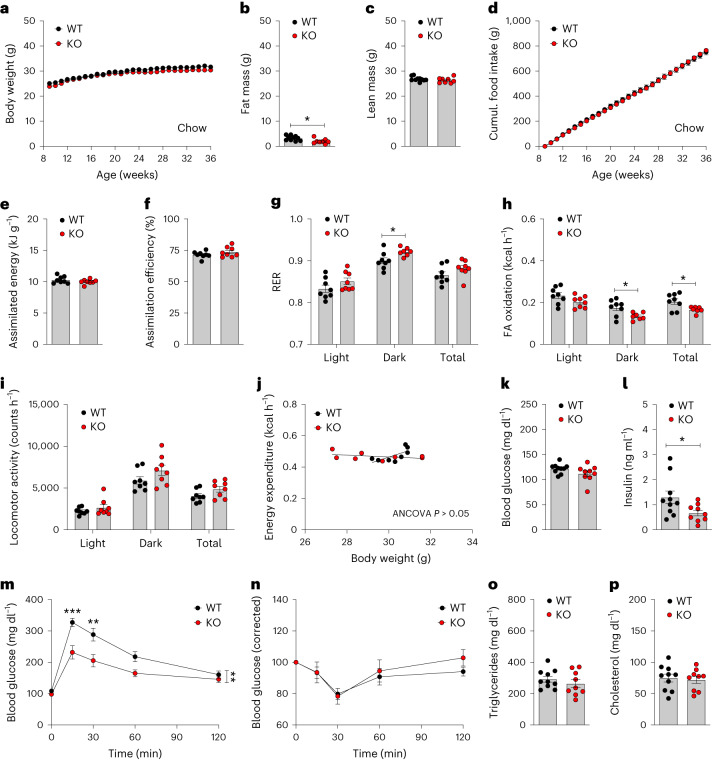
Metabolic characterization of chow-fed *Vgat-Gipr* KO mice. **a**–**c**, Body weight development (*n* = 9–10 each group) (**a**) and body composition (*n* = 8–10 each group) of 36-week-old male C57BL/6J WT and *Vgat-Gipr* KO mice (**b**,**c**). **d**–**f**, Food intake in 36-week-old male C57BL/6J mice (*n* = 8–10 each group) (**d**) as well as assimilated energy and assimilation efficiency in 36-week-old male C57BL/6J mice (*n* = 8 each group) (**e**,**f**). **g**–**j**, RER (**g**), fatty acid oxidation (**h**), locomotor activity (**i**) and energy expenditure (**j**) in 36-week-old male C57BL/6J mice (*n* = 8 each group). **k**,**l**, Fasting levels of blood glucose (**k**) and insulin (**l**) in 38-week-old male C57BL/6J mice (*n* = 9–10 each group).**m**,**n**, Glucose tolerance in 38-week-old male C57BL/6J mice (*n* = 9–10 each group) (**m**) and insulin tolerance (**n**) in 39-week-old male C57BL/6J mice (*n* = 8–10 each group). **o**,**p**, Ad libitum plasma levels of triglycerides (**o**) and cholesterol (**p**) in 40-week-old male C57BL/6J mice (*n* = 9–10 each group). Data in **a**,**d**,**m**,**n** were analyzed by repeated measures two-way ANOVA with Bonferroni’s post hoc test for comparison of individual time points. Data in **b**,**c**,**e**–**i**,**k**,**l**,**o**,**p** were analyzed using a Student’s two-tailed *t*-test. Data in **j** were analyzed using ANCOVA with body weight as covariate. Cumulative food intake in **d** was assessed per cage in *n* = 8–10 double-housed mice. Date are mean ± s.e.m.; *P < 0.05; **P < 0.01 and ***P < 0.001. Individual *P* values are shown in the Source Data file, unless *P* < 0.0001. Source data

**Extended Data Fig. 3:**
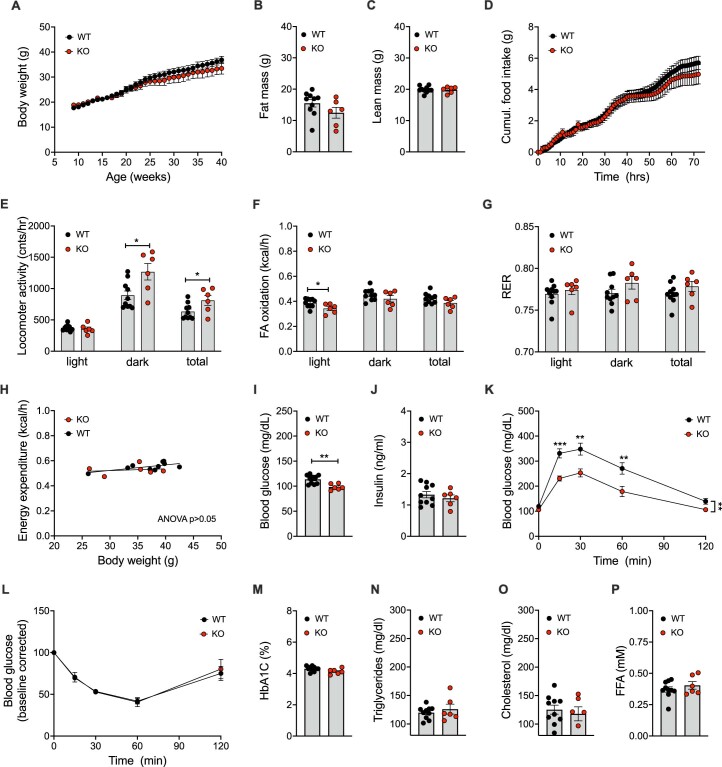
Metabolic characterization of female HFD-fed *Vgat-Gipr* KO mice. Body weight development **(a)**, and body composition **(b)** of 40-wk old female HFD-fed C57BL/6J wild-type (WT) and *Vgat-Gipr* KO (KO) mice (WT/KO n = 10/6 mice). Cumulative food intake **(d)**, as well as locomotor activity **(e)**, fatty acid (FA) oxidation **(f)**, respiratory exchange ratio (RER) **(G)** and energy expenditure **(h)** in 40-wk old female C57BL/6J mice (WT/KO n = 10/6 mice). Fasting plasma levels of blood glucose **(i)** and insulin **(j)** in 43-wk old female C57BL/6J mice (WT/KO n = 10/6 mice), intraperitoneal glucose tolerance in 42-wk old female C57BL/6J mice (WT/KO n = 10/6 mice) **(k)**, intraperitoneal insulin tolerance in 42-wk old female C57BL/6J mice (WT/KO n = 9/6 mice) **(l)** and plasma levels HbA1c **(m)**, triglycerides **(n)**, cholesterol **(o)** and free fatty acids **(p)** in 44-wk old female C57BL/6J mice (WT/KO n = 10/6 mice). Data in panel **A,D,K and L** were analyzed by repeated measures 2-way ANOVA with Bonferroni’s post hoc test for comparison of individual time points. Data in panel **B,C,E-G,I,J,M-P** were analyzed using student’s 2-sided, two-tailed t-test. Data in panel **H** were analyzed using ANCOVA with body weight as covariate. Date represent means ± SEM; asterisks indicate * p < 0.05; ** p < 0.01 and *** p < 0.001. Individual p values are shown in the Data Source file. Source data

We found that chow-fed male *Vgat-Gipr* KO mice show normal body weight but improved glucose metabolism. Male *Vgat-Gipr* KO mice also mimic the phenotype of the global^24^ and neuronal^3^ *Gipr* KO mice when fed a regular chow diet. Chow-fed *Vgat-Gipr* KO mice show no overt differences in body weight, body composition or food intake relative to wild-type controls (Fig. 2a–d). Absorption and utilization of nutrients is also not different between chow-fed *Vgat-Gipr* KO mice and wild-type controls (Fig. 2e–g), although fatty acid oxidation is decreased in the *Vgat-Gipr* KO mice (Fig. 2h). *Vgat-Gipr* KO mice show no differences in locomotor activity (Fig. 2i), energy expenditure (Fig. 2j) or fasting levels of blood glucose (Fig. 2k), but insulin levels are decreased (Fig. 2l). Chow-fed *Vgat-Gipr* KO mice further show improved glucose tolerance (Fig. 2m) without alterations in insulin sensitivity (Fig. 2n) or plasma levels of triglycerides and cholesterol (Fig. 2o,p). Similar to the chow-fed male *Vgat-Gipr* KO mice, female *Vgat-Gipr* KO mice show no difference in body weight relative to wild-type controls, even when fed a HFD (Extended Data Fig. 3a). Female *Vgat-Gipr* KO mice show no difference in body composition or food intake relative to wild-type controls, but locomotor activity is slightly enhanced, without changes in fatty acid oxidation, nutrient utilization and energy expenditure (Extended Data Fig. 3b–h). Female *Vgat-Gipr* KO mice further show decreased blood glucose with normal fasting levels of plasma insulin, but improved glucose tolerance without changes in insulin sensitivity, HbA1c and plasma levels of triglycerides, cholesterol and free fatty acids (Extended Data Fig. 3i–p).

**Fig. 3:**
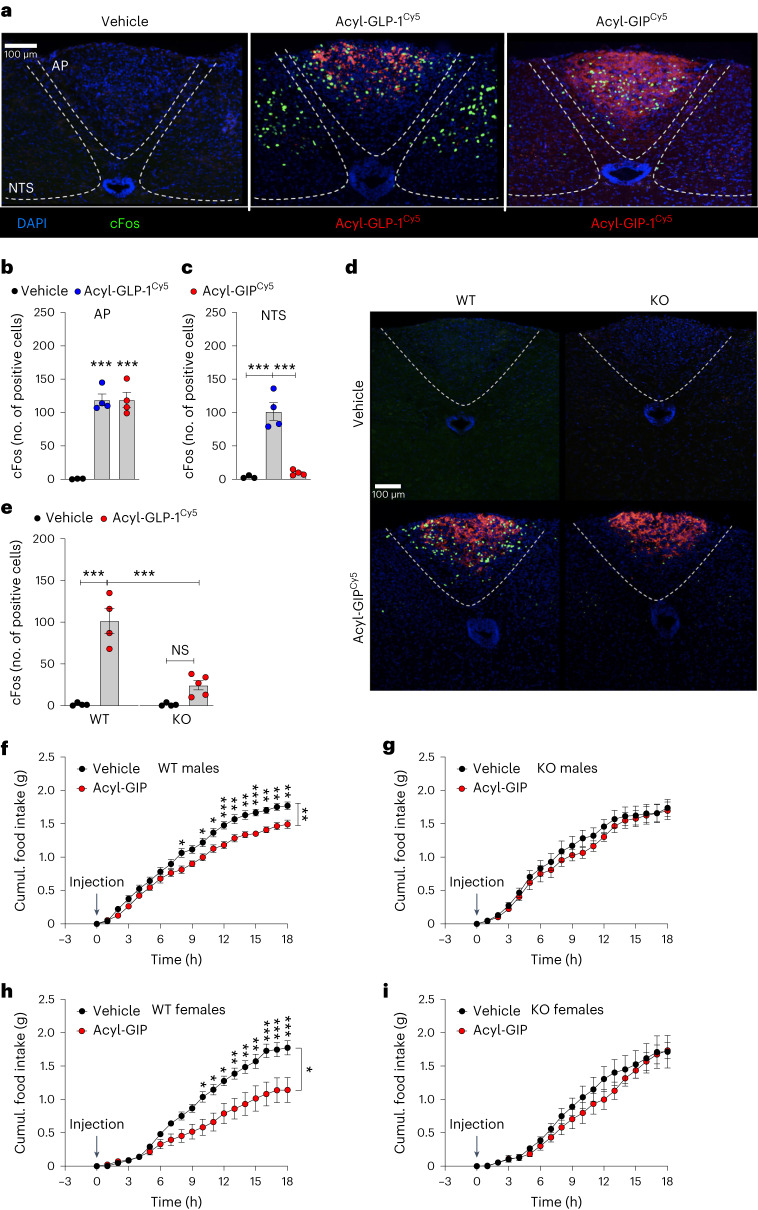
Acyl-GIP effects on cFos neuronal activation in the hindbrain and acute food intake in HFD-fed *Vgat-Gipr* KO mice. **a**–**c**, Representative image (**a**) and quantification (**b**,**c**) of cFos-positive neurons, as well as fluorescently (Cy5)-labeled drug appearance, in the area postrema (AP) and NTS of 52-week-old C57BL/6J WT mice treated with a single subcutaneous bolus (150 nmol kg^−1^) of either acyl-GLP-1^Cy5^ or acyl-GIP^Cy5^ (*n* = 3–4 mice each group). DAPI, 4,6-diamidino-2-phenylindole. **d**,**e**, Representative image (**d**) and quantification (**e**) of cFos-positive neurons, as well as Cy5-labeled drug appearance, in the area postrema of 26-week-old male C57BL/6J WT and *Vgat-Gipr* KO mice treated with a single subcutaneous dose of either vehicle or acyl-GIPCy5 (150 nmol kg^−1^; *n* = 4–5 each group) (**d**). **f**,**g**, Cumulative food intake in 35-week-old male C57BL/6J WT (**f**) and *Vgat-Gipr* KO (**g**) mice treated with a single subcutaneous dose of either vehicle or acyl-GIP (100 nmol kg^−1^, *n* = 7 each group). **h**,**i**, Cumulative food intake in 40-week-old female C57BL/6J WT (**h**) and *Vgat-Gipr* KO (**i**) mice treated with a single subcutaneous dose of either vehicle or acyl-GIP (100 nmol kg^−1^, *n* = 6–10 each group). Data in **b**,**c**,**e** were analyzed using an ordinary one-way ANOVA. Data in **f**–**i** were analyzed using repeated measures two-way ANOVA and a Bonferroni multiple comparison test for individual time points. Scale bars, 100 μm. Data are mean ± s.e.m.; NS, not significant; *P < 0.05; **P < 0.01 and ***P < 0.001. Individual *P* values are shown in the Source Data file, unless *P* < 0.0001. Source data

**Extended Data Fig. 4:**
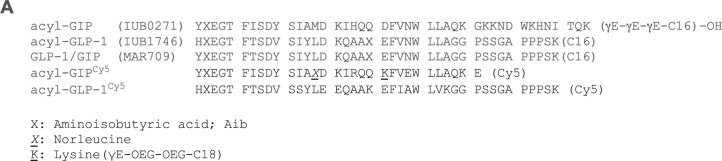
Sequence of the used GIP and GLP-1 analogs. Sequence of the used acyl-GIP, acyl-GLP-1, MAR709, acyl-GIP^Cy5^ and acyl-GLP-1^Cy5^ **(A)**.

**Extended Data Fig. 5:**
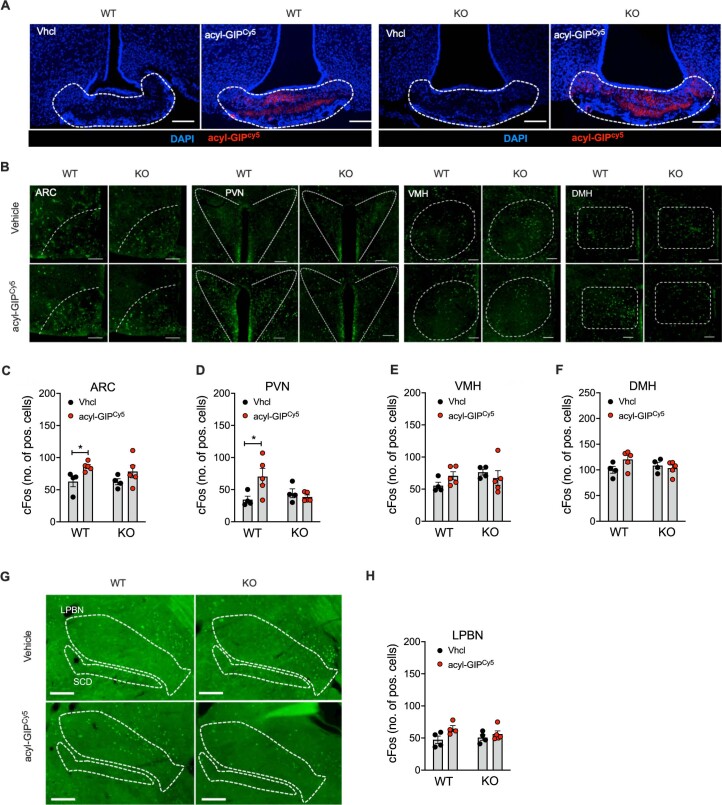
Acyl-GIP effects on cFos neuronal activation in the hypothalamus in HFD-fed *Vgat-Gipr* KO mice. Representative image of hypothalamic acyl-GIP^Cy5^ fluorescence (n = 3-5 each group) **(a)** and cFos neuronal activity in the arcuate nucleus (ARC), paraventricular nucleus (PVN), ventromedial hypothalamus (VMH) and dorsomedial hypothalamus (DMH) (n = 4-5 each group) **(b)** in 26-wk old male C57BL/6J wild-type (WT) and *Vgat-Gipr* (KO) mice treated subcutaneous (s.c.) with a single dose of either vehicle or acyl-GIP^Cy5^ (150 nmol/kg; n = 3-5 each group). Corresponding cFos quantification in the ARC (n = 4-5 each group) **(c)**, PVN (n = 4-5 each group) **(d)**, VMH (n = 4-5 each group) **(e)**, and DMH (n = 4-5 each group) **(f)**. CFos neuronal activity (G) and corresponding cFos quantification (H) in the lateral parabrachial nucleus (LPBN) in 26-wk old male C57BL/6J WT and *Vgat-Gipr* (KO) mice treated s.c. with a single dose of either vehicle or acyl-GIP^Cy5^ (150 nmol/kg; n = 4-5 each group). Data in panel **C-F,H** were analyzed using student’s 2-sided, 2-sided, 2-tailed t-test. Scale bars in panels **A** and **B** are 100 μm, scale bars in panel **G** are 200 μm. Data represent means ± SEM; asterisks indicate * p < 0.05. Individual p values are shown in the Data Source file. Source data

We found that *Vgat-Gipr* KO mice are resistant to central GIP effects on cFos neuronal activation and food intake. Based on recent data indicating that GIP acts on hindbrain GIPR neurons to regulate food intake^6^, we next assessed whether validated long-acting (fatty acid acylated) GLP-1R and GIPR agonists^2,3,8^ (Extended Data Fig. 4a) differ in their ability to induce neuronal activation in this area. After single subcutaneous administration, we find fluorescently labeled acyl-GLP-1^Cy5^ and acyl-GIP^Cy5^ to substantially accumulate in the area postrema (Fig. 3a); however, although we find acyl-GLP-1^Cy5^ to induce cFos neuronal activity in the area postrema and the nucleus tractus solitarius (NTS), acyl-GIP^Cy5^ induced cFos activity only in the area postrema, but not in the NTS (Fig. 3a–c). Notably, while acyl-GIP^Cy5^ in wild-type mice solidly induced cFos activity in the area postrema, this effect was largely blunted in the *Vgat-Gipr* KO mice, despite unchanged accumulation of acyl-GIP^Cy5^ in the area postrema (Fig. 3d,e). Preserved accumulation of acyl-GIP^Cy5^ in the area postrema of the *Vgat-Gipr* KO mice is not unexpected, given that *Vgat* is only expressed in ~18% of *Gipr* cells in the hindbrain, including for example oligodendrocytes and endothelial cells^14^ (Extended Data Table 1). Nonetheless, consistent with previous scRNA-seq data showing that *Vgat* is particularly enriched in *Gipr* neurons within the area postrema^11,13–15,17,21^ (Extended Data Table 1), these data hence demonstrate that the acyl-GIP^Cy5^-induced cFos activation in the area postrema is almost exclusively attributed to *Vgat*-expressing inhibitory GABAergic neurons. In wild-type and *Vgat-Gipr* KO mice, we also found that acyl-GIP^Cy5^, after a single subcutaneous administration, accumulates in the hypothalamic median eminence, along with increased cFos activation in the arcuate nucleus and paraventricular nucleus in wild-type mice, but not in *Vgat-Gipr* KO mice (Extended Data Fig. 5a–d). No changes were observed in cFos activation in the ventromedial and dorsomedial hypothalamus (Extended Data Fig. 5b,e,f) or the lateral parabrachial nucleus (Extended Data Fig. 5gh). We next assessed whether acyl-GIP differentially affects food intake in HFD-fed diet-induced obese (DIO) wild-type and *Vgat-Gipr* KO mice. While single subcutaneous bolus administration of acyl-GIP (100 nmol kg^−1^) acutely decreased food intake in male and female HFD-fed wild-type mice, administration of acyl-GIP failed to affect food intake in male and female *Vgat-Gipr* KO mice (Fig. 3f–i). These data hence indicate that acyl-GIP essentially requires GIPR signaling in *Vgat*-expressing inhibitory GABAergic neurons to acutely decrease food intake.

**Fig. 4:**
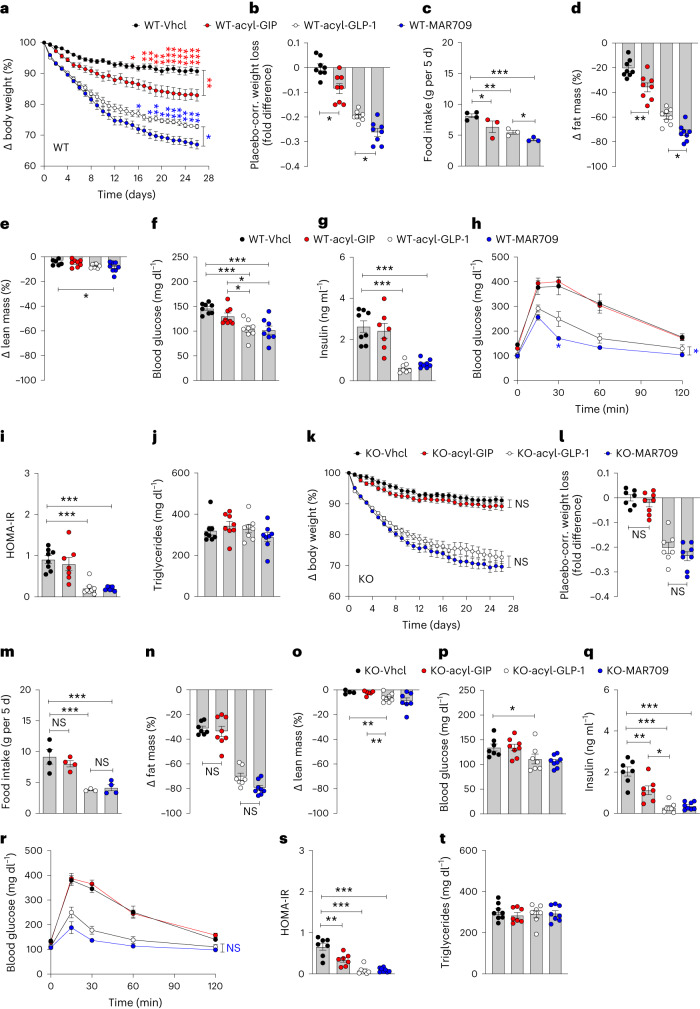
Effects of acyl-GIP, acyl-GLP-1 and MAR709 on body weight and glucose metabolism in HFD-fed *Vgat-Gipr* KO mice. **a**–**c**, Body weight development (**a**), placebo-corrected weight loss after 26 days of treatment (**b**), and food intake (**c**) of 38-week-old male C57BL/6J WT mice treated daily with vehicle (Vhcl), acyl-GIP (100 nmol kg^−1^) or 10 nmol kg^−1^ of either acyl-GLP-1 or MAR709 (*n* = 7–8 each group). **d**–**j**, Fat and lean tissue mass in 38-week-old male C57BL/6J WT mice (**d**,**e**), fasting levels of blood glucose and insulin in 38-week-old male C57BL/6J WT mice (**f**,**g**), glucose tolerance in 38-week-old male C57BL/6J WT mice (**h**), HOMA-IR in 38-week-old male WT mice (**i**) and plasma levels of triglycerides in 35-week-old male C57BL/6J WT mice (**j**) (*n* = 7–8 each group). **k**–**m**, Body weight development (**k**), placebo-corrected weight loss after 26 days of treatment (**l**) and food intake (**m**) of 35-week-old male C57BL/6J *Vgat-Gipr* KO mice treated daily with vehicle, acyl-GIP (100 nmol kg^−1^) or 10 nmol kg^−1^ of either acyl-GLP-1 or MAR709 (*n* = 7–8 each group). **n**–**t**, Fat and lean tissue mass in 35-week-old male C57BL/6J KO mice (**n**,**o**), fasting levels of blood glucose and insulin in 35-week-old male WT mice (**p**,**q**), glucose tolerance in 35-week-old male C57BL/6J WT mice (**r**), HOMA-IR in 35-week-old male WT mice (**s**) and plasma levels of triglycerides in 35-week-old male C57BL/6J WT mice (**t**) (*n* = 7–8 mice each group). Data in **a**,**h**,**k**,**r** were analyzed using repeated measures two-way ANOVA and with Bonferroni post hoc comparison for individual time points. Data in **c**,**m** were analyzed using Fishers LSD test. Data in **b**,**d**–**g**,**i**,**j**,**l**,**n**–**q**,**s**,**t** were analyzed using ordinary one-way ANOVA. Data in **g**,**i** were analyzed using a Student’s two-tailed *t*-test. Food intake in **c**,**m** was assessed per cage in double-housed mice. Cages with mice shredding food were excluded from the analysis. Data area mean ± s.e.m.; NS, not significant; *P < 0.05; **P < 0.01 and ***P < 0.001. Individual *P* values are shown in the Source Data file, unless *P* < 0.0001. Source data

**Extended Data Fig. 6:**
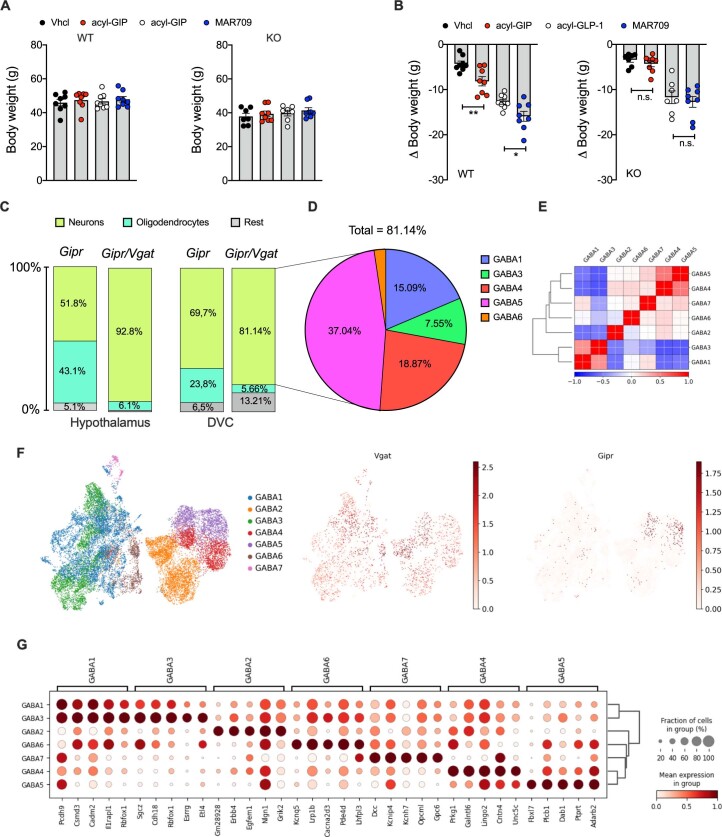
Effects of acyl-GIP, acyl-GLP-1 and MAR709 on absolute body weight in HFD-fed *Vgat-Gipr* KO mice, and analysis of *Vgat/Gipr* in published scRNA repositories. Starting body weight **(a)** and body weight change **(b)** in 38-wk old male C57BL/6J wild-type (WT) or *Vgat-Gipr* KO mice treated daily with either Vehicle, acyl-GIP (100 nmolkg), or 10 nmol/kg of either acyl-GLP-1 or MAR709 (n = 7-8 each group). Cell types expressing *Vgat* and*/*or *Gipr* in the hypothalamus and dorsal vagal complex (DVC) **(c)**. Distribution of cells co-expressing *Gipr* and *Vgat* within 7 clusters of GABAergic neurons identified in the DVC **(d)**. Correlation plots with Pearson’s correlations between 7 clusters (GABA1-7) of GABAergic neurons in the DVC **(e)**. UMAP of GABAergic neurons in the DVC colored by GABAergic neuron cluster, and expression of Vgat and Gipr in each cell, with the color corresponding to log-normalized UMI counts scaled to the maximum of each gene **(f)**. The dot plot of the top 5 genes most associated with each GABAergic neuron population as determined by Wilcoxon-rank-sum test **(g)**. Data in panel **A** were analyzed using ordinary 1-way ANOVA, data in panel **B** were analyzed using ordinary 1-way ANOVA and using Holm-Sidak multiple comparison test. Date represent means ± SEM; asterisks indicate * p < 0.05; ** p < 0.01 and *** p < 0.001. Individual p values are shown in the Data Source file, unless p < 0.0001. Data shown in panels C, D, E, F, G were generated based on publicly available snRNA Seq datasets of hypothalamus (Steuernagel et al.^15^) or DVC (Lugwig et al.^17^). Source data

We found that GIP and GIPR–GLP-1R co-agonism decreases body weight and food intake in mice via GIPR signaling in inhibitory GABAergic neurons. We assessed whether the body weight-lowering effect of acyl-GIP and GIPR–GLP-1R co-agonism depend on GIPR signaling in GABAergic neurons. In DIO wild-type mice, daily subcutaneous treatment with acyl-GIP (100 nmol kg^−1^) for 26 days significantly decreased body weight and food intake relative to vehicle controls (Fig. 4a–c and Extended Data Fig. 6a,b). The decrease in body weight in mice treated with acyl-GIP was accompanied by a decrease in fat but not lean tissue mass (Fig. 4d,e), without overt changes in blood glucose, insulin, glucose tolerance, insulin sensitivity or plasma levels of triglycerides (Fig. 4f–j). Consistent with the ability of acyl-GIP to decrease body weight and food intake in DIO wild-type mice (Fig. 4a–c), treatment with the GIPR–GLP-1R co-agonist MAR709 led to greater weight loss and further decrease in food intake and fat mass relative to mice treated with acyl-GLP-1 (Fig. 4a–d). In DIO wild-type mice, MAR709 and acyl-GLP-1 equally decreased fasting levels of blood glucose and insulin (Fig. 4f–g), but with superiority of MAR709 over acyl-GLP-1 to improve glucose tolerance (Fig. 4h). Insulin sensitivity, as estimated by HOMA-IR, was equally improved by MAR709 and acyl-GLP-1 (Fig. 4i), without effects of either treatment on plasma levels of triglycerides (Fig. 4j).

In contrast to DIO wild-type mice, treatment with acyl-GIP in *Vgat-Gipr* KO mice failed to affect body weight, food intake or body composition relative to vehicle controls (Fig. 4k–o). Treatment of *Vgat-Gipr* KO mice with acyl-GIP had no effect on blood glucose despite a slight decrease in fasting insulin levels (Fig. 4p,q). *Vgat-Gipr* KO mice treated with acyl-GIP show no difference in glucose control relative to vehicle controls, but display improved insulin sensitivity, as estimated by HOMA-IR (Fig. 4r,s), without changes in plasma triglycerides (Fig. 4t). Consistent with the demonstration that acyl-GIP loses its ability to decrease body weight and food intake in *Vgat-Gipr* KO mice (Fig. 4k–m), we found that weight loss induced by MAR709 was indistinguishable from that under acyl-GLP-1 treatment in *Vgat-Gipr* KO mice (Fig. 4k,l). The observed superiority of MAR709 over acyl-GLP-1 to further inhibit food intake in wild-type mice (Fig. 4c) likewise vanished in the *Vgat-Gipr* KO mice (Fig. 4m). MAR709 and acyl-GLP-1 further equally decreased fat mass, as well as fasting levels of blood glucose and insulin in *Vgat-Gipr* KO mice (Fig. 4o–q), with nearly identical improvements in glucose tolerance and insulin sensitivity (Fig. 4e,s) and unchanged plasma levels of triglycerides (Fig. 4t). In summary, these data show that GIPR signaling in inhibitory GABAergic neurons is essential for the ability of acyl-GIP to decrease body weight and food intake and also, for the superior metabolic effects of the GIPR–GLP-1R co-agonist MAR709 over a matched acyl-GLP-1 control. Our data are consistent with our previous report demonstrating that acyl-GIP and MAR709 act in the brain to regulate body weight and food intake via central nervous system GIPR signaling^3^. Furthermore, our data corroborate that GIPR signaling is a genuine contributor to the metabolic efficacy of GIPR–GLP-1R co-agonism, in that it drives greater weight loss and further inhibition of food intake relative to GLP-1R agonism alone. Notably, our data are further in line with the recent demonstration that a long-acting GIPR agonist decreases body weight in healthy humans^25^.

We here show that long-acting GIPR agonists and the GIPR–GLP-1R co-agonist MAR709 decrease body weight and food intake in DIO mice via GIPR signaling in inhibitory GABAergic neurons. Limitations to our study include the lack of publicly available antibodies to reliably detect GIPR, which would be of value to further delineate the neuronal mechanisms by which GIP regulates energy metabolism in GABAergic neurons. Such antibodies would have also been useful to demonstrate lack of co-staining between VGAT and GIPR in *Vgat-Gipr* KO mice and to further characterize the *Vgat*/*Gipr* coexpressing neurons. Published scRNA repositories^14,16,17,21^, however, show that ~81–93% of the *Vgat*/*Gipr-*expressing cells in the hypothalamus and the dorsal vagal complex (DVC) are neurons (Extended Data Fig. 6c) and that *Vgat*/*Gipr* coexpressing neurons in the DVC belong to five out of seven GABAergic neuron clusters with ~37% and ~19% localizing to clusters GABA5 and GABA4, respectively (Extended Data Fig. 6d–g).

Further limitations of our studies include that we cannot exclude the potential for variable long-acting GIPR agonists to differ in central GIPR signaling based on differences in pharmacokinetics and pharmacodynamics. Differences in pharmacokinetics might also explain why we see that acyl-GIP^Cy5^ restricted cFos activation in the area postrema, whereas other studies using different GIPR agonists found cFos activation in the NTS^6,11^. Although unlikely, the possibility that the pharmacokinetics of the acyl-GIP that we used slightly differs from acyl-GIP^Cy5^ may be a similar consideration. Another limitation is that the HFD-fed wild-type and *Vgat-Gipr* KO mice had a strong tendency to shred food, which prevented deeper analysis of eating behavior, including assessment of cumulative food intake and meal patterns. Notably, the demonstrated low (~14–18%) abundance of *Vgat* in *Gipr* cells using scRNA-seq analysis may be an underestimate due to limitations in complementary DNA library preparations or due to the general difficulty to adequately detect low-abundant transcripts. Finally, clarification is needed on whether acyl-GIP and GIPR–GLP-1R co-agonists decrease body weight and food intake exclusively via hindbrain GABAergic neurons or whether also GABAergic GIPR neurons in other brain regions are required for the effects on body weight and food intake shown in this study.

## Methods

### Animals and housing conditions

Experiments were performed in accordance with the Animal Protection Law of the European Union after permission by the Government of Upper Bavaria. Mice were fed ad libitum with either chow diet (1314, Altromin) or HFD (D12331, Research Diets) and were kept at 22 ± 2 °C with constant humidity (45–65%) and a 12-h light–dark cycle. C57BL/6J *Vgat-ires-cre* knock-in mice were purchased from The Jackson Laboratory (028862). *Gipr*^flx/flx^ mice^22,23^ were crossed with *Vgat-cre*^+/−^ mice to obtain *Vgat-cre*^+/−^ *Gipr*^flx+/flx+^ (*Vgat-Gipr* KO). *Vgat-cre*^+/−^*Gipr*^flx-/flx-^ were used as WT controls. For metabolic phenotyping and assessment of drug effects, age-matched mice were grouped based on genotype and double-housed at room temperature (22 ± 2 °C). For the examination of drug effects, mice were fed a HFD approximately 20 weeks before the start of the studies. Body composition was analyzed using a magnetic resonance whole-body composition analyzer (EchoMRI). For pharmacological studies, mice were treated for the indicated time duration with either acyl-GIP (100 nmol kg^−1^) or 10 nmol kg^−1^ of acyl-GLP-1 or MAR709 (Extended Data Fig. 1a).

### Drug development

Commercially available maleimide-functionalized fluorophores were conjugated to the free cysteine residue of the appropriate peptide. For Cy5-labeled compounds, the peptide was dissolved to 3–5 mM in dimethylsulfoxide containing 0.5–2.5 equivalent EDTA and 1–2.5 equivalent of reductant (Tris(2-carboxyethyl)phosphine or bis(p-sulfonatophenyl)phenylphosphine). The pH was adjusted above pH 7 with eg 1% *v*/*v* DIPEA or 0.25 M phosphate, pH 7.4, followed by the addition of 1.3 equivalent of solid sulfo-cyanine3 maleimide, overnight stirring at room temperature and purification by reversed-phase HPLC. For Cy5-labeled compounds, the peptide was dissolved to 1–2 mM in PBS, pH 7.4, containing ~3 equivalent tris(2-carboxyethyl)phosphine. The pH was adjusted above pH 7 with 1 M NaOH, followed by the addition of 3 equivalent solid sulfo-cyanine5 maleimide, stirring for 30 min at room temperature and purification by reversed-phase HPLC.

### Fasting glucose, insulin, ipGTT and ipITT

Fasting levels of blood glucose and insulin were measured in 6-h fasted mice. Insulin was measured by Ultra-Sensitive Mouse Insulin ELISA kit (90080, Crystal Chem) according to the manufacturer’s instructions. For assessment of glucose tolerance, glucose was administered intraperitoneally at a dose of 1.75 g kg^−1^. For assessment of insulin tolerance, insulin (Humalog, Eli Lilly and Co.) was injected intraperitoneally at a dose of 0.75 UI kg^−1^ (HFD-fed mice) or 0.5 UI kg^−1^ (chow-fed mice).

### Bomb calorimetry

Assimilated energy and assimilation efficiency were assessed using the C200 Oxygen Bomb Calorimeter (IKA). Feces were collected over 7 days, dried at 65 °C until weight was consistent, before measuring the food/fecal energy content.

### Indirect calorimetry

Energy expenditure, food intake, RER, FA oxidation and locomotor activity were assessed for 3–4 consecutive days in single-house mice using a climate-controlled indirect calorimetric system (TSE Phenomaster, TSE Systems). Mice were given a 24-h acclimatization phase before the start of the measurements. Data for energy expenditure were analyzed using ANCOVA with body weight as a covariate as previously suggested^26,27^. FA oxidation (kcal h^−1^) was calculated by the formula, energy expenditure (kcal h^−1^) × (1 − RER)/0.3 (ref. ^28^).

### RNA extraction and gene expression analysis

For qPCR analysis, RNA was isolated using the RNeasy kit (74106, QIAGEN) according to the manufacturer’s instructions. cDNA was synthesized using the QuantiTect RT kit (205311, QIAGEN) according to the manufacturer’s instructions. Quantitative PCR was performed in two or three technical replicates per sample, using SYBR green (4309155, Thermo Fisher Scientific) using the Applied Biosystems QuantStudio 6 or 7 (Thermo Fisher Scientific). The following primers were used: *Pomc:* 5′-CATTAGGCTTGGAGCAGGTC-3′ and 5′-TCTTGATGATGGCGTTCTTG-3′; *Cartpt:* 5′-CGAGAAGAAGTACGGCCAAG-3′ and 5′-GGAATATGGGAACCGAAGGT-3′; *Npy:* 5′-TGGACTGACCCTCGCTCTAT-3′ and 5′-TGTCTCAGGGCTGGATCTCT-3′; *Agrp:* 5′-GGCCTCAAGAAGACAACTGC-3′ and 5′-GCAAAAGGCATTGAAGAAGC-3′); *Gipr* 5′-GTGTCCACGAGGTGGTGTTT-3′ and 5′-CCGACTGCACCTCTTTGTTG-3′; *Hprt:* 5′-AAGCTTGCTGGTGAAAAGGA-3′ and 5′-TTGCGCTCATCTTAGGCTTT-3′; *Ppia:* 5′-GAGCTGTTTGCAGACAAAGTTC-3′ and 5′-CCCTGGCACATGAATCCTGG-3′; *Sst*: 5′-GAGCCCAACCAGACAGAGAA-3′ and 5′-CCTCATCTCGTCCTGCTCA-3′; *Avp:* 5′-ACTACGCTCTCCGCTTGTTT-3′ and 5′-CAGCAGATGCTTGGTCCGAA-3′; *Tac1:* 5′-CGCACCTGCGGAGCAT-3′ and 5′-CTCAAAGGGCTCCGGCATT-3′; *Pthlh*: 5′-AGAAGCGAAGGACTCGGTCT-3′ and 5′-CCTGTAACGTGTCCTTGGAAGA-3′; *Aplp1*: 5′-CTTCAGGTGATCGAAGAGCGA-3′ and 5′-GGAGGCTACCTTTGTCCTCA-3′; *Cst3:* 5′-CGCCATACAGGTGGTGAGAG-3′ and 5′-GGCTGGTCATGGAAAGGACA-3′; *Cck*: 5′-ACTGCTAGCGCGATACATCC-3′ and 5′-CATCCAGCCCATGTAGTCCC-3′; *Hcrt*: 5′-TCCTGCCGTCTCTACGAACT-3′ and 5′-TGGTTACCGTTGGCCTGAAG-3′; *Oxt:* 5′-CTGTGCTGGACCTGGATATGCG-3′ and 5′-AGCTCGTCCGCGCAGCAGATG-3′; *Glp1r*: 5′-AGCACTGTCCGTCTTCATCA-3′ and 5′-AGAAGGCCAGCAGTGTGTAT-3′; *Actb*: 5′-TTGCTGACAGGATGCAGAAG-3′ and 5′-ACATCTGCTGGAAGGTGGAC-3′. Target gene expression was assessed using the ΔΔ*C*_t_ method^29^. The expression level of each gene was normalized to the housekeeping genes *Hprt, Ppia*, *Gapdh*, *Actb* or *Tbp*, depending on which genes showed the lowest variability across genotypes in the respective tissue.

### Immunofluorescence

For assessment of cFos, mice were habituated to a daily injection by subcutaneous saline administration for three constitutive days. On day 4, mice were treated subcutaneous with a single dose of acyl-GIP^Cy5^ or acyl-GLP-1^Cy5^ (150 nmol kg^−1^). The mice were exposed to CO_2_ overdose 90 min after drug exposure and were briefly perfused with ice-cold TBS following by buffered 4% formaldehyde. Twenty-four hours after fixation, brains were coronally cryosectioned and 35-μm-thick slices were immunolabelled with the monoclonal rabbit anti-cFos antibody (MA5-15055, Invitrogen, 1:400 dilution) and the anti-rabbit Alexa546 secondary antibody (A10040, Invitrogen, 1:2,000 dilution). According to the Allen Mouse Brain Atlas, the DVC containing the area postrema and NTS was captured by ×20 objective in *z*-stack mode using a Leica SP8 confocal microscope. In each region, the number of cFos-positive cells was automatically counted in using Fiji/ImageJ software. For cFos quantification, whole brain slices were scanned in z-stack mode with AxioScan 7 digital slide scanner (Zeiss, ZEN Blue v.3.5, ×20 objective) and imaged using LAS X (v.3.5.7.23225, Leica Microsystems). The Allen Brain Atlas was imputed and aligned to the whole slide images using Fiji with the ABBA plugin and the number of cFos-positive cells in each identified region was measured by using QuPath v.0.4.4 software^30^.

### Immunohistochemistry for α- and β-cell volume and islet size

Pancreata were fixed in 10% formalin (HT501128, Sigma-Aldrich) for 24 h at room temperature and processed for paraffin embedding (Tissue Tec VIP.6, Sakura Europe). Paraffinized pancreata were exhaustively cross-sectioned into 3–4 parallel, equidistant slices per case. Maintaining their orientation, the tissue slices were vertically embedded in paraffin. After co-staining for insulin (monoclonal rabbit anti-insulin, 3014, Cell Signaling 1:800 dilution; AlexaFluor750-conjugated goat anti-rabbit, A21039, Invitrogen 1:100 dilution) and for glucagon (polyclonal guinea pig anti-glucagon, M182, Takara 1:3,000 dilution; goat anti-guinea pig AF555, A21435, Invitrogen 1:200 dilution) nuclei were labeled with Hoechst33342 (H1399, Thermo Fisher, 7.5 µg ml^−1^). The stained tissue sections were scanned with an AxioScan 7 digital slide scanner (Zeiss, ZEN Blue v.3.5) equipped with a ×20 magnification objective. Quantification of insulin- or glucagon-expressing cells was performed on the entire tissue sections using the image analysis software Visiopharm. The insulin- or glucagon-expressing cells were classified automatically using the fluorescence intensity of each hormone. The β-cell volume (mg) was calculated by multiplying the detected relative insulin-positive cell area by total pancreatic weight. The α-cell volume (mg) was similarly calculated based on the detected glucagon-positive cell area. The area of the pancreatic islet was calculated based on the insulin and glucagon-positive area.

### Islet isolation

Islets were isolated via collagenase P perfusion of the pancreas^31^. In brief, collagenase P solution (1 mg ml^−1^, Roche) was injected through the ampulla of Vater and pancreata were digested at 37.5 °C for 12 min. Digestion was stopped by addition of ice-cold HBSS (Thermo Fisher), including 0.05% (*w*/*v*) BSA (Sigma-Aldrich). The tissue suspension was centrifuged and islets were purified by density gradient purification using 15% OptiPrep density gradient medium (Sigma-Aldrich). Islets in the visible density gradient layer were collected, rinsed with HBSS and incubated in complete RPMI-1640 medium (Gibco) at 37 °C with 5 % CO_2_.

### Single-cell RNA-seq analysis

The published RNA-seq datasets^14–17,21^ were analyzed using Scanpy (v.2.11.0)^32^, Seurat v.5 (ref. ^33^) or CELLxGENE (v.1.1.2) (Chan Zuckerberg Initiative). The authors’ original pre-processing and cell-type annotations were adopted without any changes. Only cells with at least one unique molecular identifier were considered in the analysis.

### Serum analysis

Blood was collected by cardiac puncture during organ withdrawal, stored on ice and centrifuged (2,500*g* for 10 min at 4 °C) for serum separation and collection. The levels of leptin (KMC2281, Leptin mouse ELISA, Invitrogen), GIP (EZRMGIP-55K, rat/mouse GIP ELISA, Merck Millipore) and GLP-1 (1508, Mouse GLP-1 ELISA, Crystal Chem) were measured according to the manufacturer’s instructions.

### Histological analysis

Excised samples were fixed in 4% (w/v) neutrally buffered formalin, embedded in paraffin and cut into 3-µm slices for hematoxylin and eosin and scanned with an AxioScan 7 digital slide scanner (Zeiss) equipped with a ×20 magnification objective. Steatosis was graded by the presence of fat vacuoles in liver cells according to the percentage of affected tissue (0, <5%; 1, 5–33%; 2, 33–66%; and 3, >66%). Lobular inflammation was scored by overall assessment of inflammatory foci per ×200 field (0, no foci; 1, <2 foci; 2, 2–4 foci; and 3, >4 foci). The morphometric quantification of mean size of adipocytes was performed using the commercially available image analysis software Visiopharm (v. 2018.9; Visiopharm).

### Statistical analysis

Statistical analyses were performed using GraphPad Prism v.9 and SPSS v.28.0.1.1. Analysis of energy expenditure were performed using ANCOVA with body weight as a covariate as previously suggested^26,27^. For each analysis, the statistical tests and sample sizes are indicated in the figure legends. *P* < 0.05 was considered statistically significant. All data represent mean ± s.e.m.

### Reporting summary

Further information on research design is available in the Nature Portfolio Reporting Summary linked to this article.

### Supplementary information

Reporting Summary

Supplementary Data Original pictures of cFos and Cy5 drug appearance shown in Extended Data Fig. 5a–h, including replicates used for quantification.

Supplementary Data Original pictures of cFos and Cy5 drug appearance shown in Fig. 3, including replicates used for quantification.

### Source data

Source Data Fig. 1 Statistical Source Data for Fig. 1.

Source Data Fig. 2 Statistical Source Data for Fig. 2.

Source Data Fig. 3 Statistical Source Data for Fig. 3.

Source Data Fig. 4 Statistical Source Data for Fig. 4.

Source Data Extended Data Fig. 1 Statistical Source Data for Extended Data Fig. 1.

Source Data Extended Data Fig. 2 Statistical Source Data for Extended Data Fig. 2.

Source Data Extended Data Fig. 3 Statistical Source Data for Extended Data Fig. 3.

Source Data Extended Data Fig. 5 Statistical Source Data for Extended Data Fig. 5.

Source Data Extended Data Fig. 6 Statistical Source Data for Extended Data Fig. 6.

## Figures and Tables

**Extended Data Table 1:**
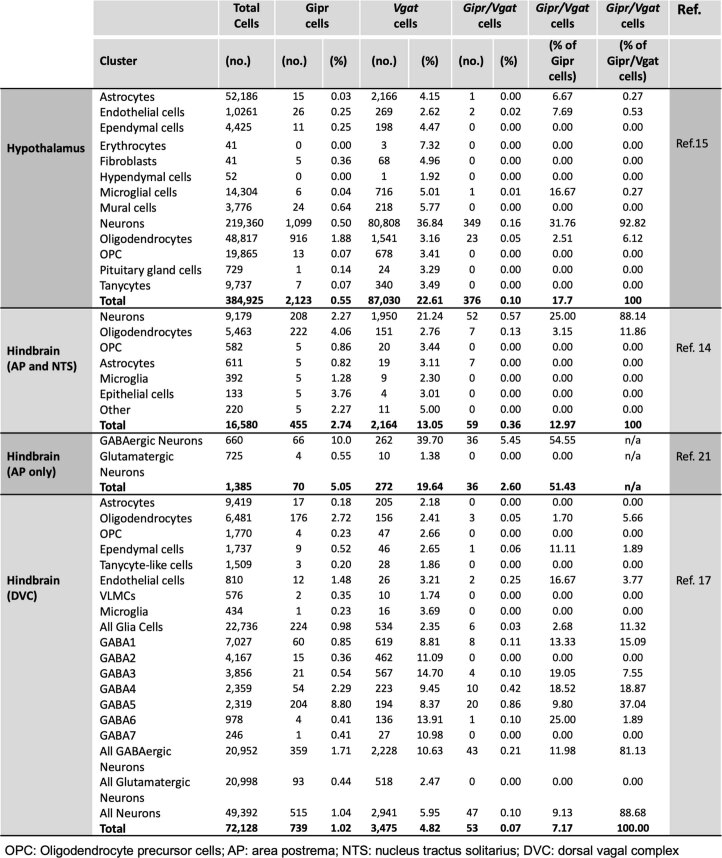
Overview of hypothalamic and hindbrain Gipr and Vgat expression based on published murine scRNA-seq studies

## Data Availability

The data used for the statistical analysis are available in source data files, along with the GraphPad Prism-derived statistical reports as appropriate, which contain the mean difference between the treatment groups, 95% confidence intervals, the significance summary and exact *P* values (unless *P* < 0.0001). The used scRNA-seq datasets are available via the Gene Expression Omnibus under accession codes GSE160938, GSE166649 and GSE168737. The HypoMap is available in an interactive CELLxGENE viewer (https://www.mrl.ims.cam.ac.uk). The corresponding Seurat object is deposited at University of Cambridge’s Apollo Repository (10.17863/CAM.87955). Other used databases include the Allen Mouse Atlas. All raw images are provided in the source data files, with the exception of the histology pictures for Extended Data Figs. 1m–s and 2d–f, which were too large for public repositories; due to the large file size of these pictures, they are only available upon request. Source data are provided with this paper.

## References

[1] Coskun T (2018). LY3298176, a novel dual GIP and GLP-1 receptor agonist for the treatment of type 2 diabetes mellitus: From discovery to clinical proof of concept. Mol. Metab..

[2] Finan B (2013). Unimolecular dual incretins maximize metabolic benefits in rodents, monkeys, and humans. Sci. Transl. Med.

[3] Zhang Q (2021). The glucose-dependent insulinotropic polypeptide (GIP) regulates body weight and food intake via CNS-GIPR signaling. Cell Metab..

[4] Heise T (2022). Effects of subcutaneous tirzepatide versus placebo or semaglutide on pancreatic islet function and insulin sensitivity in adults with type 2 diabetes: a multicentre, randomised, double-blind, parallel-arm, phase 1 clinical trial. Lancet Diabetes Endocrinol..

[5] Frias JP (2021). Tirzepatide versus semaglutide once weekly in patients with type 2 diabetes. N. Engl. J. Med..

[6] Adriaenssens, A. et al. Hypothalamic and brainstem glucose-dependent insulinotropic polypeptide receptor neurons employ distinct mechanisms to affect feeding. *JCI Insight*10.1172/jci.insight.164921 (2023). 10.1172/jci.insight.164921 PMC10322681 37212283

[7] Adriaenssens AE (2019). Glucose-dependent insulinotropic polypeptide receptor-expressing cells in the hypothalamus regulate food intake. Cell Metab..

[8] Mroz PA (2019). Optimized GIP analogs promote body weight lowering in mice through GIPR agonism not antagonism. Mol. Metab..

[9] Muller TD, Bluher M, Tschop MH, DiMarchi RD (2022). Anti-obesity drug discovery: advances and challenges. Nat. Rev. Drug Discov..

[10] El K (2023). The incretin co-agonist tirzepatide requires GIPR for hormone secretion from human islets. Nat. Metab..

[11] Borner T (2021). GIP receptor agonism attenuates GLP-1 receptor agonist-induced nausea and emesis in preclinical models. Diabetes.

[12] Zhang C, Vincelette LK, Reimann F, Liberles SD (2022). A brainstem circuit for nausea suppression. Cell Rep..

[13] Borner T (2023). GIP receptor agonism blocks chemotherapy-induced nausea and vomiting. Mol. Metab..

[14] Dowsett GKC (2021). A survey of the mouse hindbrain in the fed and fasted states using single-nucleus RNA sequencing. Mol. Metab..

[15] Steuernagel L (2022). HypoMap-a unified single-cell gene expression atlas of the murine hypothalamus. Nat. Metab..

[16] Ludwig MQ, Todorov PV, Egerod KL, Olson DP, Pers TH (2021). Single-cell mapping of GLP-1 and GIP receptor expression in the dorsal vagal complex. Diabetes.

[17] Ludwig MQ (2021). A genetic map of the mouse dorsal vagal complex and its role in obesity. Nat. Metab..

[18] Jennings JH (2015). Visualizing hypothalamic network dynamics for appetitive and consummatory behaviors. Cell.

[19] Marino RAM (2020). Control of food approach and eating by a GABAergic projection from lateral hypothalamus to dorsal pons. Proc. Natl Acad. Sci. USA.

[20] Russo FM, De Bie FR, Partridge EA, Allegaert K, Deprest J (2022). The antenatal sildenafil STRIDER trial for severe fetal growth restriction, are post hoc reflections ad rem?. Eur. J. Pediatr..

[21] Zhang C (2021). Area postrema cell types that mediate nausea-associated behaviors. Neuron.

[22] Campbell JE (2016). TCF1 links GIPR signaling to the control of β cell function and survival. Nat. Med.

[23] Ussher JR (2018). Inactivation of the glucose-dependent insulinotropic polypeptide receptor improves outcomes following experimental myocardial infarction. Cell Metab..

[24] Miyawaki K (2002). Inhibition of gastric inhibitory polypeptide signaling prevents obesity. Nat. Med.

[25] Knop, F. K. et al. A long-acting glucose dependent insulinotropic polypeptide receptor agonist shows weight loss without nausea or vomiting. In *American Diabetes Association – 83rd Annual Scientific Sessions*; San Diego, CA, USA; 23–26 June 2023 Poster 56-OR (ADA, 2023).

[26] Muller TD, Klingenspor M, Tschop MH (2021). Revisiting energy expenditure: how to correct mouse metabolic rate for body mass. Nat. Metab..

[27] Tschop MH (2011). A guide to analysis of mouse energy metabolism. Nat. Methods.

[28] Liskiewicz D (2023). Neuronal loss of TRPM8 leads to obesity and glucose intolerance in male mice. Mol. Metab..

[29] Livak KJ, Schmittgen TD (2001). Analysis of relative gene expression data using real-time quantitative PCR and the 2-^ΔΔCT^method. Methods.

[30] Bankhead P (2017). QuPath: open source software for digital pathology image analysis. Sci. Rep..

[31] Li DS, Yuan YH, Tu HJ, Liang QL, Dai LJ (2009). A protocol for islet isolation from mouse pancreas. Nat. Protoc..

[32] Wolf FA, Angerer P, Theis FJ (2018). SCANPY: large-scale single-cell gene expression data analysis. Genome Biol..

[33] Satija R, Farrell JA, Gennert D, Schier AF, Regev A (2015). Spatial reconstruction of single-cell gene expression data. Nat. Biotechnol..

