# Supplementary material for: Glucose-dependent insulinotropic polypeptide regulates body weight and food intake via GABAergic neurons in mice
Source: Nat Metab. 2023 Nov 9;5(12):2075–85. doi: 10.1038/s42255-023-00931-7 (PMC10730394; doi:10.1038/s42255-023-00931-7)
Supplement: Supplementary file 1 — Reporting Summary [file 42255_2023_931_MOESM1_ESM.pdf]

nature portfolio

Corresponding author(s): Timo D. Müller

Last updated by author(s): Oct 9, 2023

Reporting Summary

Nature Portfolio wishes to improve the reproducibility of the work that we publish. This form provides structure for consistency and transparency in reporting. For further information on Nature Portfolio policies, see our Editorial Policies and the Editorial Policy Checklist.

Statistics

For all statistical analyses, confirm that the following items are present in the figure legend, table legend, main text, or Methods section.

n/a	Confirmed	
☐	☒	The exact sample size (n) for each experimental group/condition, given as a discrete number and unit of measurement
☐	☒	A statement on whether measurements were taken from distinct samples or whether the same sample was measured repeatedly
☐	☒	The statistical test(s) used AND whether they are one- or two-sided<br>*Only common tests should be described solely by name; describe more complex techniques in the Methods section.*
☐	☒	A description of all covariates tested
☐	☒	A description of any assumptions or corrections, such as tests of normality and adjustment for multiple comparisons
☐	☒	A full description of the statistical parameters including central tendency (e.g. means) or other basic estimates (e.g. regression coefficient) AND variation (e.g. standard deviation) or associated estimates of uncertainty (e.g. confidence intervals)
☐	☒	For null hypothesis testing, the test statistic (e.g. F, t, r) with confidence intervals, effect sizes, degrees of freedom and P value noted<br>*Give P values as exact values whenever suitable.*
☒	☐	For Bayesian analysis, information on the choice of priors and Markov chain Monte Carlo settings
☒	☐	For hierarchical and complex designs, identification of the appropriate level for tests and full reporting of outcomes
☐	☒	Estimates of effect sizes (e.g. Cohen's d, Pearson's r), indicating how they were calculated

Our web collection on statistics for biologists contains articles on many of the points above.

Software and code

Policy information about availability of computer code

Data collection	cFOS was imaged using LAS X (version 3.5.7.23225, Leica Microsystems CMS GmbH)
Data analysis	Statistical analyses were performed using GraphPad Prism version 9 and SPSS version 28.0.1.1. cFOS data were analyzed using QuPath (version 0.4.4). Single cell RNAseq analysis were analyzed using Scranpy (version 2.11.0), Seurat (v.5) or CellxGene (v. 1.1.2). Histological analysis were performed using Visiopharm (v. 2018.9)

For manuscripts utilizing custom algorithms or software that are central to the research but not yet described in published literature, software must be made available to editors and reviewers. We strongly encourage code deposition in a community repository (e.g. GitHub). See the Nature Portfolio guidelines for submitting code & software for further information.

Data

Policy information about availability of data

All manuscripts must include a data availability statement. This statement should provide the following information, where applicable:

- Accession codes, unique identifiers, or web links for publicly available datasets
- A description of any restrictions on data availability
- For clinical datasets or third party data, please ensure that the statement adheres to our policy

The data used for the statistical analysis are available in the data source file, along with the GraphPad Prizm-derived report on the statistical analysis as appropriate. The statistical report contains the mean difference between the treatment groups, the 95% confidence intervals, the significance summary, and the exact P values (unless P < 0.0001). The scRNAseq datasets used in the study are available via the GEO accession #GSE160938, #GSE166649 and #GSE168737. The used HypoMap is available in an interactive CellxGene viewer (https://www.mrl.ims.cam.ac.uk); the corresponding Seurat object is deposited at University of Cambridge's Apollo Repository (10.17863/CAM.87955). Other used databases include the Allen mouse atlas. All raw images are provided in the data source files, with exception of the

histology pictures for Extended Data Figures 1M-S and 2D-F, which were too large for public repositories. Due to the file size of these pictures, they are only available upon request.

Field-specific reporting

Please select the one below that is the best fit for your research. If you are not sure, read the appropriate sections before making your selection.

☒ Life sciences ☐ Behavioural & social sciences ☐ Ecological, evolutionary & environmental sciences

For a reference copy of the document with all sections, see nature.com/documents/nr-reporting-summary-flat.pdf

Life sciences study design

All studies must disclose on these points even when the disclosure is negative.

Sample size	For animal studies, sample sizes were calculated based on a power analysis assuming that a greater or equal (>/=) 5 g difference in body weight between genotypes can be assessed with a power of >/= 75% when using a 2-sided statistical test under the assumption of a standard deviation of 3.5 and an alpha level of 0.05.
Data exclusions	No data were excluded from the analysis unless scientific (e.g. significant outlier identified by the Grubbs test for outlier) or animal welfare reasons (e.g. injury due to fighting) demanded exclusion. Outliers are stated in the data source file.
Replication	In vivo and ex vivo data were obtained in independent biological replicates as indicated in the figure legends.
Randomization	Animals were either randomly assigned into treatment groups, or were grouped based on their genotype (WT or KO). At study start, only age-matched mice were included in the studies. There were no other covariats controlled.
Blinding	For in vivo studies, drugs were aliquoted by a lead scientist in number-coded vials and most, but not all, handling investigators were blinded to the treatment condition. Analyses of glucose and insulin tolerance were performed by experienced research assistants which did not know prior treatment conditions. Ex vivo studies were performed in ID coded vials without statement of treatment on the vials. Ex vivo studies were performed in ID coded vials, and with with most, but not all investigators, being blinded to the underlying genotypes.

Reporting for specific materials, systems and methods

We require information from authors about some types of materials, experimental systems and methods used in many studies. Here, indicate whether each material, system or method listed is relevant to your study. If you are not sure if a list item applies to your research, read the appropriate section before selecting a response.

Materials & experimental systems

n/a	Involved in the study	
☐	☒	Antibodies
☒	☐	Eukaryotic cell lines
☒	☐	Palaeontology and archaeology
☐	☒	Animals and other organisms
☒	☐	Human research participants
☒	☐	Clinical data
☒	☐	Dual use research of concern

Methods

n/a	Involved in the study	
☒	☐	ChIP-seq
☒	☐	Flow cytometry
☒	☐	MRI-based neuroimaging

Antibodies

Antibodies used	cFOS (Invitrogen, #MA5-15055; Dilution 1:400)<br>anti-rabbit Alexa546 (Invitrogen, #A10040; Dilution1:2,000)<br>anti-insulin (Cell Signaling, #3014, Dilution 1:800)<br>AlexaFluor750-conjugated goat anti-rabbit (Invitrogen, A21039, Dilution 1:100)<br>anti-glucagon (Takara, M182, Dilution 1:3000)<br>goat anti-guinea pig AF555 (Invitrogen, A21435, Dilution 1:2000)
Validation	cFOS (Invitrogen, #MA5-15055): The cFOS monoclonal antibody Invitrogen #MA5-15055 was verified by Relative expression to ensure that the antibody binds to the antigen stated. The antibody shows reactivity in bovine, hamster, human, mouse, pig and rat. The antibody can be used for western blot, immunhistochemistry, immuncytochemistry, flow cytometry and ChIP assays. The antibody does not cross-react with other Fos proteins, including FosB, FRA1 and FRA2. Immunofluorescence analysis of c-Fos was performed using 70% confluent log phase HeLa cells treated with 200 ng/mL EGF for 30 min. The cells were fixed with 4% paraformaldehyde for 10 minutes, permeabilized with 0.1% Triton™ X-100 for 10 minutes, and blocked with 1% BSA for 1 hour at room temperature. The cells were labeled with c-Fos Monoclonal Antibody (T.142.5) (product # MA5-15055) at 1:250 dilution in 0.1% BSA, incubated overnight at 4 degree Celsius and then labeled with Goat anti-Rabbit IgG (H+L) Superclonal™

Anti-rabbit Alexa546 (Invitrogen, #A10040) is an Alexa Fluor 546-conjugated polyclonal donkey anti-rabbit antibody, supplied by Invitrogen Antibodies, cited in 648 publications, with 15 published images. Applications used include IHC, IHC-IF, ICC-IF, IF, and Others. These donkey anti-rabbit IgG (H+L) whole secondary antibodies have been affinity-purified and show minimum cross-reactivity to bovine, chicken, goat, guinea pig, hamster, horse, human, mouse, rat, and sheep serum proteins. Cross-adsorption or pre- adsorption is a purification step to increase specificity of the antibody resulting in higher sensitivity and less background staining. The secondary antibody solution is passed through a column matrix containing immobilized serum proteins from potentially cross-reactive species. Only the nonspecific-binding secondary antibodies are captured in the column, and the highly specific secondaries flow through. The benefits of this extra step are apparent in multiplexing/multicolor-staining experiments (e.g., flow cytometry) where there is potential cross-reactivity with other primary antibodies or in tissue/cell fluorescent staining experiments where there may be the presence of endogenous immunoglobulins. Alexa Fluor dyes are among the most trusted fluorescent dyes available today. InvitrogenTM Alexa Fluor 546 dye is a bright, orange-fluorescent dye with excitation ideally suited to the 546 nm laser line. For stable signal generation in imaging and flow cytometry, Alexa Fluor 546 dye is pH-insensitive over a wide molar range. Probes with high fluorescence quantum yield and highphotostability allow detection of low-abundance biological structures with great sensitivity. Alexa Fluor 546 dye molecules can be attached to proteins at high molar ratios without significant self-quenching, enabling brighter conjugates and more sensitive detection. The degree of labeling for each conjugate is typically 2-8 fluorophore molecules per IgG molecule; the exact degree of labeling is indicated on the certificate of analysis for each product lot.
Using conjugate solutions: Centrifuge the protein conjugate solution briefly in a microcentrifuge before use; add only the supernatant to the experiment. This step will help eliminate any protein aggregates that may have formed during storage, thereby reducing nonspecific background staining. Because staining protocols vary with application, the appropriate dilution of antibody should be determined empirically. For the fluorophore-labeled antibodies a final concentration of 1-10 µg/mL should be satisfactory for most immunohistochemistry and flow cytometry applications.

Insulin (C27C9) Rabbit mAb #3014 (Cell Signaling) is a validated monoclonal antibody used in 119 publications. Applications include Immunohistochemistry, ChIP, and Immunoprecipitation. The antibody reacts with Insulin from human, mouse and rat. Based on the manufacturer, the antibody meats all of the quality control standards defined by Cell Signaling Technology, Inc. Validations include Immunohistochemical analysis of paraffin-embedded human pancreas, showing the staining of β cells, using Insulin (C27C9) Rabbit mAb. The antibody is further reported to show very clear staining at 1:2000 with no background staining in primary human cells

AlexaFluor750-conjugated goat anti-rabbit (Invitrogen, A21039). An Alexa Fluor 750-conjugated polyclonal goat anti-rabbit antibody, supplied by Invitrogen Antibodies, cited in 72 publications, with 1 published image. Applications used include WB, IHC, IHC-IF, FC/FACS, and 3 Others.

anti-glucagon (Takara, M182) is a polyclonal guinea pig antibody, supplied by Takara Bio, cited in 24 publications. Applications used include IHC and CLARITY. Its a Guinea Pig polyclonal antibody raised against the peptide [HSQGTFTSDYSKYLDSRRAQDFVQWLMNT] of human Glucagon conjugated with KLH as an immunogen.The lyophilized antibody was dissolved in 50 µl of specifed water. The antibody dilutions were applied for ELISA assay by colorimetric detection using a microtiter plate immobilized with human Glucagon peptide. The expected antibody titration was obtained. Manufacturing Control:
Purification: Guinea Pig serum IgG was purified by affinity column chromatography, dissolved in 10 mM PBS, pH 7.4, containing 1.0% bovine serum albumin, and then lyophilized. The lyophilized antibody does not contain preservative.

goat anti-guinea pig AF555 (Invitrogen, A21435) is an Alexa Fluor 555-conjugated polyclonal goat anti-guinea pig antibody, supplied by Invitrogen Antibodies, cited in 215 publications, with 1 published image. Applications used include IHC, IHC-IF, ICC, ICC-IF, and 9 Others.

Animals and other organisms

Policy information about studies involving animals; ARRIVE guidelines recommended for reporting animal research

Laboratory animals

Figure 1A-J: 35-wk old male C57BL/6J Vgat Cre+/- Gipr wt/wt (WT) and Vgat Cre+/- Gipr flx/flx (KO) mice
Figure 1K and L: 37-wk old male C57BL/6J Vgat Cre+/- Gipr wt/wt (WT) and Vgat Cre+/- Gipr flx/flx (KO) mice
Figure 1M and N: 38-wk old male C57BL/6J Vgat Cre+/- Gipr wt/wt (WT) and Vgat Cre+/- Gipr flx/flx (KO) mice
Figure 1O and P: 34-wk old male C57BL/6J wVgat Cre+/- Gipr wt/wt (WT) and Vgat Cre+/- Gipr flx/flx (KO) mice
Figure 1Q-T: 40-wk old male C57BL/6J Vgat Cre+/- Gipr wt/wt (WT) and Vgat Cre+/- Gipr flx/flx (KO) mice
Figure 2A-J: 36-wk old male C57BL/6J Vgat Cre+/- Gipr wt/wt (WT) and Vgat Cre+/- Gipr flx/flx (KO) mice
Figure 2K-M: 38-wk old male C57BL/6J Vgat Cre+/- Gipr wt/wt (WT) and Vgat Cre+/- Gipr flx/flx (KO) mice
Figure 2N: 39-wk old male C57BL/6J Vgat Cre+/- Gipr wt/wt (WT) and Vgat Cre+/- Gipr flx/flx (KO) mice
Figure 2O and P: 40-wk old male C57BL/6J Vgat Cre+/- Gipr wt/wt (WT) and Vgat Cre+/- Gipr flx/flx (KO) mice
Figure 3A-C: 52-wk old male C57BL/6J wildtype mice
Figure 3D and E: 26-wk old male C57BL/6J Vgat Cre+/- Gipr wt/wt (WT) and Vgat Cre+/- Gipr flx/flx (KO) mice
Figure 3F and G: 35-wk old male C57BL/6J Vgat Cre+/- Gipr wt/wt (WT) and Vgat Cre+/- Gipr flx/flx (KO) mice
Figure 3H and I: 40-wk old female C57BL/6J Vgat Cre+/- Gipr wt/wt (WT) and Vgat Cre+/- Gipr flx/flx (KO) mice
Figure 4A-I: 38-wk old male C57BL/6J Vgat Cre+/- Gipr wt/wt (WT) mice
Figure 4J: 35-wk old male C57BL/6J Vgat Cre+/- Gipr wt/wt (WT) mice
Figure 4K-T: 35-wk old male C57BL/6J Vgat Cre+/- Gipr flx/flx (KO) mice
Extended Data Figure 1 A-C, E: 40-wk old male C57BL/6J Vgat Cre+/- Gipr wt/wt (WT) and Vgat Cre+/- Gipr flx/flx (KO) mice
Extended Data Figure 1 D, x-wk old male C57BL/6J Vgat Cre+/- Gipr wt/wt (WT) and Vgat Cre+/- Gipr flx/flx (KO) mice
Extended Data Figure 1 F-S: 38-wk old male C57BL/6J Vgat Cre+/- Gipr wt/wt (WT) and Vgat Cre+/- Gipr flx/flx (KO) mice
Extended Data Figure 2 A-T: 40-wk old male C57BL/6J Vgat Cre+/- Gipr wt/wt (WT) and Vgat Cre+/- Gipr flx/flx (KO) mice
Extended Data Figure 3A-H: 40-wk old female Vgat Cre+/- Gipr wt/wt (WT) and Vgat Cre+/- Gipr flx/flx (KO) mice
Extended Data Figure 3I,J: 43-wk old female Vgat Cre+/- Gipr wt/wt (WT) and Vgat Cre+/- Gipr flx/flx (KO) mice
Extended Data Figure 3K: 42-wk old female Vgat Cre+/- Gipr wt/wt (WT) and Vgat Cre+/- Gipr flx/flx (KO) mice
Extended Data Figure 3L-P: 44-wk old female Vgat Cre+/- Gipr wt/wt (WT) and Vgat Cre+/- Gipr flx/flx (KO) mice
Extended Data Figure 5: A-F, 26-wk old male C57BL/6J Vgat Cre+/- Gipr wt/wt (WT) and Vgat Cre+/- Gipr flx/flx (KO) mice
Extended Data Figure 6: A,B, E: 38-wk old male C57BL/6J Vgat Cre+/- Gipr wt/wt (WT) and Vgat Cre+/- Gipr flx/flx (KO) mice

Wild animals	no wild animals were used in the study
Field-collected samples	no field collected animals were used in the study
Ethics oversight	Experiments were performed in accordance with the Animal Protection Law of the European Union after permission by the Government of Upper Bavaria, Germany

Note that full information on the approval of the study protocol must also be provided in the manuscript.
