# Supplementary figures and images for: Glucose-dependent insulinotropic polypeptide regulates body weight and food intake via GABAergic neurons in mice (part 2 of 2)
Source: Nat Metab. 2023 Nov 9;5(12):2075–85. doi: 10.1038/s42255-023-00931-7 (PMC10730394; doi:10.1038/s42255-023-00931-7)

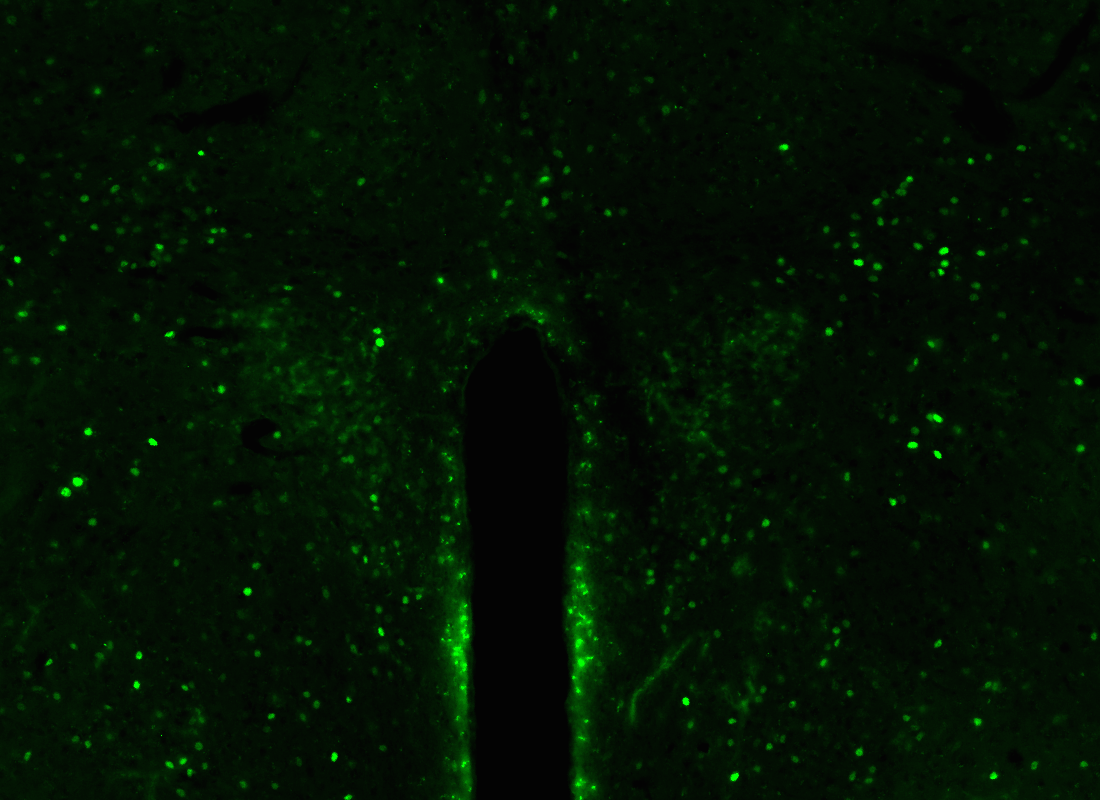

Supplement: Supplementary file 2 — Original pictures of cFos and Cy5 drug appearance shown in Extended Data Fig. 5a–h, including replicates used for quantification. [file 42255_2023_931_MOESM2_ESM.zip › Raw Data Extended Data Figure 5/04-PVN/1-M-KO-GIP-PVN.tif]

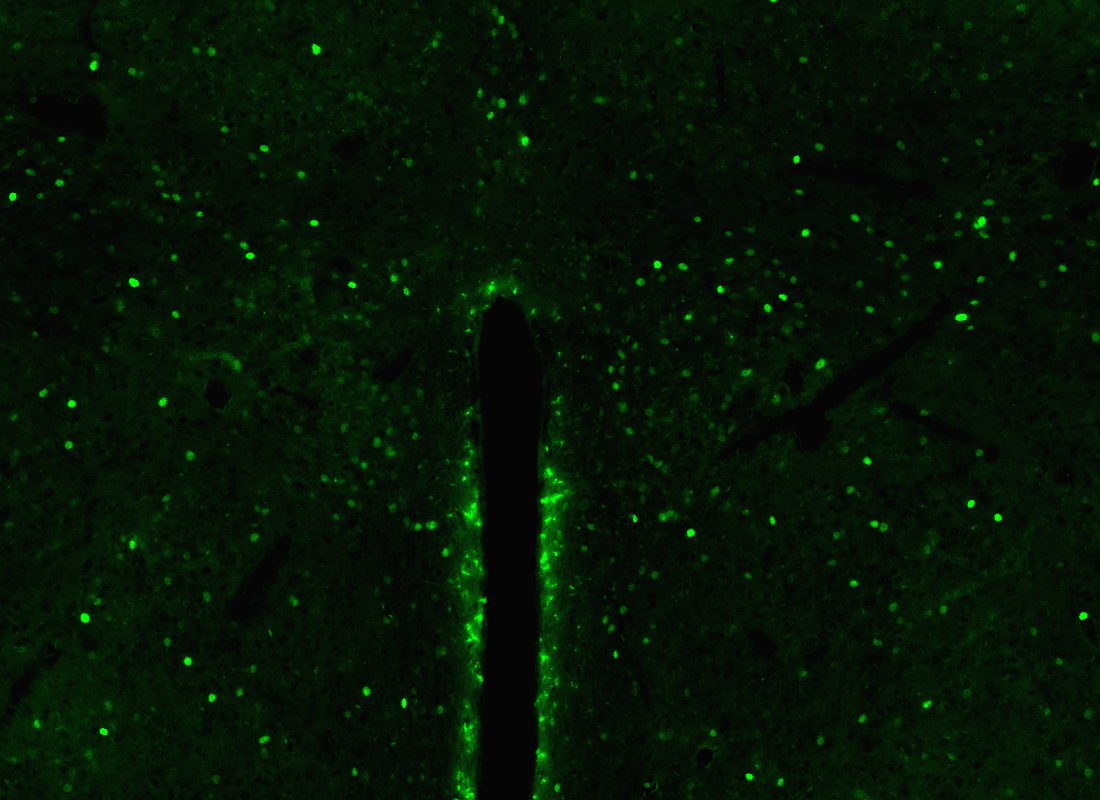

Supplement: Supplementary file 2 — Original pictures of cFos and Cy5 drug appearance shown in Extended Data Fig. 5a–h, including replicates used for quantification. [file 42255_2023_931_MOESM2_ESM.zip › Raw Data Extended Data Figure 5/04-PVN/54-M-WT-GIP-PVN.tif]

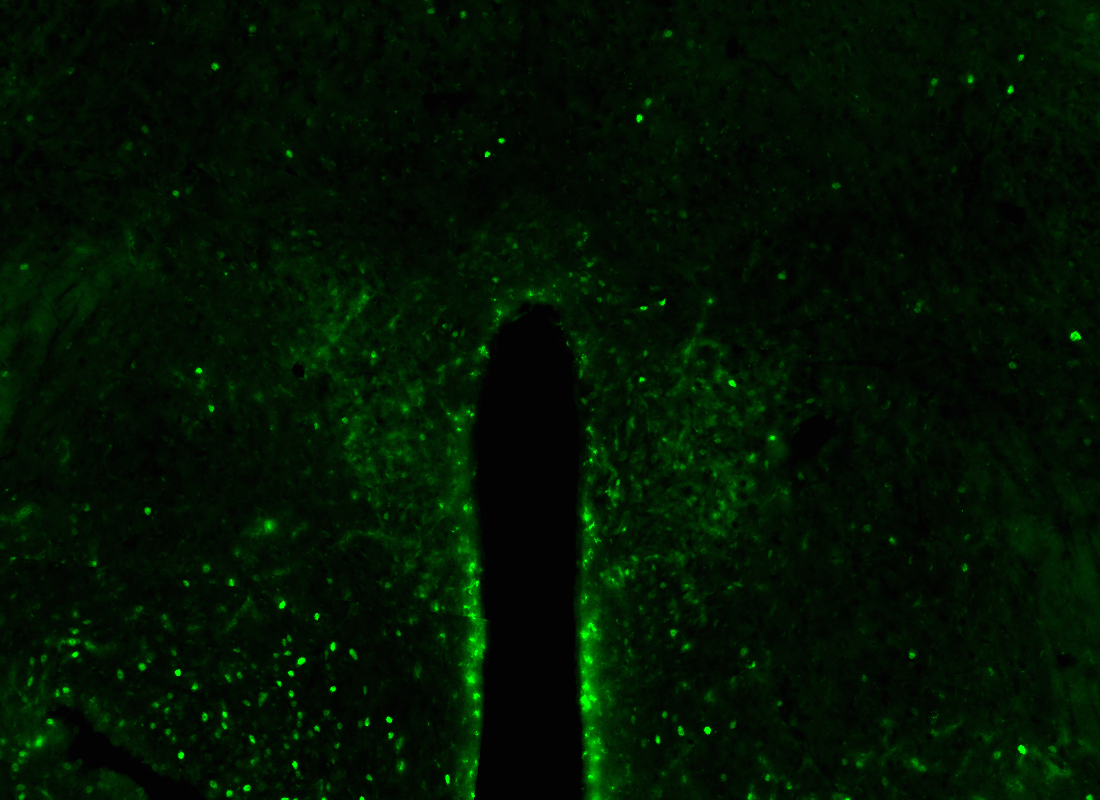

Supplement: Supplementary file 2 — Original pictures of cFos and Cy5 drug appearance shown in Extended Data Fig. 5a–h, including replicates used for quantification. [file 42255_2023_931_MOESM2_ESM.zip › Raw Data Extended Data Figure 5/04-PVN/14-M-KO-GIP-PVN.tif]

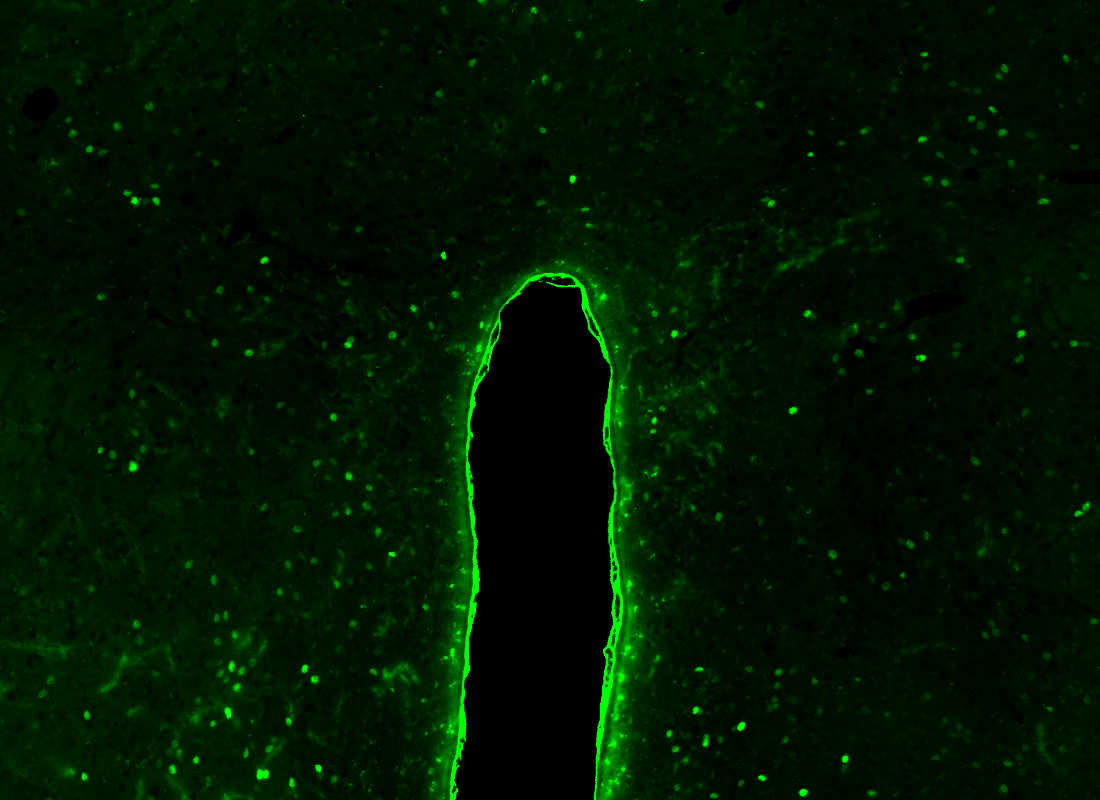

Supplement: Supplementary file 2 — Original pictures of cFos and Cy5 drug appearance shown in Extended Data Fig. 5a–h, including replicates used for quantification. [file 42255_2023_931_MOESM2_ESM.zip › Raw Data Extended Data Figure 5/04-PVN/64-M-WT-Veh-PVN.tif]

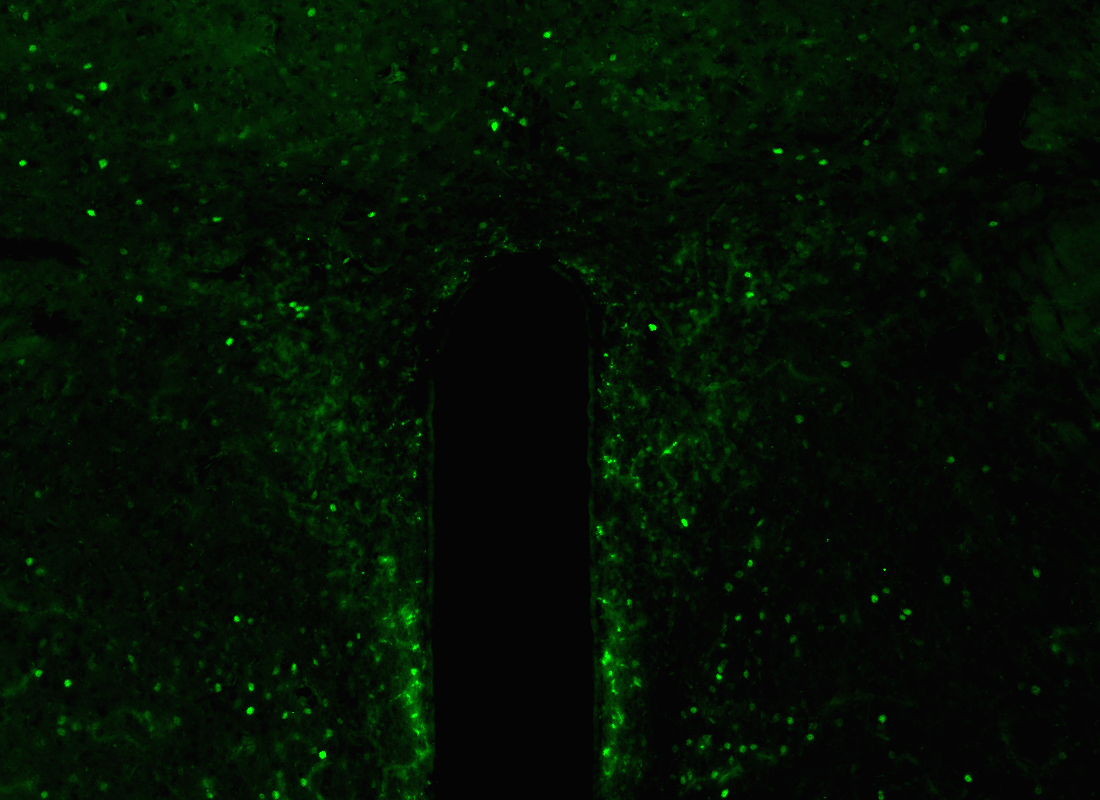

Supplement: Supplementary file 2 — Original pictures of cFos and Cy5 drug appearance shown in Extended Data Fig. 5a–h, including replicates used for quantification. [file 42255_2023_931_MOESM2_ESM.zip › Raw Data Extended Data Figure 5/04-PVN/55-M-WT-GIP-PVN.tif]

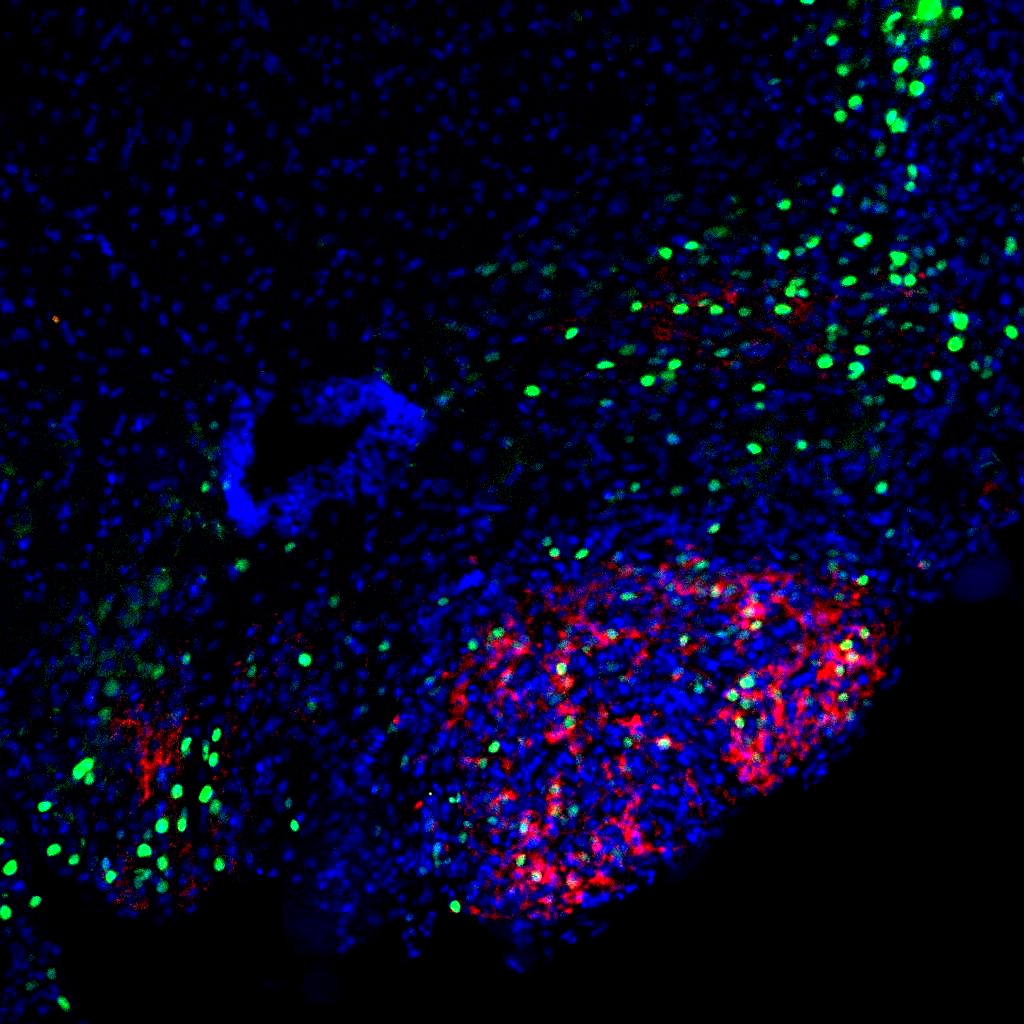

Supplement: Supplementary file 3 — Original pictures of cFos and Cy5 drug appearance shown in Fig. 3, including replicates used for quantification. [file 42255_2023_931_MOESM3_ESM.zip › Raw Data Figure 3/Figure 3A-C/Adjusted/07-GLP1_Cy5_3a.jpg]

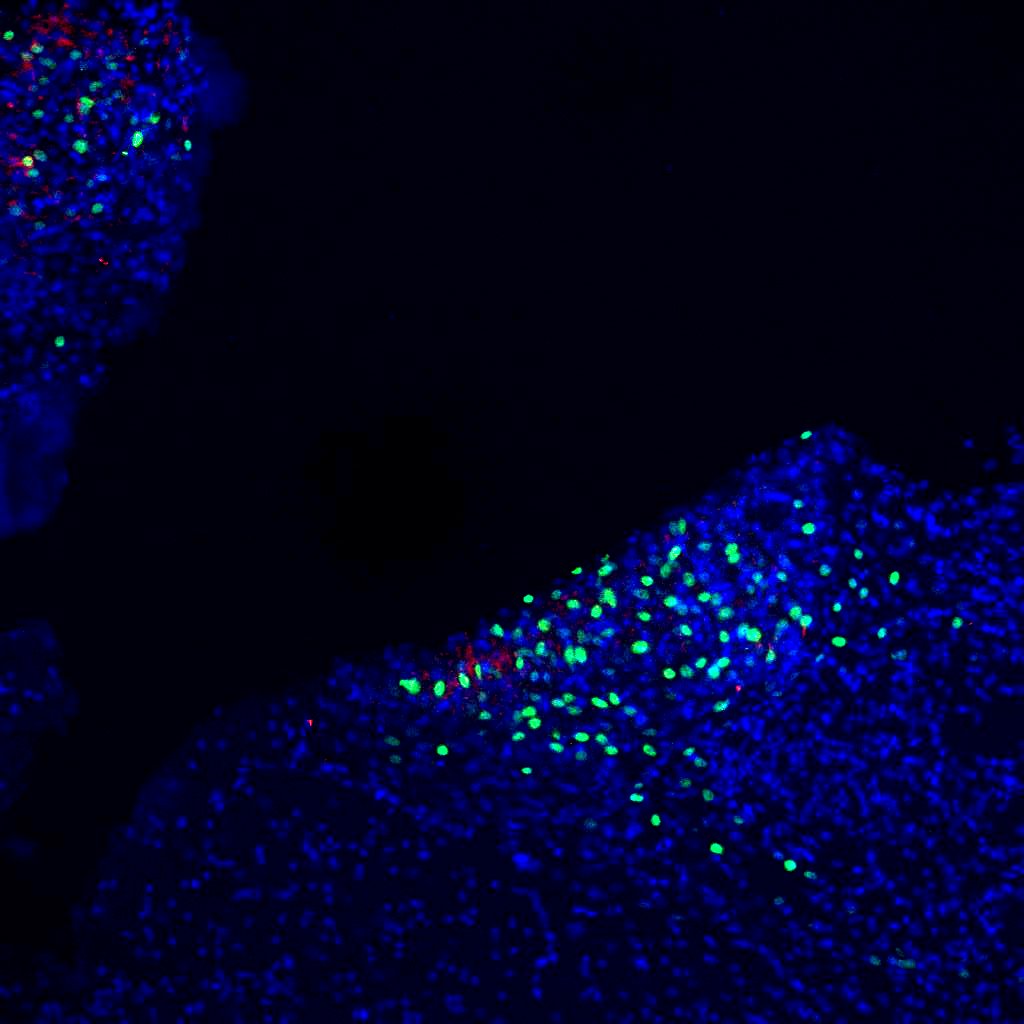

Supplement: Supplementary file 3 — Original pictures of cFos and Cy5 drug appearance shown in Fig. 3, including replicates used for quantification. [file 42255_2023_931_MOESM3_ESM.zip › Raw Data Figure 3/Figure 3A-C/Adjusted/05-GLP1_Cy5_1a_part2.jpg]

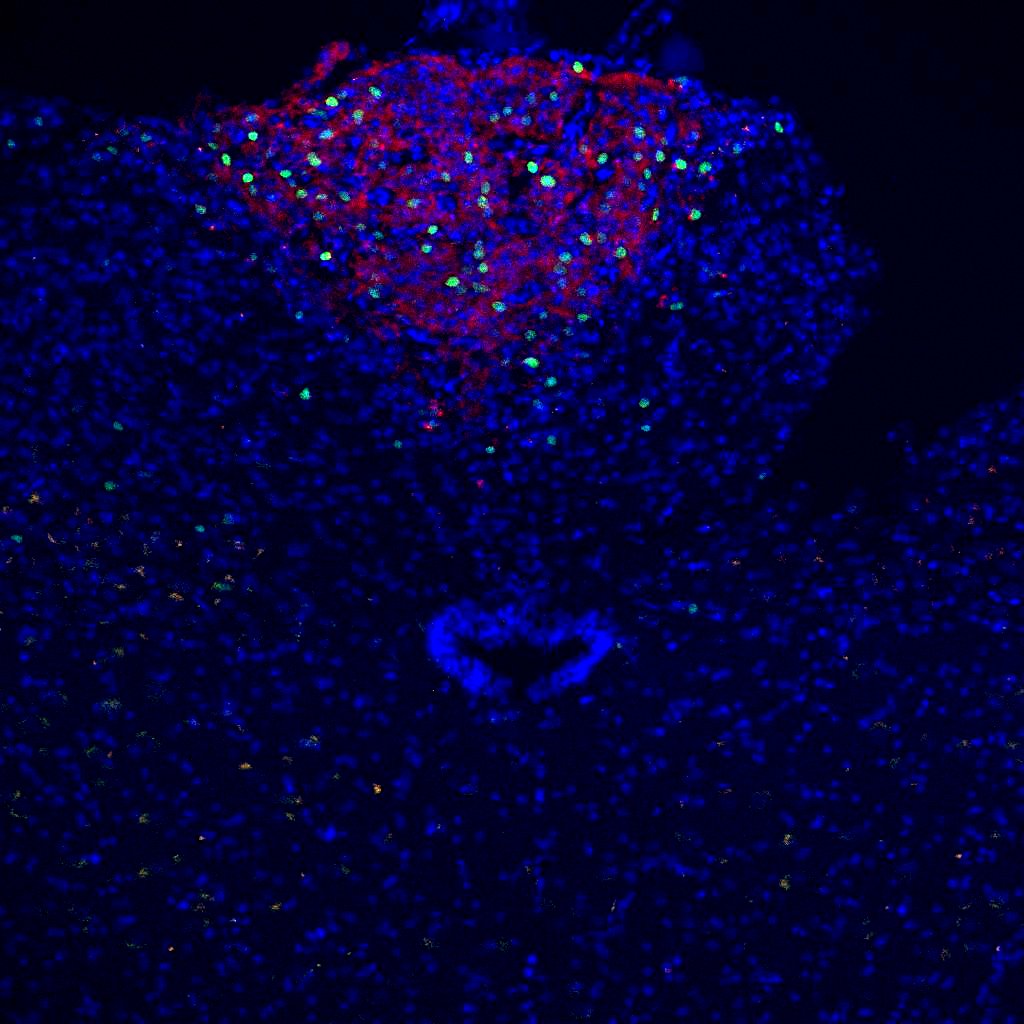

Supplement: Supplementary file 3 — Original pictures of cFos and Cy5 drug appearance shown in Fig. 3, including replicates used for quantification. [file 42255_2023_931_MOESM3_ESM.zip › Raw Data Figure 3/Figure 3A-C/Adjusted/10-GIP_Cy5_2a.jpg]

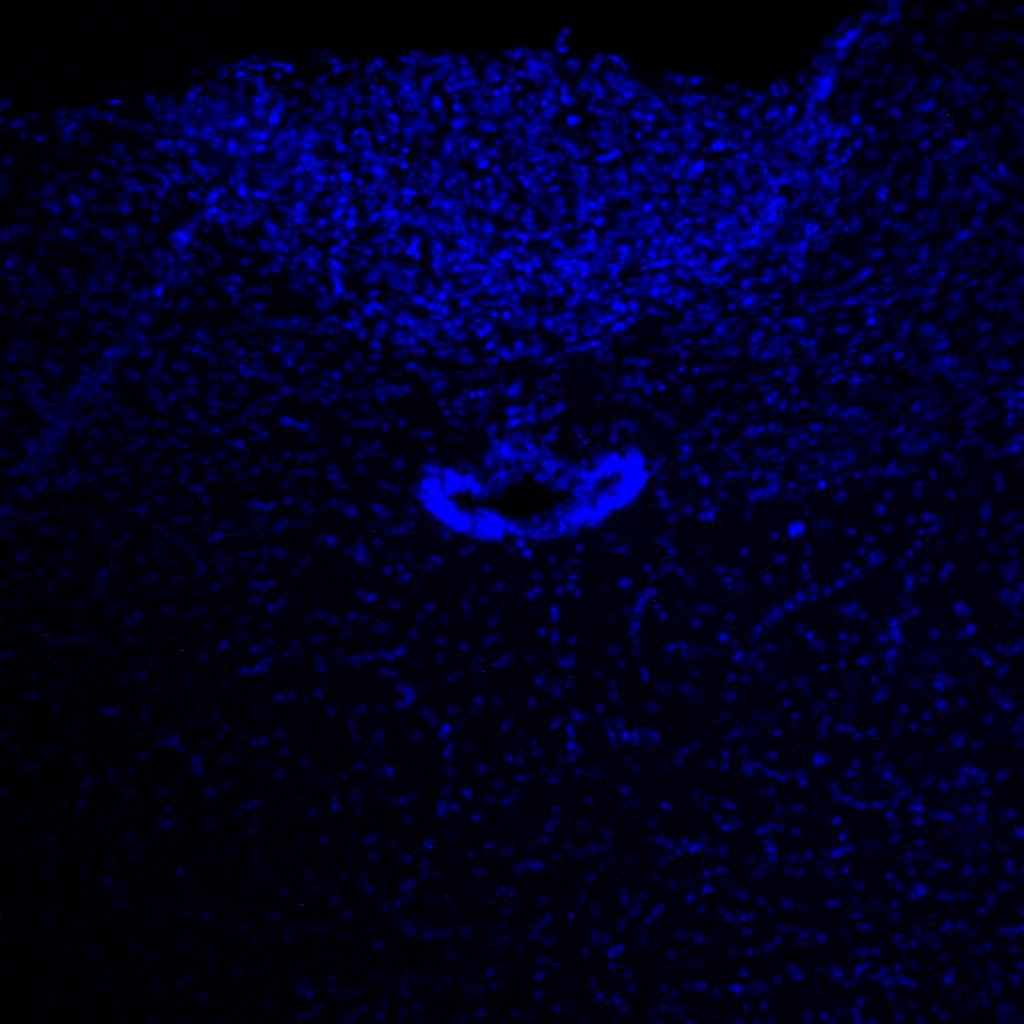

Supplement: Supplementary file 3 — Original pictures of cFos and Cy5 drug appearance shown in Fig. 3, including replicates used for quantification. [file 42255_2023_931_MOESM3_ESM.zip › Raw Data Figure 3/Figure 3A-C/Adjusted/01-Veh_1a.jpg]

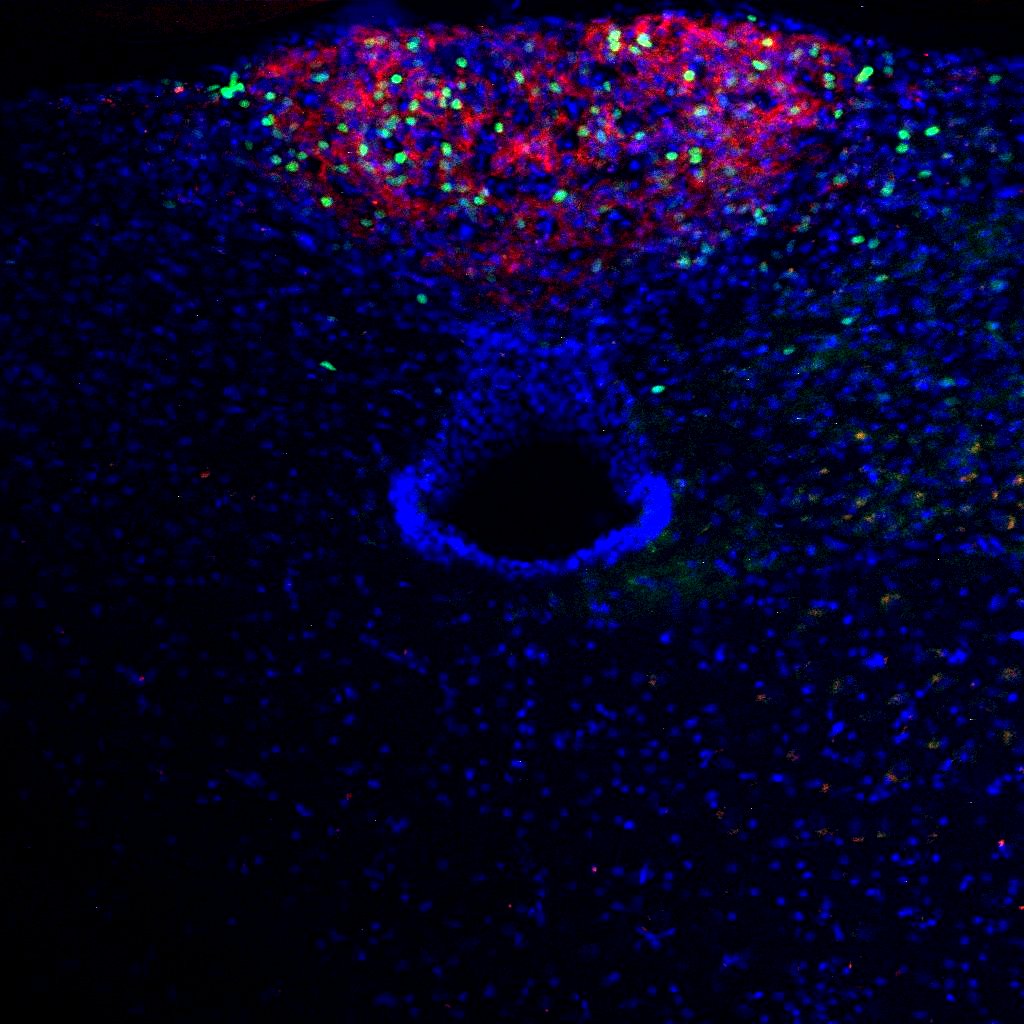

Supplement: Supplementary file 3 — Original pictures of cFos and Cy5 drug appearance shown in Fig. 3, including replicates used for quantification. [file 42255_2023_931_MOESM3_ESM.zip › Raw Data Figure 3/Figure 3A-C/Adjusted/11-GIP_Cy5_3a.jpg]

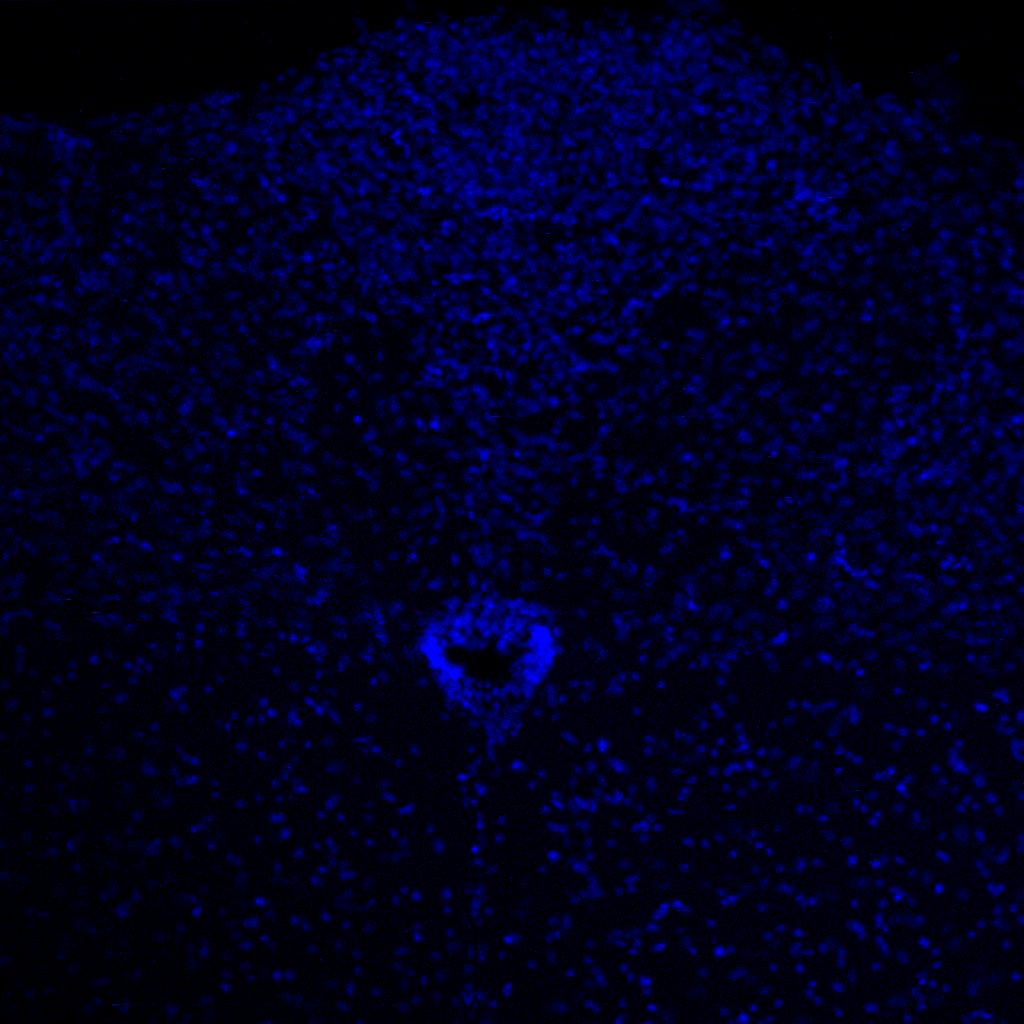

Supplement: Supplementary file 3 — Original pictures of cFos and Cy5 drug appearance shown in Fig. 3, including replicates used for quantification. [file 42255_2023_931_MOESM3_ESM.zip › Raw Data Figure 3/Figure 3A-C/Adjusted/02-Veh_2a.jpg]

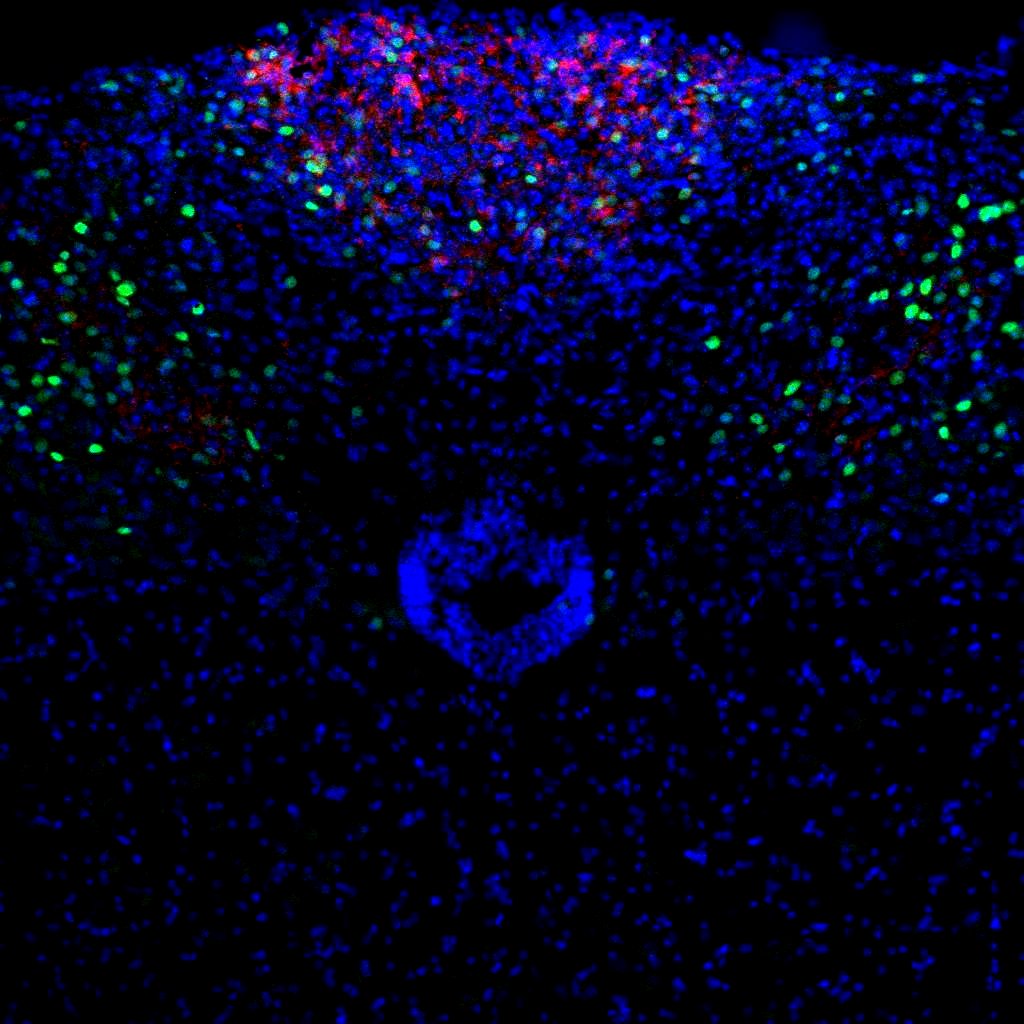

Supplement: Supplementary file 3 — Original pictures of cFos and Cy5 drug appearance shown in Fig. 3, including replicates used for quantification. [file 42255_2023_931_MOESM3_ESM.zip › Raw Data Figure 3/Figure 3A-C/Adjusted/08-GLP1_Cy5_4a.jpg]

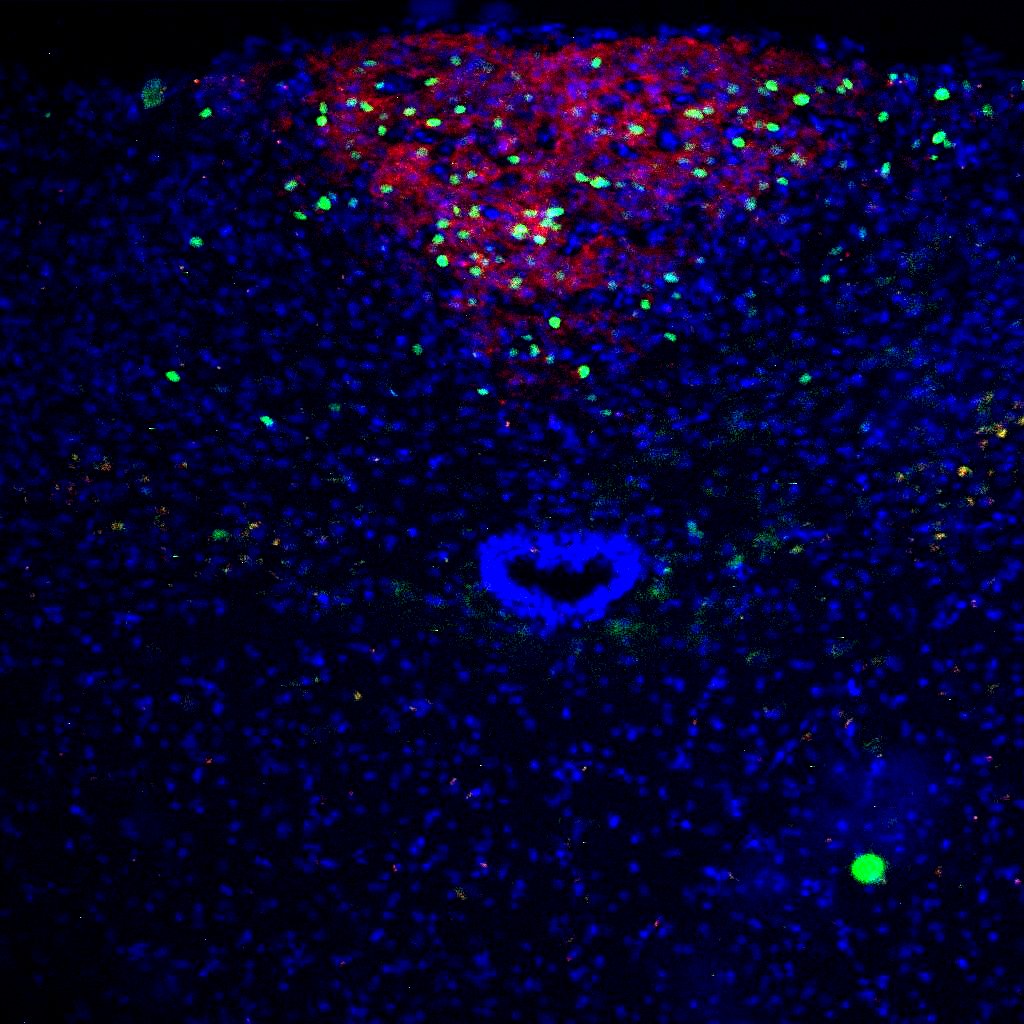

Supplement: Supplementary file 3 — Original pictures of cFos and Cy5 drug appearance shown in Fig. 3, including replicates used for quantification. [file 42255_2023_931_MOESM3_ESM.zip › Raw Data Figure 3/Figure 3A-C/Adjusted/12-GIP_Cy5_4a.jpg]

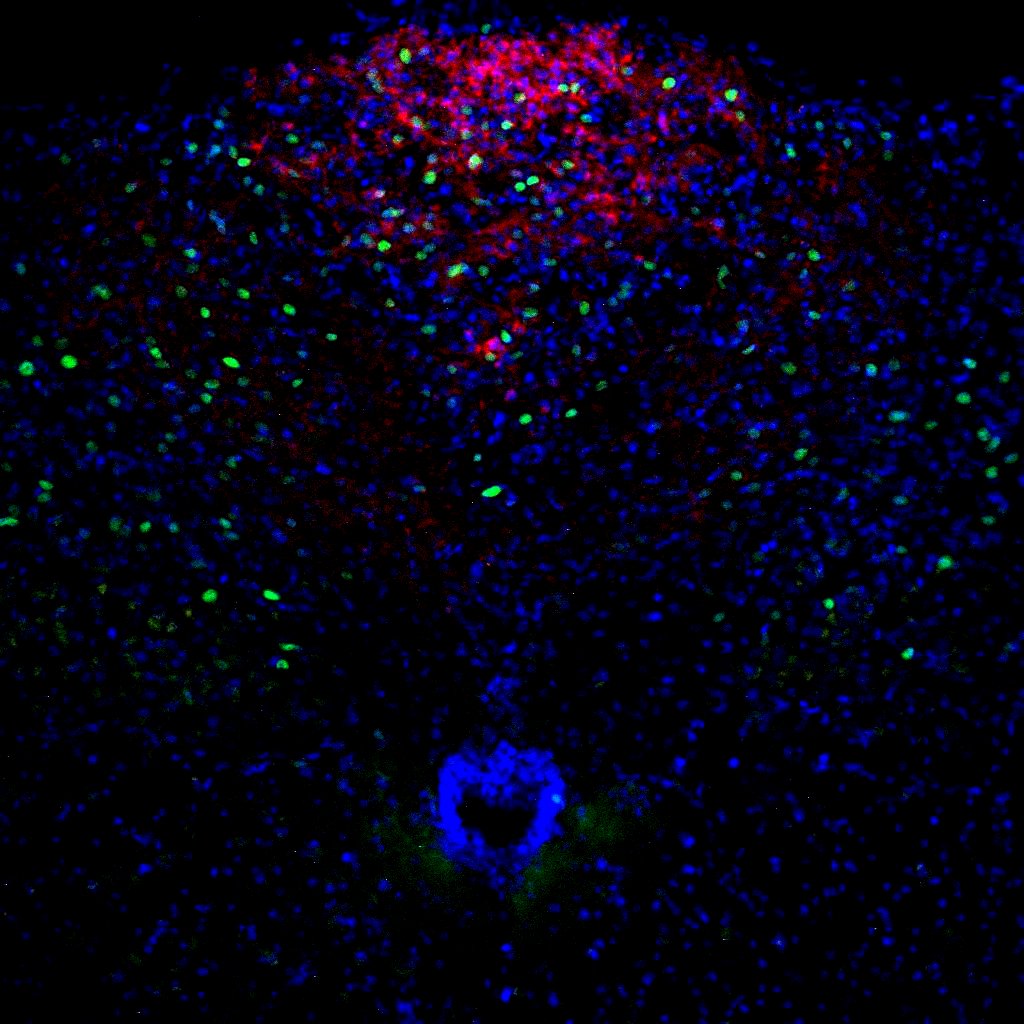

Supplement: Supplementary file 3 — Original pictures of cFos and Cy5 drug appearance shown in Fig. 3, including replicates used for quantification. [file 42255_2023_931_MOESM3_ESM.zip › Raw Data Figure 3/Figure 3A-C/Adjusted/06-GLP1_Cy5_2a.jpg]

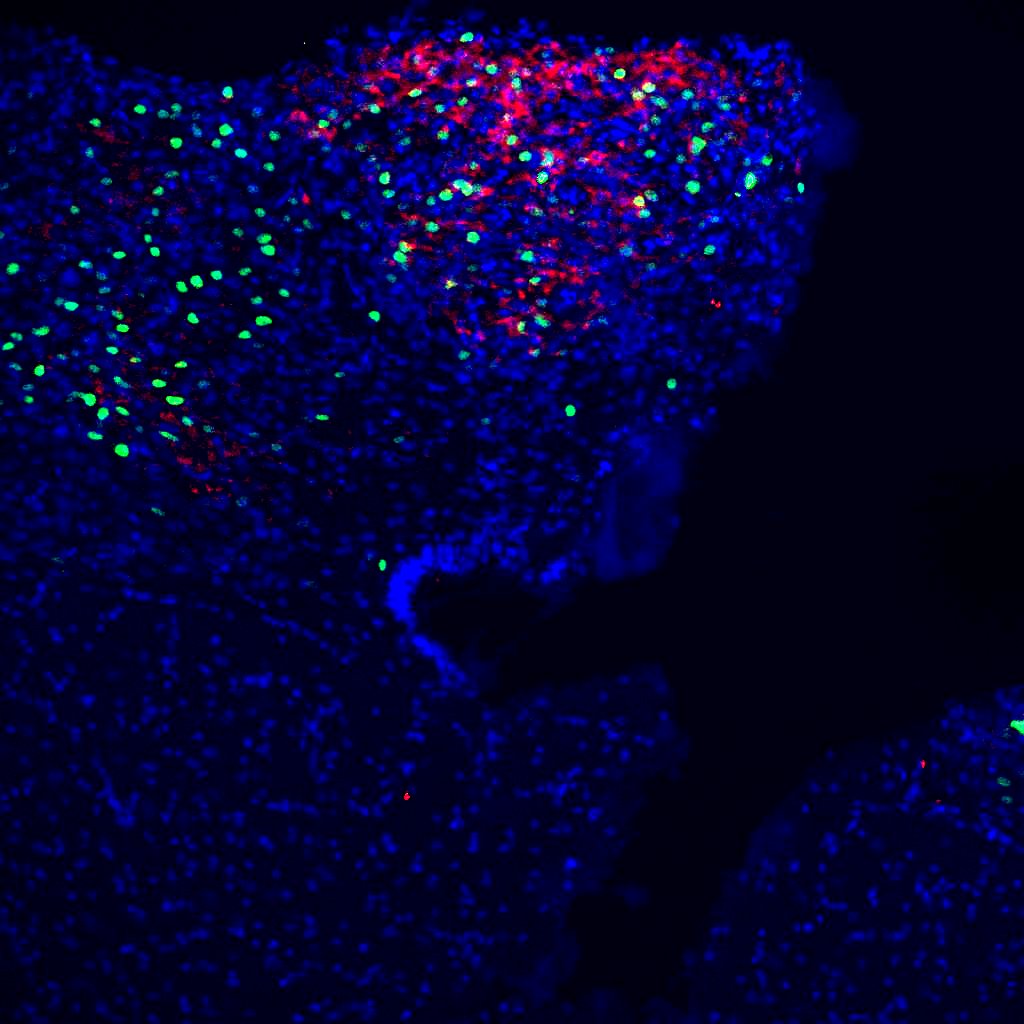

Supplement: Supplementary file 3 — Original pictures of cFos and Cy5 drug appearance shown in Fig. 3, including replicates used for quantification. [file 42255_2023_931_MOESM3_ESM.zip › Raw Data Figure 3/Figure 3A-C/Adjusted/04-GLP1_Cy5_1a_part1.jpg]

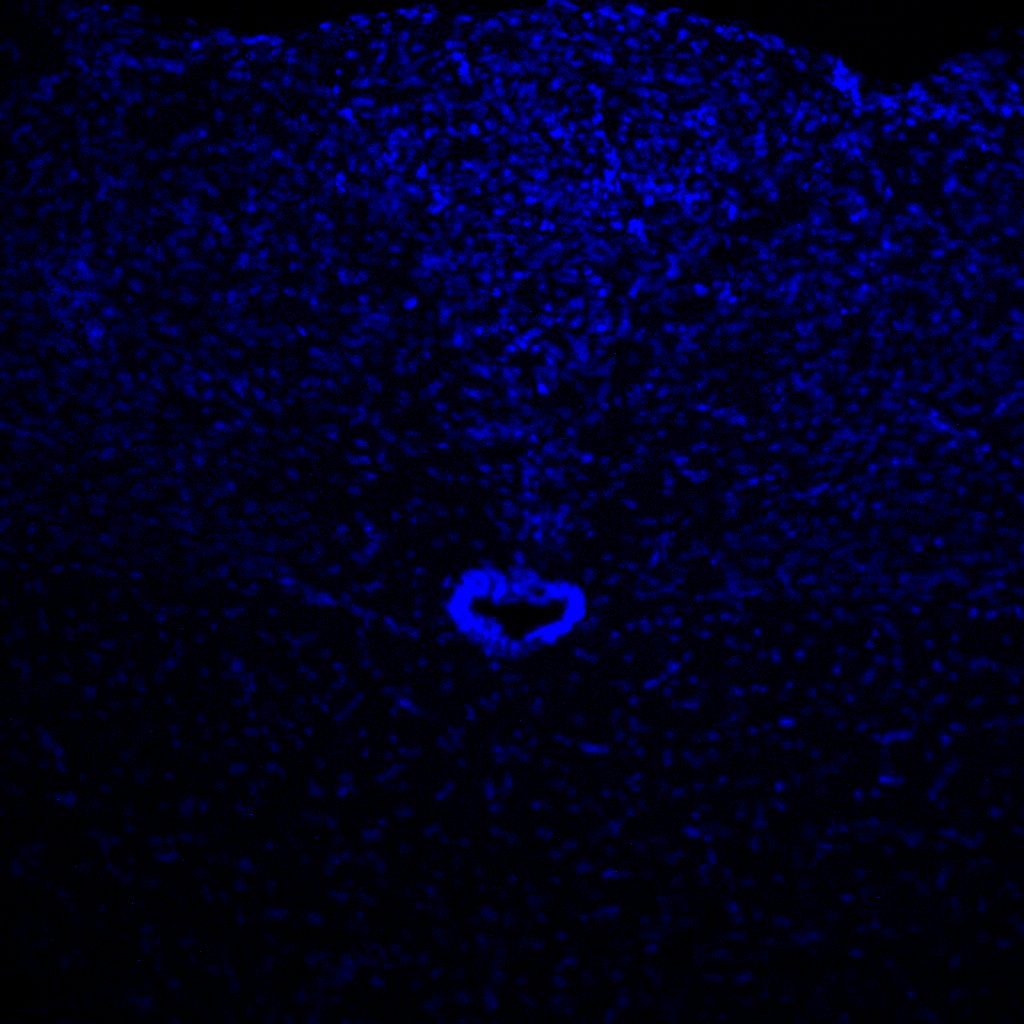

Supplement: Supplementary file 3 — Original pictures of cFos and Cy5 drug appearance shown in Fig. 3, including replicates used for quantification. [file 42255_2023_931_MOESM3_ESM.zip › Raw Data Figure 3/Figure 3A-C/Adjusted/03-Veh_3a.jpg]

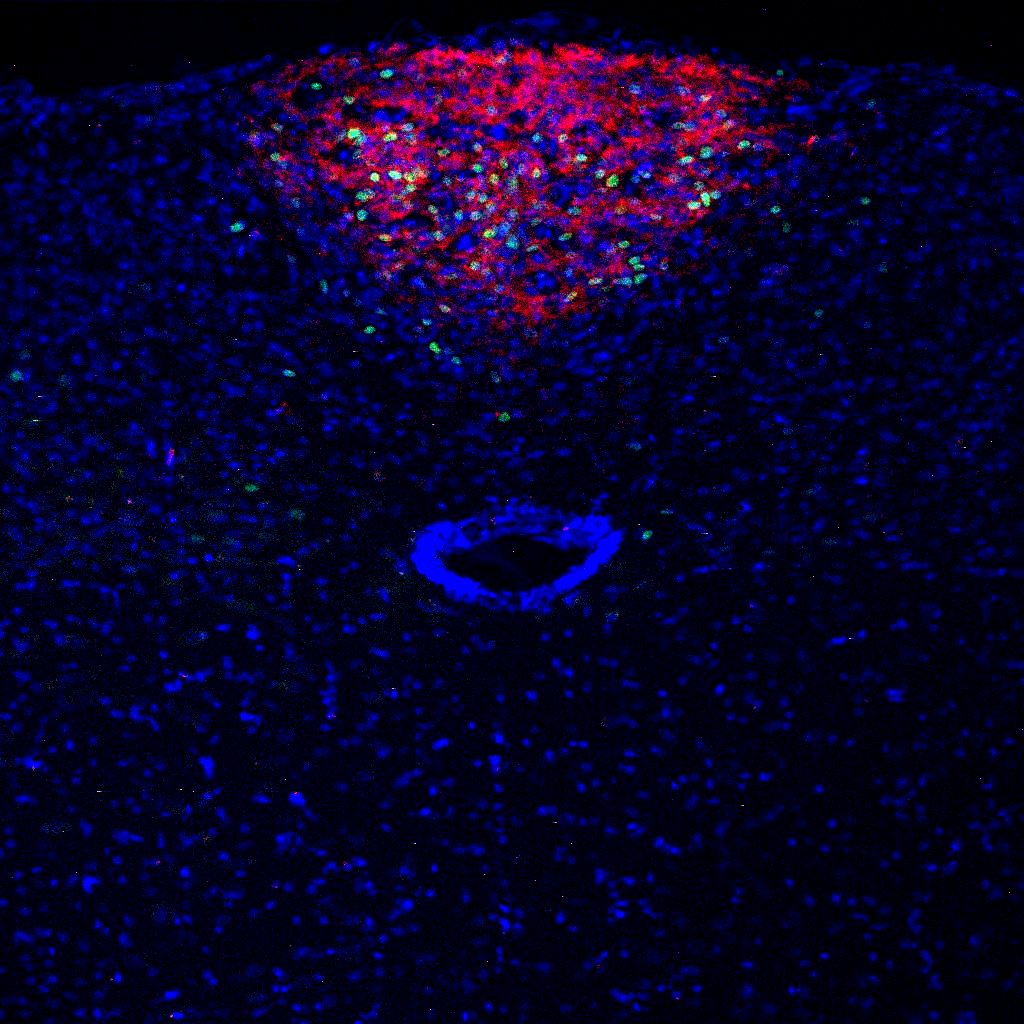

Supplement: Supplementary file 3 — Original pictures of cFos and Cy5 drug appearance shown in Fig. 3, including replicates used for quantification. [file 42255_2023_931_MOESM3_ESM.zip › Raw Data Figure 3/Figure 3A-C/Adjusted/09-GIP_Cy5_1a.jpg]

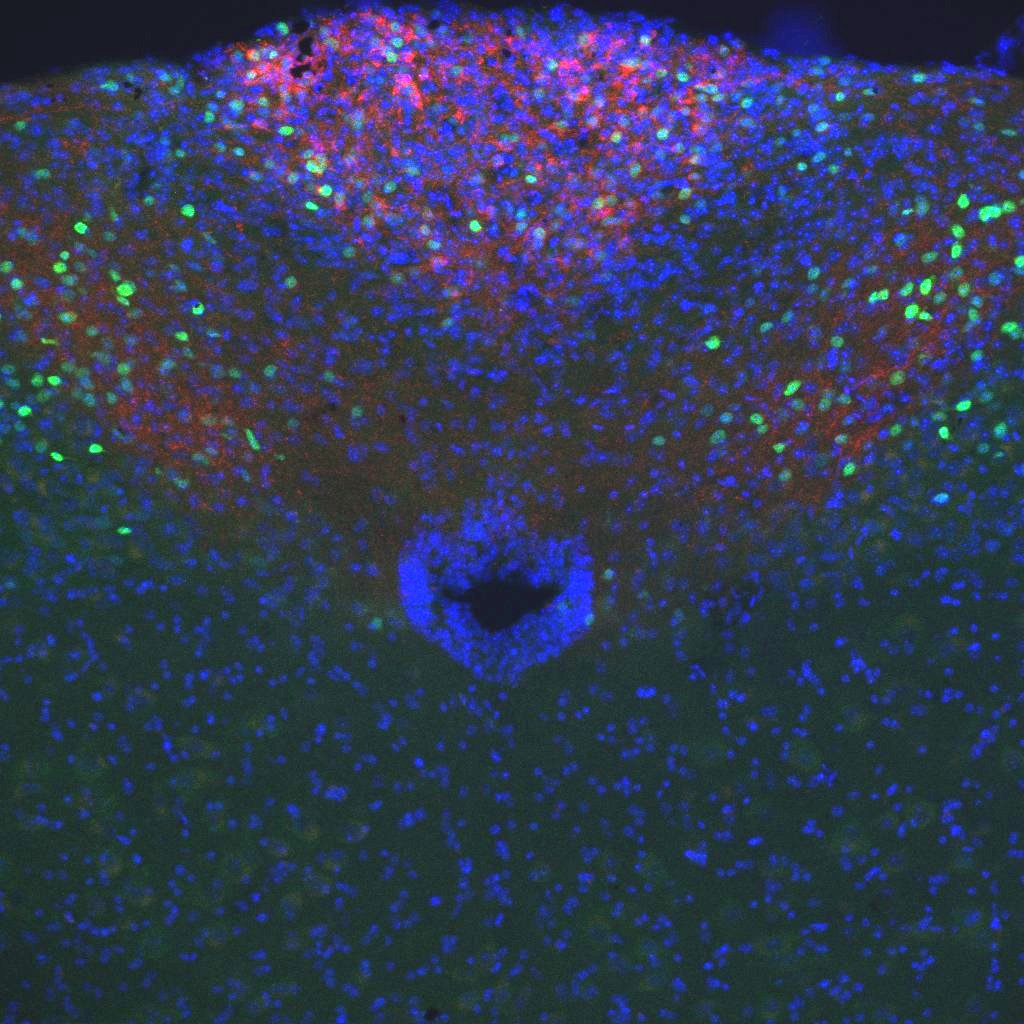

Supplement: Supplementary file 3 — Original pictures of cFos and Cy5 drug appearance shown in Fig. 3, including replicates used for quantification. [file 42255_2023_931_MOESM3_ESM.zip › Raw Data Figure 3/Figure 3A-C/Unadjusted/Fig3A_GLP1_Cy5_4.jpg]

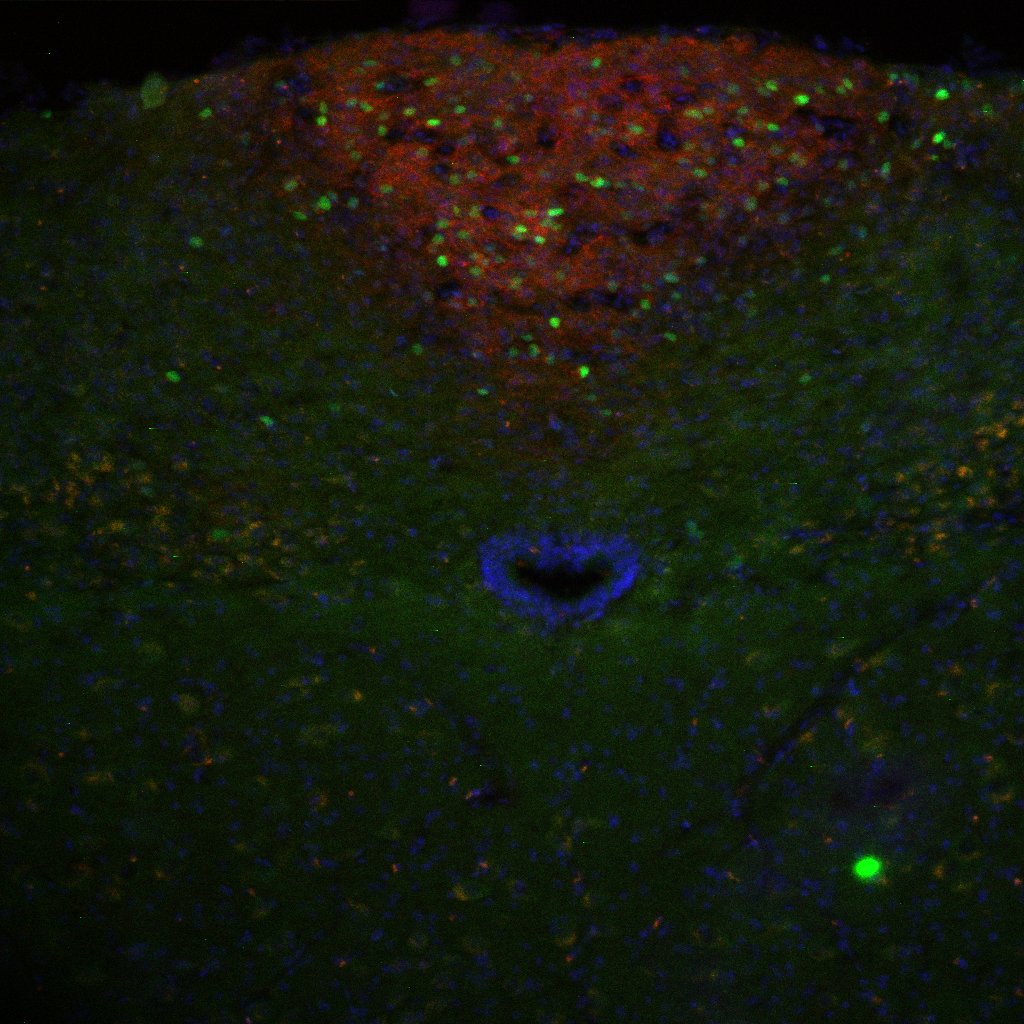

Supplement: Supplementary file 3 — Original pictures of cFos and Cy5 drug appearance shown in Fig. 3, including replicates used for quantification. [file 42255_2023_931_MOESM3_ESM.zip › Raw Data Figure 3/Figure 3A-C/Unadjusted/Fig3A_GIP_Cy5_4.jpg]

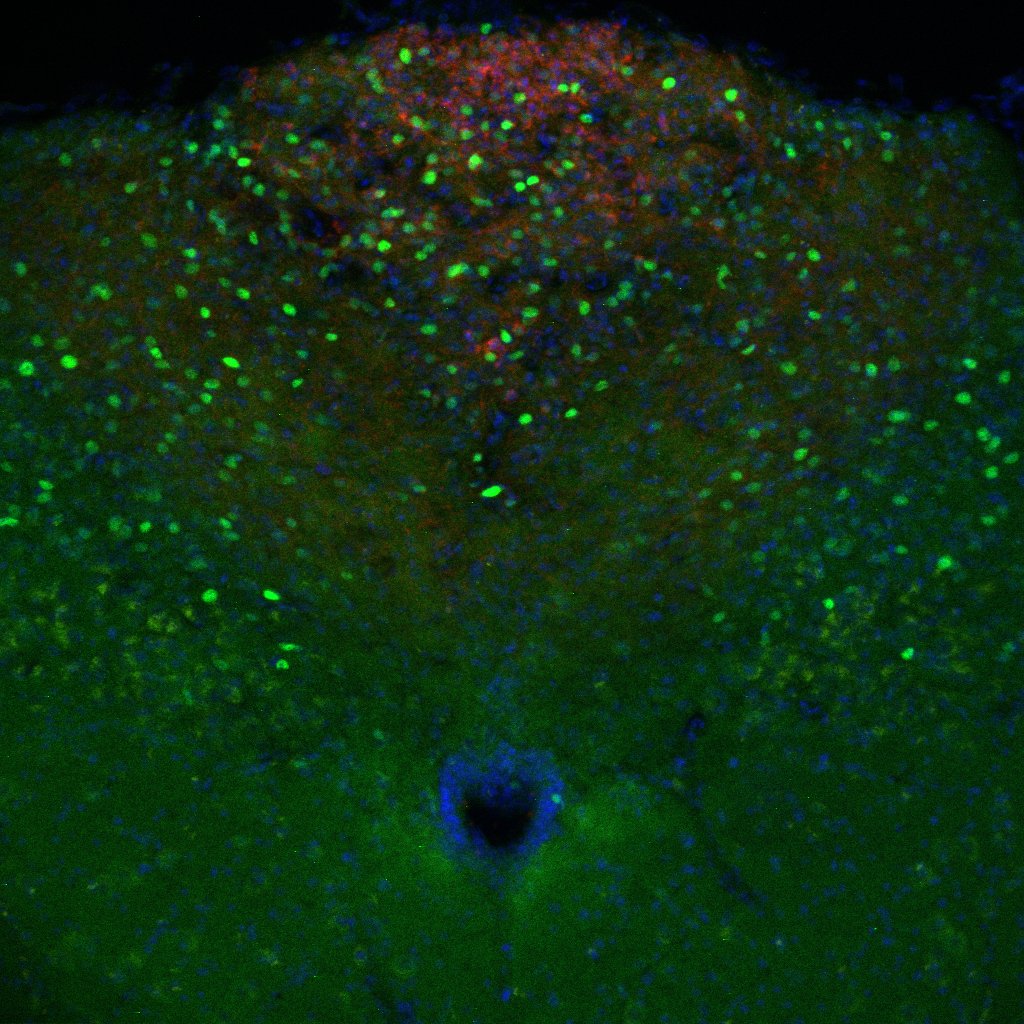

Supplement: Supplementary file 3 — Original pictures of cFos and Cy5 drug appearance shown in Fig. 3, including replicates used for quantification. [file 42255_2023_931_MOESM3_ESM.zip › Raw Data Figure 3/Figure 3A-C/Unadjusted/Fig3A_GLP1_Cy5_2.jpg]

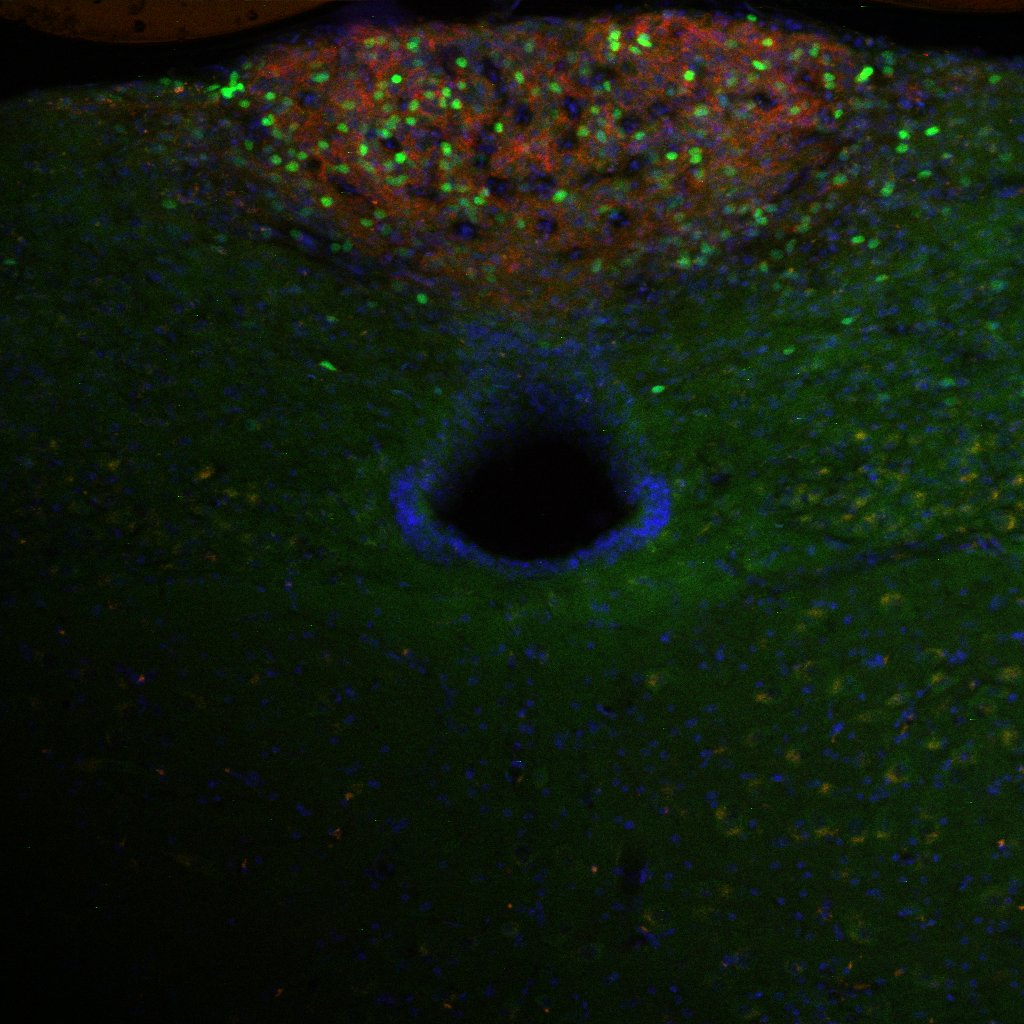

Supplement: Supplementary file 3 — Original pictures of cFos and Cy5 drug appearance shown in Fig. 3, including replicates used for quantification. [file 42255_2023_931_MOESM3_ESM.zip › Raw Data Figure 3/Figure 3A-C/Unadjusted/Fig3A_GIP_Cy5_3.jpg]

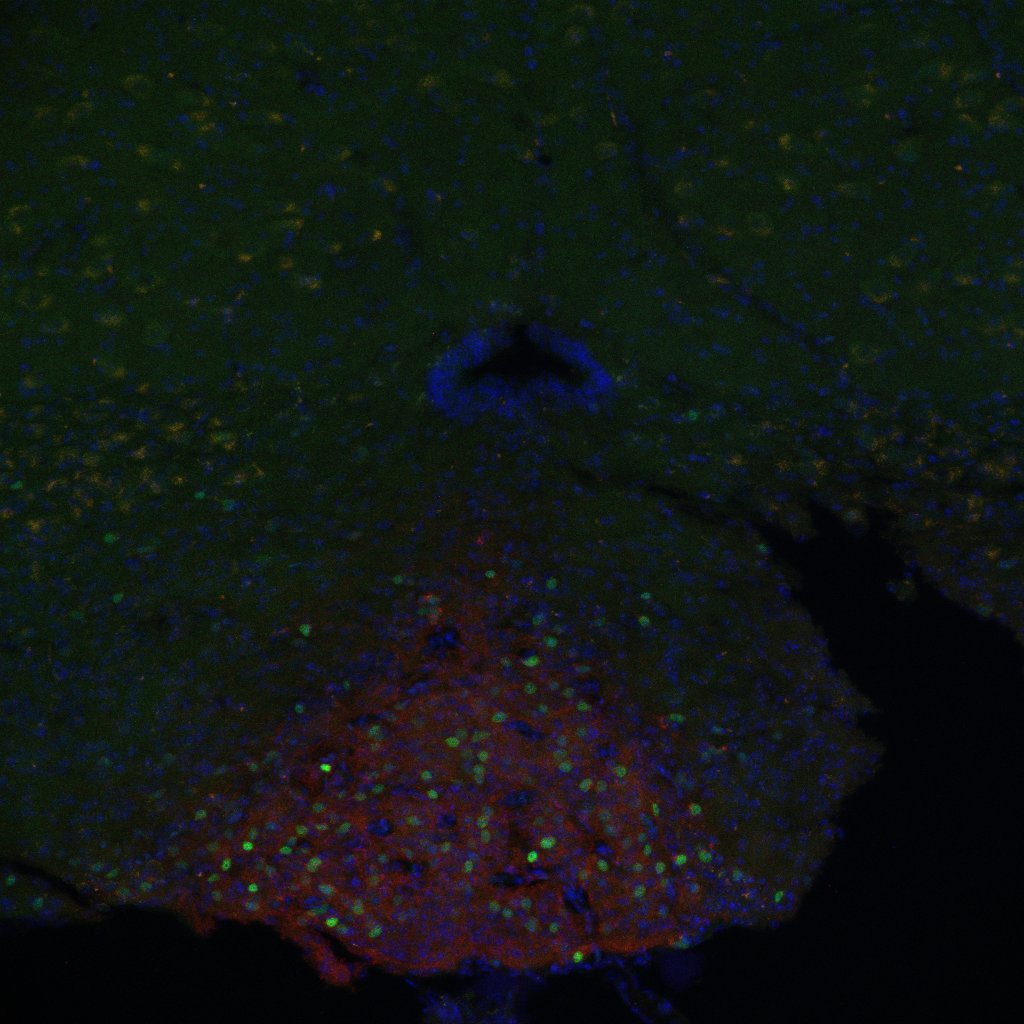

Supplement: Supplementary file 3 — Original pictures of cFos and Cy5 drug appearance shown in Fig. 3, including replicates used for quantification. [file 42255_2023_931_MOESM3_ESM.zip › Raw Data Figure 3/Figure 3A-C/Unadjusted/Fig3A_GIP_Cy5_2.jpg]

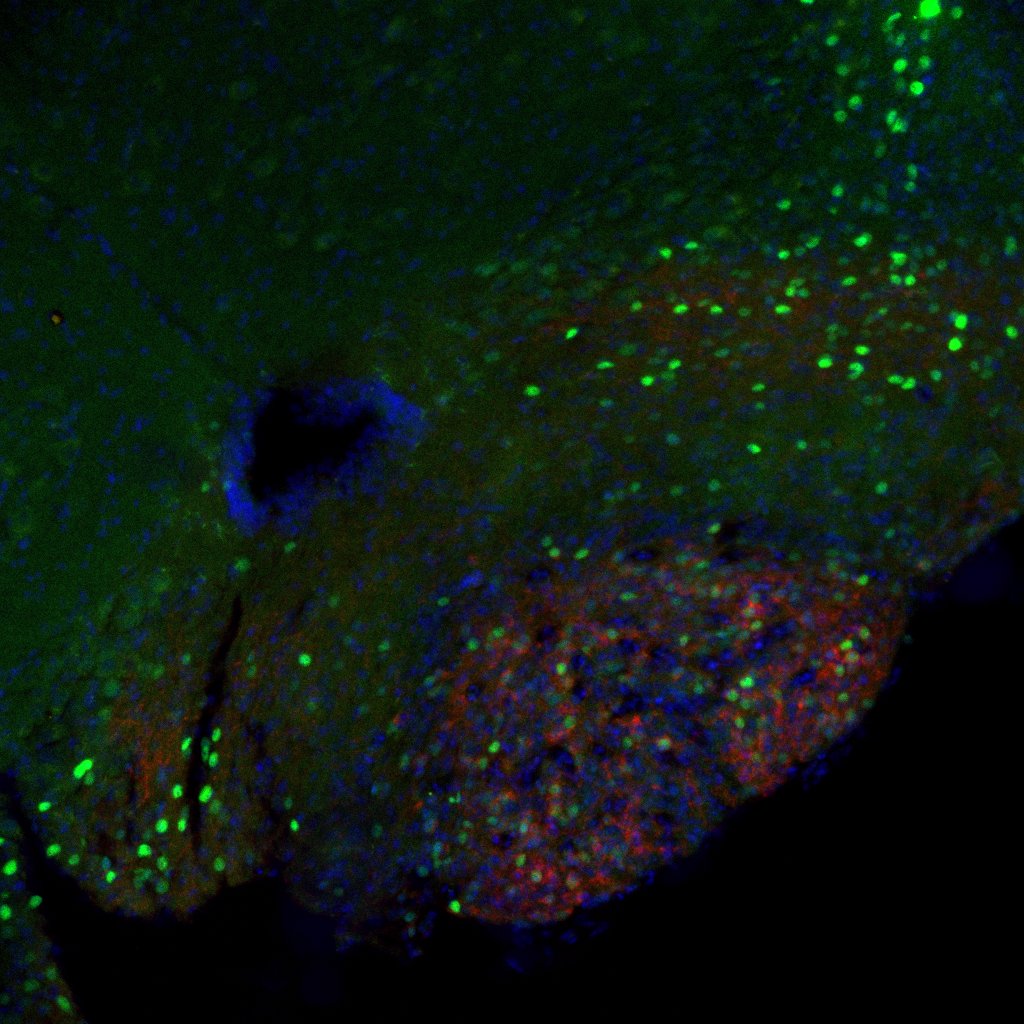

Supplement: Supplementary file 3 — Original pictures of cFos and Cy5 drug appearance shown in Fig. 3, including replicates used for quantification. [file 42255_2023_931_MOESM3_ESM.zip › Raw Data Figure 3/Figure 3A-C/Unadjusted/Fig3A_GLP1_Cy5_3.jpg]

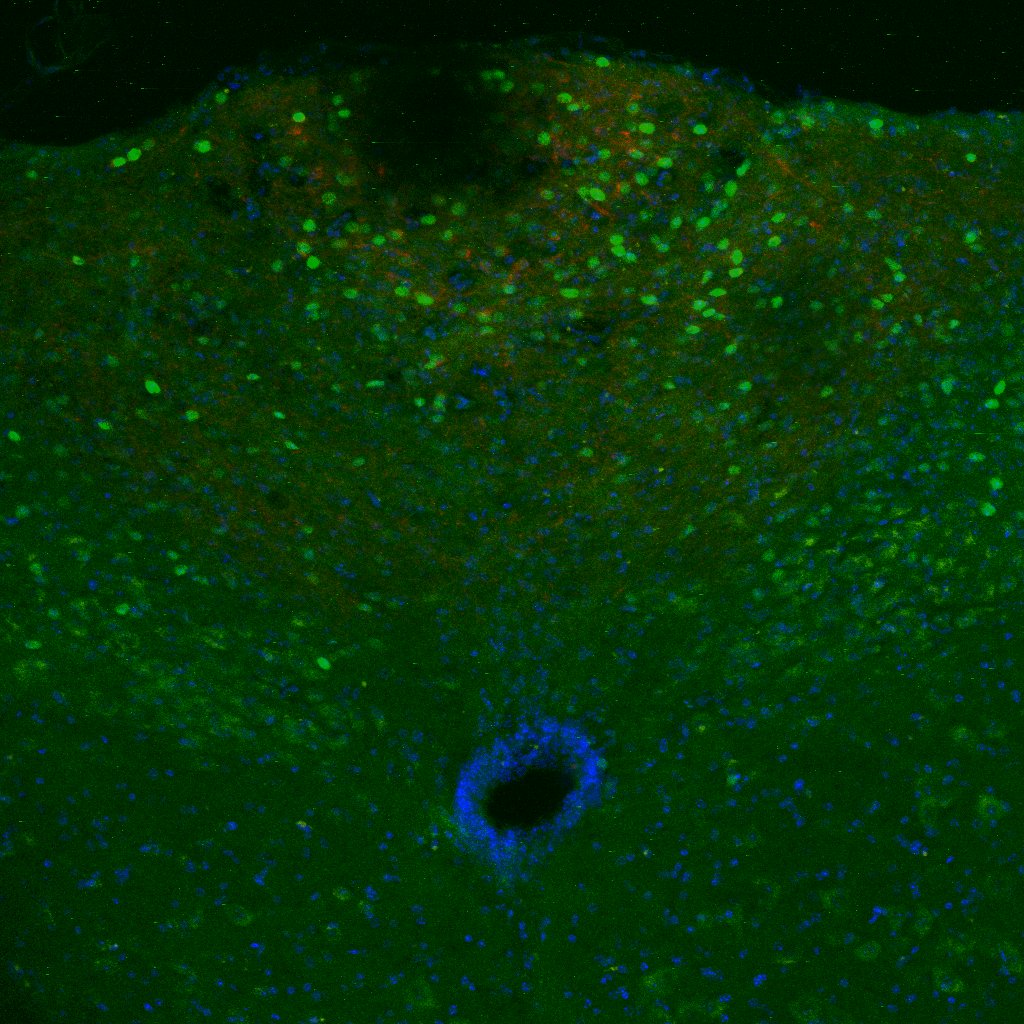

Supplement: Supplementary file 3 — Original pictures of cFos and Cy5 drug appearance shown in Fig. 3, including replicates used for quantification. [file 42255_2023_931_MOESM3_ESM.zip › Raw Data Figure 3/Figure 3A-C/Unadjusted/Fig3A_GLP1_Cy5_1.jpg]

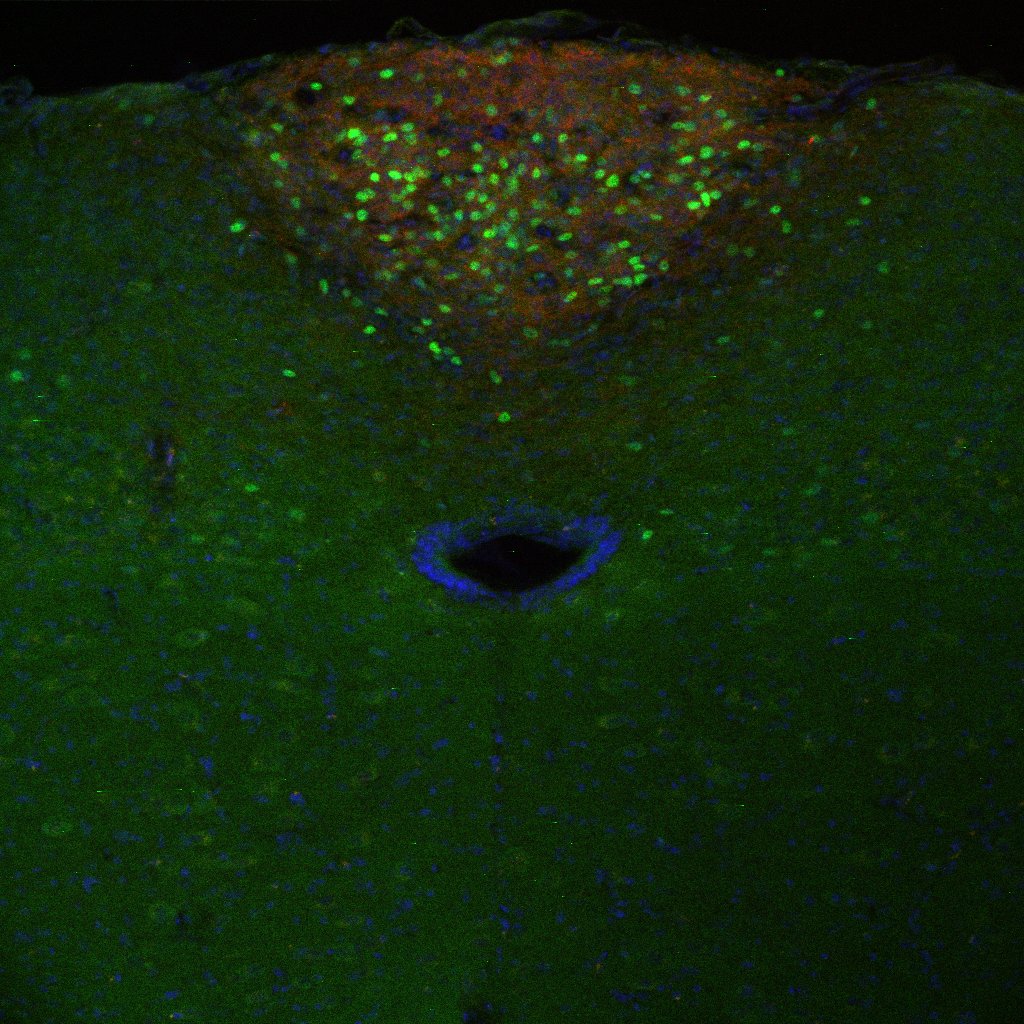

Supplement: Supplementary file 3 — Original pictures of cFos and Cy5 drug appearance shown in Fig. 3, including replicates used for quantification. [file 42255_2023_931_MOESM3_ESM.zip › Raw Data Figure 3/Figure 3A-C/Unadjusted/Fig3A_GIP_Cy5_1.jpg]

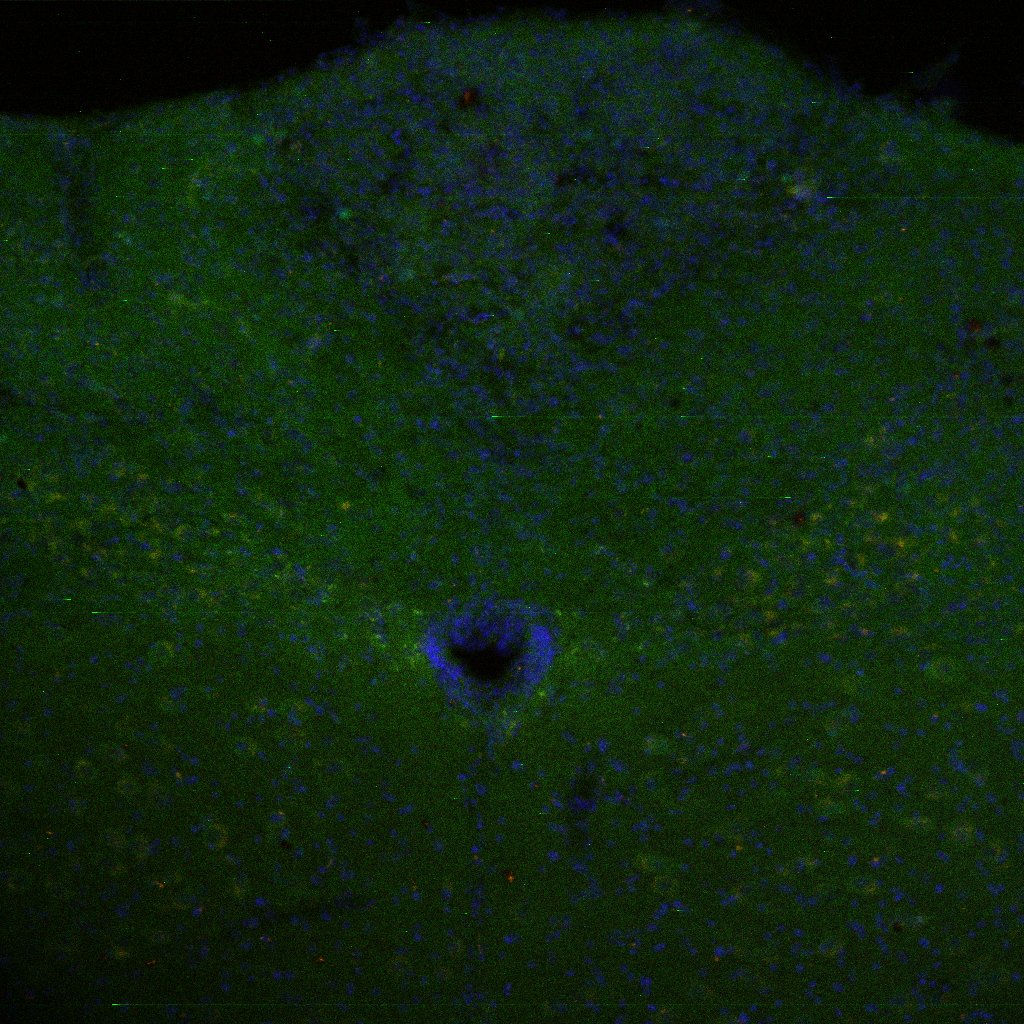

Supplement: Supplementary file 3 — Original pictures of cFos and Cy5 drug appearance shown in Fig. 3, including replicates used for quantification. [file 42255_2023_931_MOESM3_ESM.zip › Raw Data Figure 3/Figure 3A-C/Unadjusted/Fig3A_Veh_2.jpg]

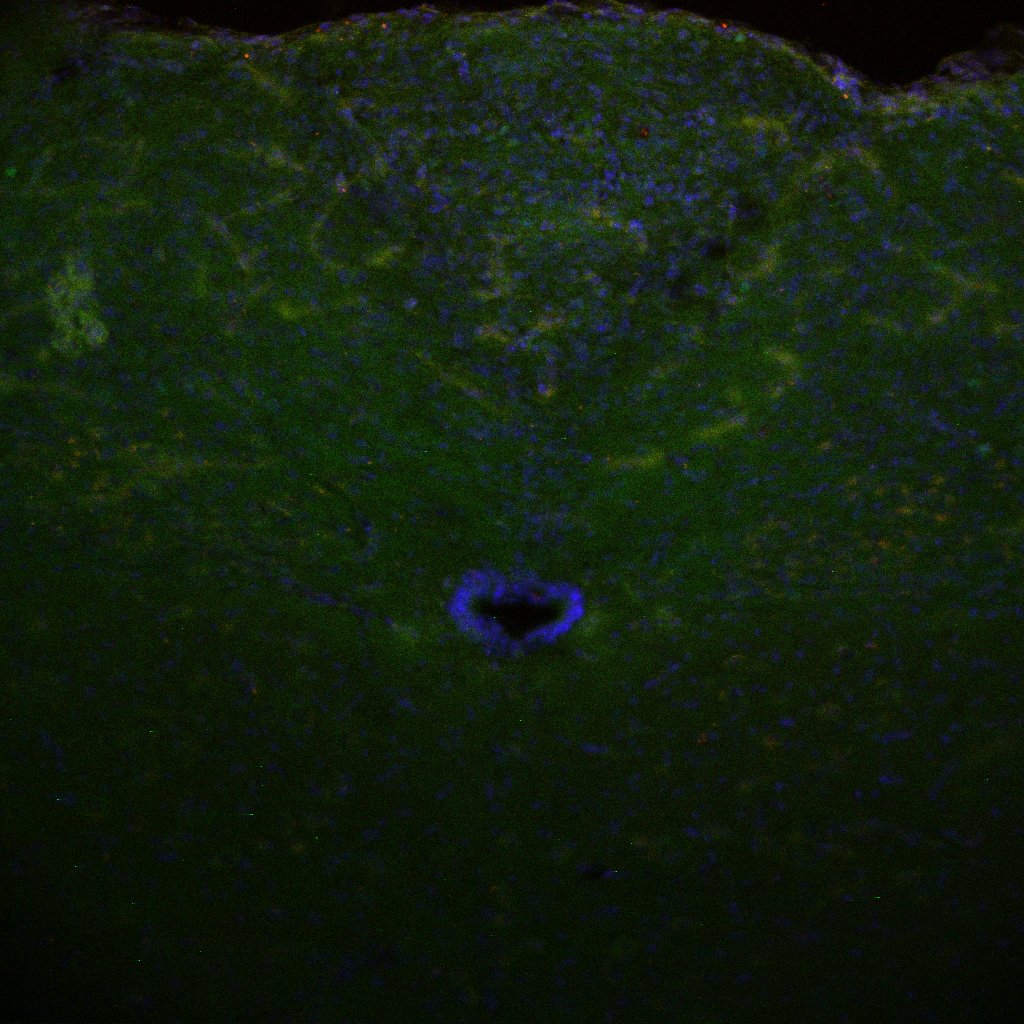

Supplement: Supplementary file 3 — Original pictures of cFos and Cy5 drug appearance shown in Fig. 3, including replicates used for quantification. [file 42255_2023_931_MOESM3_ESM.zip › Raw Data Figure 3/Figure 3A-C/Unadjusted/Fig3A_Veh_3.jpg]

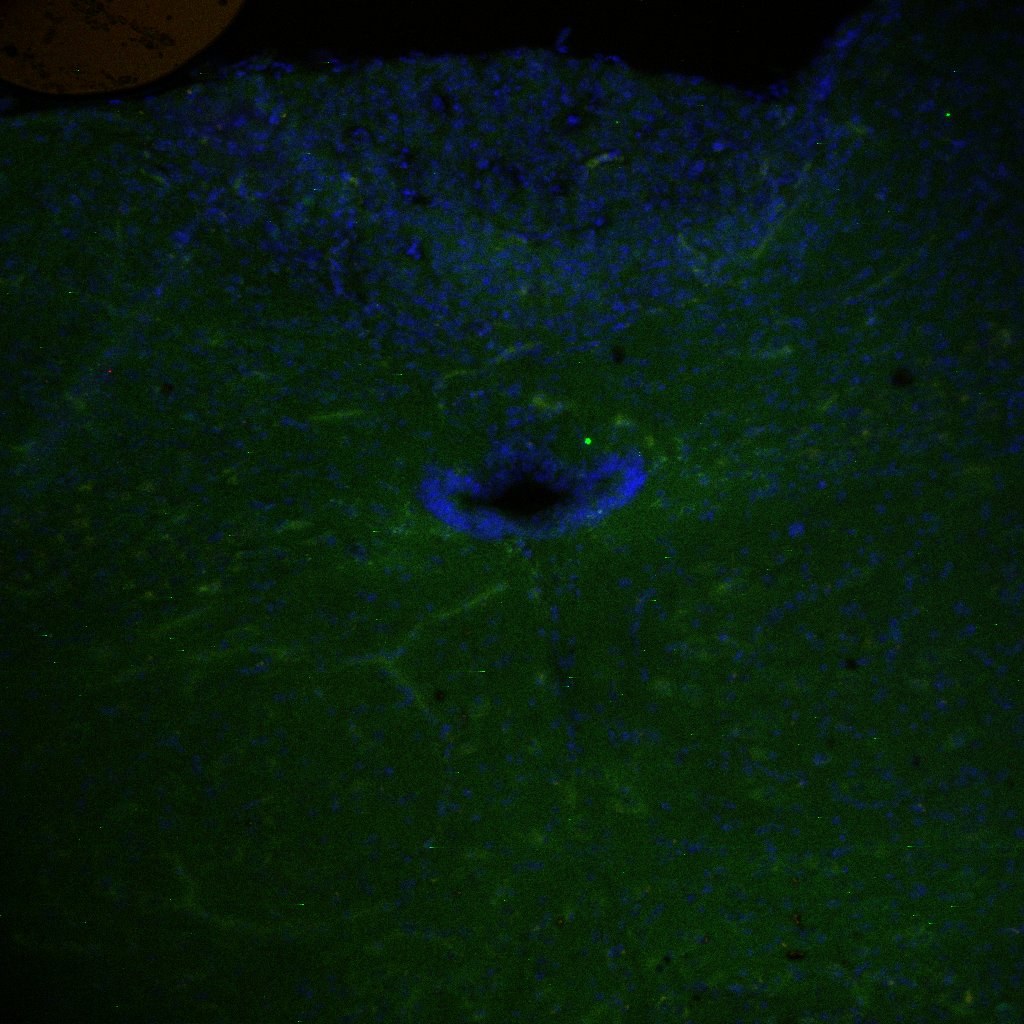

Supplement: Supplementary file 3 — Original pictures of cFos and Cy5 drug appearance shown in Fig. 3, including replicates used for quantification. [file 42255_2023_931_MOESM3_ESM.zip › Raw Data Figure 3/Figure 3A-C/Unadjusted/Fig3A_Veh_1.jpg]

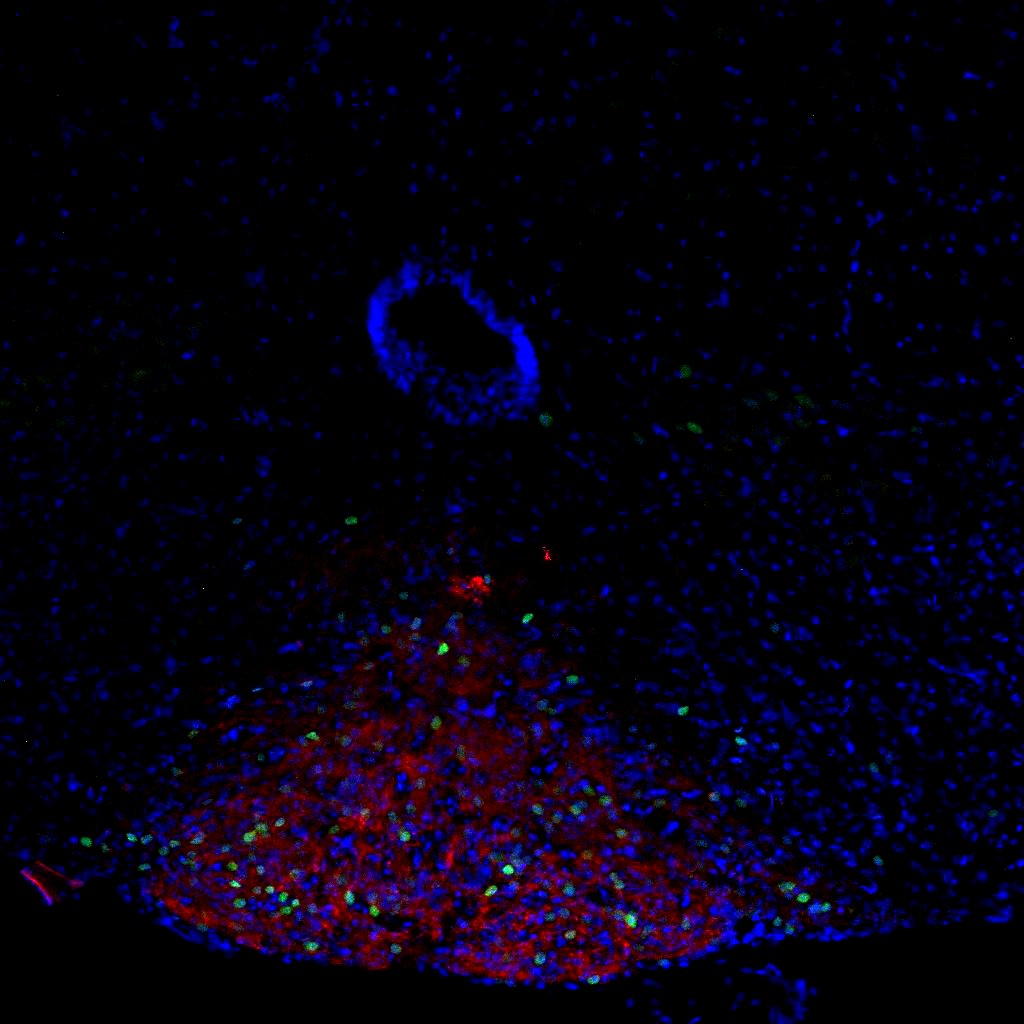

Supplement: Supplementary file 3 — Original pictures of cFos and Cy5 drug appearance shown in Fig. 3, including replicates used for quantification. [file 42255_2023_931_MOESM3_ESM.zip › Raw Data Figure 3/Figure 3D and E/Adjusted/07-WT_GIPcy5_3a.jpg]

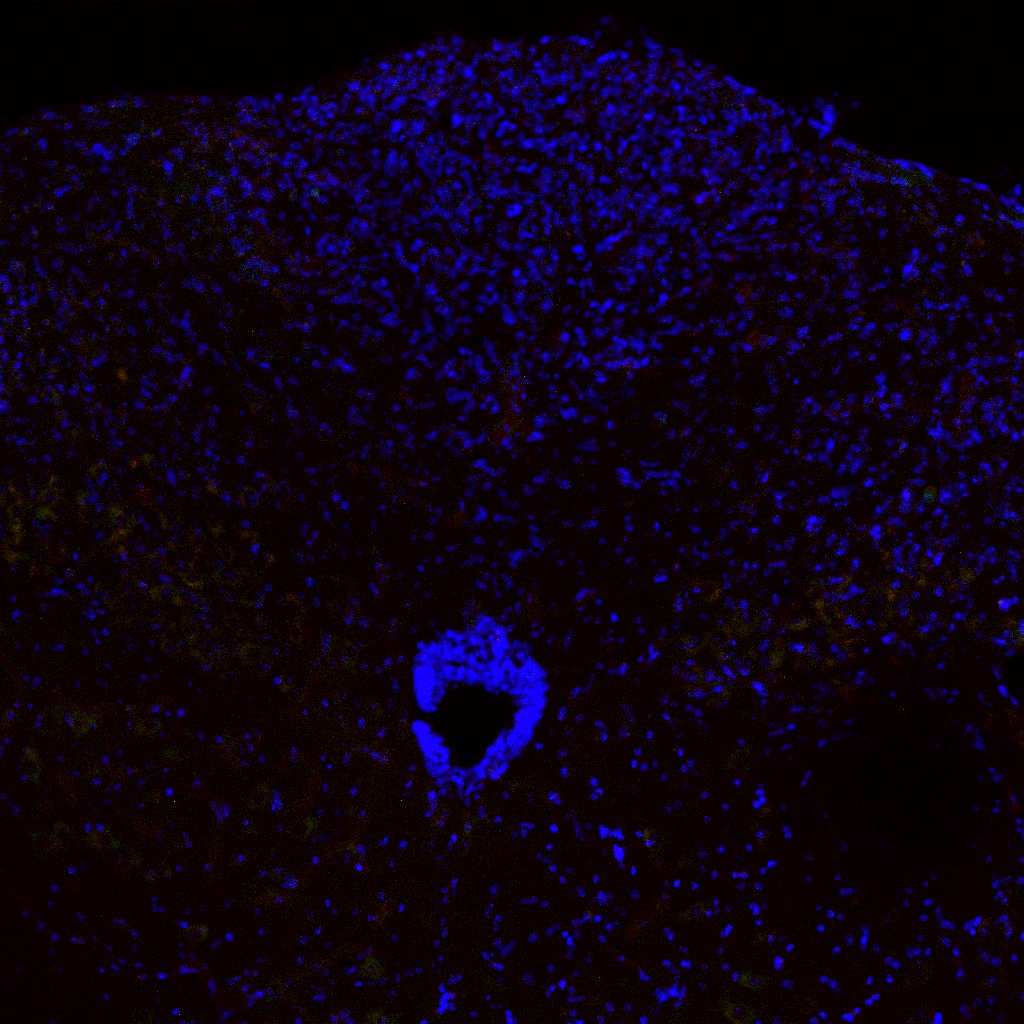

Supplement: Supplementary file 3 — Original pictures of cFos and Cy5 drug appearance shown in Fig. 3, including replicates used for quantification. [file 42255_2023_931_MOESM3_ESM.zip › Raw Data Figure 3/Figure 3D and E/Adjusted/03-WT_Veh_3a.jpg]

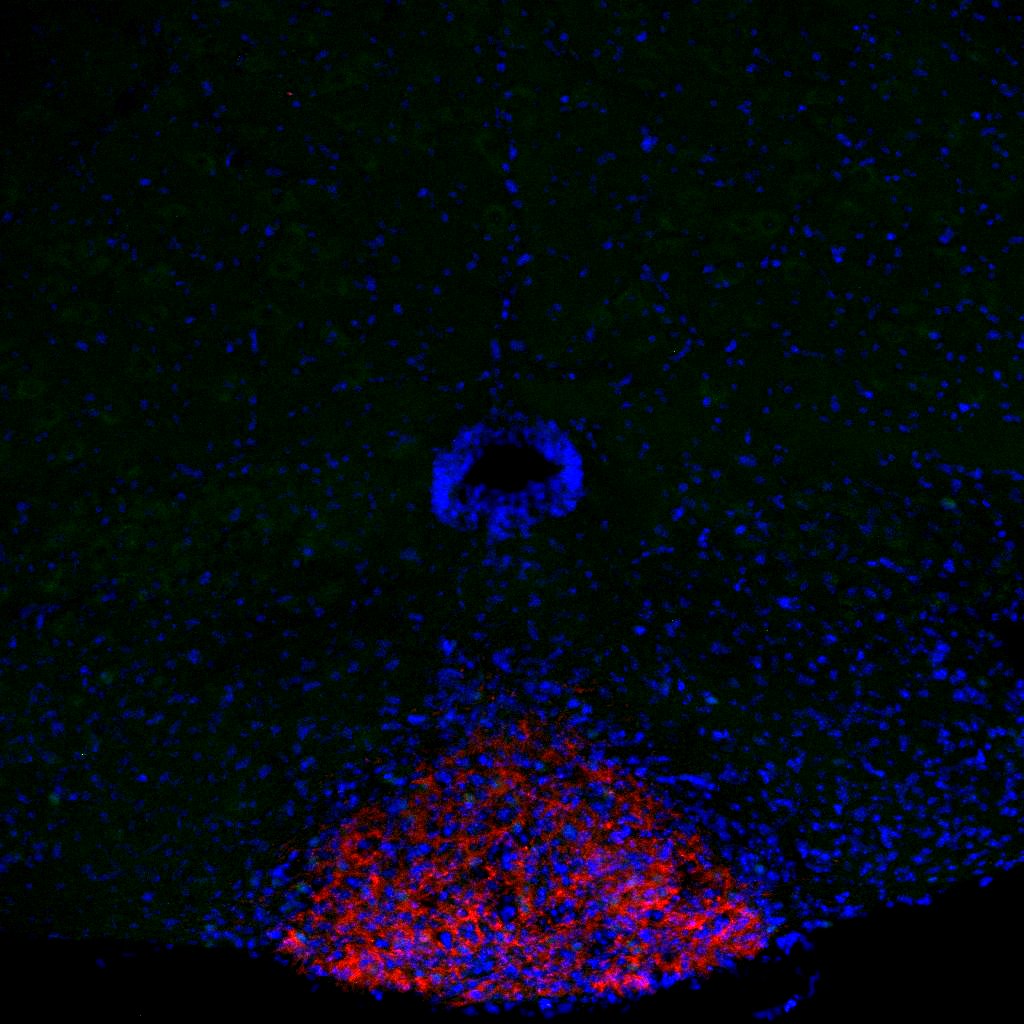

Supplement: Supplementary file 3 — Original pictures of cFos and Cy5 drug appearance shown in Fig. 3, including replicates used for quantification. [file 42255_2023_931_MOESM3_ESM.zip › Raw Data Figure 3/Figure 3D and E/Adjusted/15-KO_GIPcy5_3a.jpg]

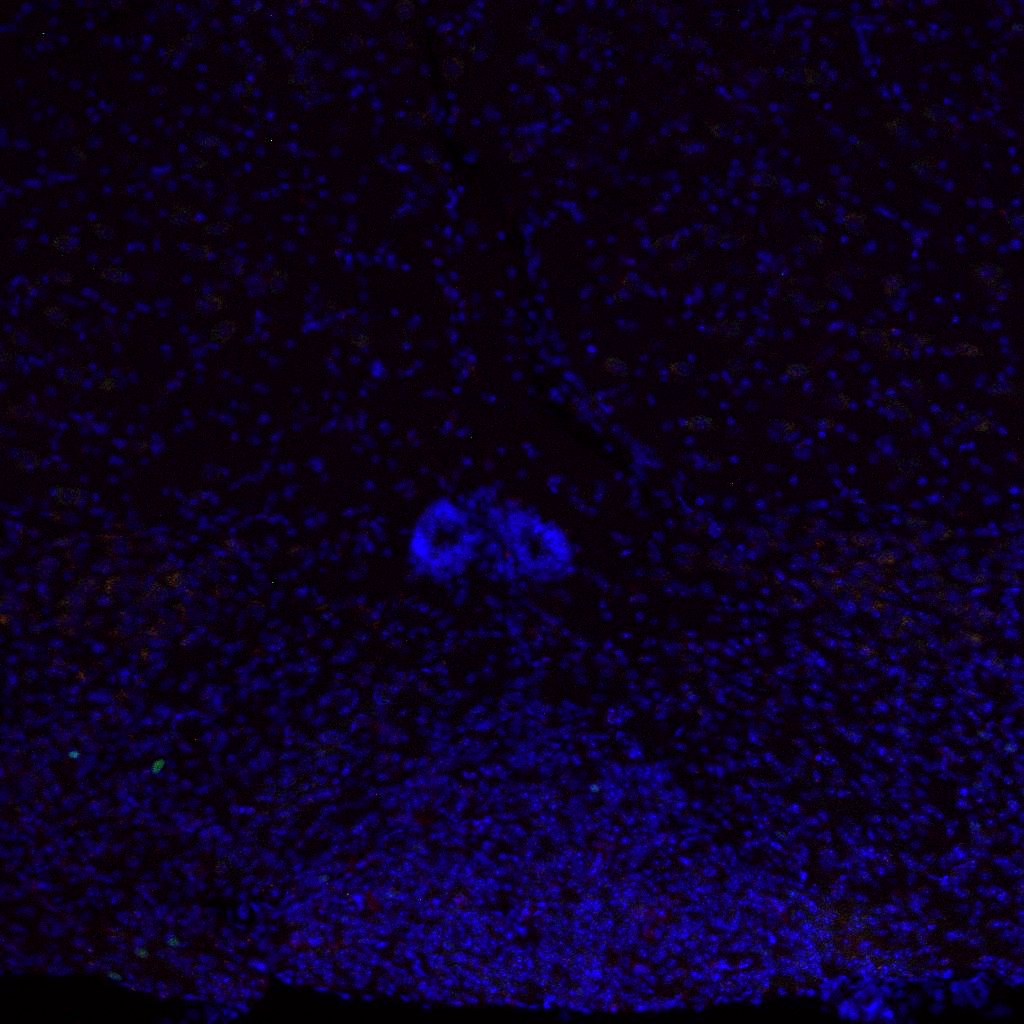

Supplement: Supplementary file 3 — Original pictures of cFos and Cy5 drug appearance shown in Fig. 3, including replicates used for quantification. [file 42255_2023_931_MOESM3_ESM.zip › Raw Data Figure 3/Figure 3D and E/Adjusted/04-WT_Veh_4a.jpg]

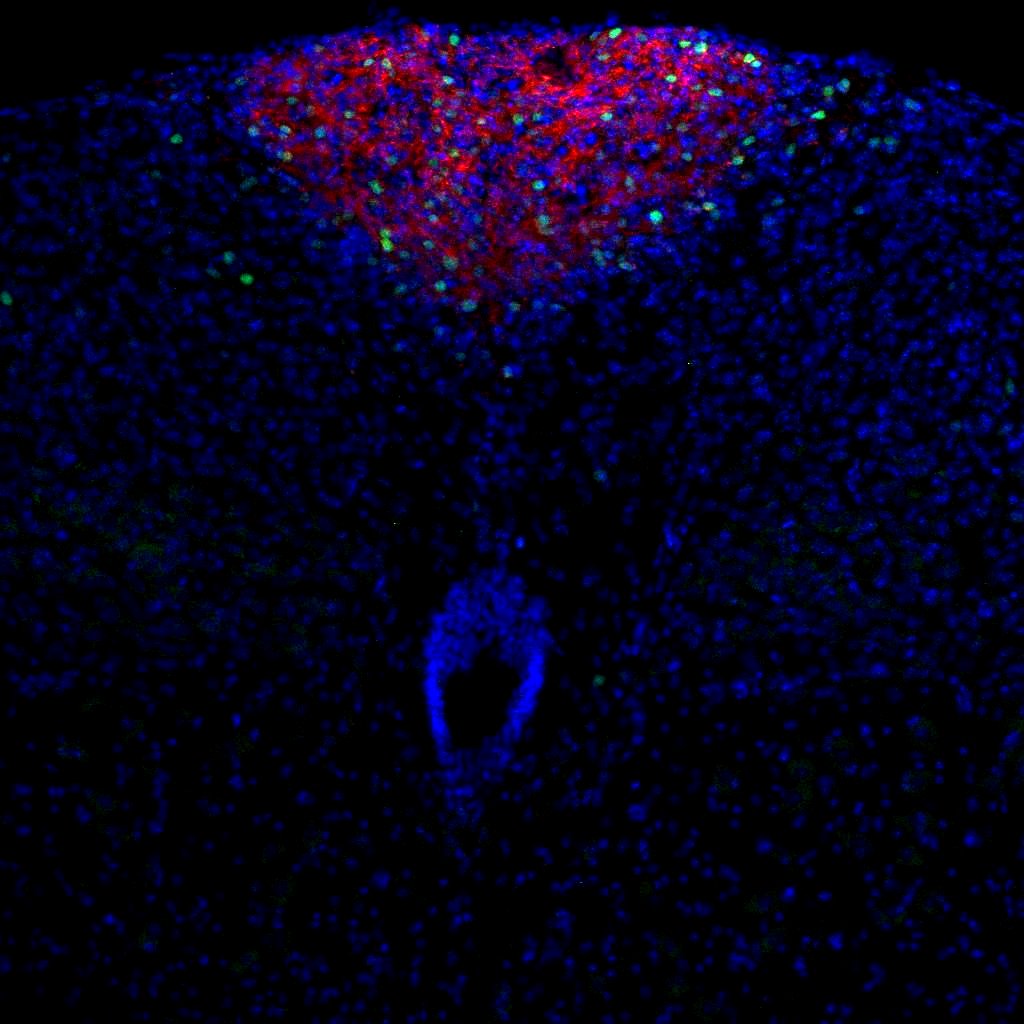

Supplement: Supplementary file 3 — Original pictures of cFos and Cy5 drug appearance shown in Fig. 3, including replicates used for quantification. [file 42255_2023_931_MOESM3_ESM.zip › Raw Data Figure 3/Figure 3D and E/Adjusted/08-WT_GIPcy5_4a.jpg]

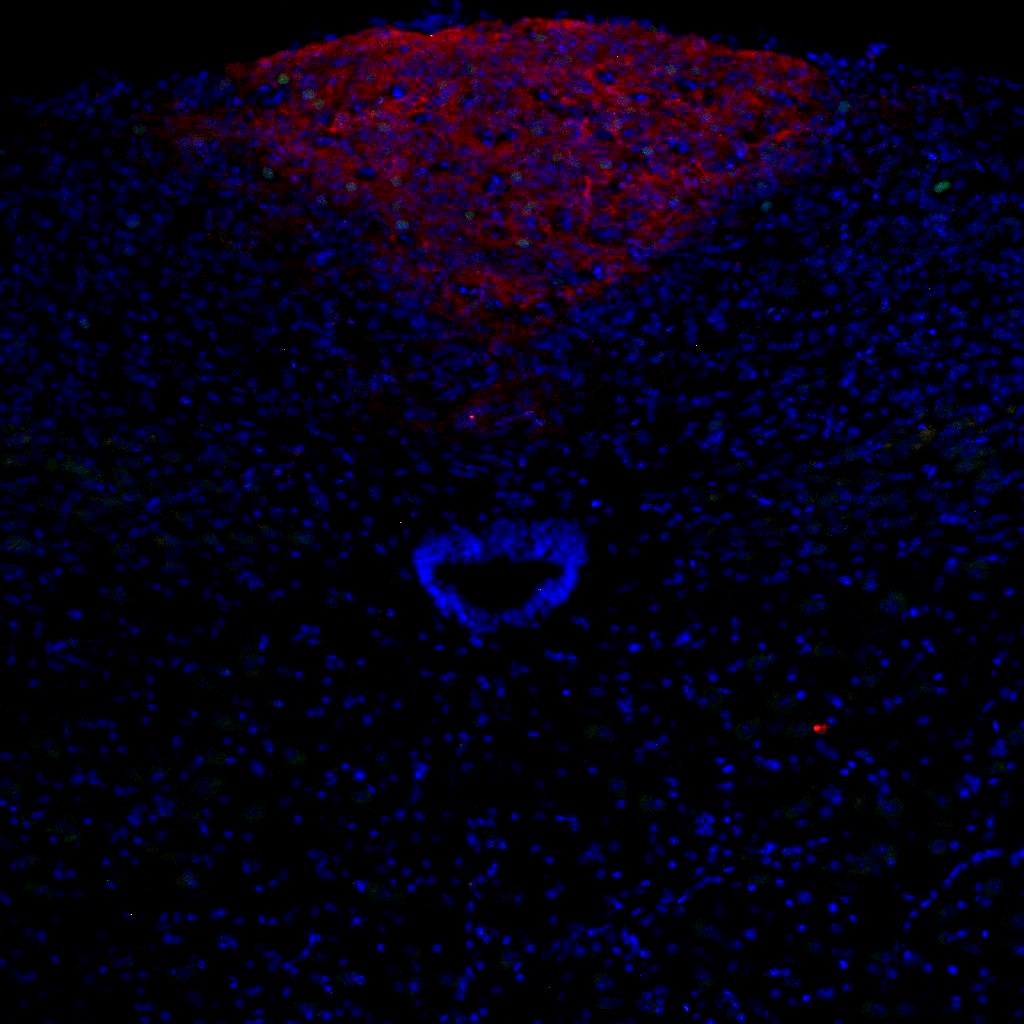

Supplement: Supplementary file 3 — Original pictures of cFos and Cy5 drug appearance shown in Fig. 3, including replicates used for quantification. [file 42255_2023_931_MOESM3_ESM.zip › Raw Data Figure 3/Figure 3D and E/Adjusted/13-KO_GIPcy5_1a.jpg]

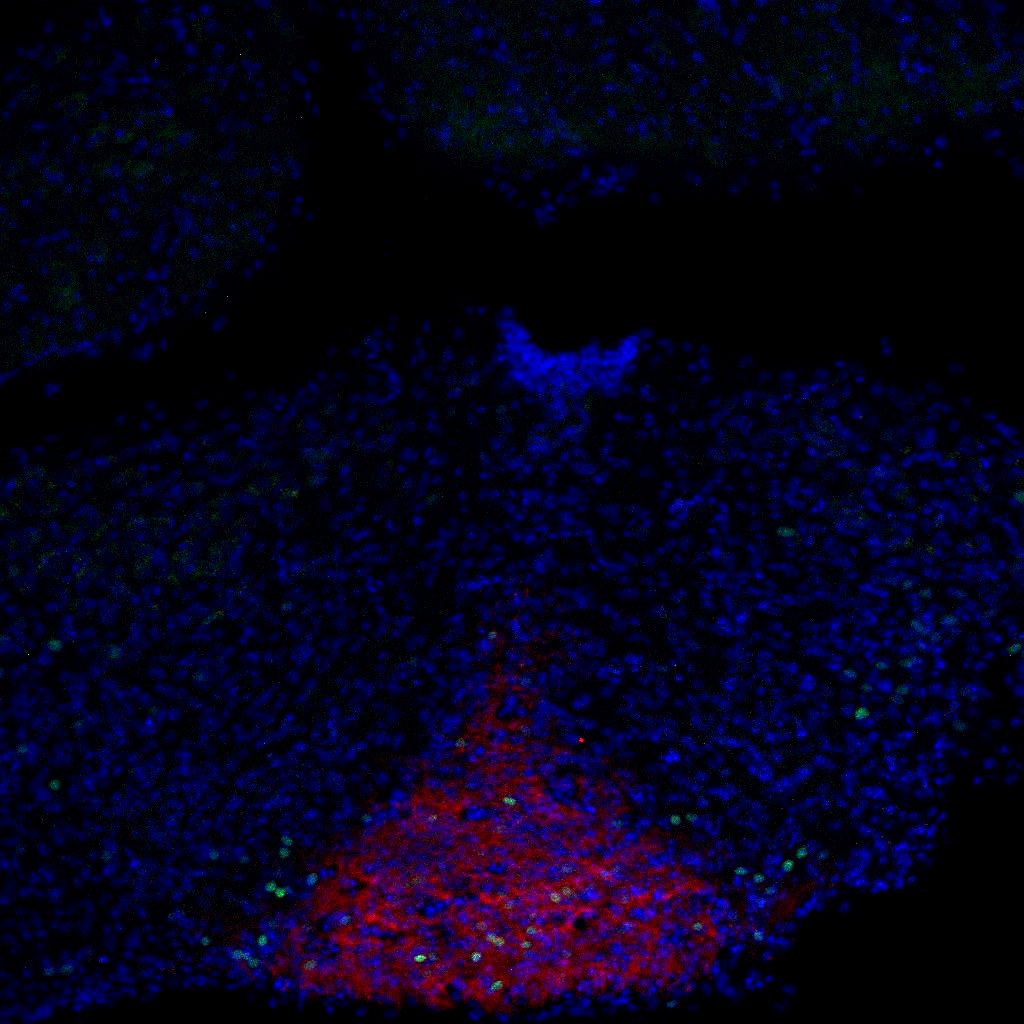

Supplement: Supplementary file 3 — Original pictures of cFos and Cy5 drug appearance shown in Fig. 3, including replicates used for quantification. [file 42255_2023_931_MOESM3_ESM.zip › Raw Data Figure 3/Figure 3D and E/Adjusted/14-KO_GIPcy5_2a.jpg]

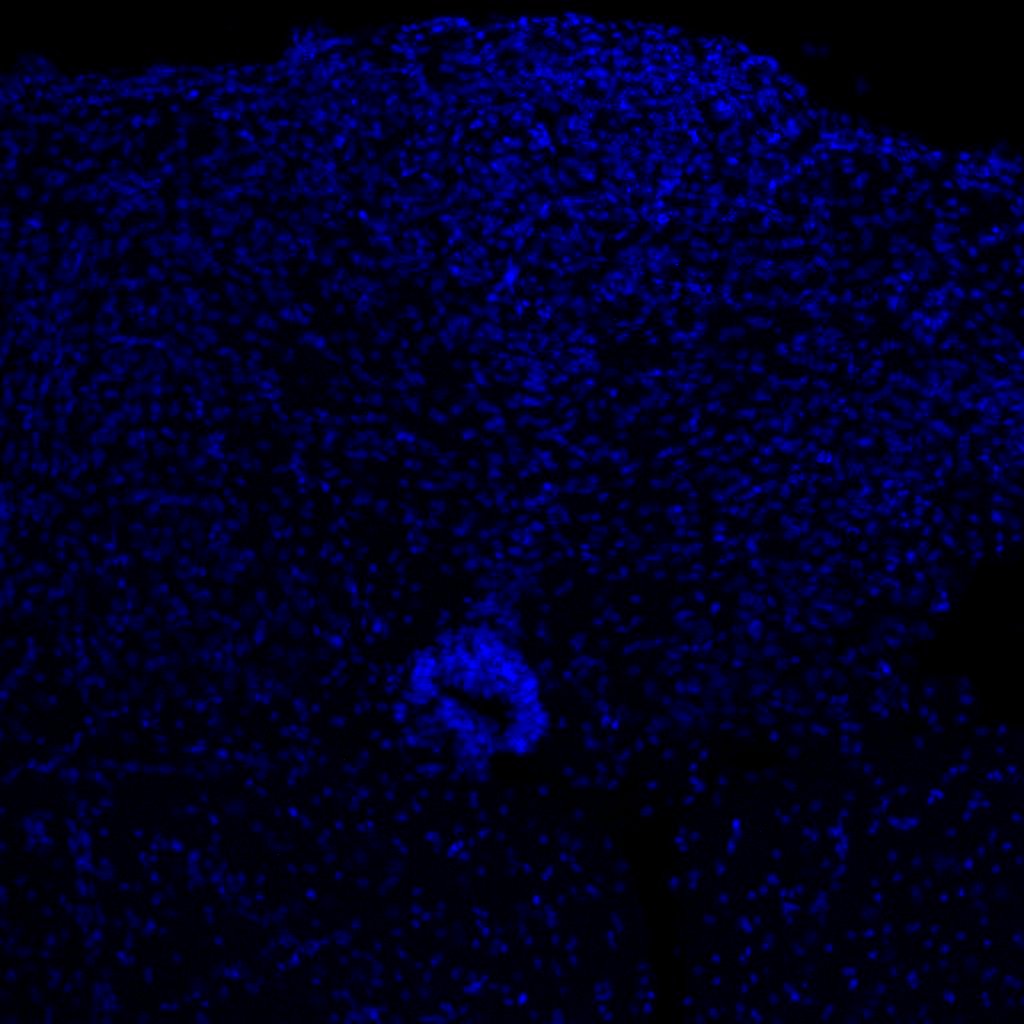

Supplement: Supplementary file 3 — Original pictures of cFos and Cy5 drug appearance shown in Fig. 3, including replicates used for quantification. [file 42255_2023_931_MOESM3_ESM.zip › Raw Data Figure 3/Figure 3D and E/Adjusted/10-KO_Veh_2a.jpg]

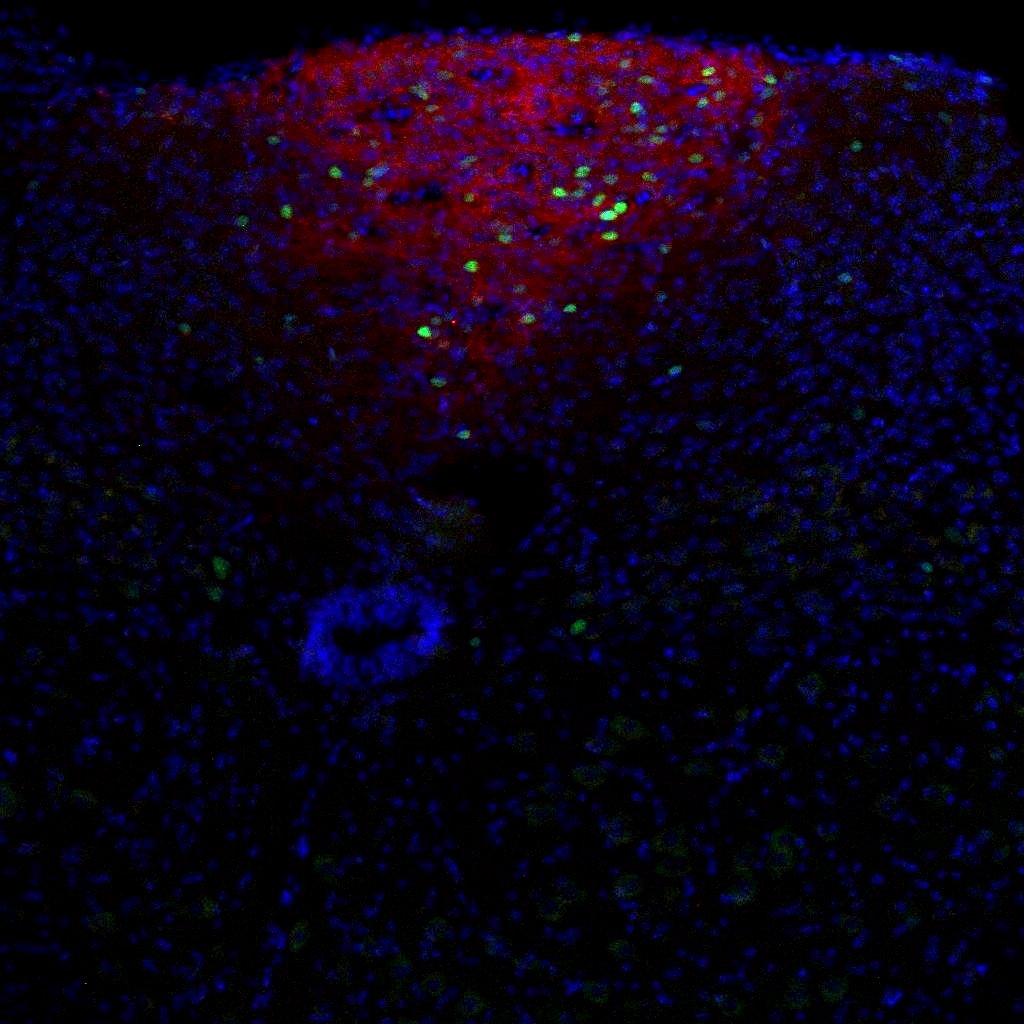

Supplement: Supplementary file 3 — Original pictures of cFos and Cy5 drug appearance shown in Fig. 3, including replicates used for quantification. [file 42255_2023_931_MOESM3_ESM.zip › Raw Data Figure 3/Figure 3D and E/Adjusted/06-WT_GIPcy5_2a.jpg]

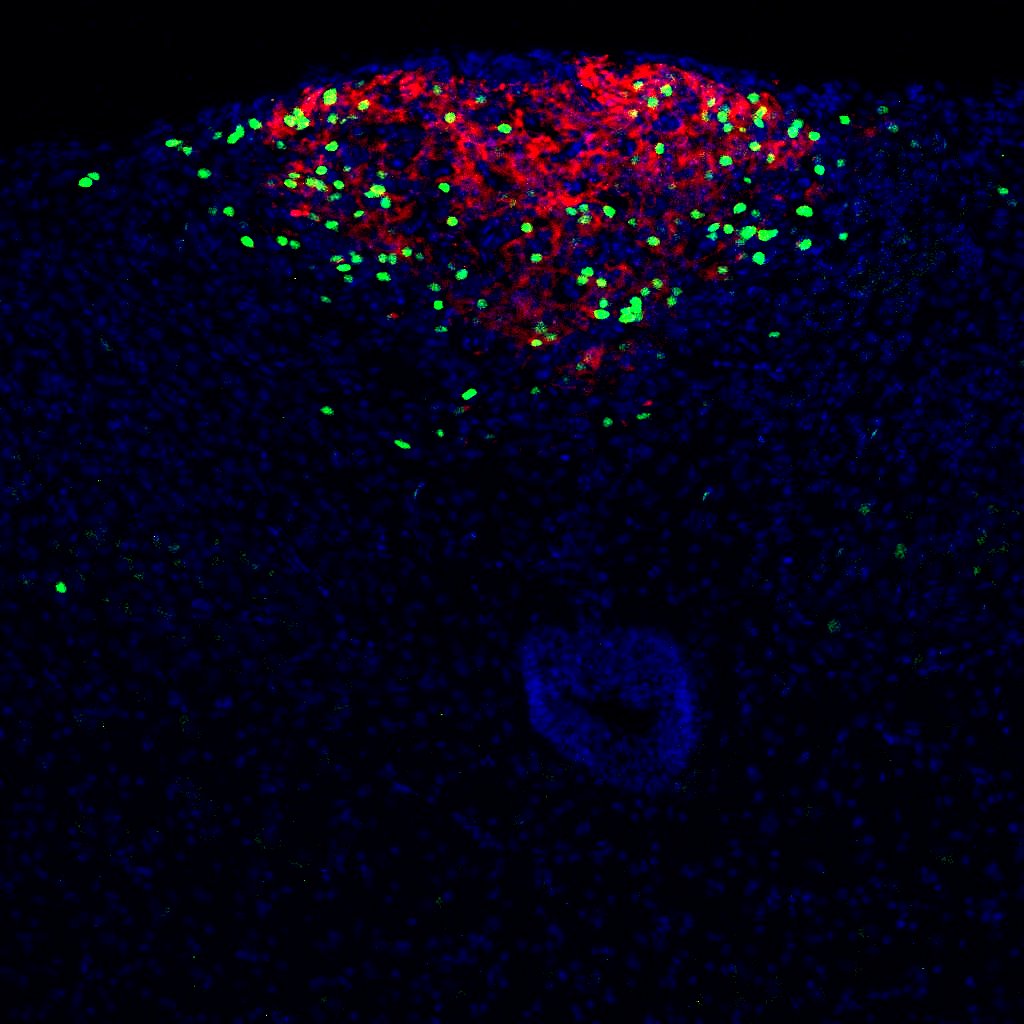

Supplement: Supplementary file 3 — Original pictures of cFos and Cy5 drug appearance shown in Fig. 3, including replicates used for quantification. [file 42255_2023_931_MOESM3_ESM.zip › Raw Data Figure 3/Figure 3D and E/Adjusted/05-WT_GIPcy5_1aa.jpg]

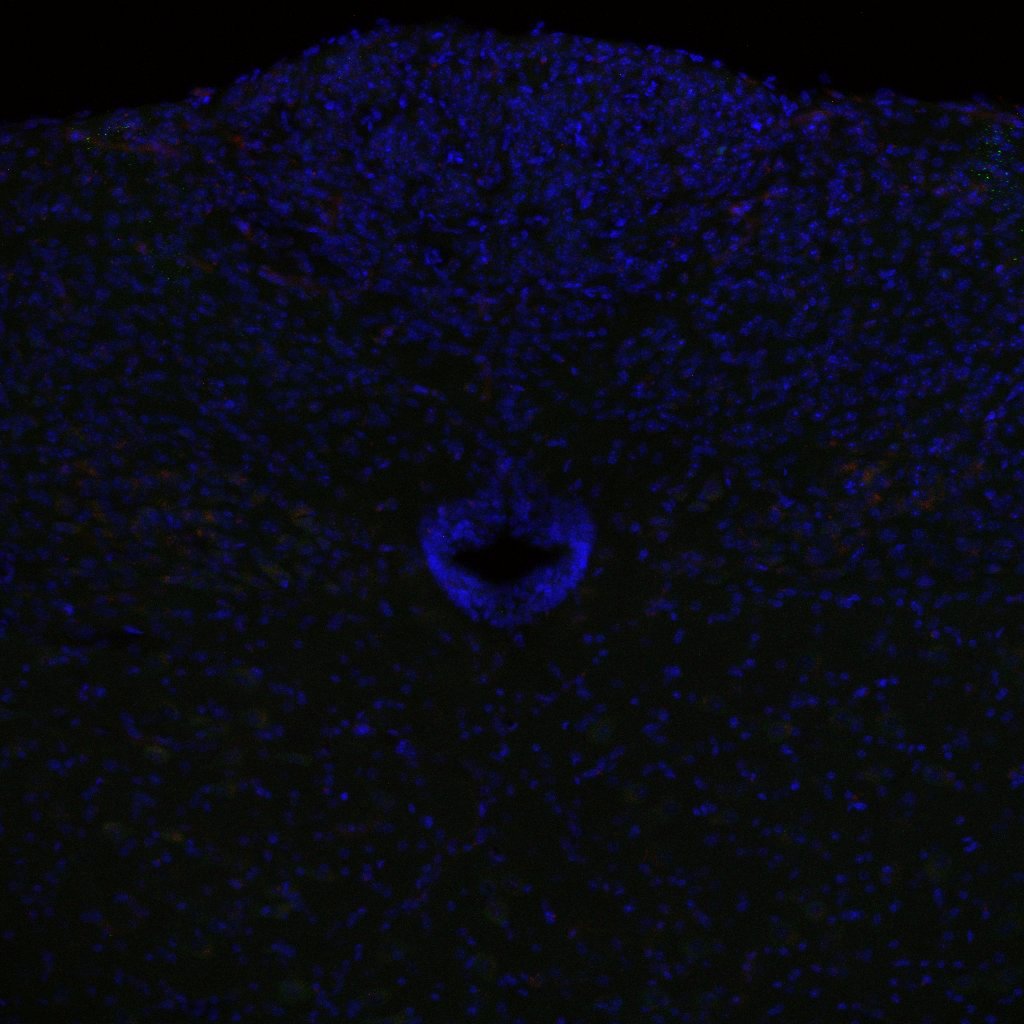

Supplement: Supplementary file 3 — Original pictures of cFos and Cy5 drug appearance shown in Fig. 3, including replicates used for quantification. [file 42255_2023_931_MOESM3_ESM.zip › Raw Data Figure 3/Figure 3D and E/Adjusted/11-KO_Veh_3aa.jpg]

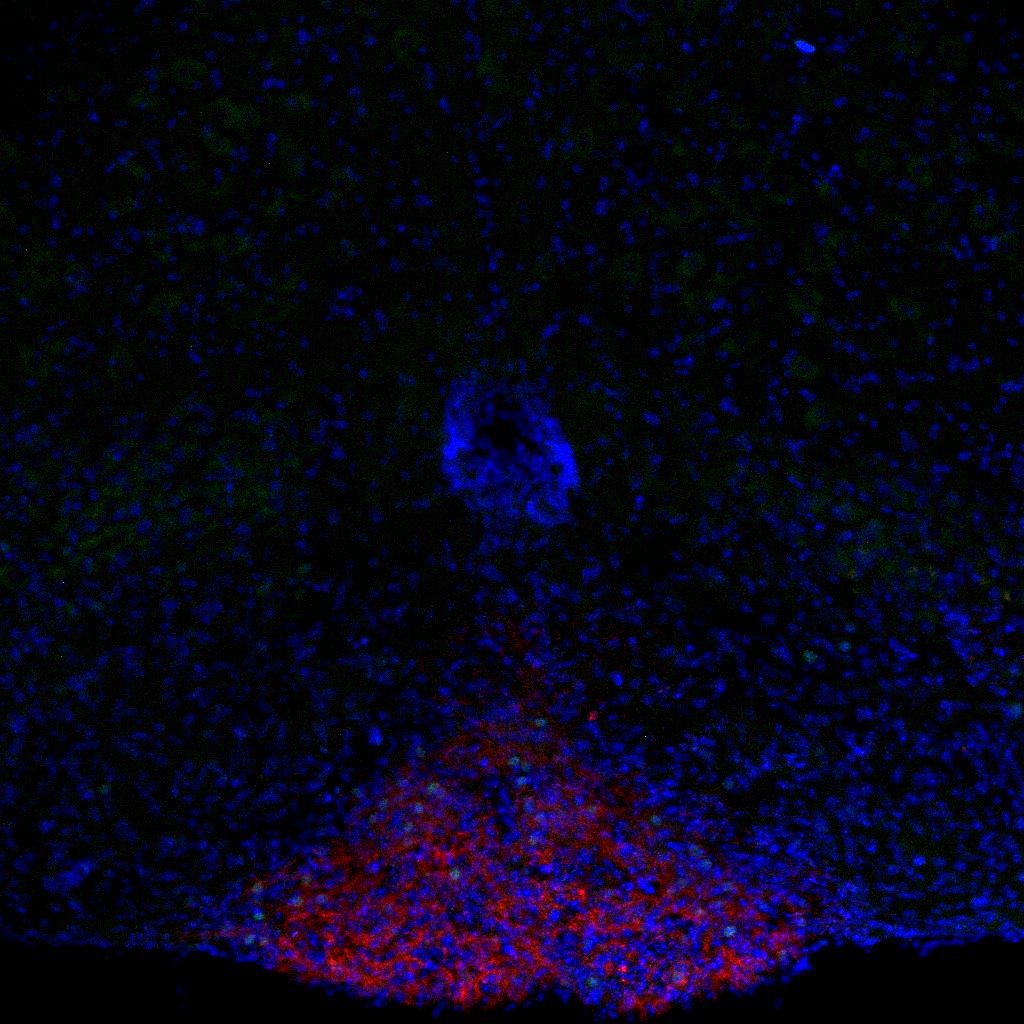

Supplement: Supplementary file 3 — Original pictures of cFos and Cy5 drug appearance shown in Fig. 3, including replicates used for quantification. [file 42255_2023_931_MOESM3_ESM.zip › Raw Data Figure 3/Figure 3D and E/Adjusted/16-KO_GIPcy5_4a.jpg]

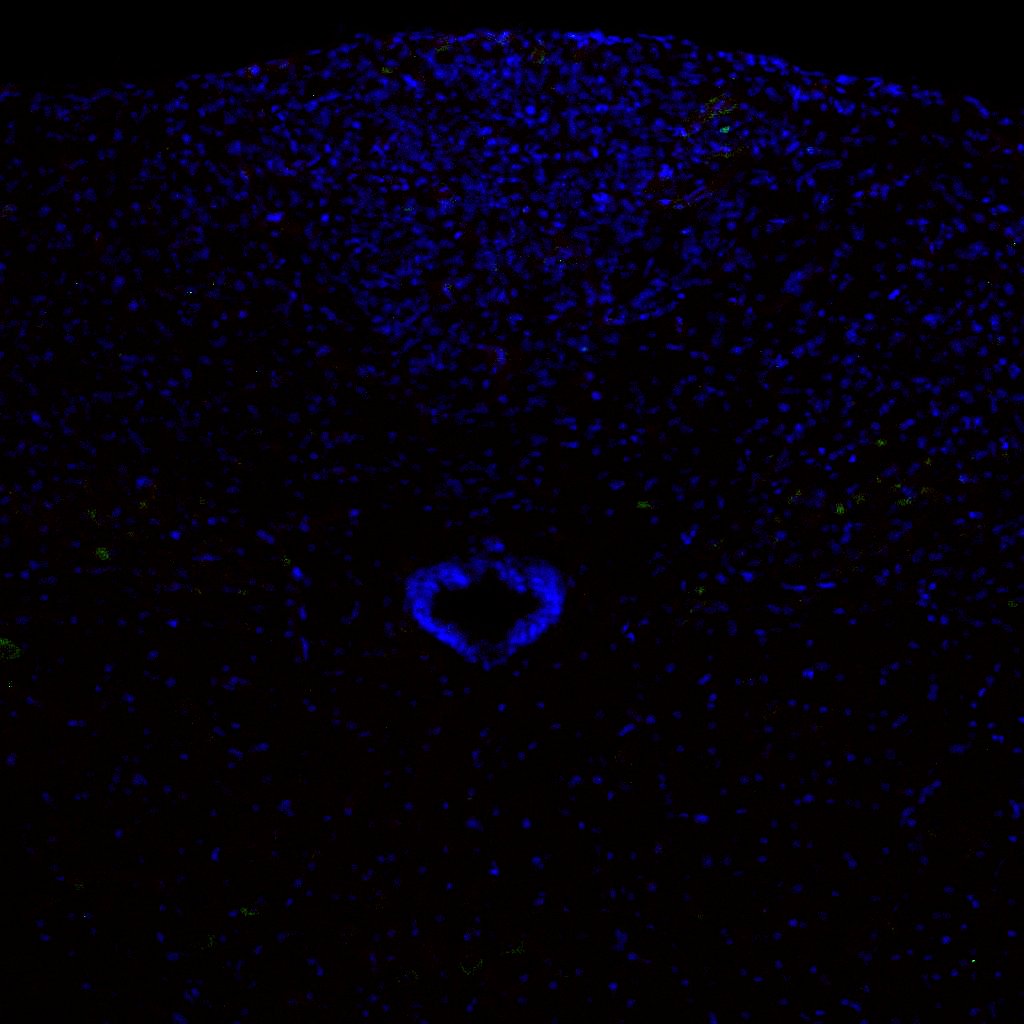

Supplement: Supplementary file 3 — Original pictures of cFos and Cy5 drug appearance shown in Fig. 3, including replicates used for quantification. [file 42255_2023_931_MOESM3_ESM.zip › Raw Data Figure 3/Figure 3D and E/Adjusted/01-WT_Veh_1aa.jpg]

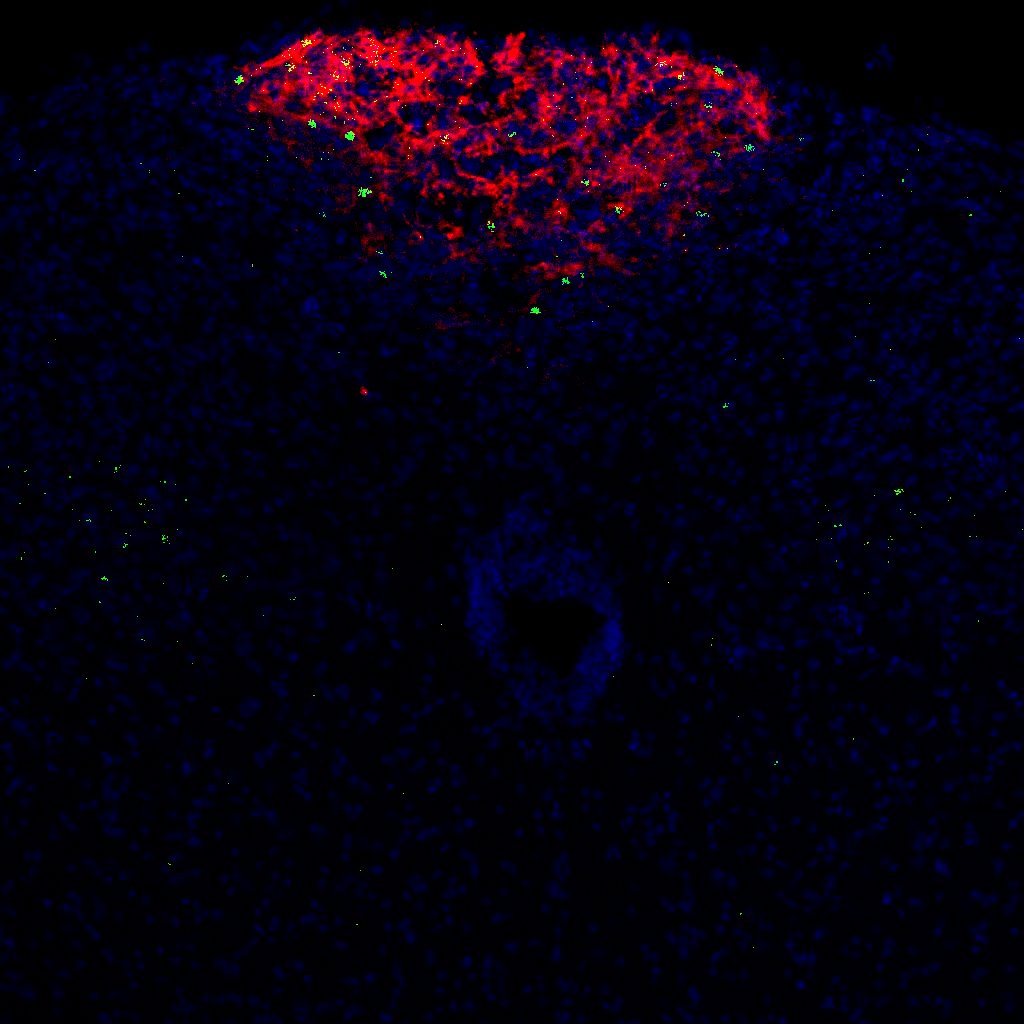

Supplement: Supplementary file 3 — Original pictures of cFos and Cy5 drug appearance shown in Fig. 3, including replicates used for quantification. [file 42255_2023_931_MOESM3_ESM.zip › Raw Data Figure 3/Figure 3D and E/Adjusted/17-KO_GIPcy5_5aa.jpg]

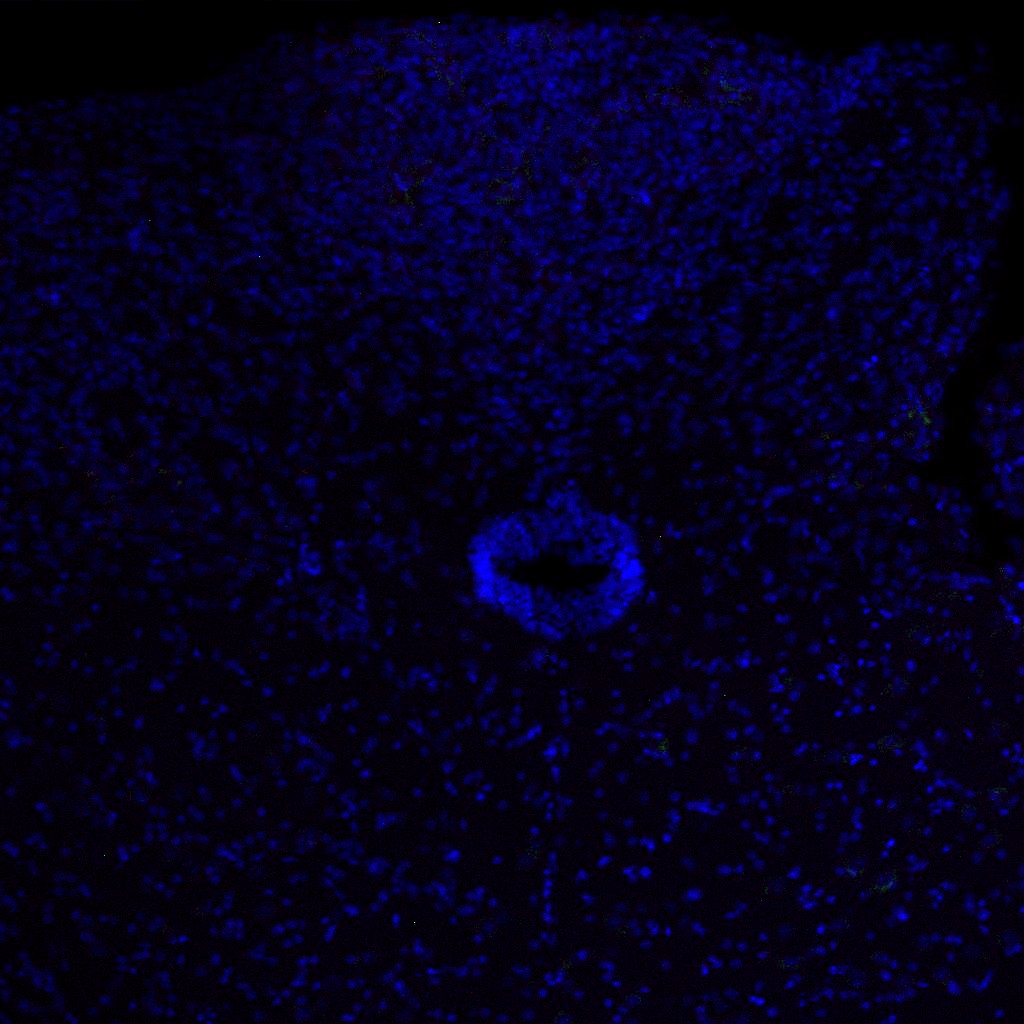

Supplement: Supplementary file 3 — Original pictures of cFos and Cy5 drug appearance shown in Fig. 3, including replicates used for quantification. [file 42255_2023_931_MOESM3_ESM.zip › Raw Data Figure 3/Figure 3D and E/Adjusted/02-WT_Veh_2a.jpg]

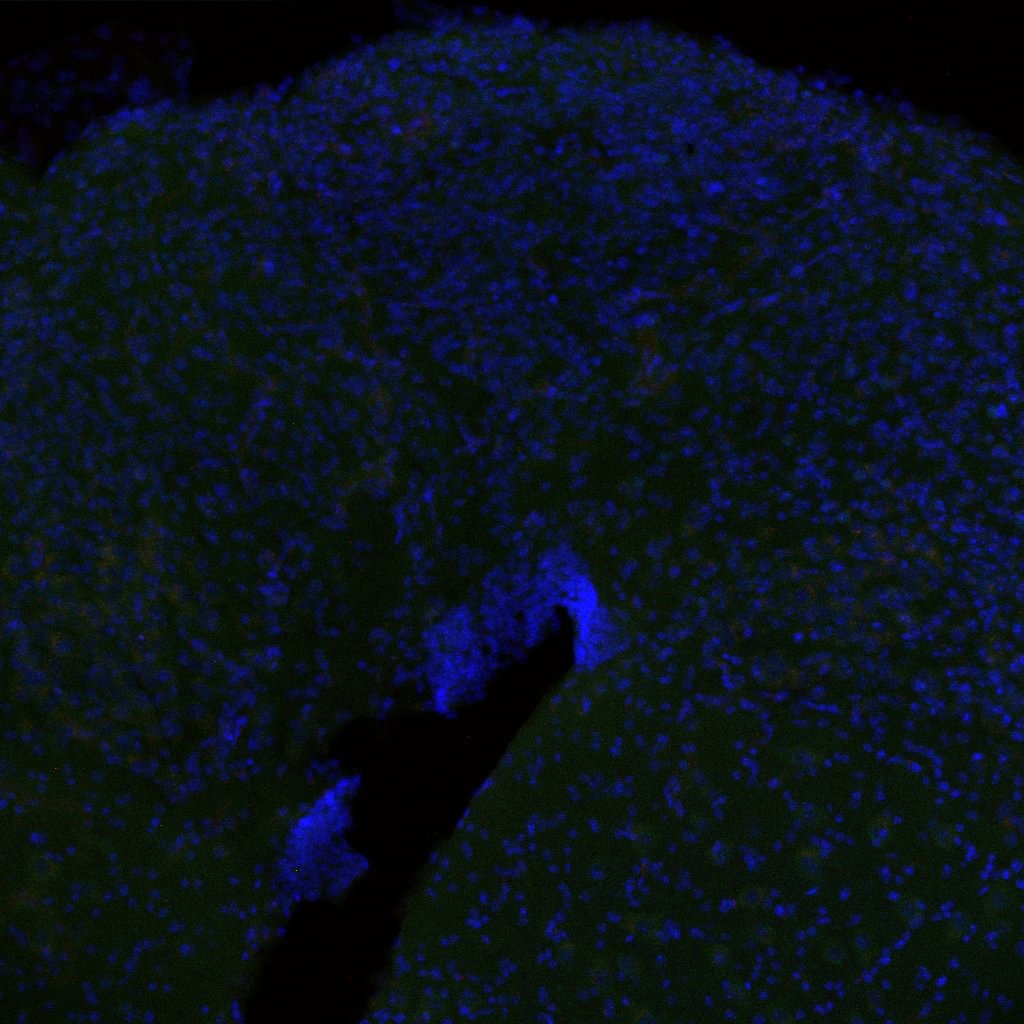

Supplement: Supplementary file 3 — Original pictures of cFos and Cy5 drug appearance shown in Fig. 3, including replicates used for quantification. [file 42255_2023_931_MOESM3_ESM.zip › Raw Data Figure 3/Figure 3D and E/Adjusted/12-KO_Veh_4a.jpg]

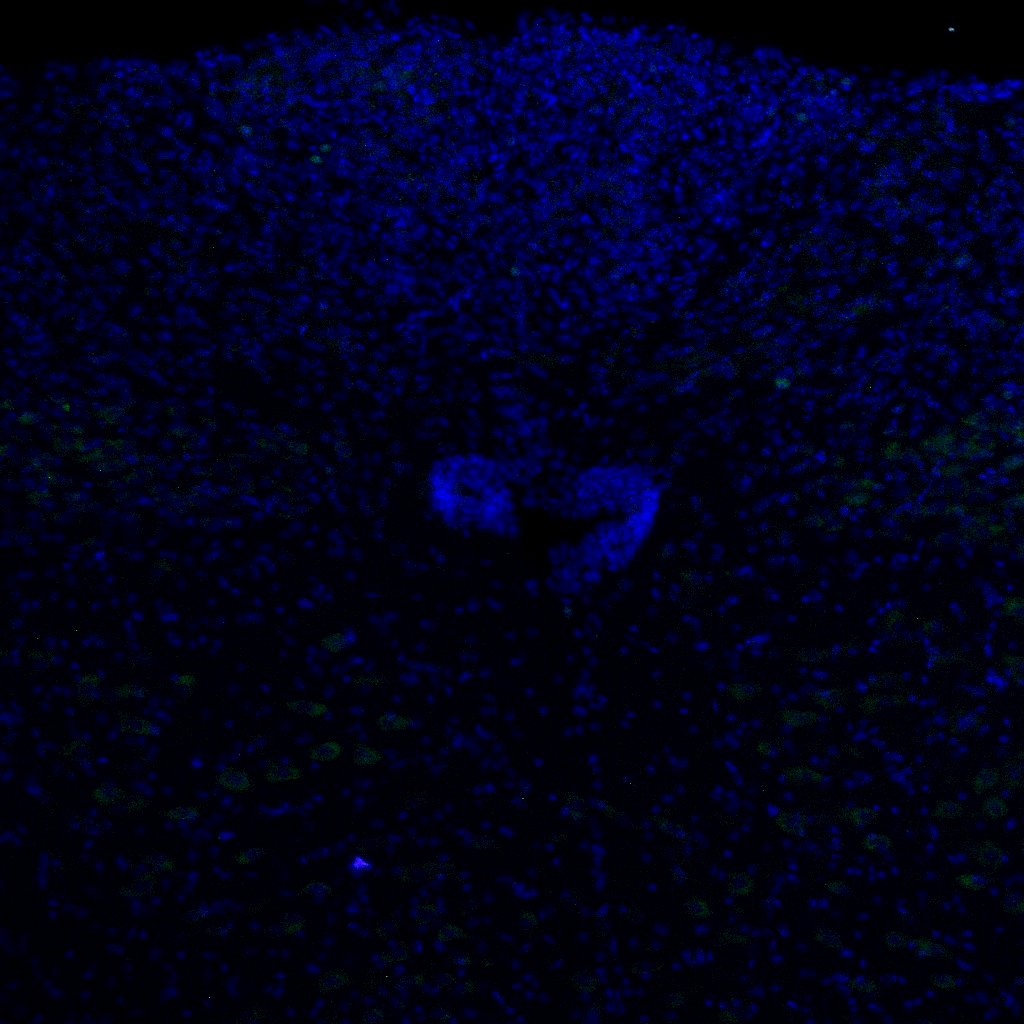

Supplement: Supplementary file 3 — Original pictures of cFos and Cy5 drug appearance shown in Fig. 3, including replicates used for quantification. [file 42255_2023_931_MOESM3_ESM.zip › Raw Data Figure 3/Figure 3D and E/Adjusted/09-KO_Veh_1a.jpg]

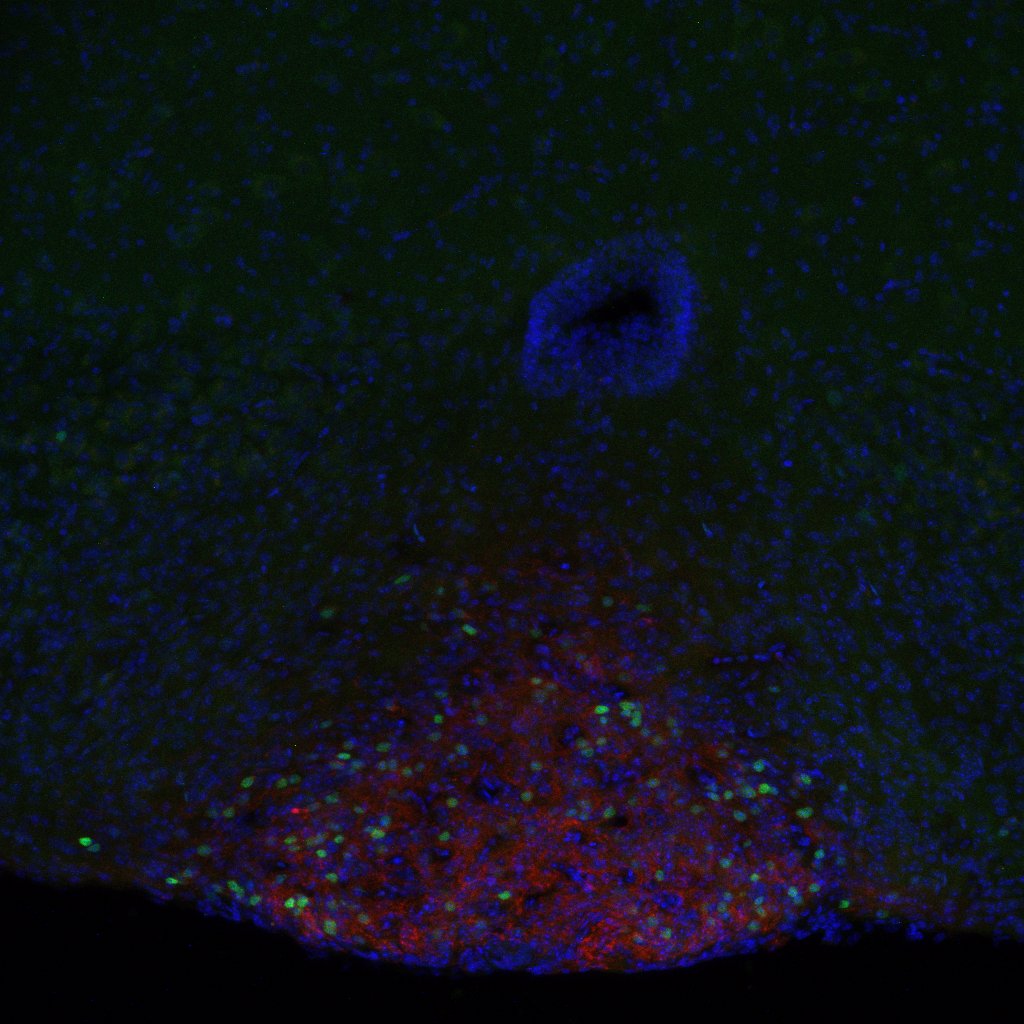

Supplement: Supplementary file 3 — Original pictures of cFos and Cy5 drug appearance shown in Fig. 3, including replicates used for quantification. [file 42255_2023_931_MOESM3_ESM.zip › Raw Data Figure 3/Figure 3D and E/Unadjusted/Fig3D_WT_GIPcy5_1.jpg]

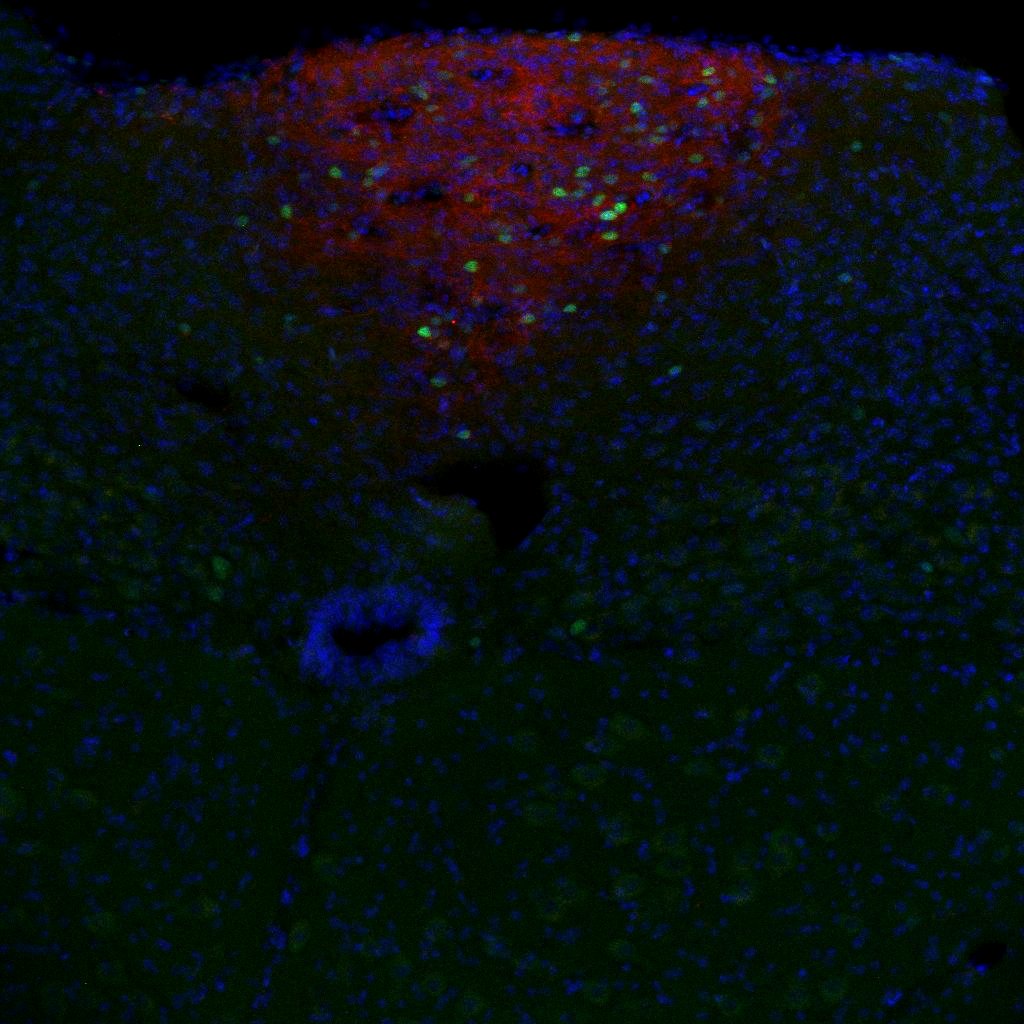

Supplement: Supplementary file 3 — Original pictures of cFos and Cy5 drug appearance shown in Fig. 3, including replicates used for quantification. [file 42255_2023_931_MOESM3_ESM.zip › Raw Data Figure 3/Figure 3D and E/Unadjusted/Fig3D_WT_GIPcy5_2.jpg]

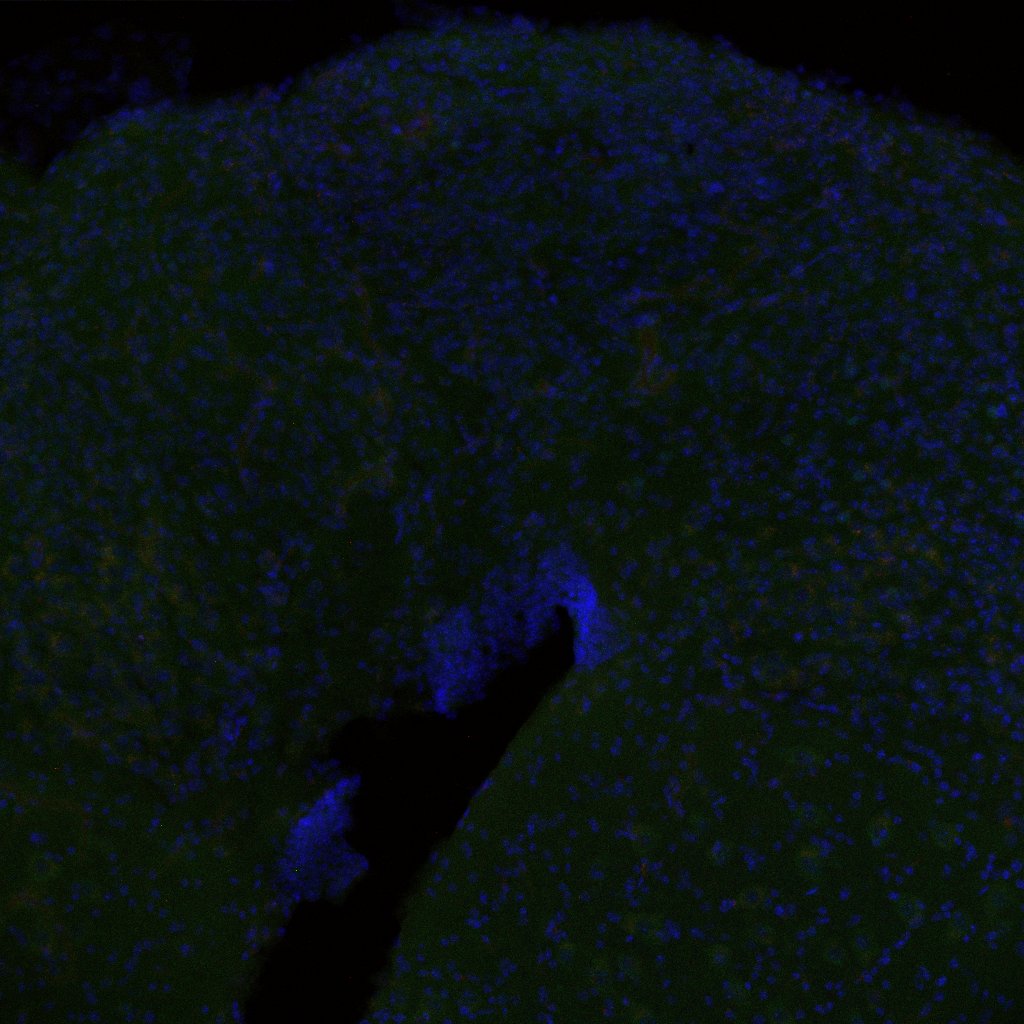

Supplement: Supplementary file 3 — Original pictures of cFos and Cy5 drug appearance shown in Fig. 3, including replicates used for quantification. [file 42255_2023_931_MOESM3_ESM.zip › Raw Data Figure 3/Figure 3D and E/Unadjusted/Fig3D_KO_Veh_4.jpg]

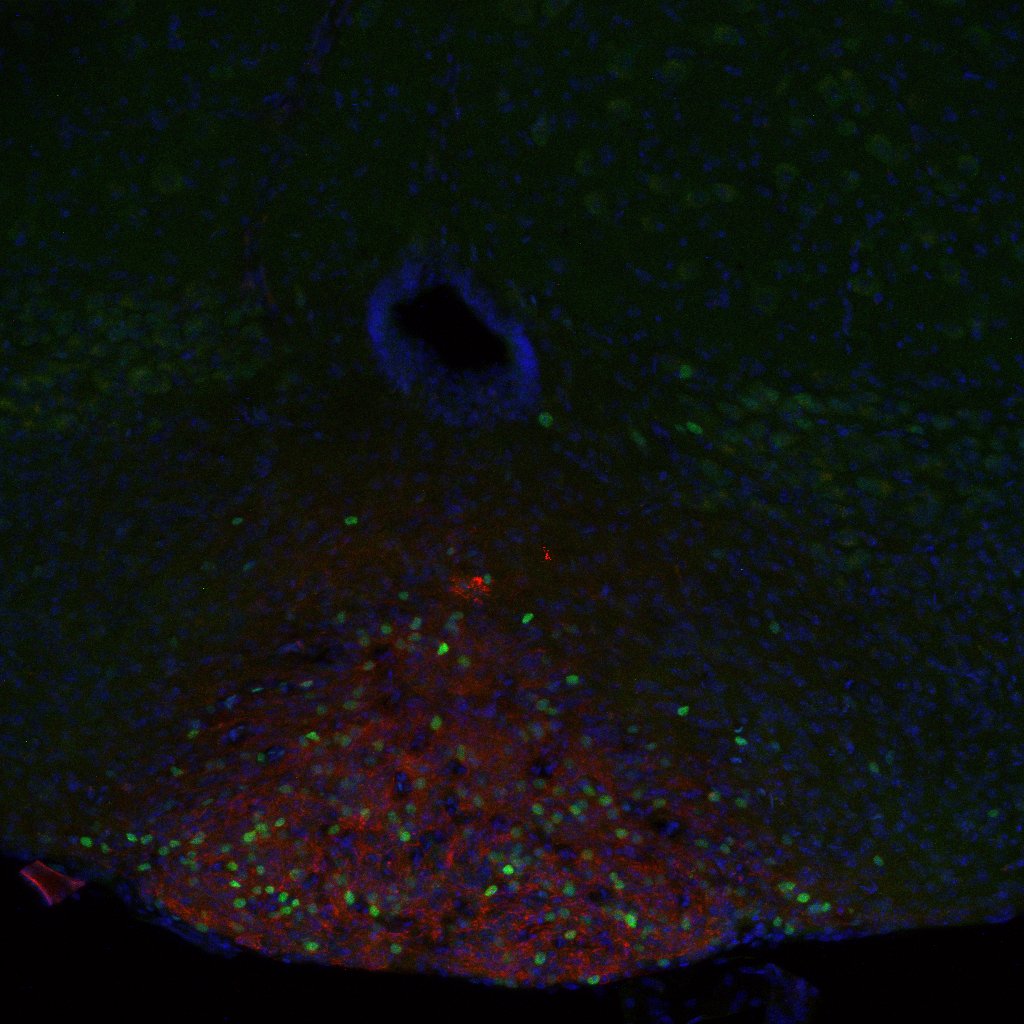

Supplement: Supplementary file 3 — Original pictures of cFos and Cy5 drug appearance shown in Fig. 3, including replicates used for quantification. [file 42255_2023_931_MOESM3_ESM.zip › Raw Data Figure 3/Figure 3D and E/Unadjusted/Fig3D_WT_GIPcy5_3.jpg]

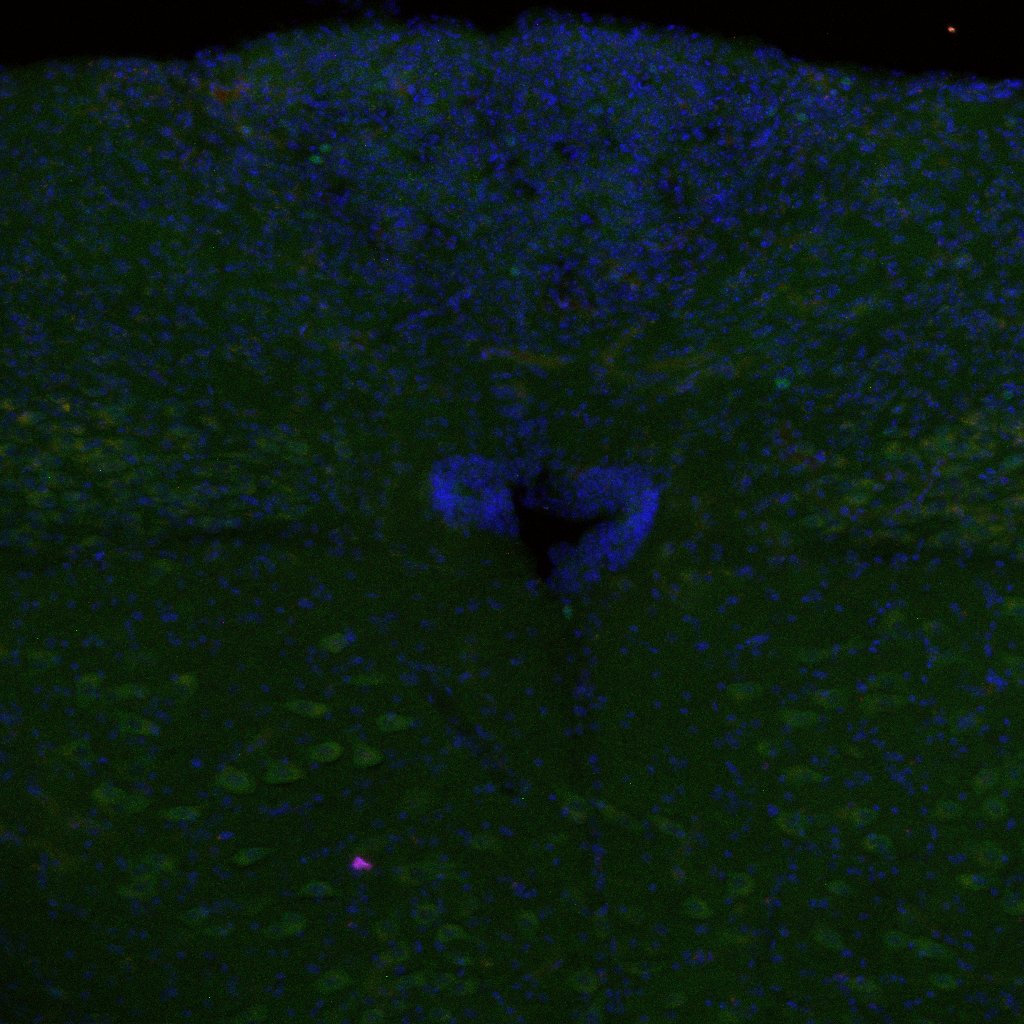

Supplement: Supplementary file 3 — Original pictures of cFos and Cy5 drug appearance shown in Fig. 3, including replicates used for quantification. [file 42255_2023_931_MOESM3_ESM.zip › Raw Data Figure 3/Figure 3D and E/Unadjusted/Fig3D_KO_Veh_1.jpg]

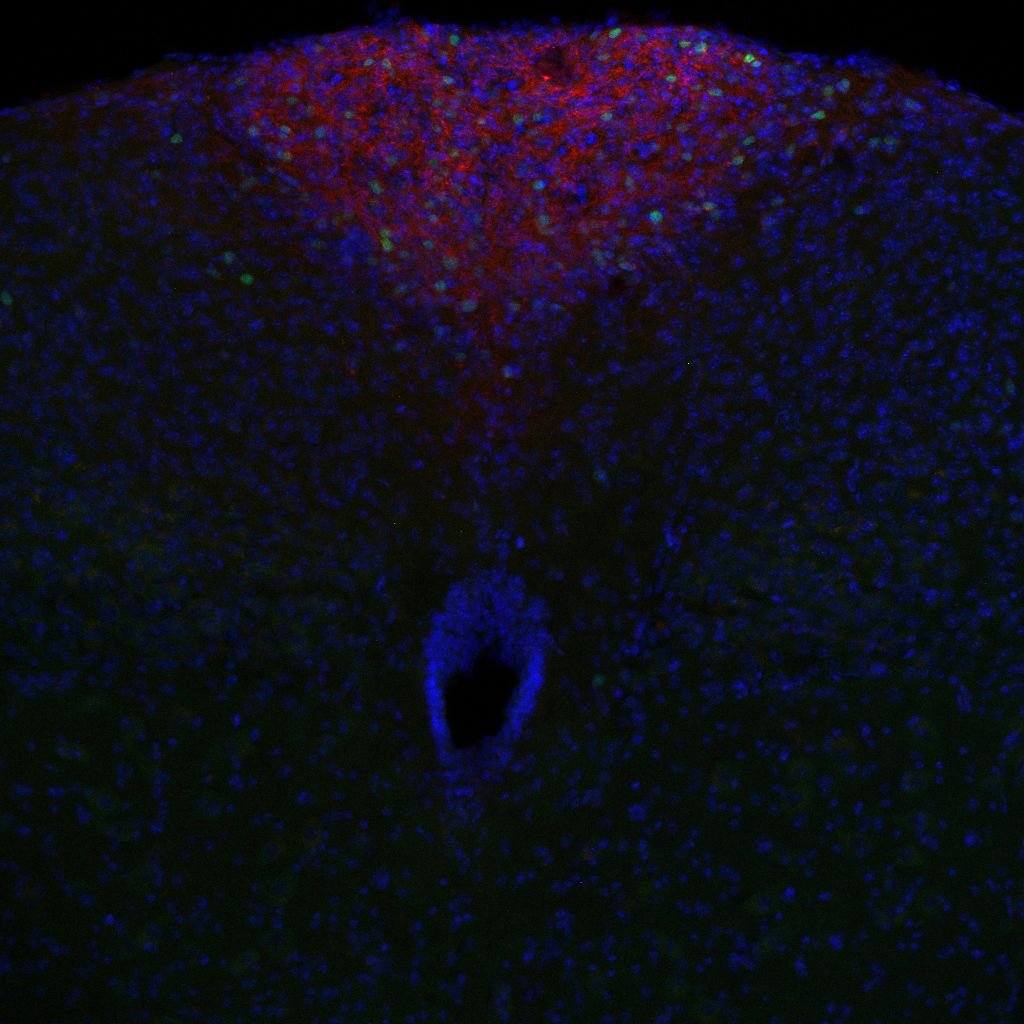

Supplement: Supplementary file 3 — Original pictures of cFos and Cy5 drug appearance shown in Fig. 3, including replicates used for quantification. [file 42255_2023_931_MOESM3_ESM.zip › Raw Data Figure 3/Figure 3D and E/Unadjusted/Fig3D_WT_GIPcy5_4.jpg]

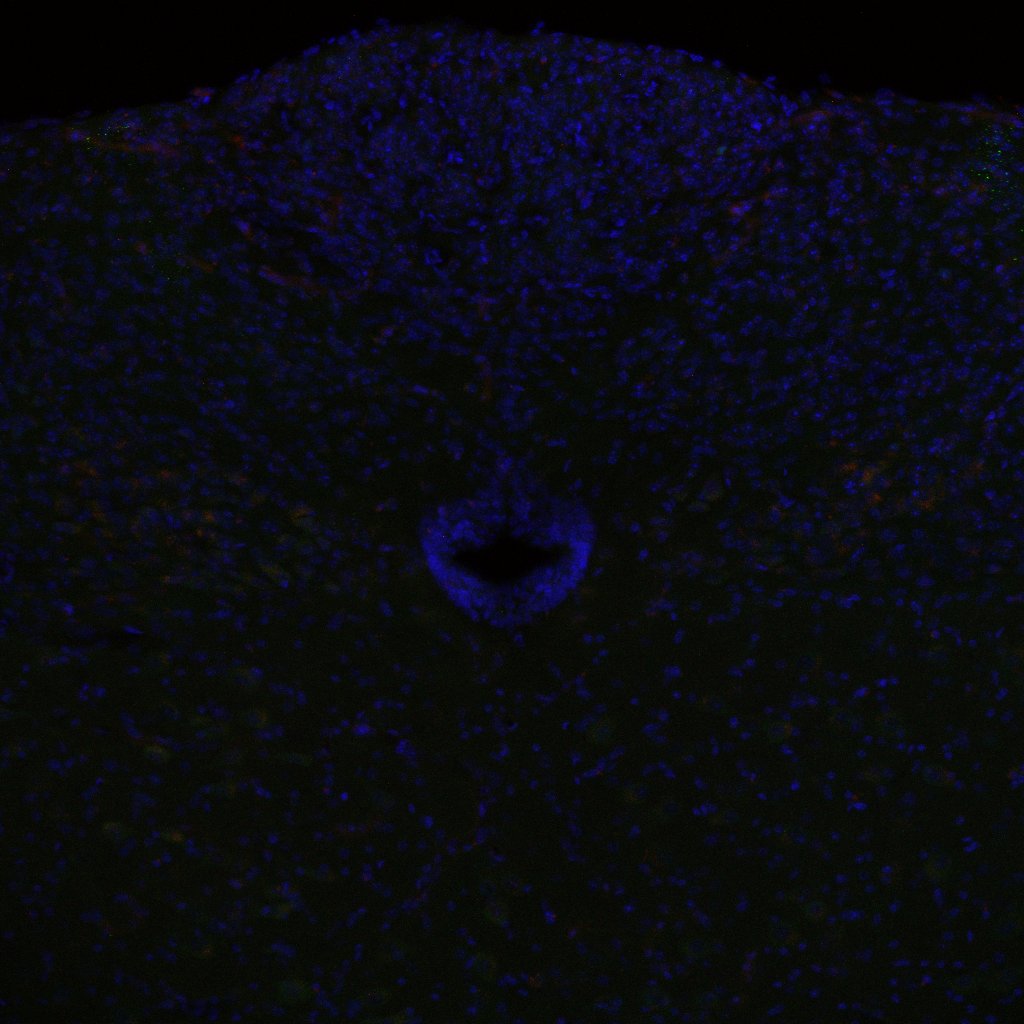

Supplement: Supplementary file 3 — Original pictures of cFos and Cy5 drug appearance shown in Fig. 3, including replicates used for quantification. [file 42255_2023_931_MOESM3_ESM.zip › Raw Data Figure 3/Figure 3D and E/Unadjusted/Fig3D_KO_Veh_3.jpg]

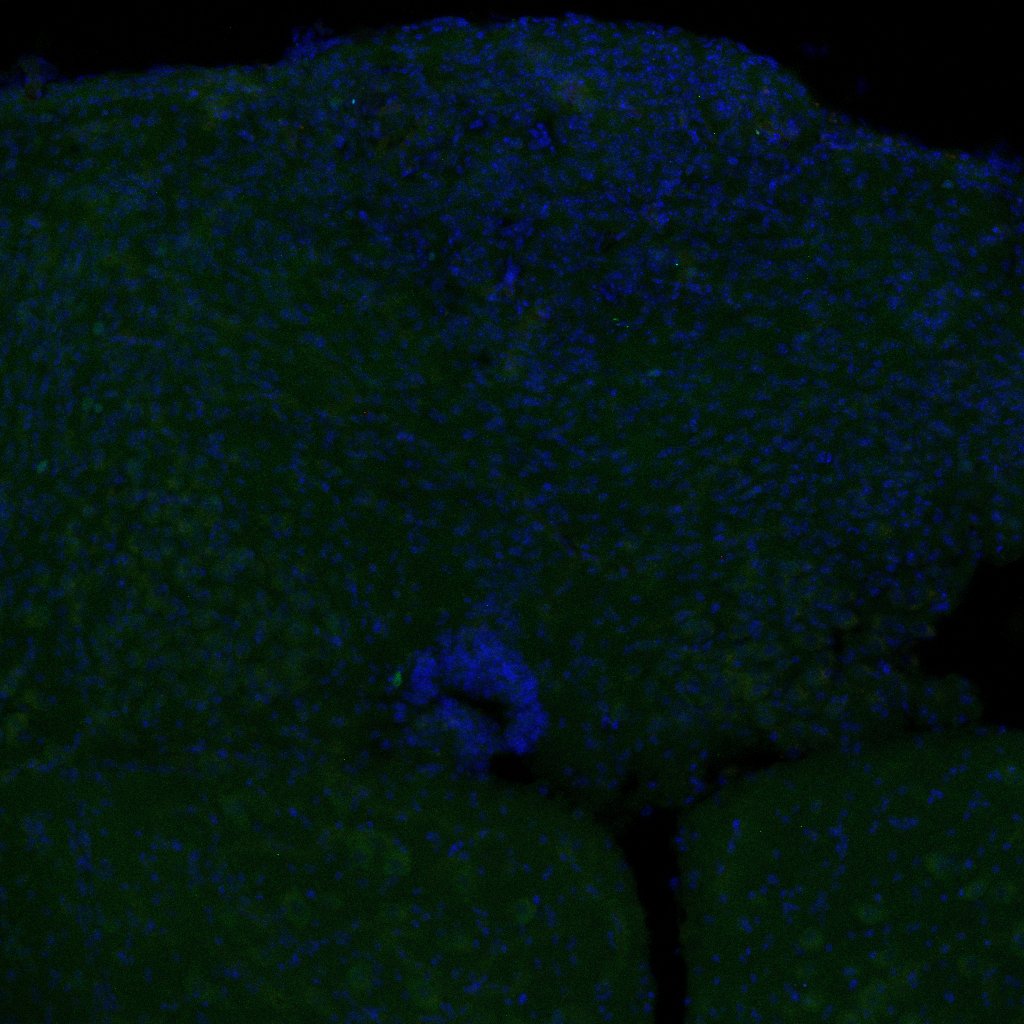

Supplement: Supplementary file 3 — Original pictures of cFos and Cy5 drug appearance shown in Fig. 3, including replicates used for quantification. [file 42255_2023_931_MOESM3_ESM.zip › Raw Data Figure 3/Figure 3D and E/Unadjusted/Fig3D_KO_Veh_2.jpg]

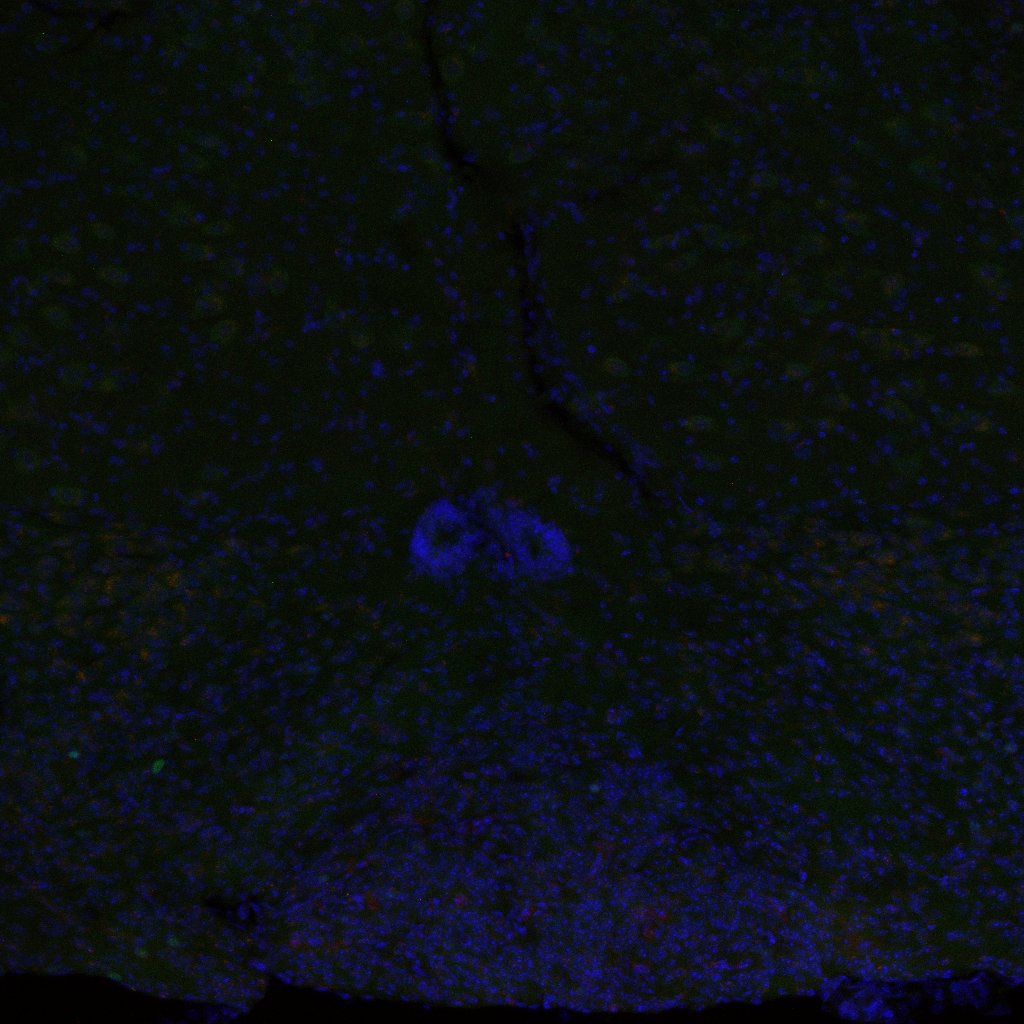

Supplement: Supplementary file 3 — Original pictures of cFos and Cy5 drug appearance shown in Fig. 3, including replicates used for quantification. [file 42255_2023_931_MOESM3_ESM.zip › Raw Data Figure 3/Figure 3D and E/Unadjusted/Fig3D_WT_Veh_4.jpg]

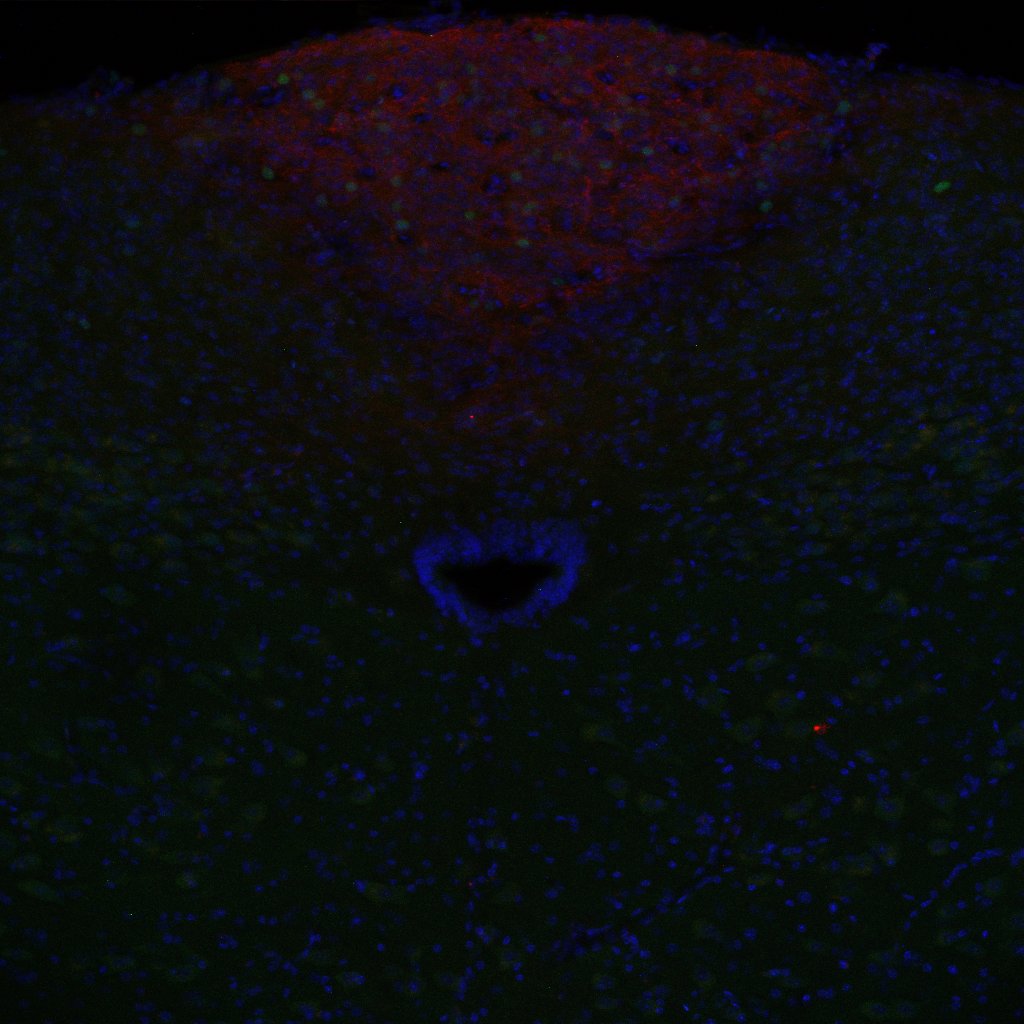

Supplement: Supplementary file 3 — Original pictures of cFos and Cy5 drug appearance shown in Fig. 3, including replicates used for quantification. [file 42255_2023_931_MOESM3_ESM.zip › Raw Data Figure 3/Figure 3D and E/Unadjusted/Fig3D_KO_GIPcy5_1.jpg]

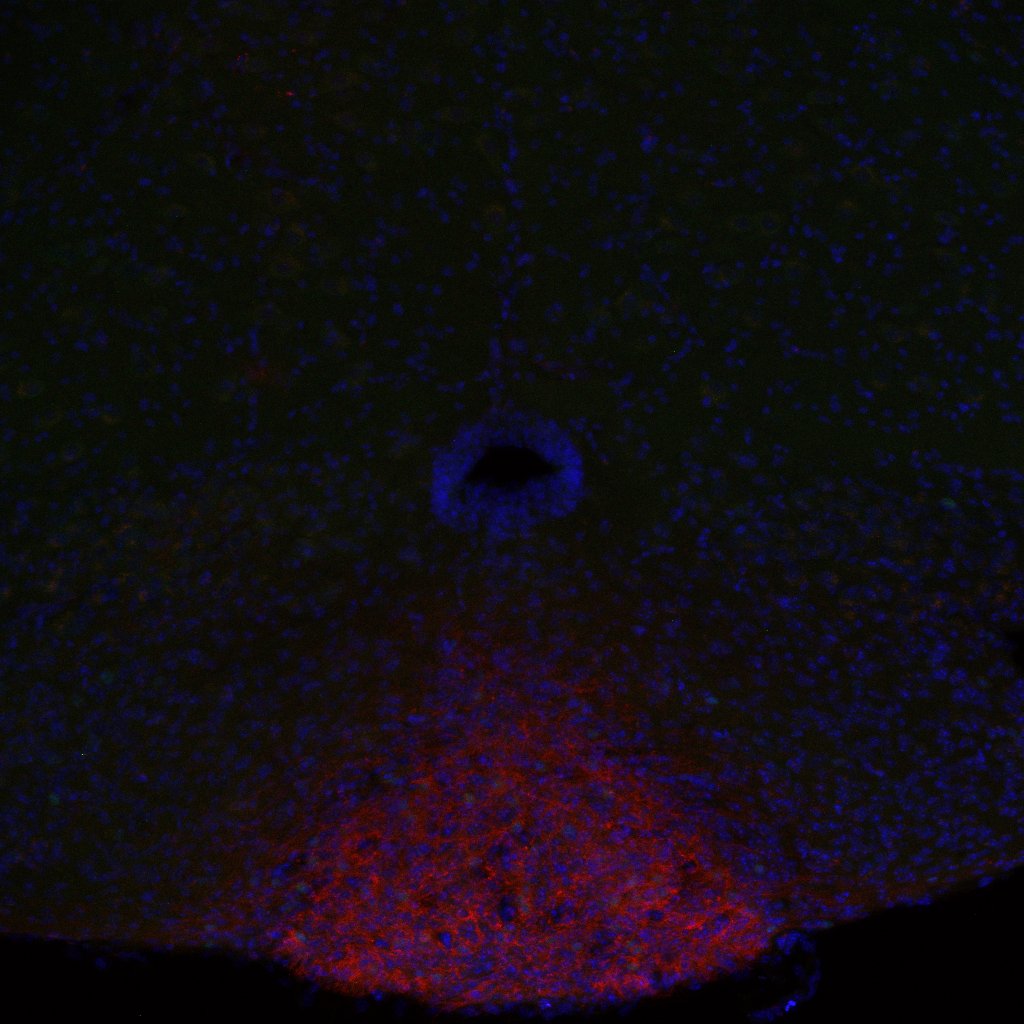

Supplement: Supplementary file 3 — Original pictures of cFos and Cy5 drug appearance shown in Fig. 3, including replicates used for quantification. [file 42255_2023_931_MOESM3_ESM.zip › Raw Data Figure 3/Figure 3D and E/Unadjusted/Fig3D_KO_GIPcy5_3.jpg]

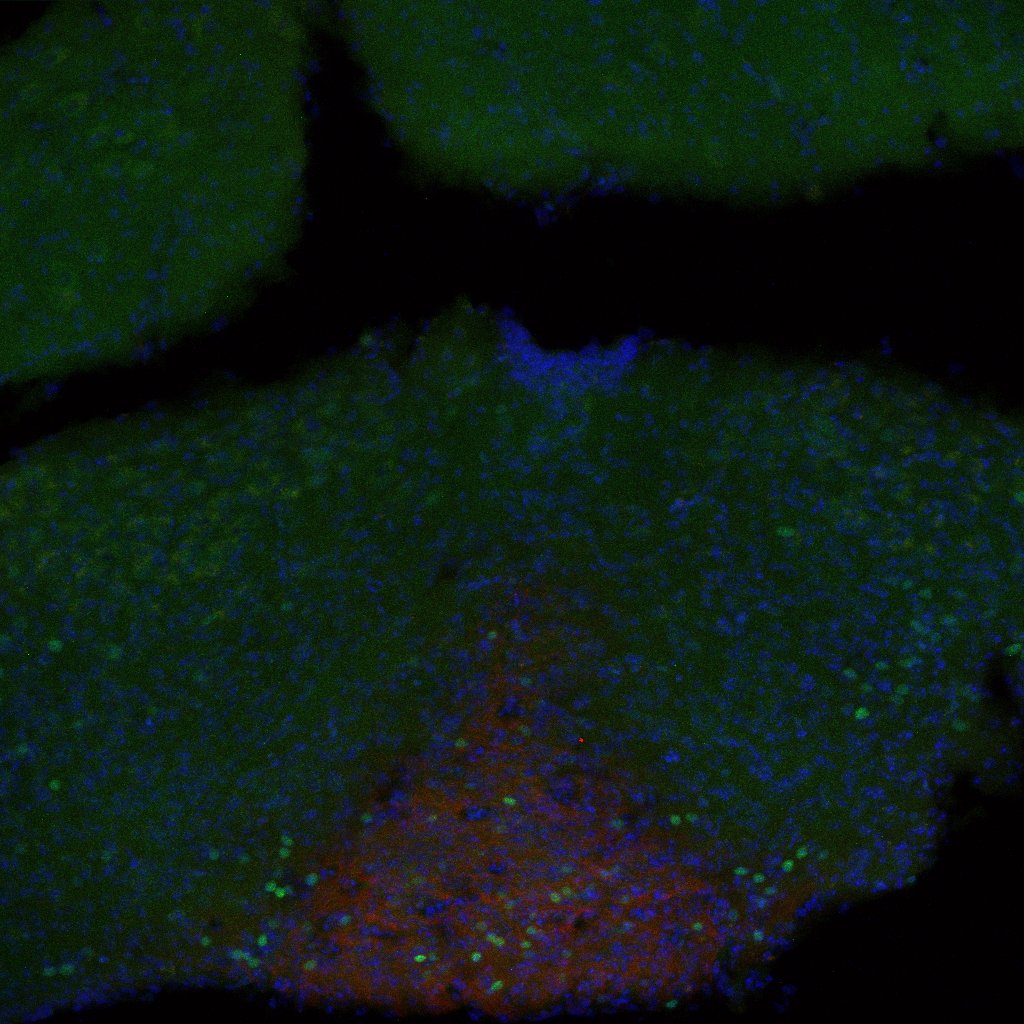

Supplement: Supplementary file 3 — Original pictures of cFos and Cy5 drug appearance shown in Fig. 3, including replicates used for quantification. [file 42255_2023_931_MOESM3_ESM.zip › Raw Data Figure 3/Figure 3D and E/Unadjusted/Fig3D_KO_GIPcy5_2.jpg]

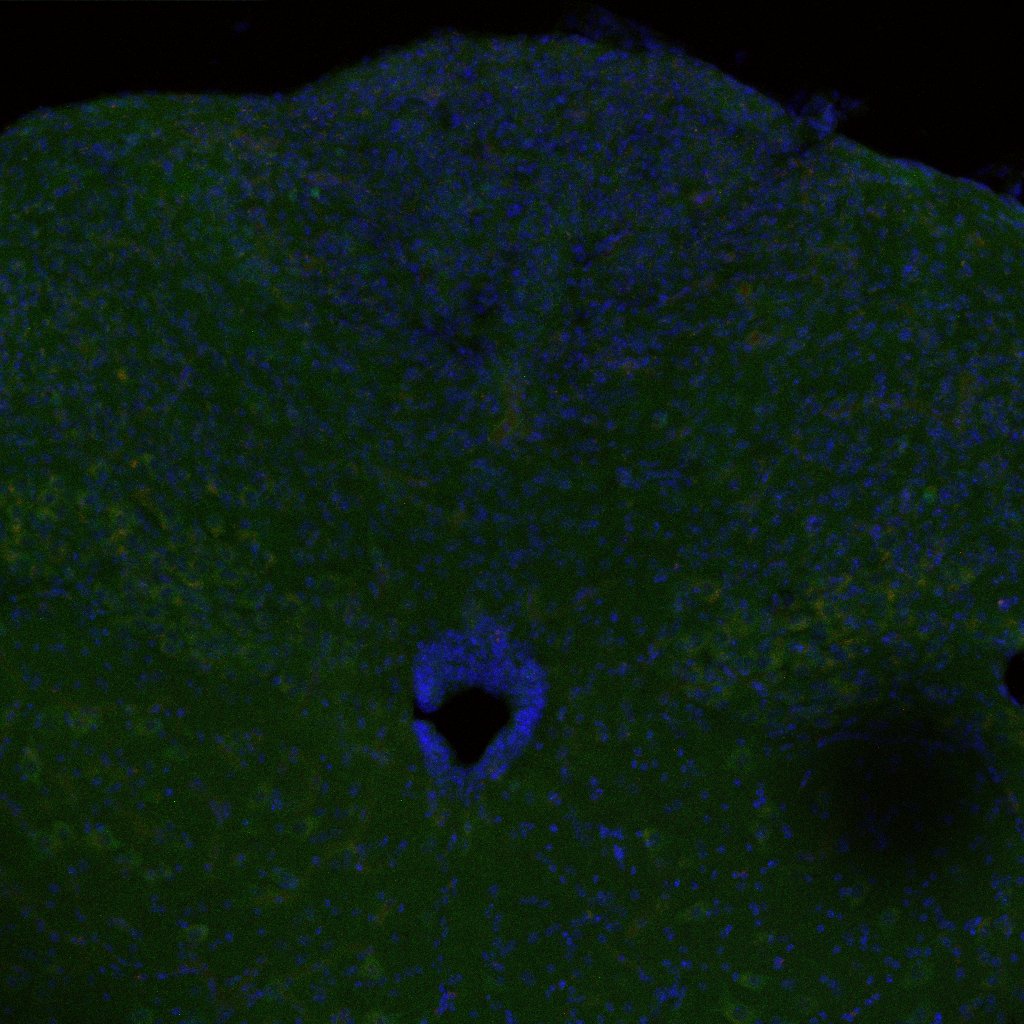

Supplement: Supplementary file 3 — Original pictures of cFos and Cy5 drug appearance shown in Fig. 3, including replicates used for quantification. [file 42255_2023_931_MOESM3_ESM.zip › Raw Data Figure 3/Figure 3D and E/Unadjusted/Fig3D_WT_Veh_3.jpg]

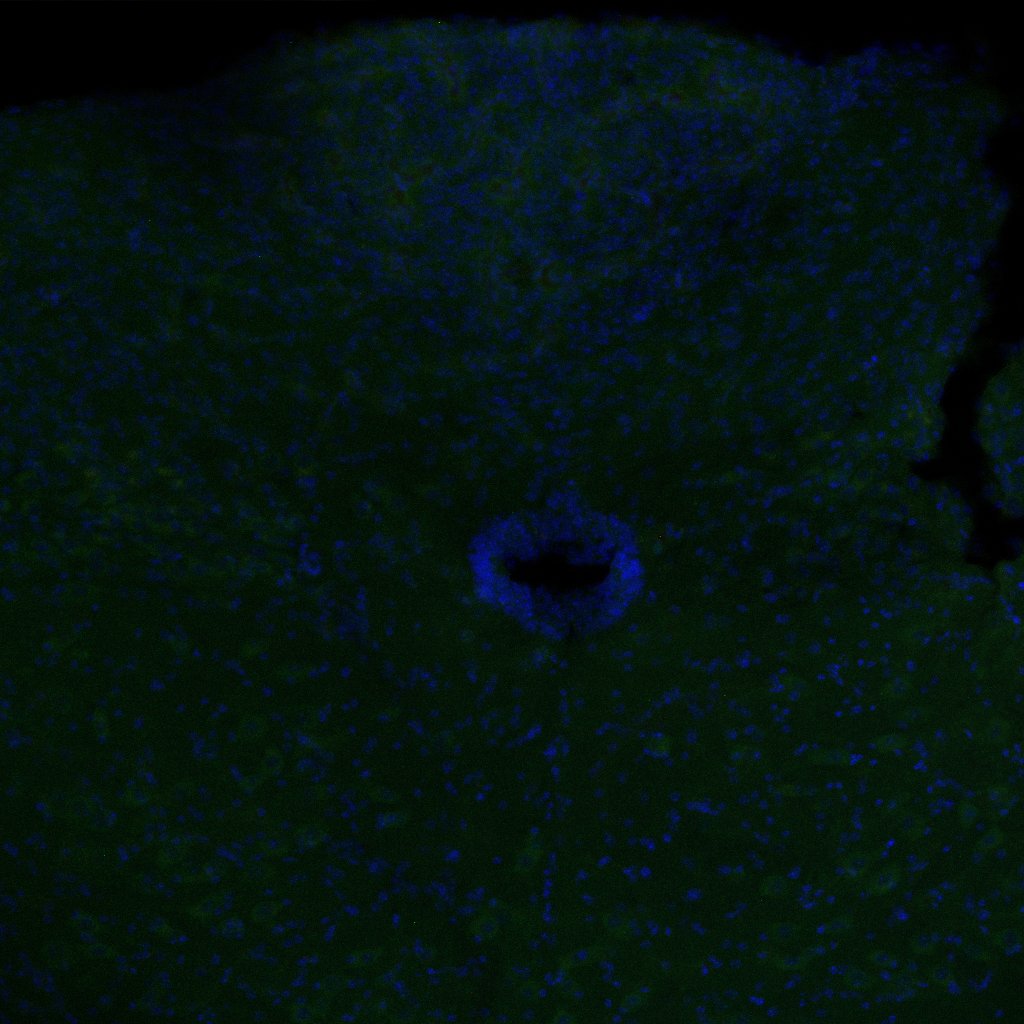

Supplement: Supplementary file 3 — Original pictures of cFos and Cy5 drug appearance shown in Fig. 3, including replicates used for quantification. [file 42255_2023_931_MOESM3_ESM.zip › Raw Data Figure 3/Figure 3D and E/Unadjusted/Fig3D_WT_Veh_2.jpg]

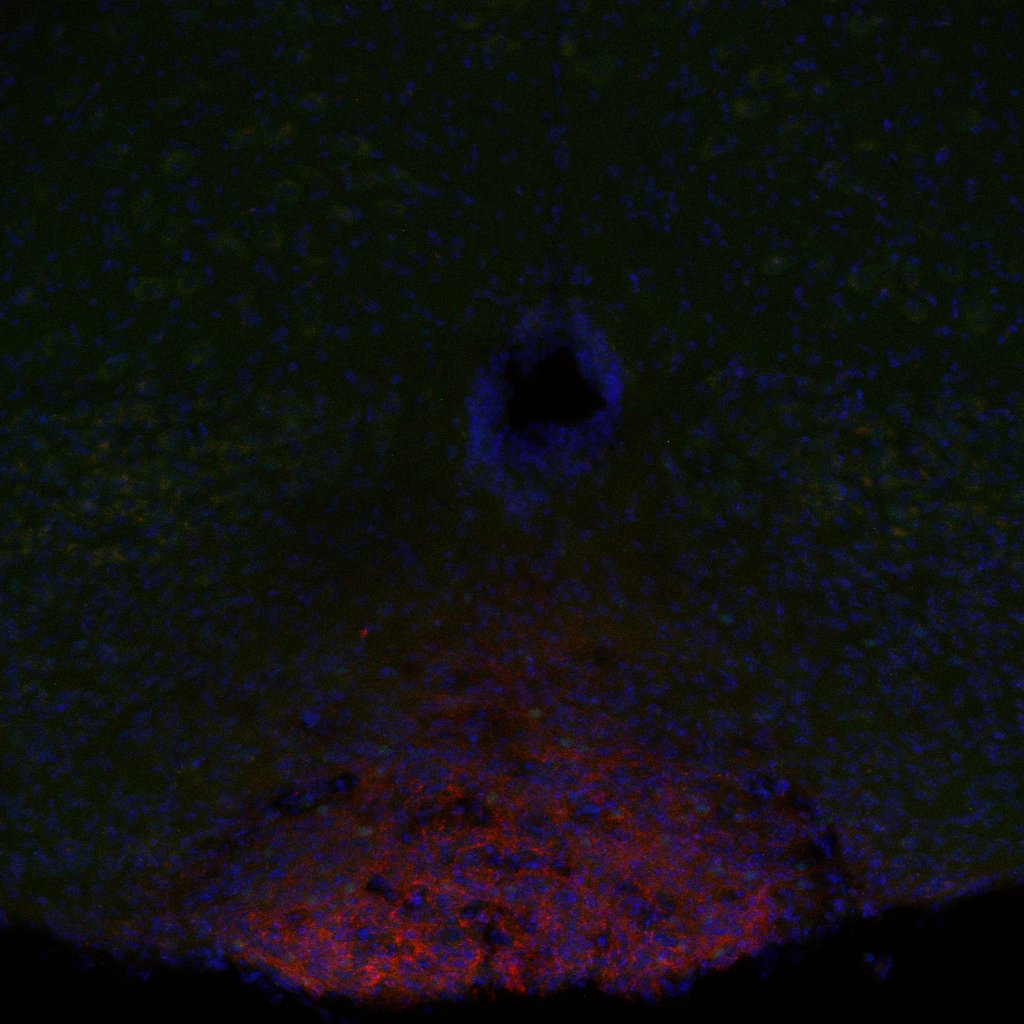

Supplement: Supplementary file 3 — Original pictures of cFos and Cy5 drug appearance shown in Fig. 3, including replicates used for quantification. [file 42255_2023_931_MOESM3_ESM.zip › Raw Data Figure 3/Figure 3D and E/Unadjusted/Fig3D_KO_GIPcy5_5.jpg]

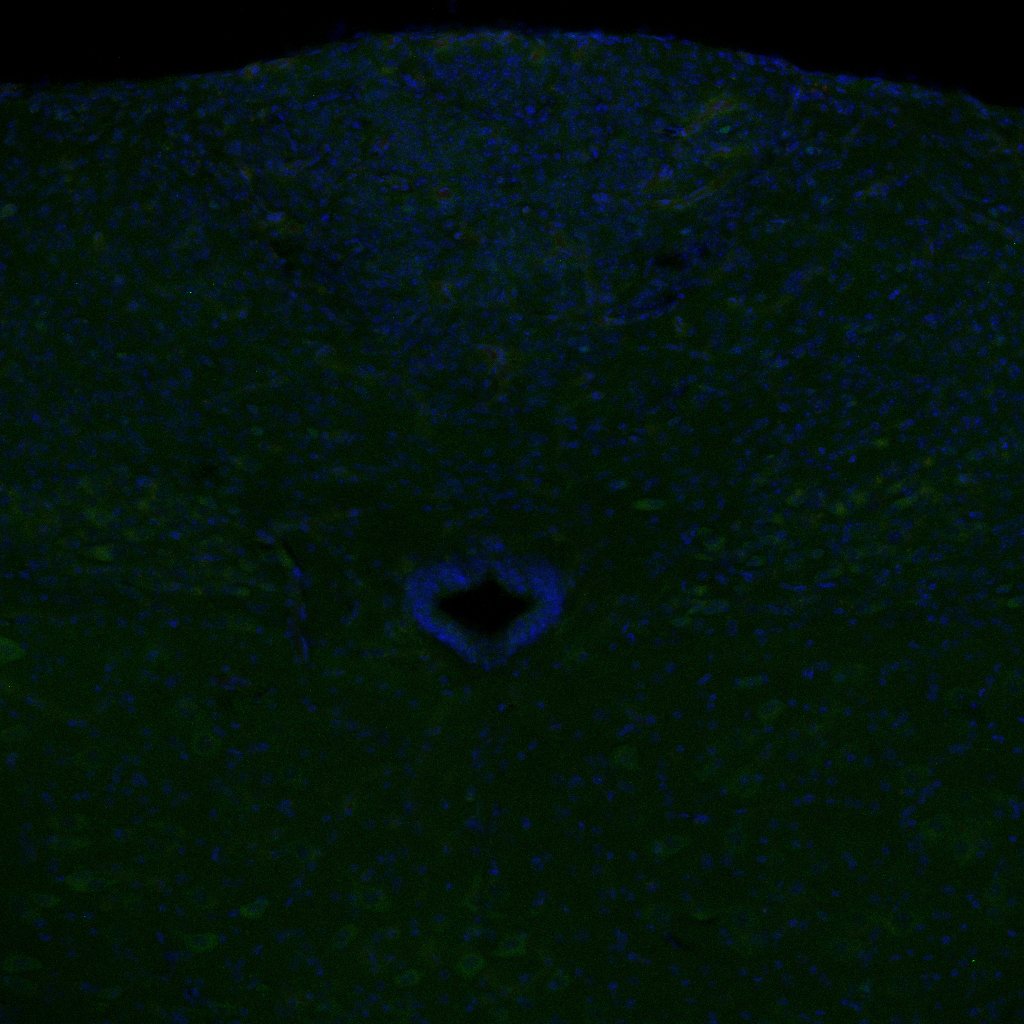

Supplement: Supplementary file 3 — Original pictures of cFos and Cy5 drug appearance shown in Fig. 3, including replicates used for quantification. [file 42255_2023_931_MOESM3_ESM.zip › Raw Data Figure 3/Figure 3D and E/Unadjusted/Fig3D_WT_Veh_1.jpg]

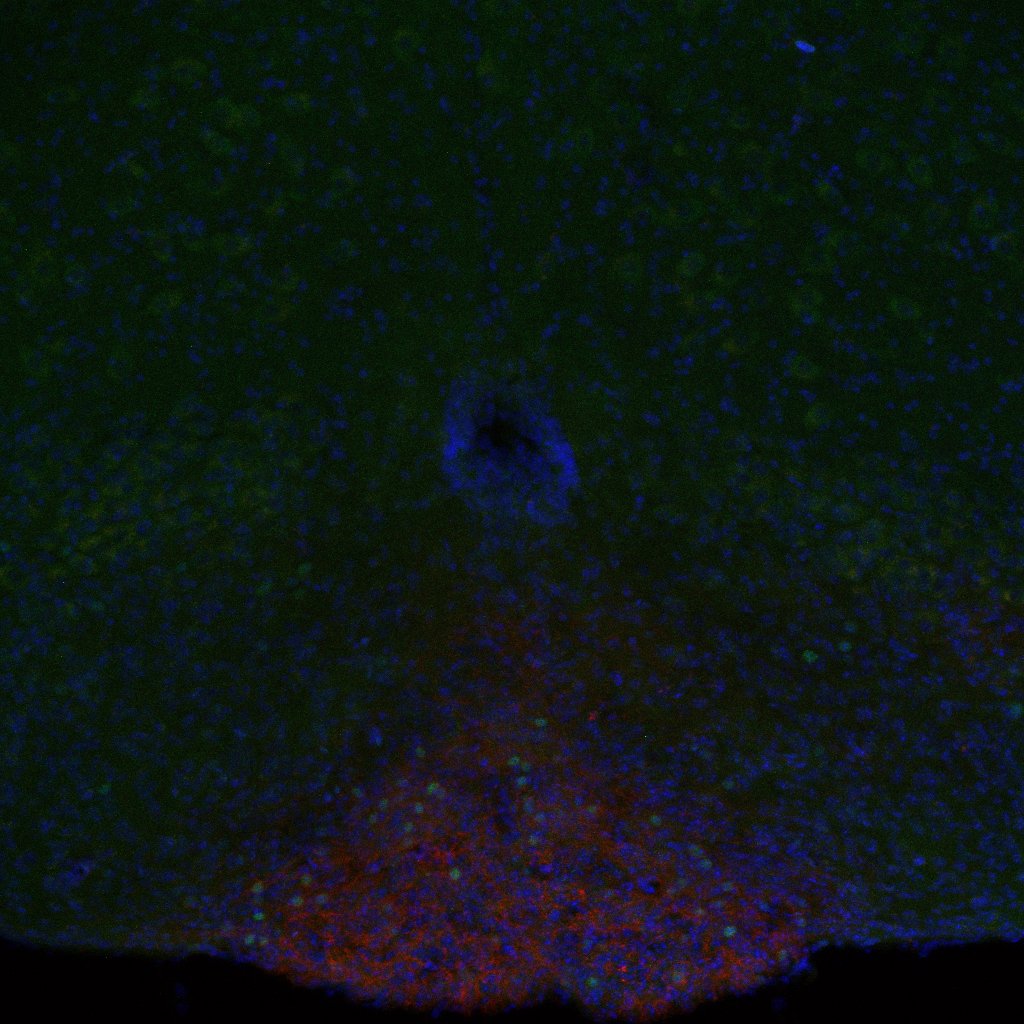

Supplement: Supplementary file 3 — Original pictures of cFos and Cy5 drug appearance shown in Fig. 3, including replicates used for quantification. [file 42255_2023_931_MOESM3_ESM.zip › Raw Data Figure 3/Figure 3D and E/Unadjusted/Fig3D_KO_GIPcy5_4.jpg]
