# Supplementary figures and images for: Glucose-dependent insulinotropic polypeptide regulates body weight and food intake via GABAergic neurons in mice (part 1 of 2)
Source: Nat Metab. 2023 Nov 9;5(12):2075–85. doi: 10.1038/s42255-023-00931-7 (PMC10730394; doi:10.1038/s42255-023-00931-7)

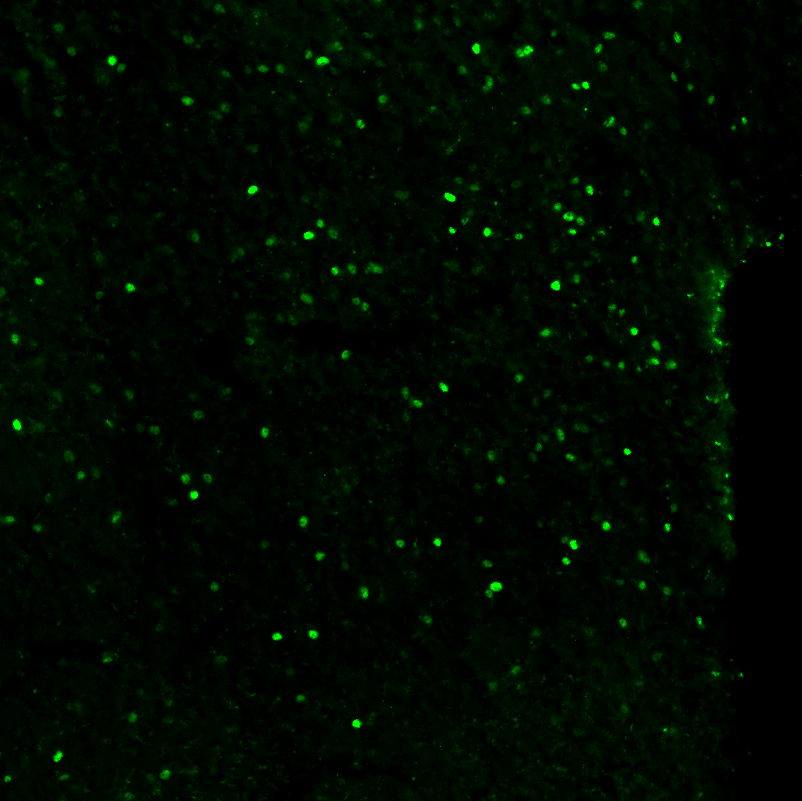

Supplement: Supplementary file 2 — Original pictures of cFos and Cy5 drug appearance shown in Extended Data Fig. 5a–h, including replicates used for quantification. [file 42255_2023_931_MOESM2_ESM.zip › Raw Data Extended Data Figure 5/02-DMH/2-M-KO-GIP-HYPO.tif]

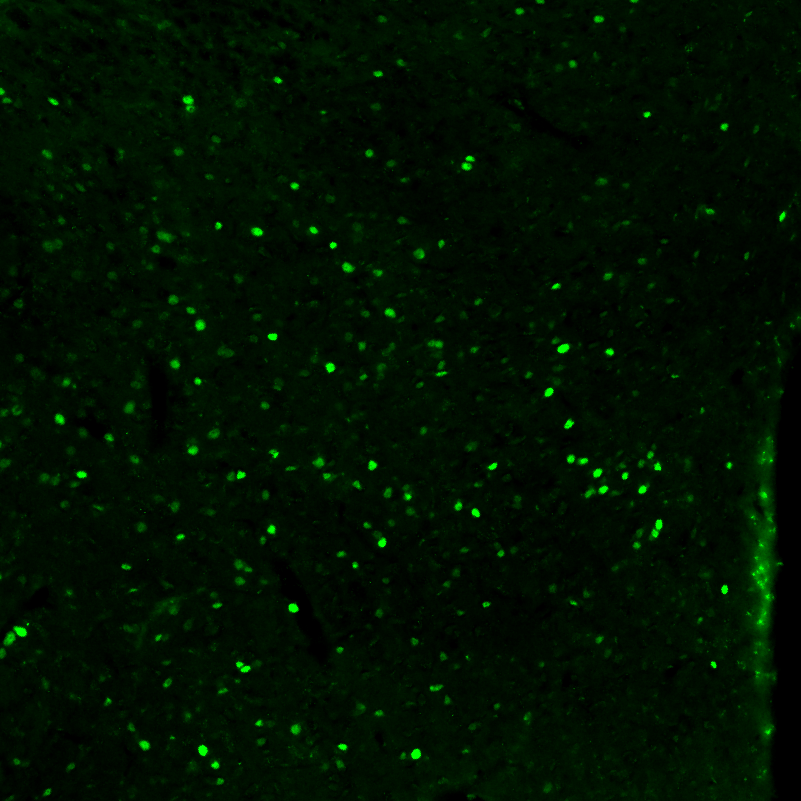

Supplement: Supplementary file 2 — Original pictures of cFos and Cy5 drug appearance shown in Extended Data Fig. 5a–h, including replicates used for quantification. [file 42255_2023_931_MOESM2_ESM.zip › Raw Data Extended Data Figure 5/02-DMH/46-M-WT-GIP-HYPO.tif]

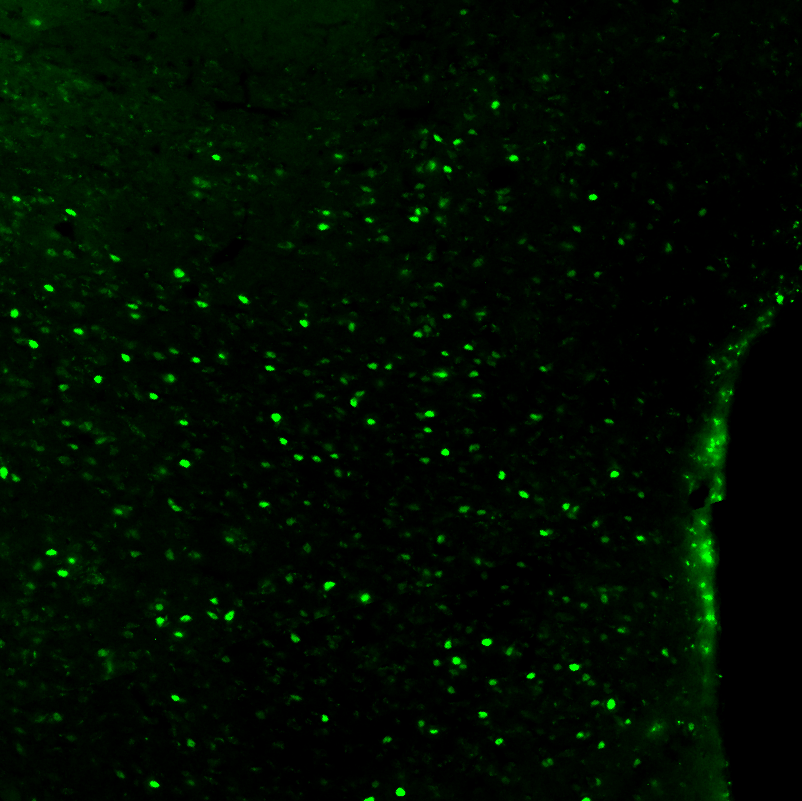

Supplement: Supplementary file 2 — Original pictures of cFos and Cy5 drug appearance shown in Extended Data Fig. 5a–h, including replicates used for quantification. [file 42255_2023_931_MOESM2_ESM.zip › Raw Data Extended Data Figure 5/02-DMH/3-M-KO-GIP-HYPO.tif]

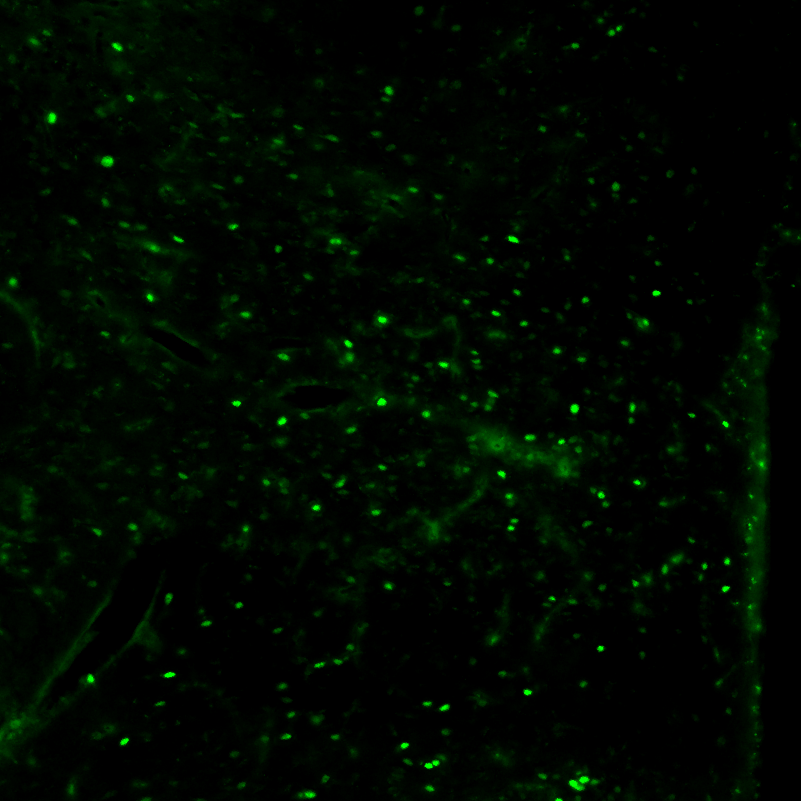

Supplement: Supplementary file 2 — Original pictures of cFos and Cy5 drug appearance shown in Extended Data Fig. 5a–h, including replicates used for quantification. [file 42255_2023_931_MOESM2_ESM.zip › Raw Data Extended Data Figure 5/02-DMH/36-M-KO-Veh-HYPO.tif]

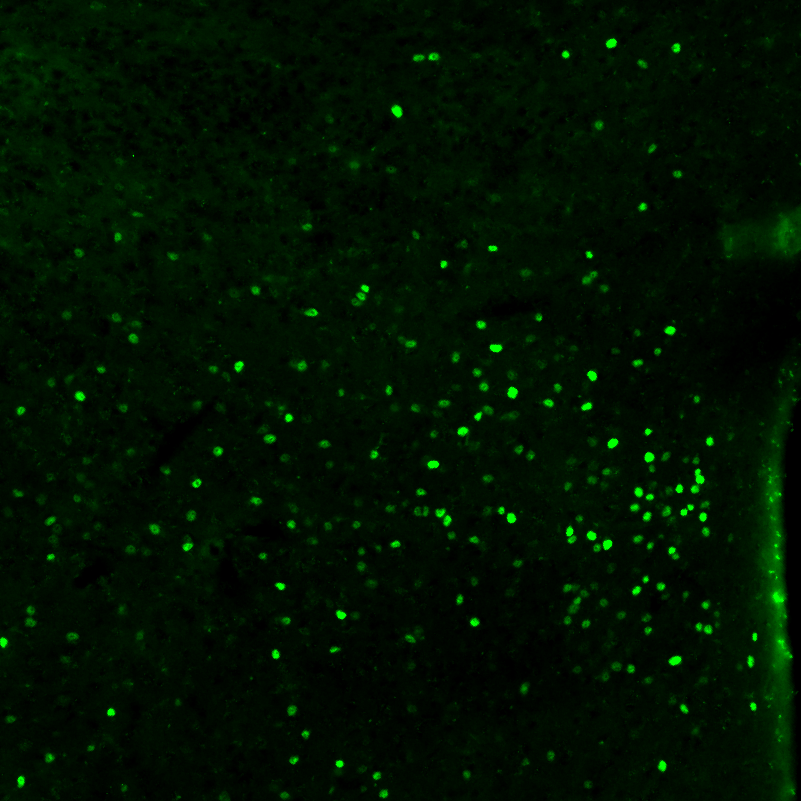

Supplement: Supplementary file 2 — Original pictures of cFos and Cy5 drug appearance shown in Extended Data Fig. 5a–h, including replicates used for quantification. [file 42255_2023_931_MOESM2_ESM.zip › Raw Data Extended Data Figure 5/02-DMH/44-M-WT-GIP-HYPO.tif]

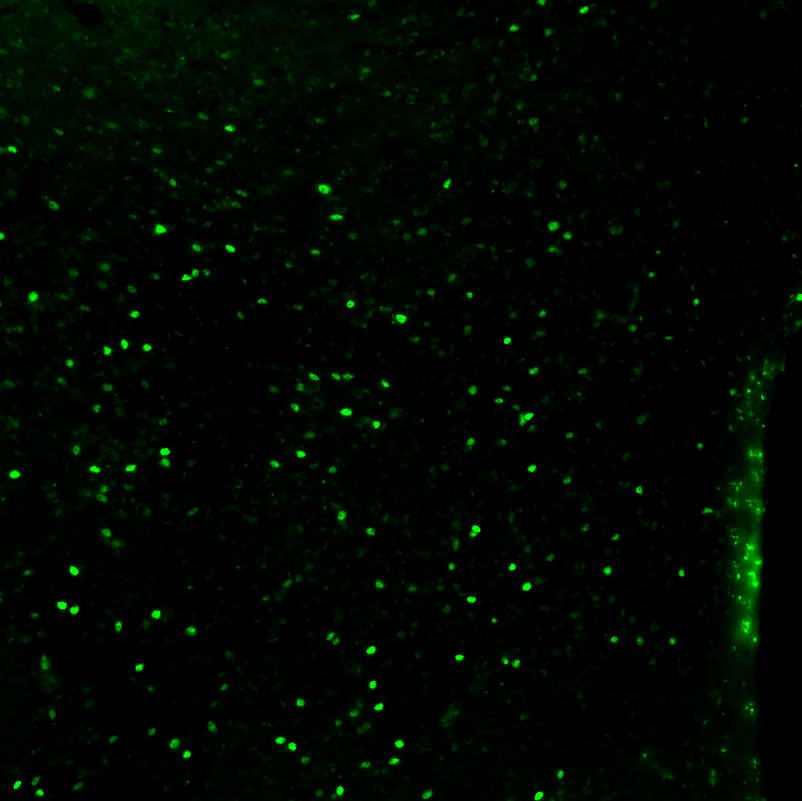

Supplement: Supplementary file 2 — Original pictures of cFos and Cy5 drug appearance shown in Extended Data Fig. 5a–h, including replicates used for quantification. [file 42255_2023_931_MOESM2_ESM.zip › Raw Data Extended Data Figure 5/02-DMH/1-M-KO-GIP-HYPO.tif]

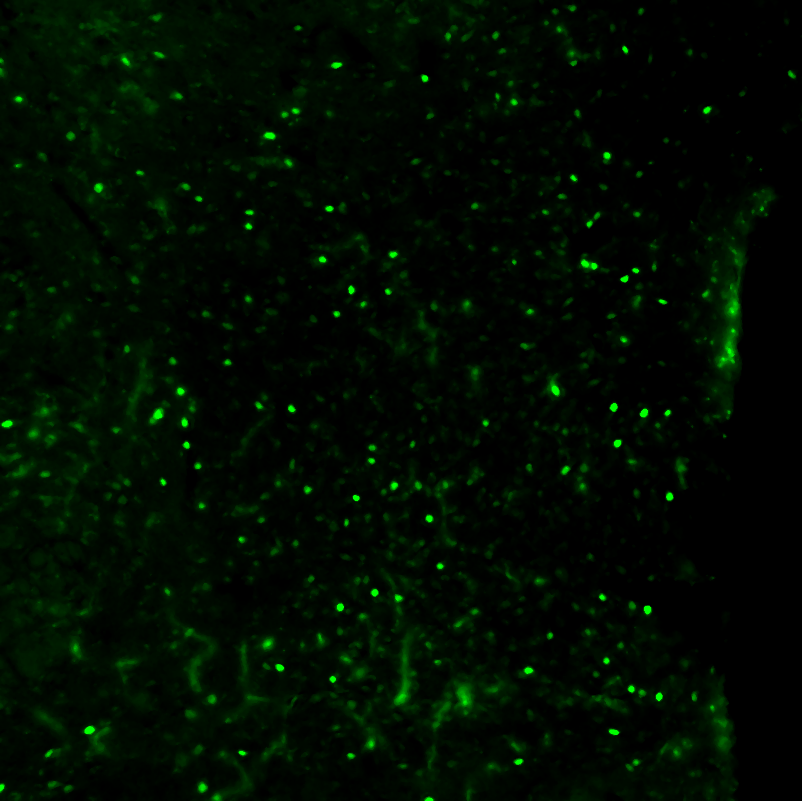

Supplement: Supplementary file 2 — Original pictures of cFos and Cy5 drug appearance shown in Extended Data Fig. 5a–h, including replicates used for quantification. [file 42255_2023_931_MOESM2_ESM.zip › Raw Data Extended Data Figure 5/02-DMH/37-M-KO-Veh-HYPO.tif]

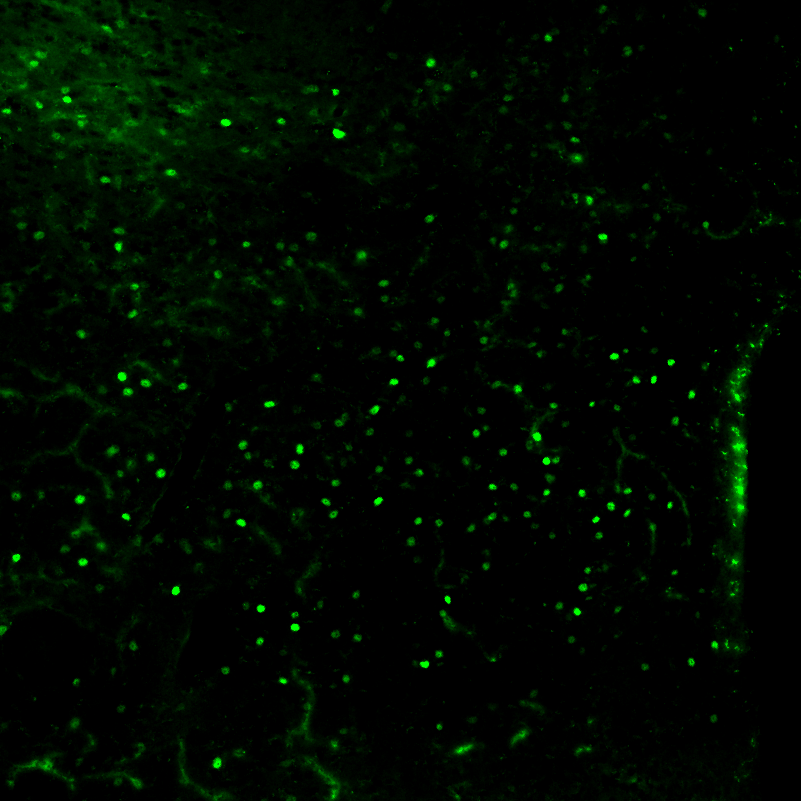

Supplement: Supplementary file 2 — Original pictures of cFos and Cy5 drug appearance shown in Extended Data Fig. 5a–h, including replicates used for quantification. [file 42255_2023_931_MOESM2_ESM.zip › Raw Data Extended Data Figure 5/02-DMH/66-M-WT-Veh-HYPO.tif]

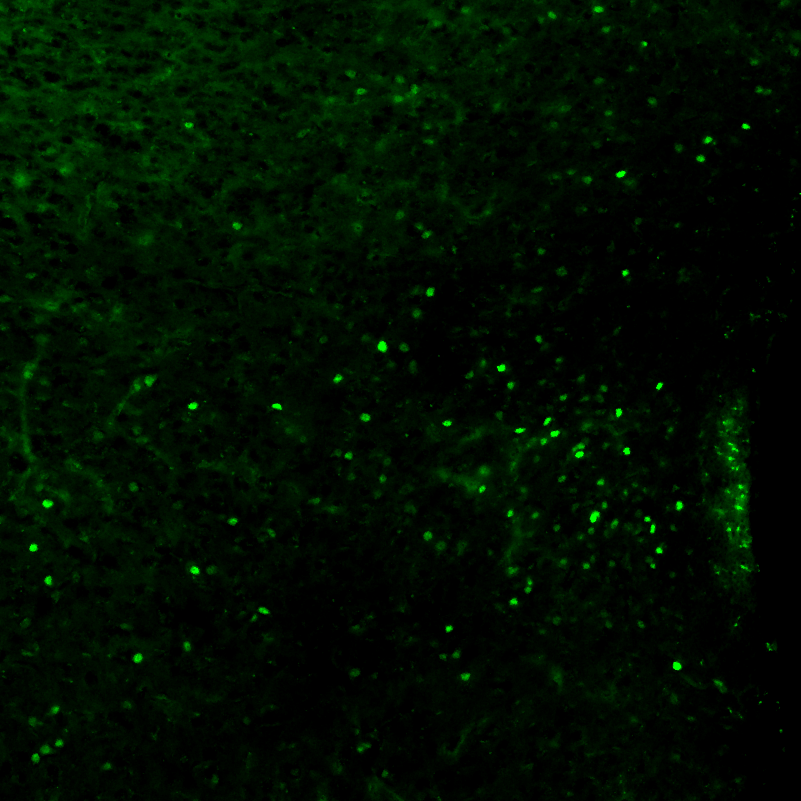

Supplement: Supplementary file 2 — Original pictures of cFos and Cy5 drug appearance shown in Extended Data Fig. 5a–h, including replicates used for quantification. [file 42255_2023_931_MOESM2_ESM.zip › Raw Data Extended Data Figure 5/02-DMH/55-M-WT-GIP-HYPO.tif]

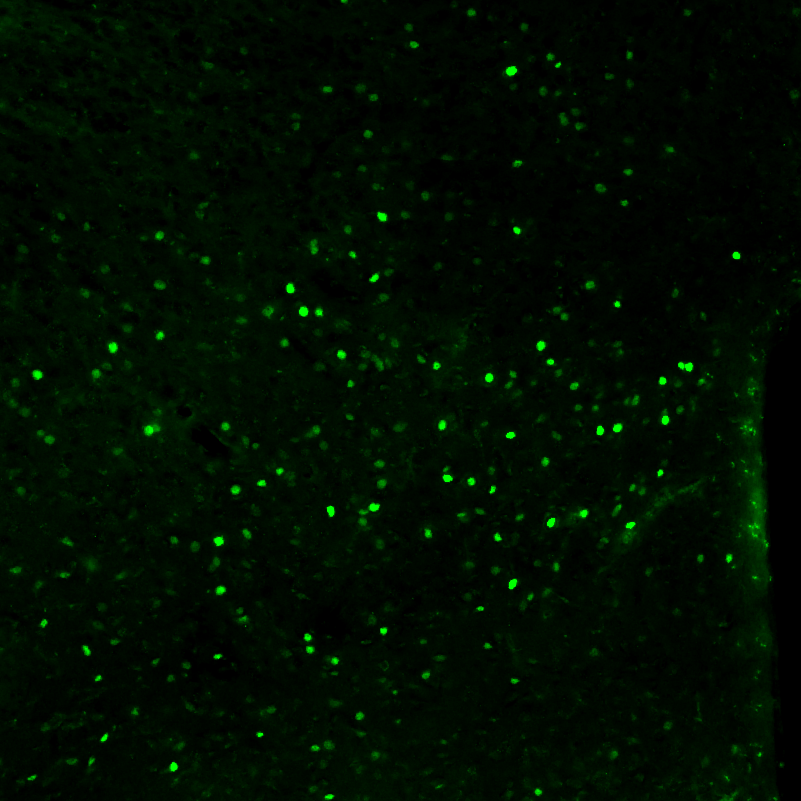

Supplement: Supplementary file 2 — Original pictures of cFos and Cy5 drug appearance shown in Extended Data Fig. 5a–h, including replicates used for quantification. [file 42255_2023_931_MOESM2_ESM.zip › Raw Data Extended Data Figure 5/02-DMH/14-M-KO-GIP-HYPO.tif]

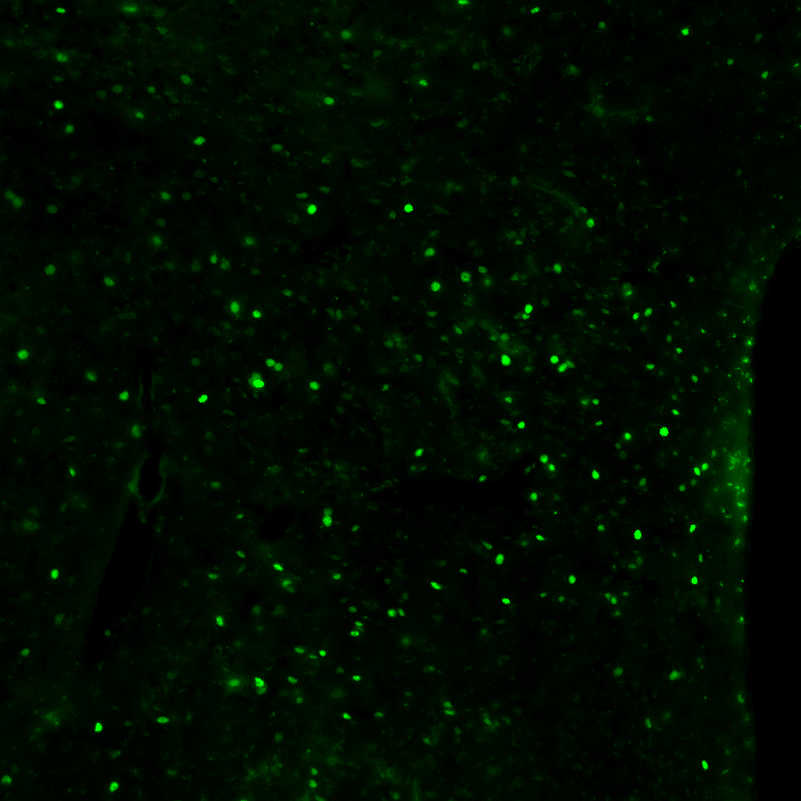

Supplement: Supplementary file 2 — Original pictures of cFos and Cy5 drug appearance shown in Extended Data Fig. 5a–h, including replicates used for quantification. [file 42255_2023_931_MOESM2_ESM.zip › Raw Data Extended Data Figure 5/02-DMH/17-M-KO-Veh-HYPO.tif]

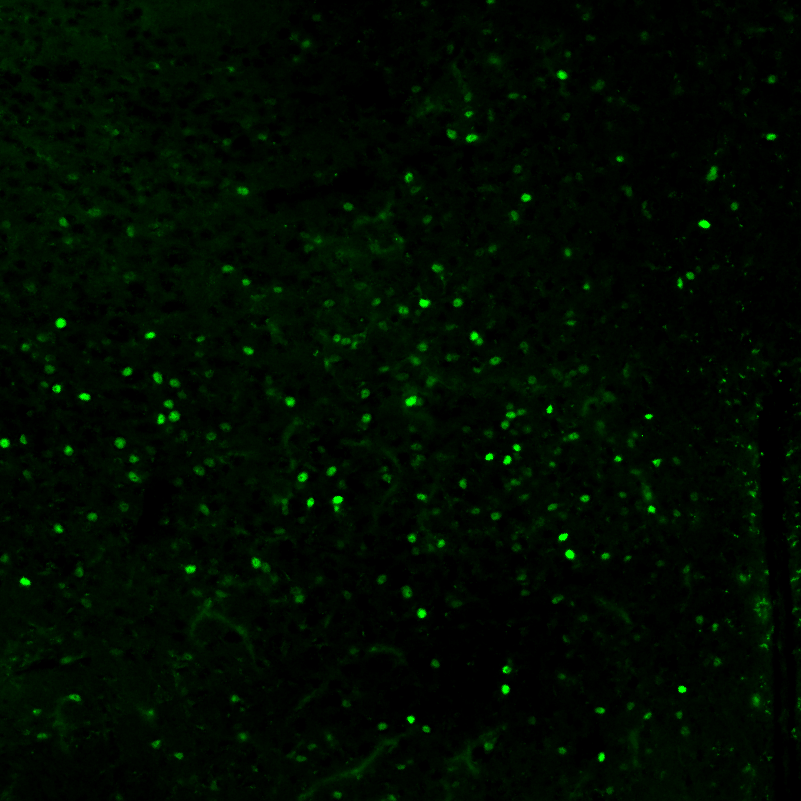

Supplement: Supplementary file 2 — Original pictures of cFos and Cy5 drug appearance shown in Extended Data Fig. 5a–h, including replicates used for quantification. [file 42255_2023_931_MOESM2_ESM.zip › Raw Data Extended Data Figure 5/02-DMH/67-M-WT-Veh-HYPO.tif]

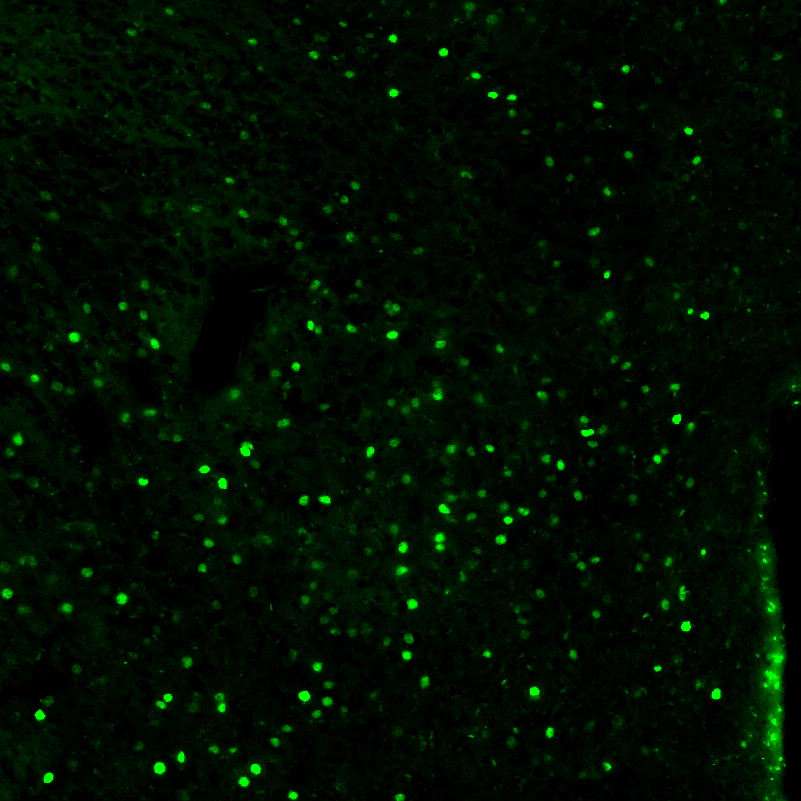

Supplement: Supplementary file 2 — Original pictures of cFos and Cy5 drug appearance shown in Extended Data Fig. 5a–h, including replicates used for quantification. [file 42255_2023_931_MOESM2_ESM.zip › Raw Data Extended Data Figure 5/02-DMH/54-M-WT-GIP-HYPO.tif]

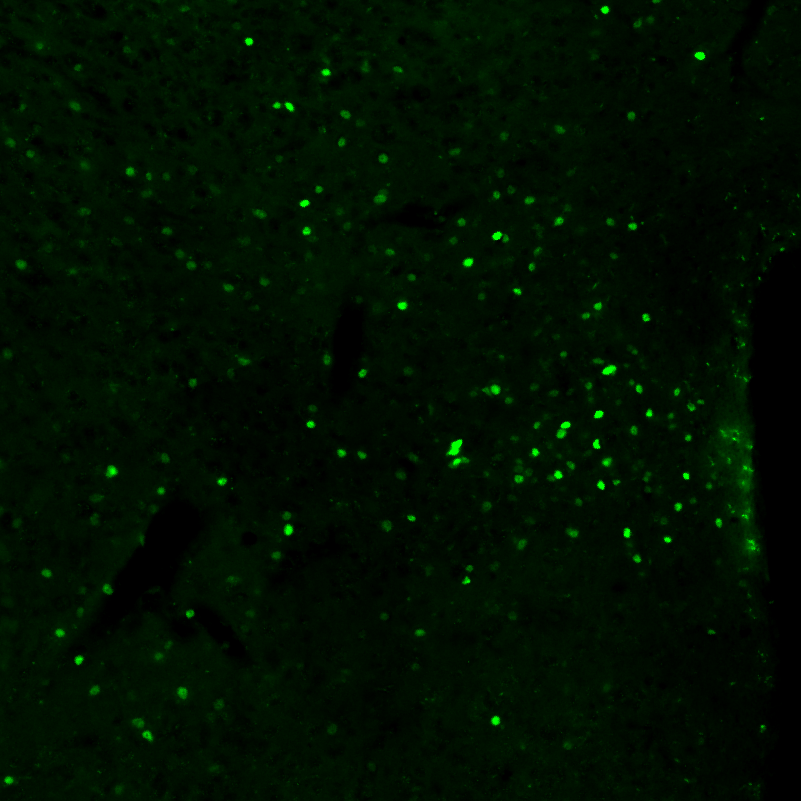

Supplement: Supplementary file 2 — Original pictures of cFos and Cy5 drug appearance shown in Extended Data Fig. 5a–h, including replicates used for quantification. [file 42255_2023_931_MOESM2_ESM.zip › Raw Data Extended Data Figure 5/02-DMH/15-M-KO-GIP-HYPO.tif]

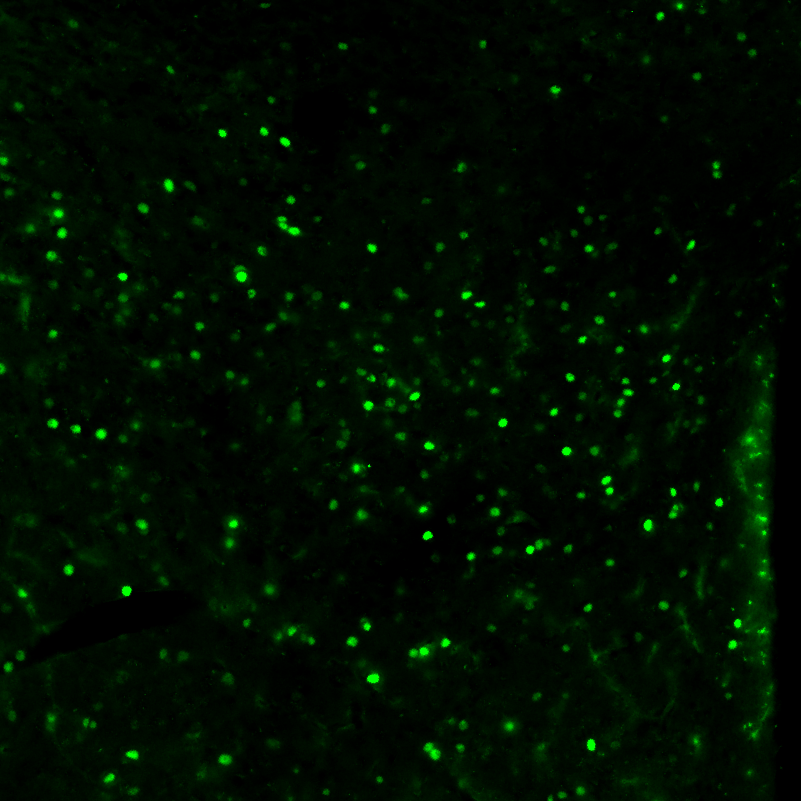

Supplement: Supplementary file 2 — Original pictures of cFos and Cy5 drug appearance shown in Extended Data Fig. 5a–h, including replicates used for quantification. [file 42255_2023_931_MOESM2_ESM.zip › Raw Data Extended Data Figure 5/02-DMH/20-M-KO-Veh-HYPO.tif]

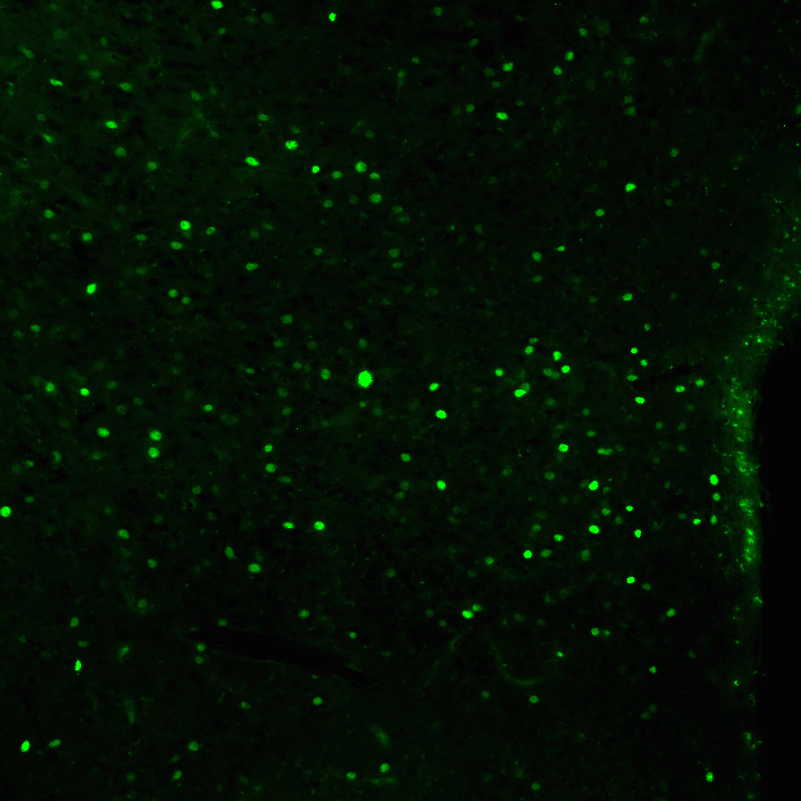

Supplement: Supplementary file 2 — Original pictures of cFos and Cy5 drug appearance shown in Extended Data Fig. 5a–h, including replicates used for quantification. [file 42255_2023_931_MOESM2_ESM.zip › Raw Data Extended Data Figure 5/02-DMH/64-M-WT-Veh-HYPO.tif]

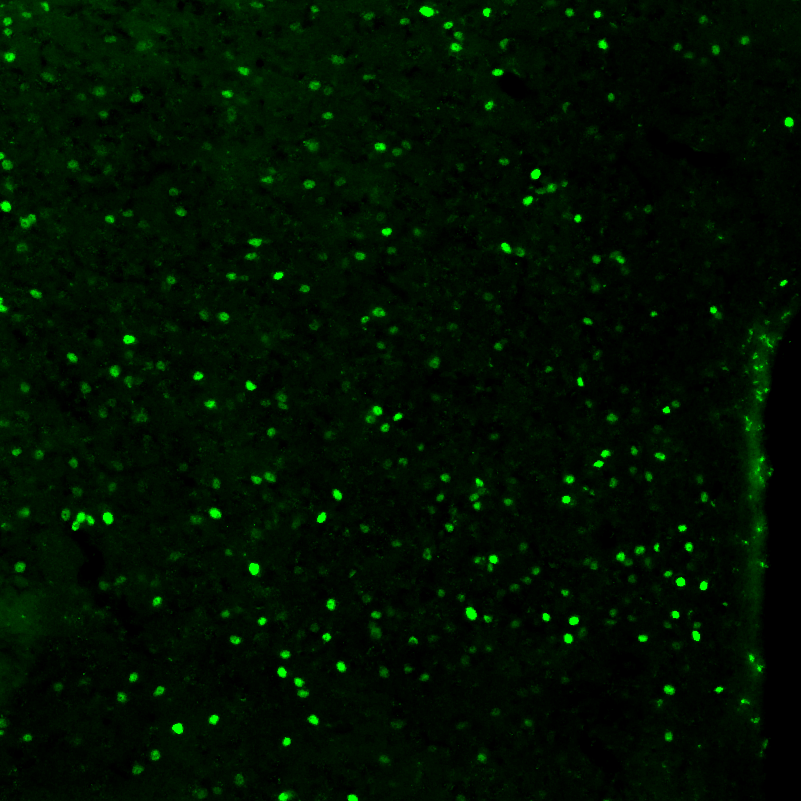

Supplement: Supplementary file 2 — Original pictures of cFos and Cy5 drug appearance shown in Extended Data Fig. 5a–h, including replicates used for quantification. [file 42255_2023_931_MOESM2_ESM.zip › Raw Data Extended Data Figure 5/02-DMH/53-M-WT-GIP-HYPO.tif]

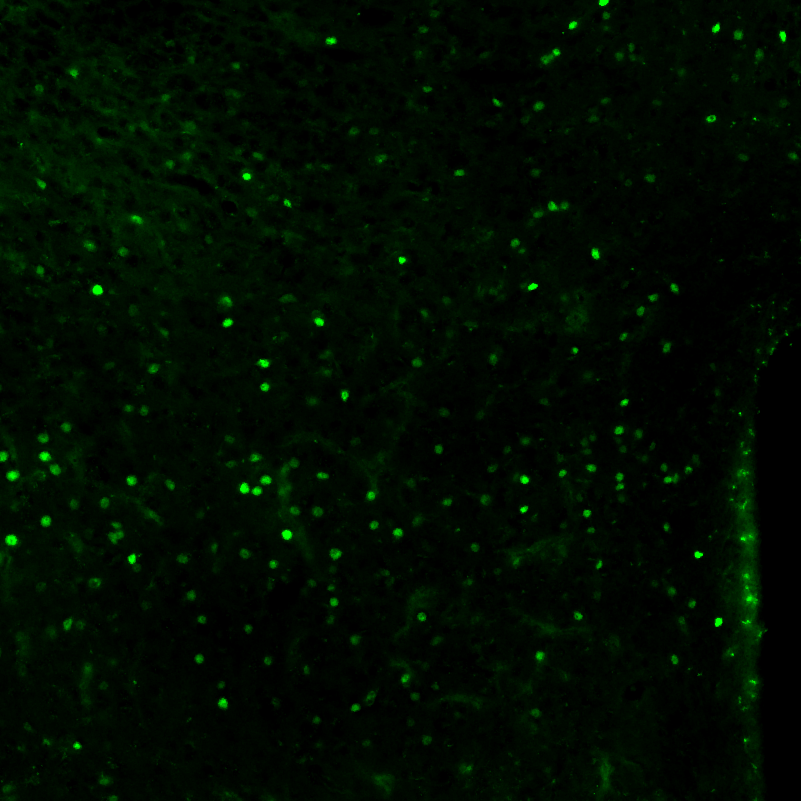

Supplement: Supplementary file 2 — Original pictures of cFos and Cy5 drug appearance shown in Extended Data Fig. 5a–h, including replicates used for quantification. [file 42255_2023_931_MOESM2_ESM.zip › Raw Data Extended Data Figure 5/02-DMH/65-M-WT-Veh-HYPO.tif]

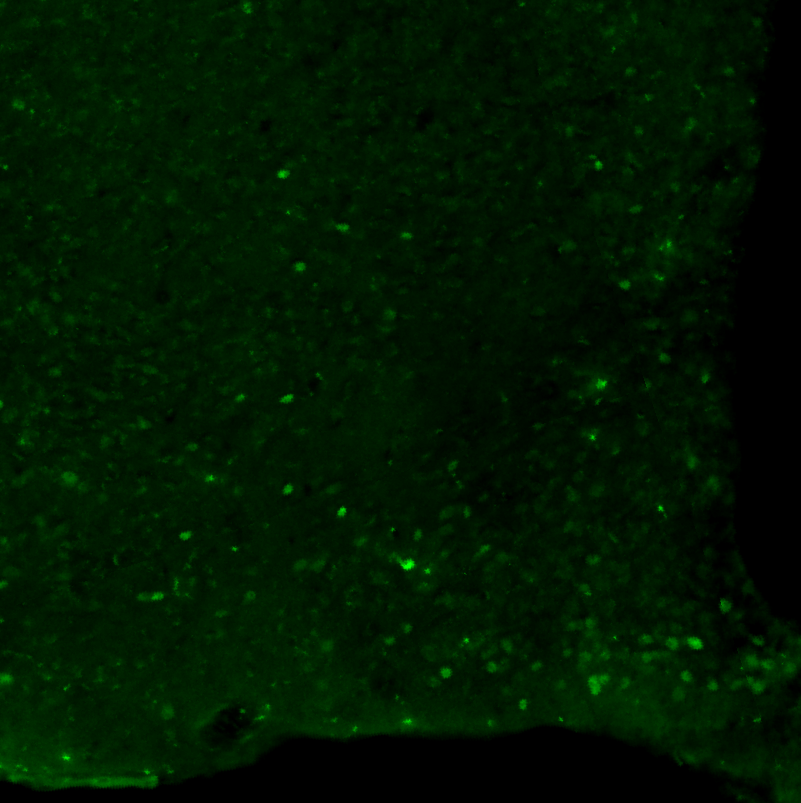

Supplement: Supplementary file 2 — Original pictures of cFos and Cy5 drug appearance shown in Extended Data Fig. 5a–h, including replicates used for quantification. [file 42255_2023_931_MOESM2_ESM.zip › Raw Data Extended Data Figure 5/01-ARC/2-M-KO-GIP-HYPO.tif]

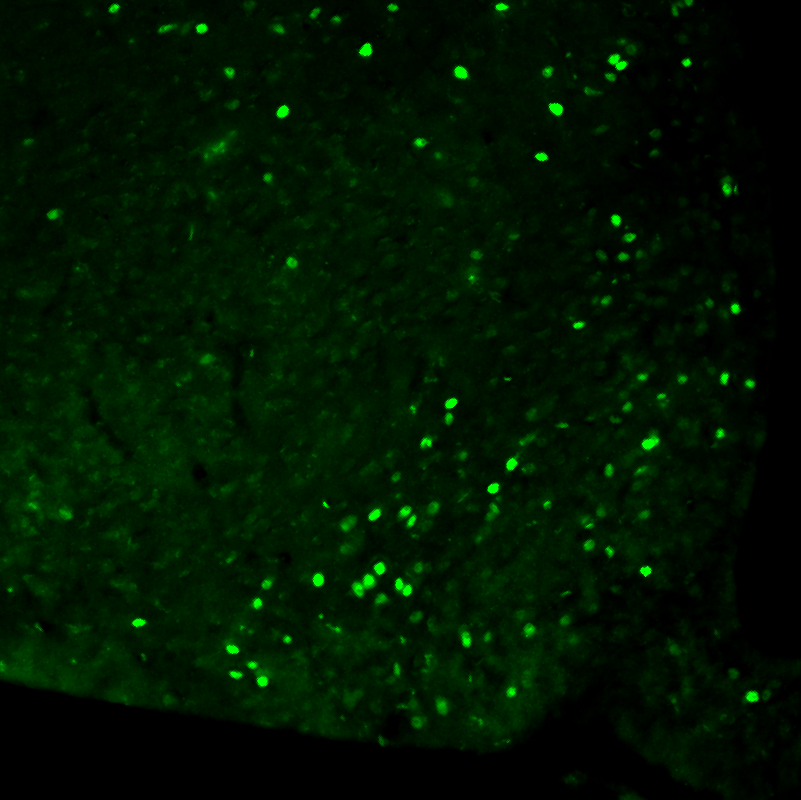

Supplement: Supplementary file 2 — Original pictures of cFos and Cy5 drug appearance shown in Extended Data Fig. 5a–h, including replicates used for quantification. [file 42255_2023_931_MOESM2_ESM.zip › Raw Data Extended Data Figure 5/01-ARC/46-M-WT-GIP-HYPO.tif]

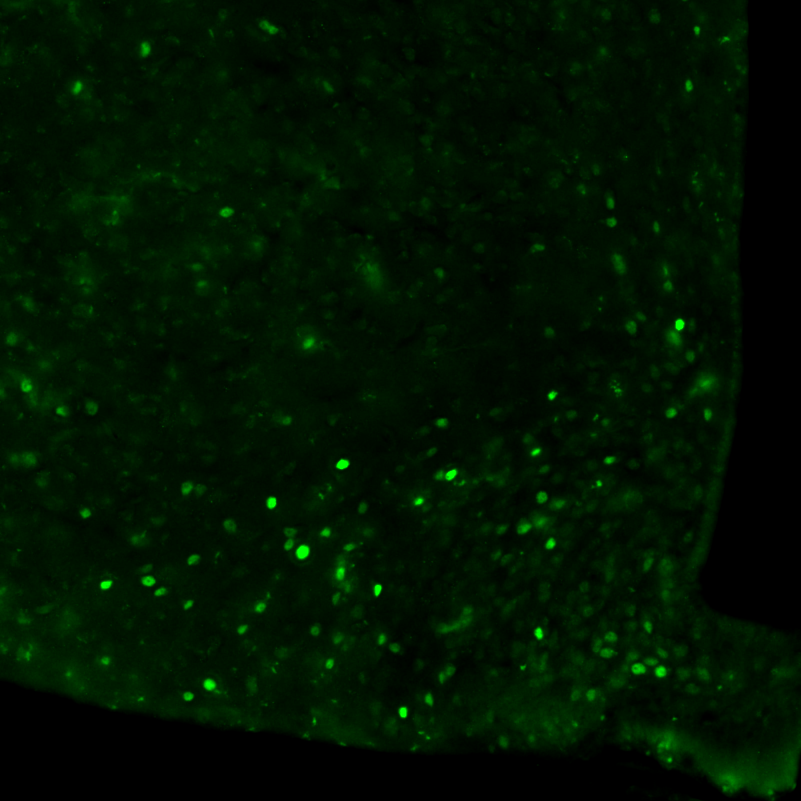

Supplement: Supplementary file 2 — Original pictures of cFos and Cy5 drug appearance shown in Extended Data Fig. 5a–h, including replicates used for quantification. [file 42255_2023_931_MOESM2_ESM.zip › Raw Data Extended Data Figure 5/01-ARC/17-M-KO-Veh-HYPO-ARC.tif]

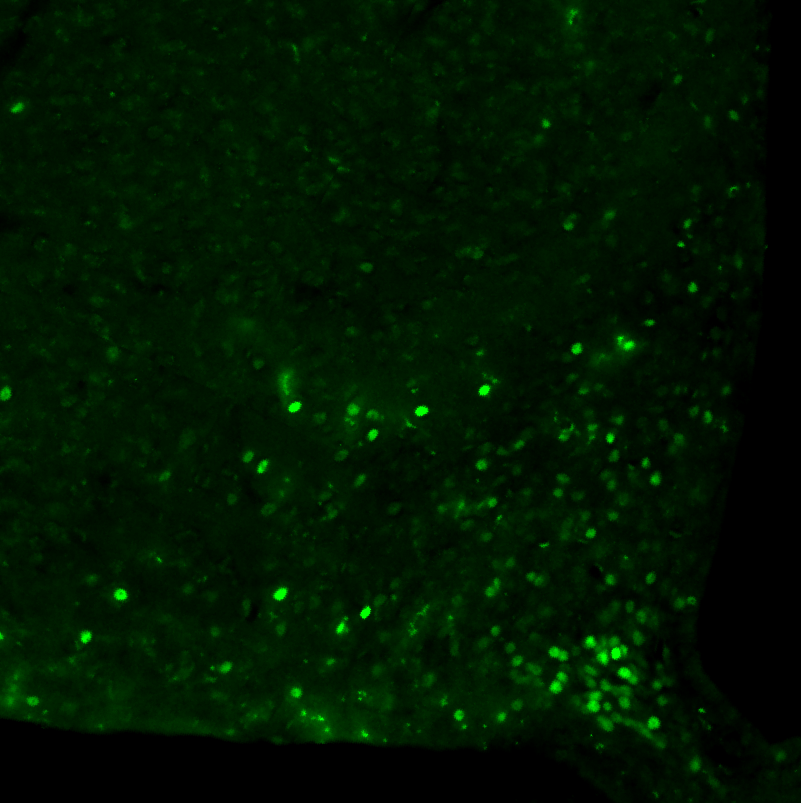

Supplement: Supplementary file 2 — Original pictures of cFos and Cy5 drug appearance shown in Extended Data Fig. 5a–h, including replicates used for quantification. [file 42255_2023_931_MOESM2_ESM.zip › Raw Data Extended Data Figure 5/01-ARC/3-M-KO-GIP-HYPO.tif]

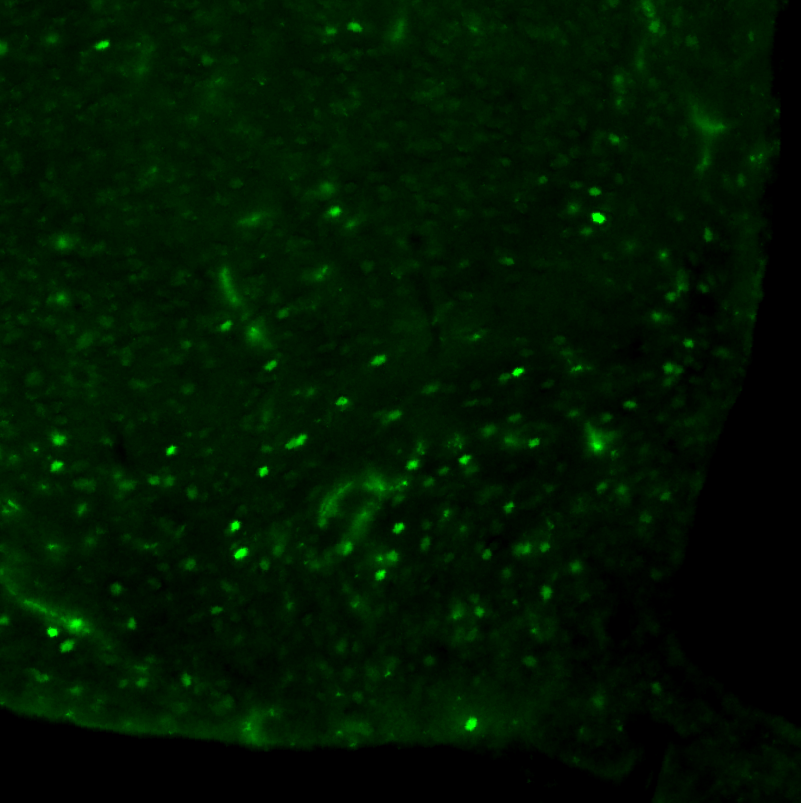

Supplement: Supplementary file 2 — Original pictures of cFos and Cy5 drug appearance shown in Extended Data Fig. 5a–h, including replicates used for quantification. [file 42255_2023_931_MOESM2_ESM.zip › Raw Data Extended Data Figure 5/01-ARC/36-M-KO-Veh-HYPO.tif]

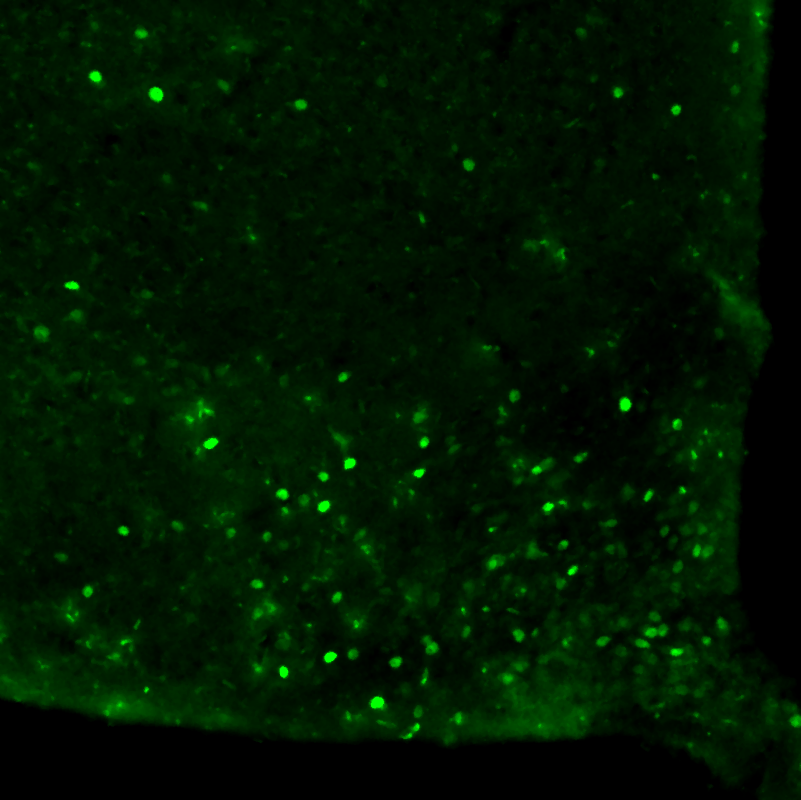

Supplement: Supplementary file 2 — Original pictures of cFos and Cy5 drug appearance shown in Extended Data Fig. 5a–h, including replicates used for quantification. [file 42255_2023_931_MOESM2_ESM.zip › Raw Data Extended Data Figure 5/01-ARC/44-M-WT-GIP-HYPO.tif]

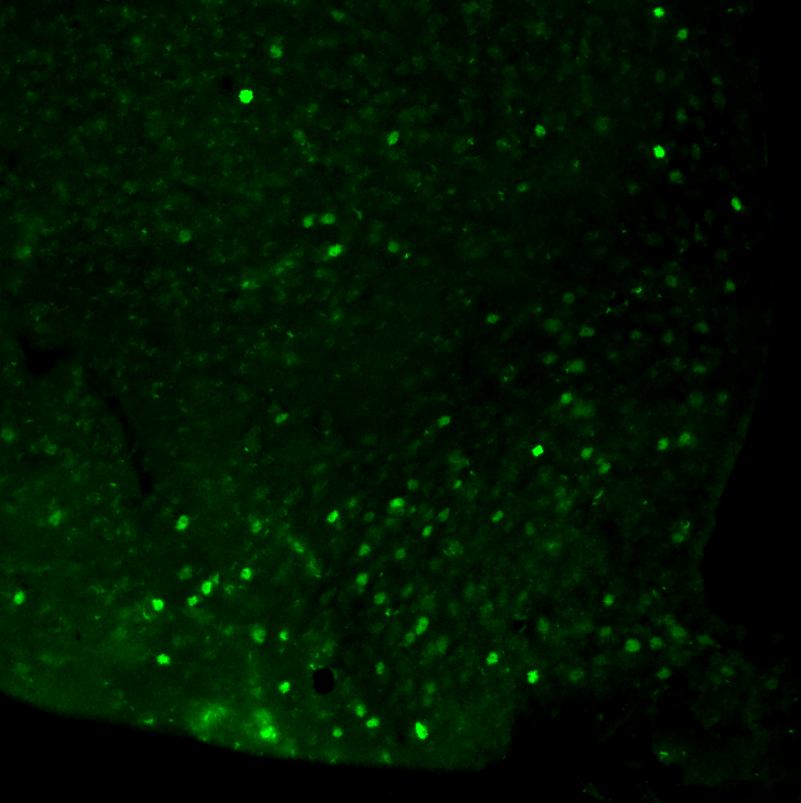

Supplement: Supplementary file 2 — Original pictures of cFos and Cy5 drug appearance shown in Extended Data Fig. 5a–h, including replicates used for quantification. [file 42255_2023_931_MOESM2_ESM.zip › Raw Data Extended Data Figure 5/01-ARC/1-M-KO-GIP-HYPO.tif]

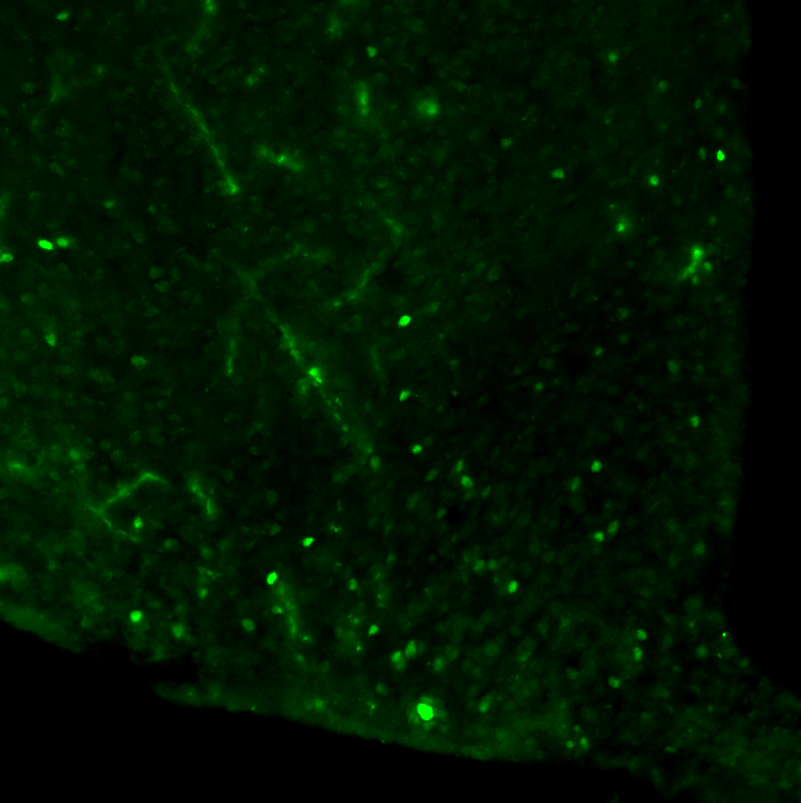

Supplement: Supplementary file 2 — Original pictures of cFos and Cy5 drug appearance shown in Extended Data Fig. 5a–h, including replicates used for quantification. [file 42255_2023_931_MOESM2_ESM.zip › Raw Data Extended Data Figure 5/01-ARC/37-M-KO-Veh-HYPO.tif]

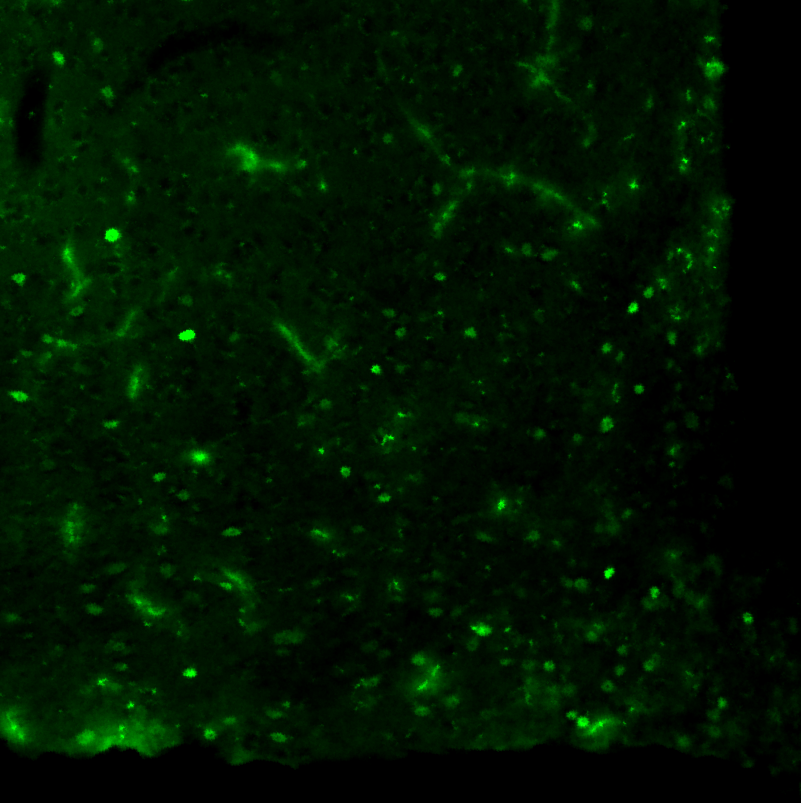

Supplement: Supplementary file 2 — Original pictures of cFos and Cy5 drug appearance shown in Extended Data Fig. 5a–h, including replicates used for quantification. [file 42255_2023_931_MOESM2_ESM.zip › Raw Data Extended Data Figure 5/01-ARC/66-M-WT-Veh-HYPO.tif]

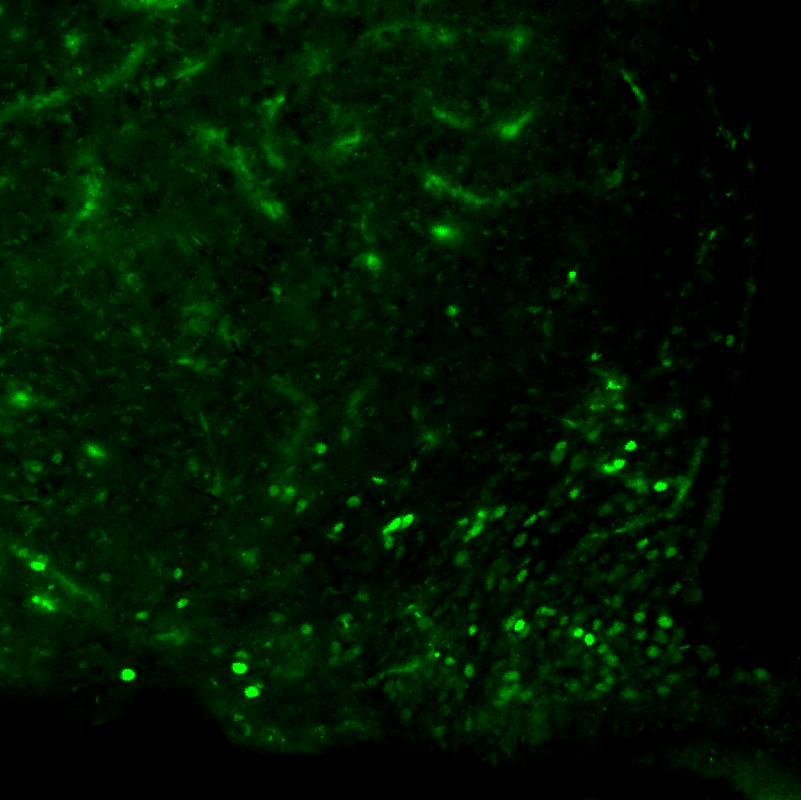

Supplement: Supplementary file 2 — Original pictures of cFos and Cy5 drug appearance shown in Extended Data Fig. 5a–h, including replicates used for quantification. [file 42255_2023_931_MOESM2_ESM.zip › Raw Data Extended Data Figure 5/01-ARC/55-M-WT-GIP-HYPO.tif]

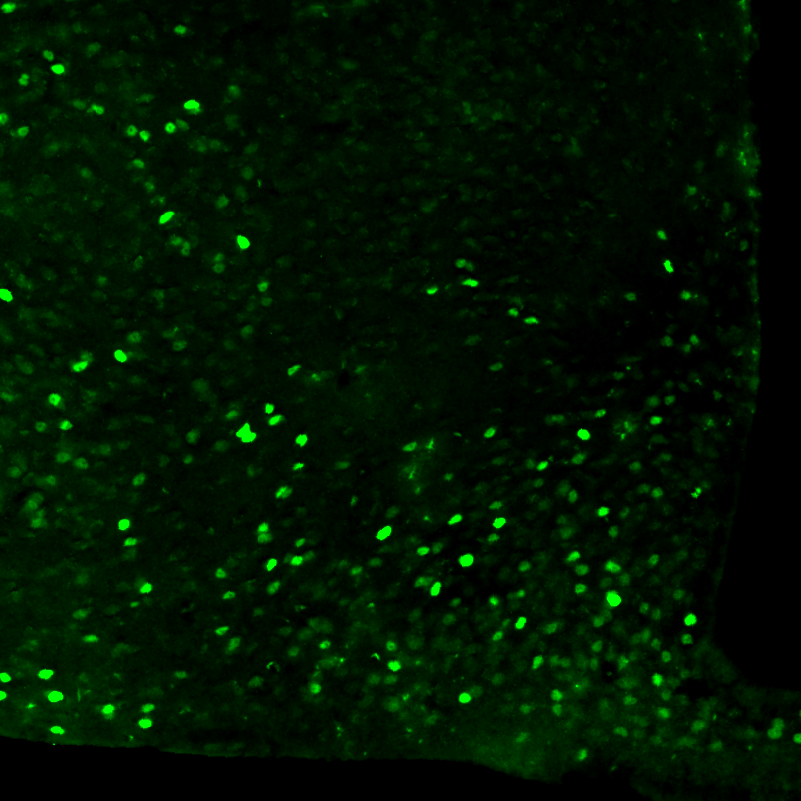

Supplement: Supplementary file 2 — Original pictures of cFos and Cy5 drug appearance shown in Extended Data Fig. 5a–h, including replicates used for quantification. [file 42255_2023_931_MOESM2_ESM.zip › Raw Data Extended Data Figure 5/01-ARC/14-M-KO-GIP-HYPO.tif]

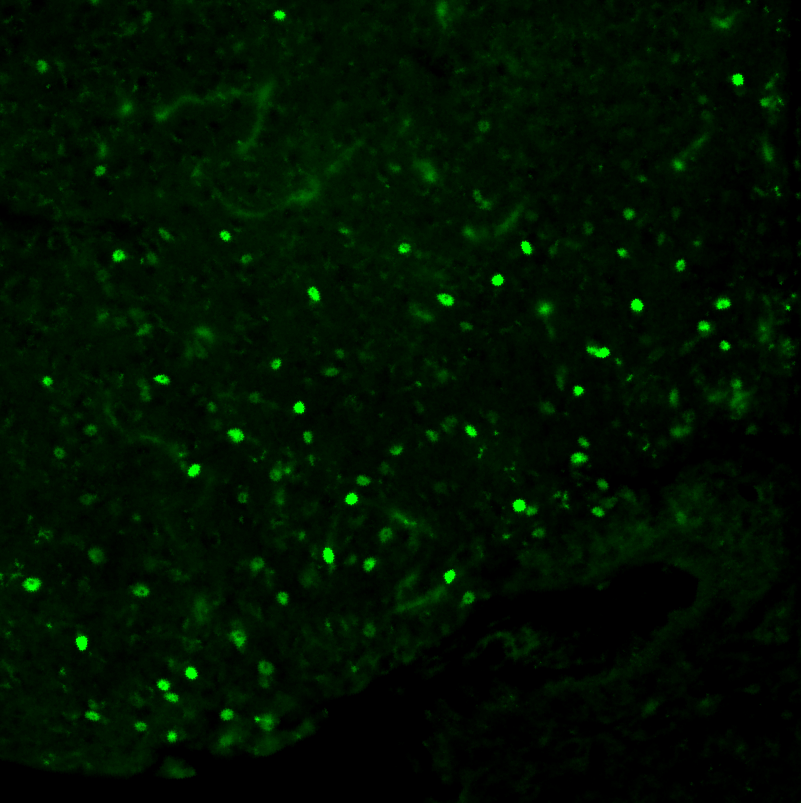

Supplement: Supplementary file 2 — Original pictures of cFos and Cy5 drug appearance shown in Extended Data Fig. 5a–h, including replicates used for quantification. [file 42255_2023_931_MOESM2_ESM.zip › Raw Data Extended Data Figure 5/01-ARC/67-M-WT-Veh-HYPO.tif]

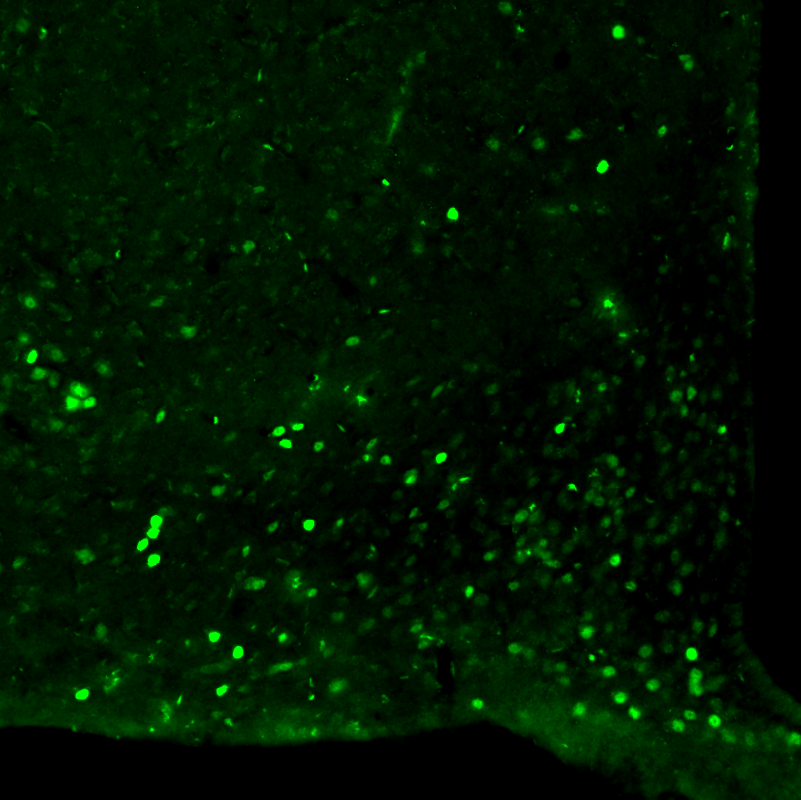

Supplement: Supplementary file 2 — Original pictures of cFos and Cy5 drug appearance shown in Extended Data Fig. 5a–h, including replicates used for quantification. [file 42255_2023_931_MOESM2_ESM.zip › Raw Data Extended Data Figure 5/01-ARC/54-M-WT-GIP-HYPO.tif]

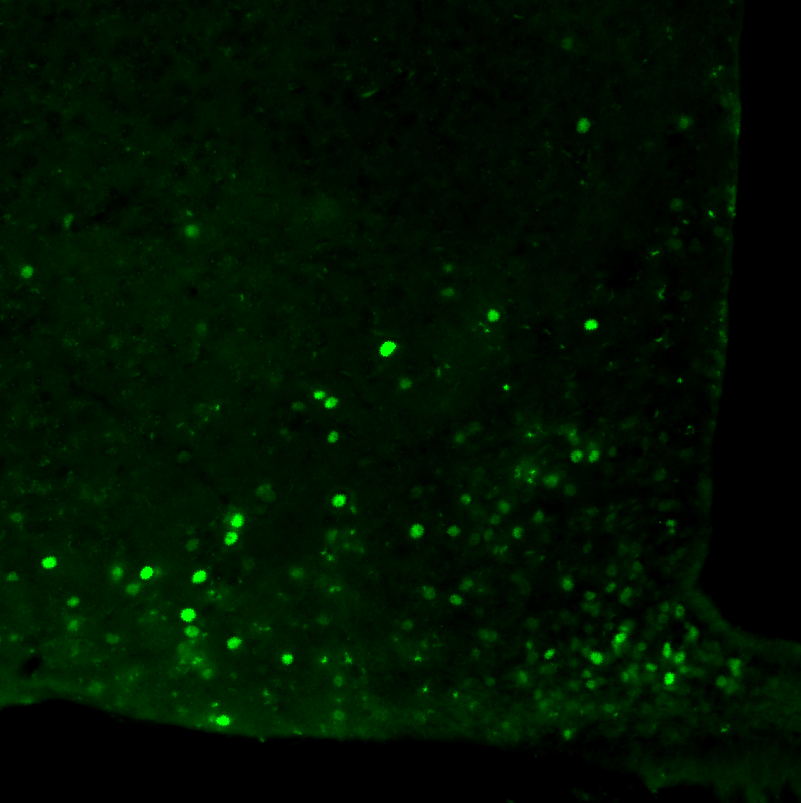

Supplement: Supplementary file 2 — Original pictures of cFos and Cy5 drug appearance shown in Extended Data Fig. 5a–h, including replicates used for quantification. [file 42255_2023_931_MOESM2_ESM.zip › Raw Data Extended Data Figure 5/01-ARC/15-M-KO-GIP-HYPO.tif]

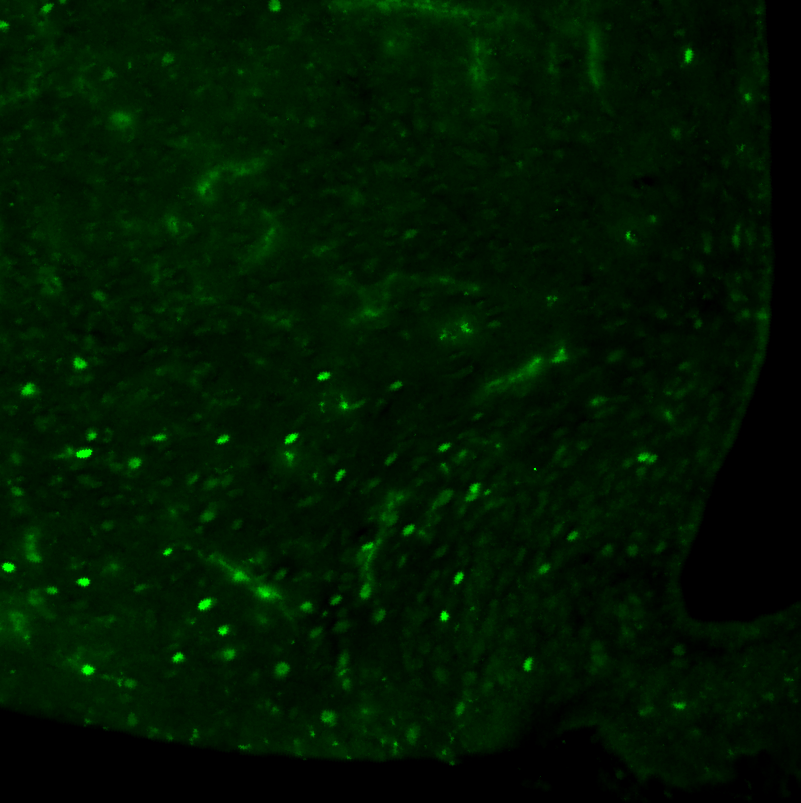

Supplement: Supplementary file 2 — Original pictures of cFos and Cy5 drug appearance shown in Extended Data Fig. 5a–h, including replicates used for quantification. [file 42255_2023_931_MOESM2_ESM.zip › Raw Data Extended Data Figure 5/01-ARC/20-M-KO-Veh-HYPO.tif]

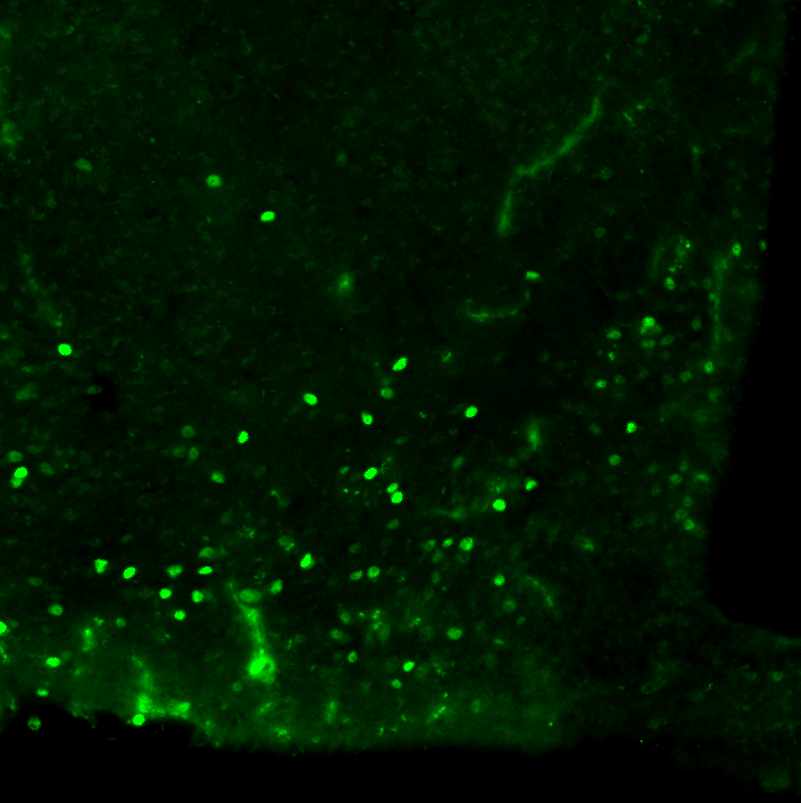

Supplement: Supplementary file 2 — Original pictures of cFos and Cy5 drug appearance shown in Extended Data Fig. 5a–h, including replicates used for quantification. [file 42255_2023_931_MOESM2_ESM.zip › Raw Data Extended Data Figure 5/01-ARC/64-M-WT-Veh-HYPO.tif]

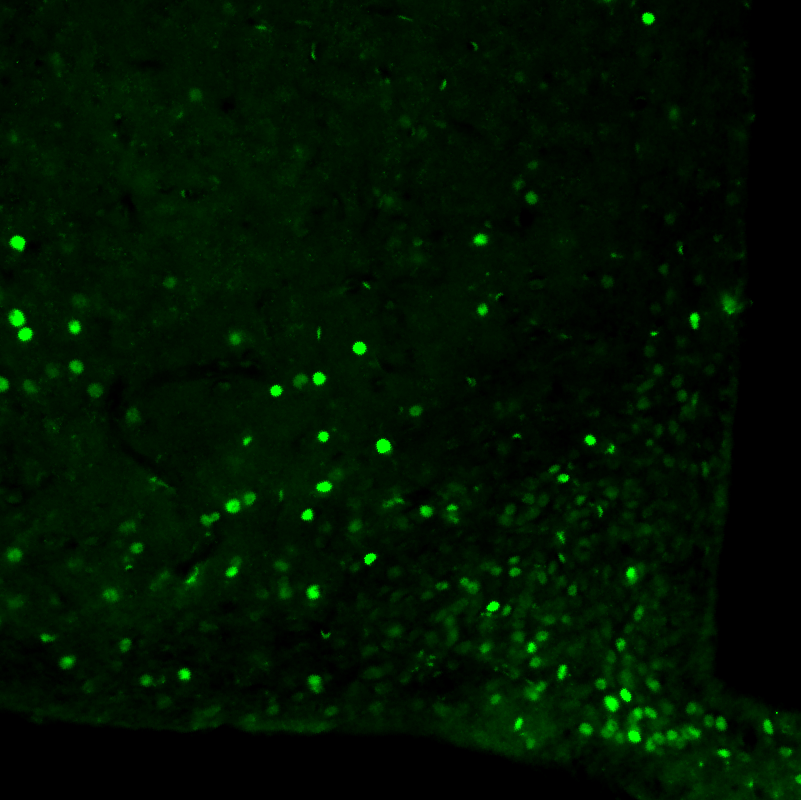

Supplement: Supplementary file 2 — Original pictures of cFos and Cy5 drug appearance shown in Extended Data Fig. 5a–h, including replicates used for quantification. [file 42255_2023_931_MOESM2_ESM.zip › Raw Data Extended Data Figure 5/01-ARC/53-M-WT-GIP-HYPO.tif]

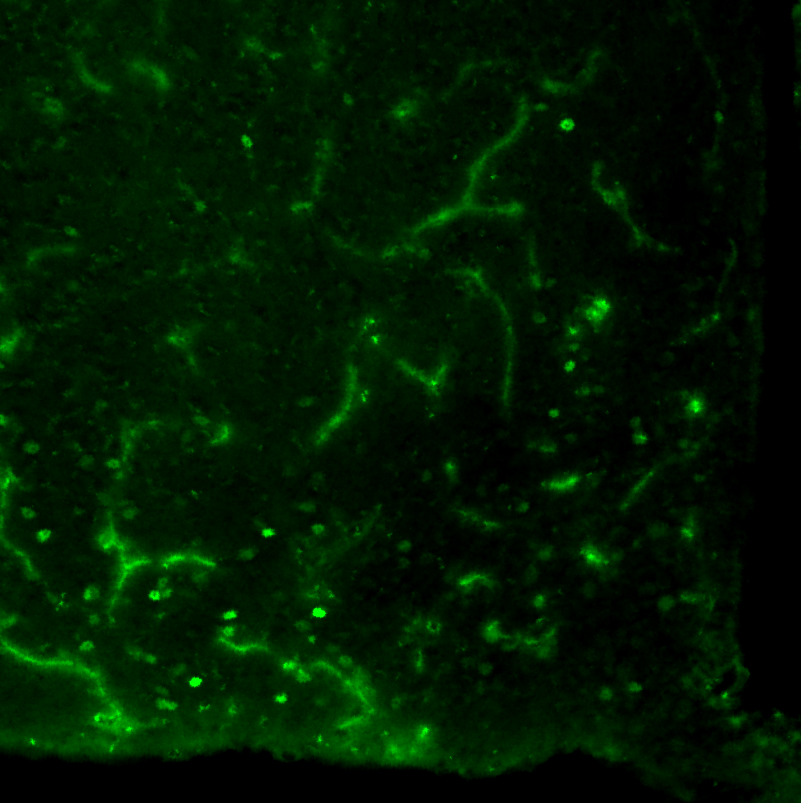

Supplement: Supplementary file 2 — Original pictures of cFos and Cy5 drug appearance shown in Extended Data Fig. 5a–h, including replicates used for quantification. [file 42255_2023_931_MOESM2_ESM.zip › Raw Data Extended Data Figure 5/01-ARC/65-M-WT-Veh-HYPO.tif]

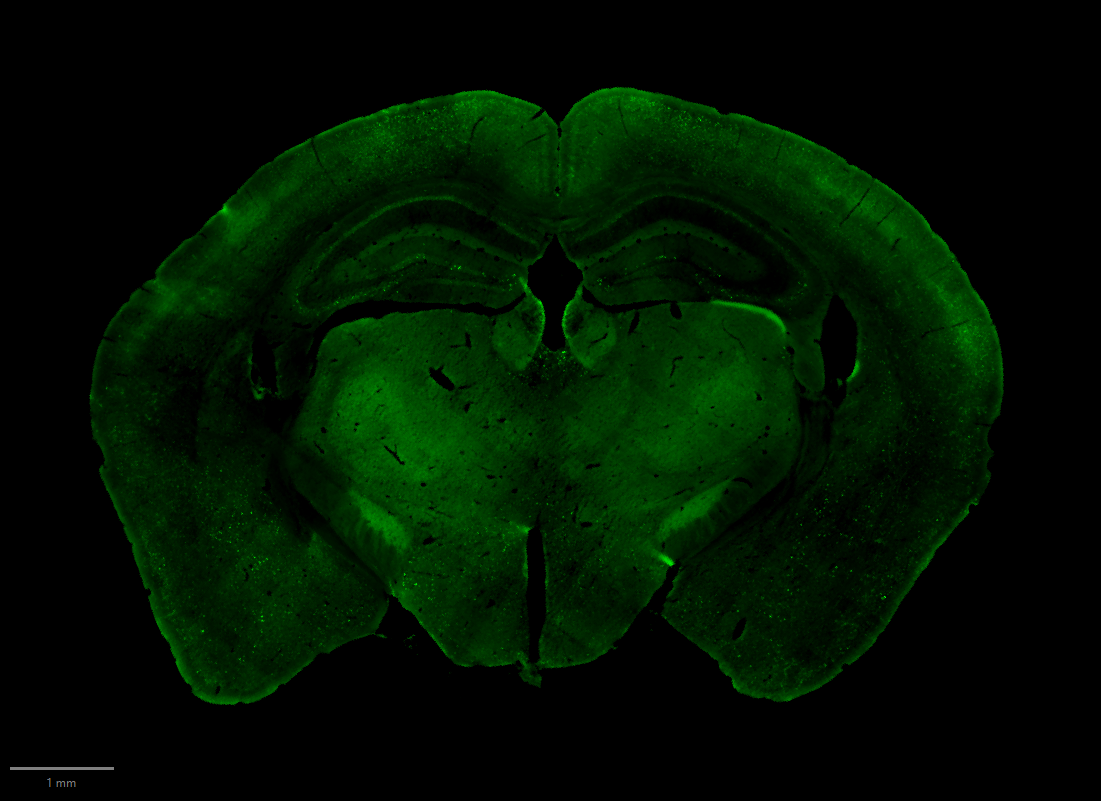

Supplement: Supplementary file 2 — Original pictures of cFos and Cy5 drug appearance shown in Extended Data Fig. 5a–h, including replicates used for quantification. [file 42255_2023_931_MOESM2_ESM.zip › Raw Data Extended Data Figure 5/07-whole brain images/2-M-KO-GIP-HYPO.tif]

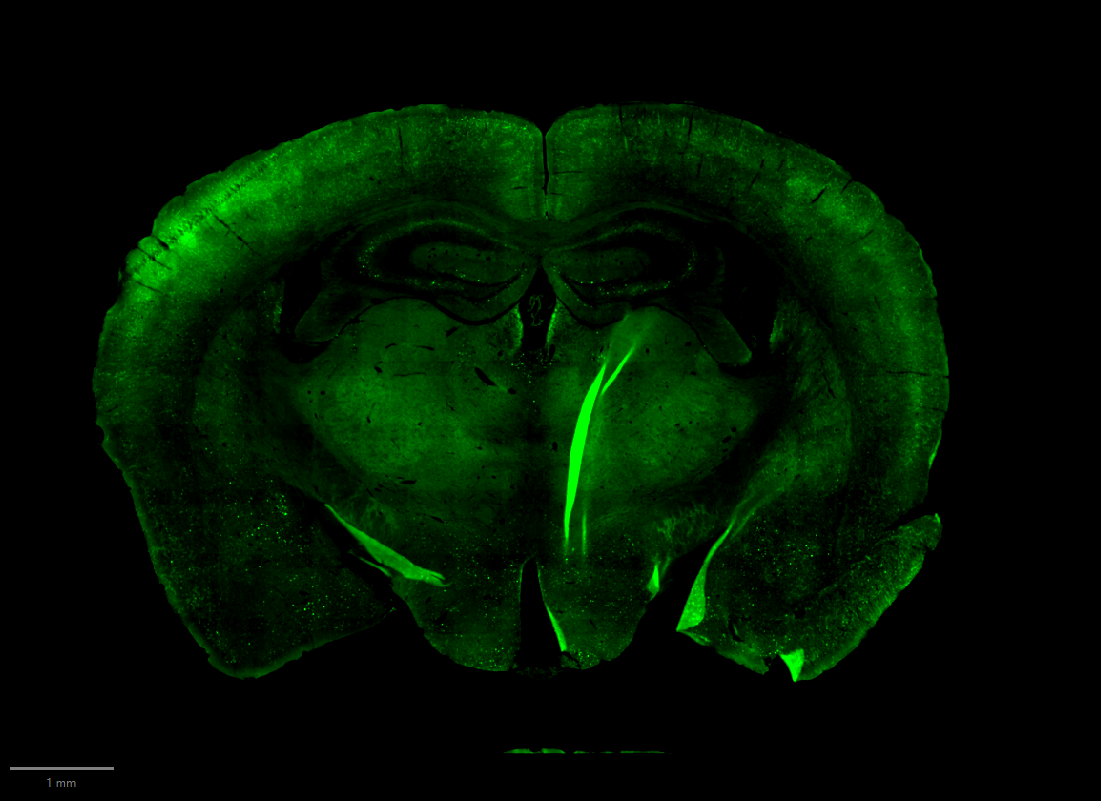

Supplement: Supplementary file 2 — Original pictures of cFos and Cy5 drug appearance shown in Extended Data Fig. 5a–h, including replicates used for quantification. [file 42255_2023_931_MOESM2_ESM.zip › Raw Data Extended Data Figure 5/07-whole brain images/46-M-WT-GIP-HYPO.tif]

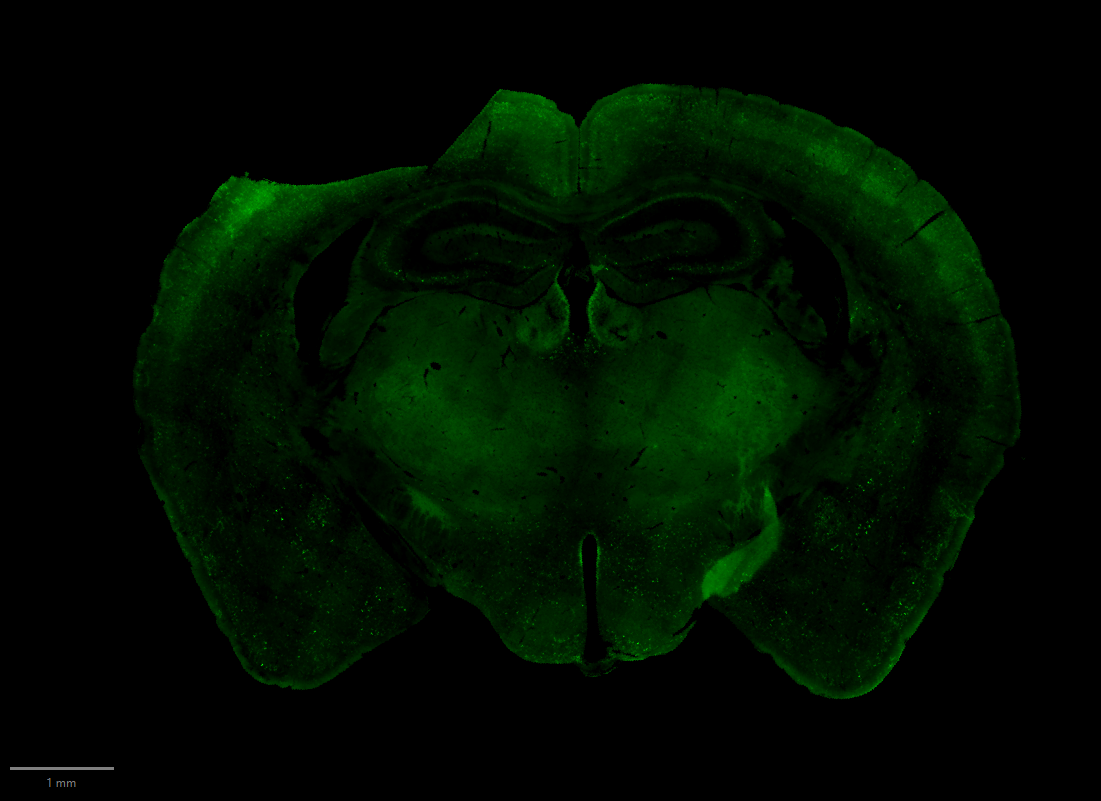

Supplement: Supplementary file 2 — Original pictures of cFos and Cy5 drug appearance shown in Extended Data Fig. 5a–h, including replicates used for quantification. [file 42255_2023_931_MOESM2_ESM.zip › Raw Data Extended Data Figure 5/07-whole brain images/3-M-KO-GIP-HYPO.tif]

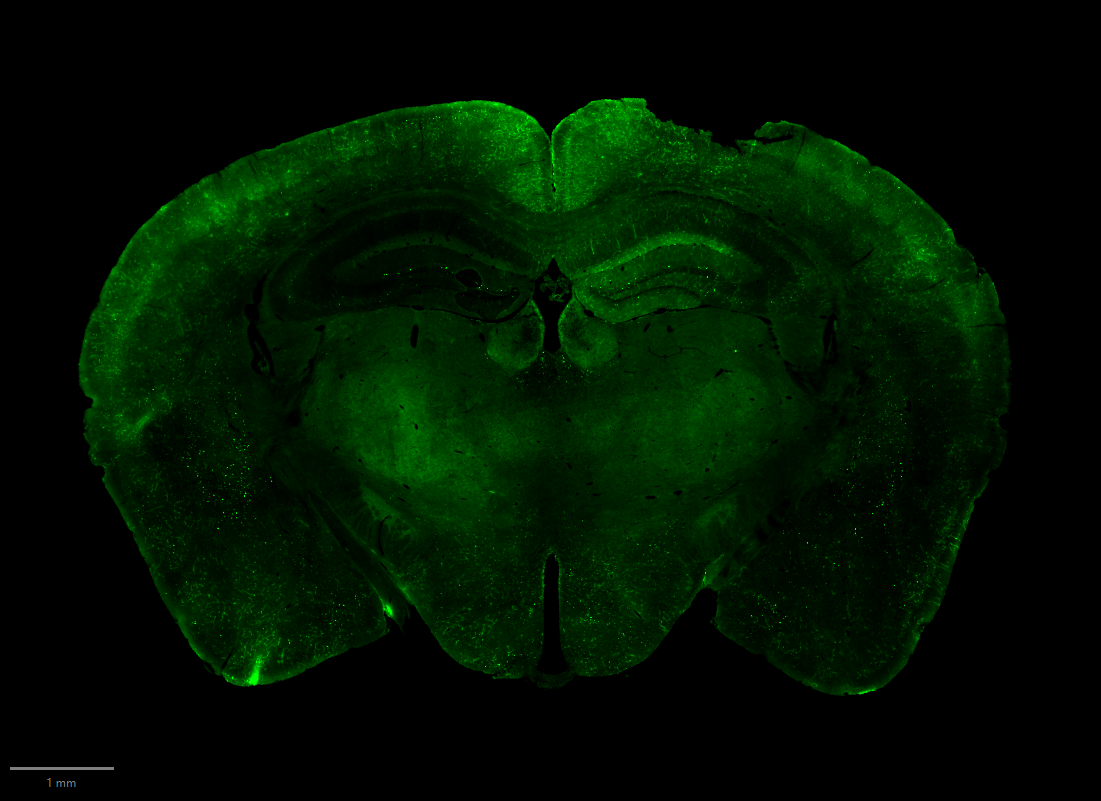

Supplement: Supplementary file 2 — Original pictures of cFos and Cy5 drug appearance shown in Extended Data Fig. 5a–h, including replicates used for quantification. [file 42255_2023_931_MOESM2_ESM.zip › Raw Data Extended Data Figure 5/07-whole brain images/36-M-KO-Veh-HYPO.tif]

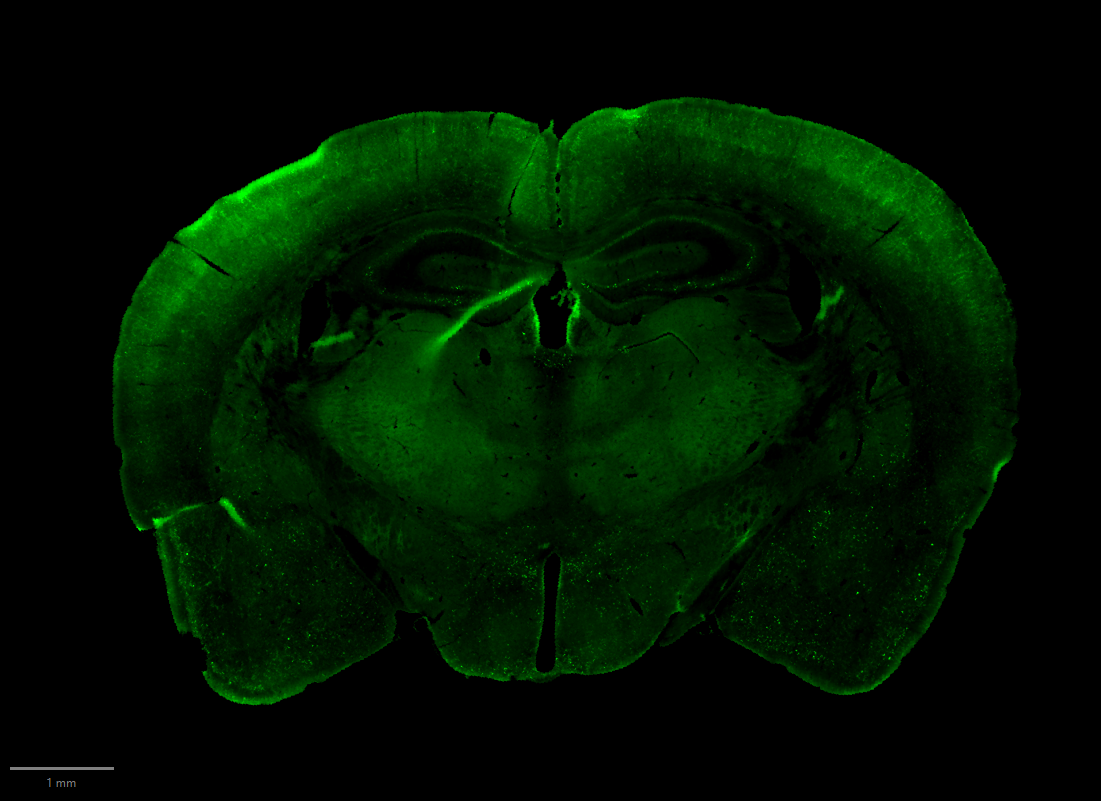

Supplement: Supplementary file 2 — Original pictures of cFos and Cy5 drug appearance shown in Extended Data Fig. 5a–h, including replicates used for quantification. [file 42255_2023_931_MOESM2_ESM.zip › Raw Data Extended Data Figure 5/07-whole brain images/44-M-WT-GIP-HYPO.tif]

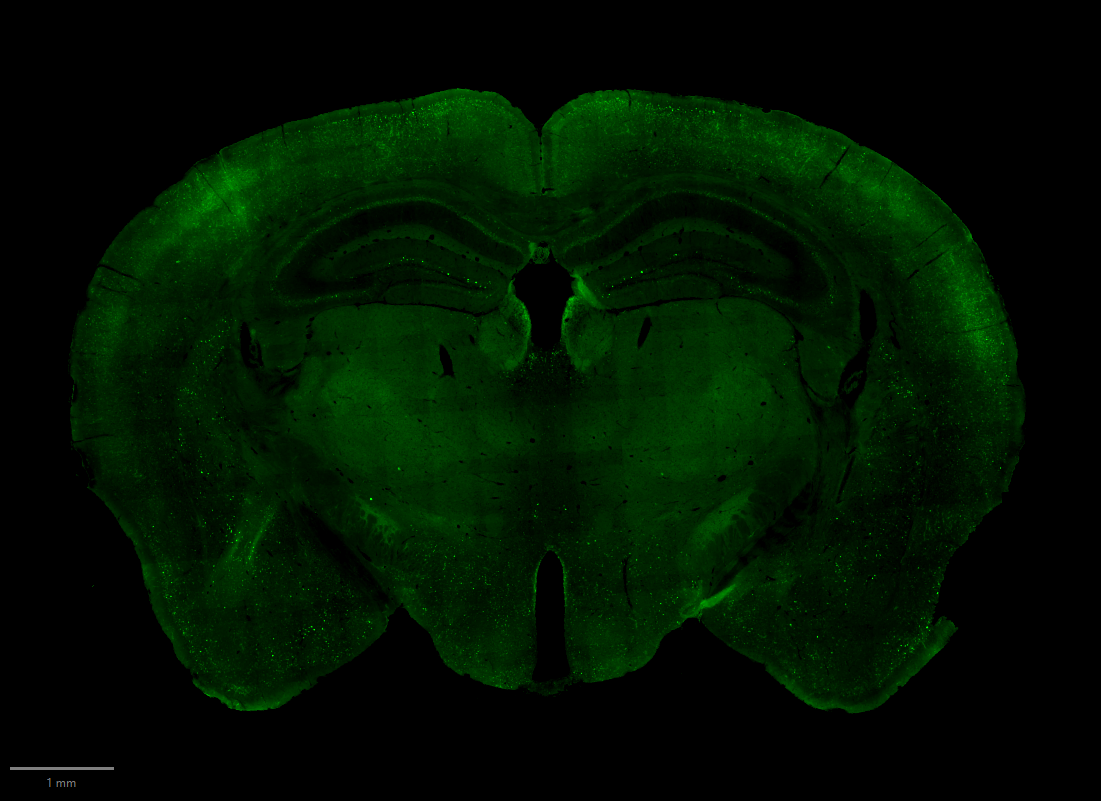

Supplement: Supplementary file 2 — Original pictures of cFos and Cy5 drug appearance shown in Extended Data Fig. 5a–h, including replicates used for quantification. [file 42255_2023_931_MOESM2_ESM.zip › Raw Data Extended Data Figure 5/07-whole brain images/1-M-KO-GIP-HYPO.tif]

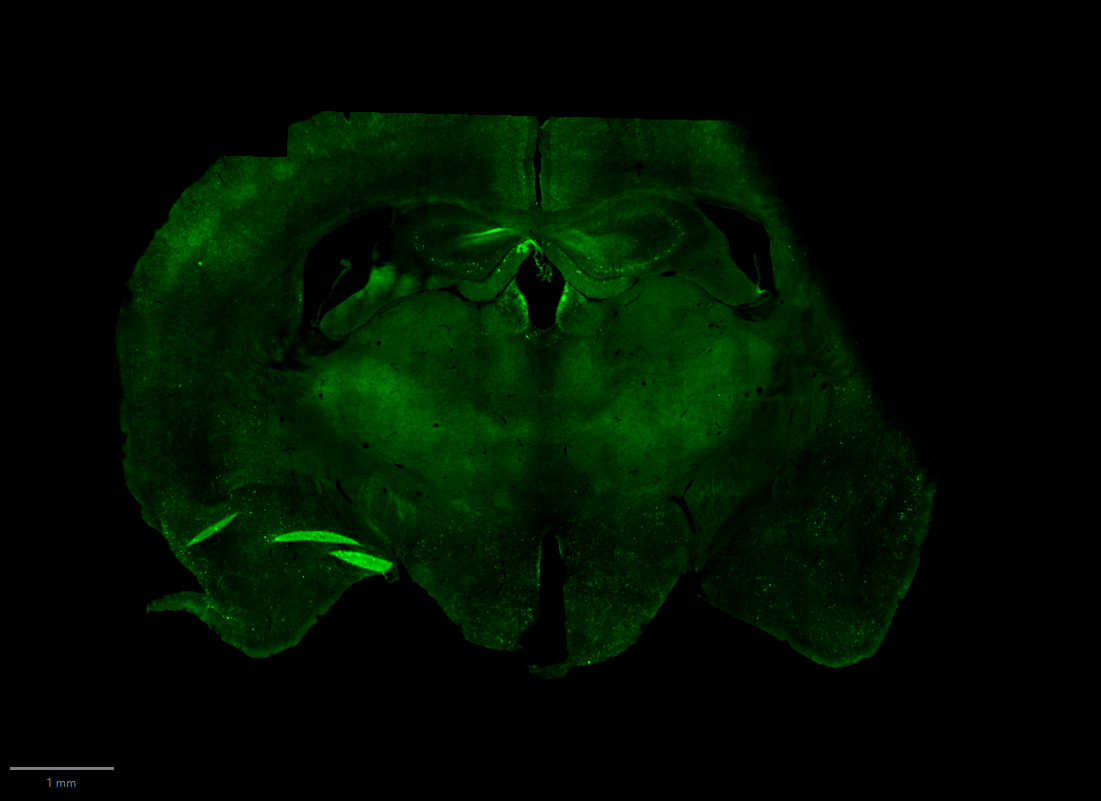

Supplement: Supplementary file 2 — Original pictures of cFos and Cy5 drug appearance shown in Extended Data Fig. 5a–h, including replicates used for quantification. [file 42255_2023_931_MOESM2_ESM.zip › Raw Data Extended Data Figure 5/07-whole brain images/37-M-KO-Veh-HYPO.tif]

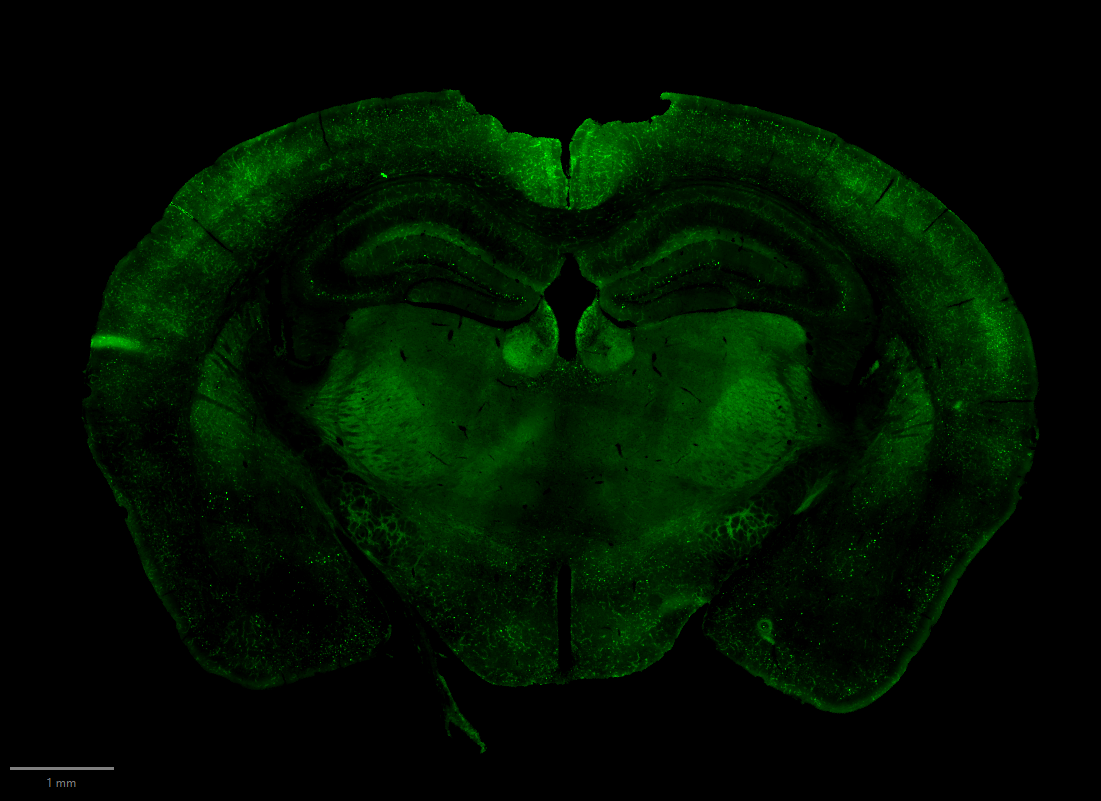

Supplement: Supplementary file 2 — Original pictures of cFos and Cy5 drug appearance shown in Extended Data Fig. 5a–h, including replicates used for quantification. [file 42255_2023_931_MOESM2_ESM.zip › Raw Data Extended Data Figure 5/07-whole brain images/66-M-WT-Veh-HYPO.tif]

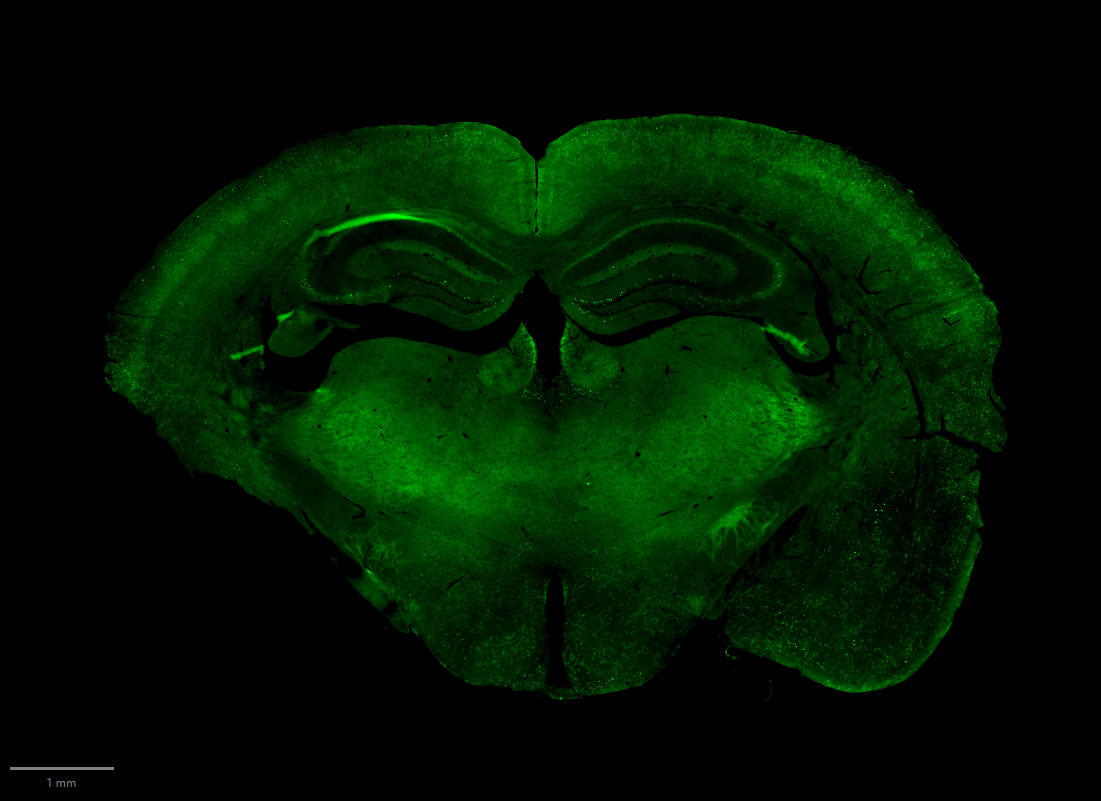

Supplement: Supplementary file 2 — Original pictures of cFos and Cy5 drug appearance shown in Extended Data Fig. 5a–h, including replicates used for quantification. [file 42255_2023_931_MOESM2_ESM.zip › Raw Data Extended Data Figure 5/07-whole brain images/55-M-WT-GIP-HYPO.tif]

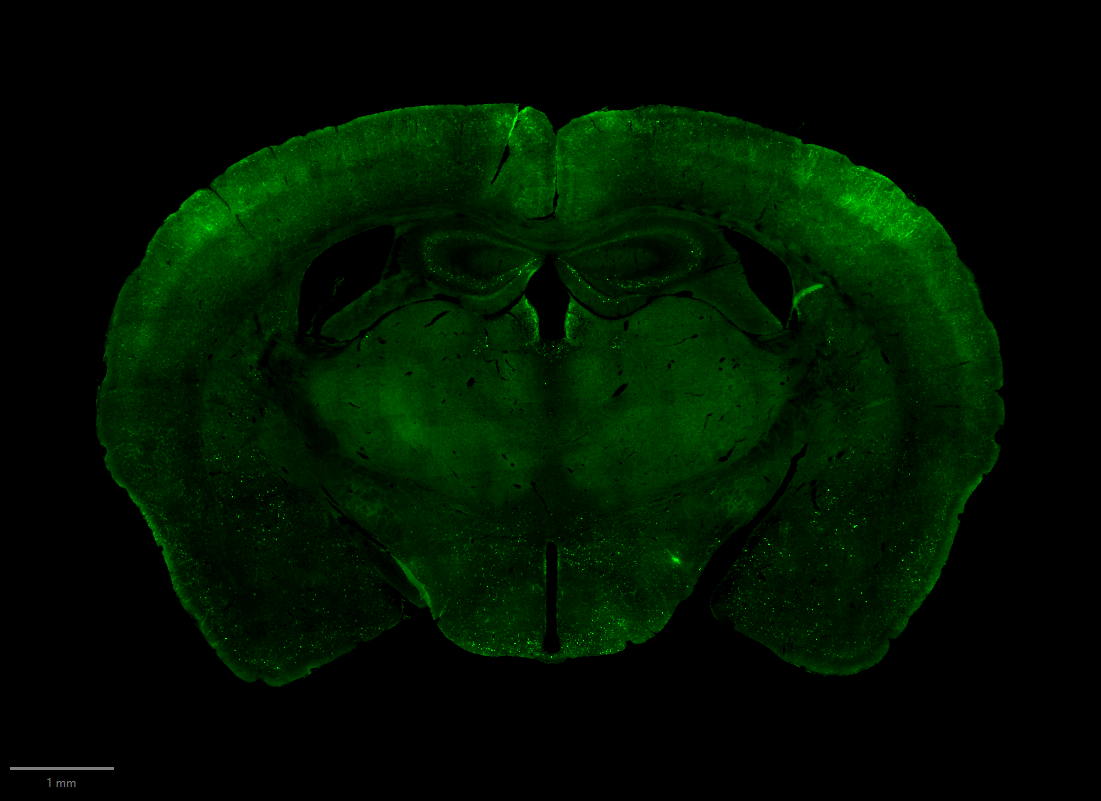

Supplement: Supplementary file 2 — Original pictures of cFos and Cy5 drug appearance shown in Extended Data Fig. 5a–h, including replicates used for quantification. [file 42255_2023_931_MOESM2_ESM.zip › Raw Data Extended Data Figure 5/07-whole brain images/14-M-KO-GIP-HYPO.tif]

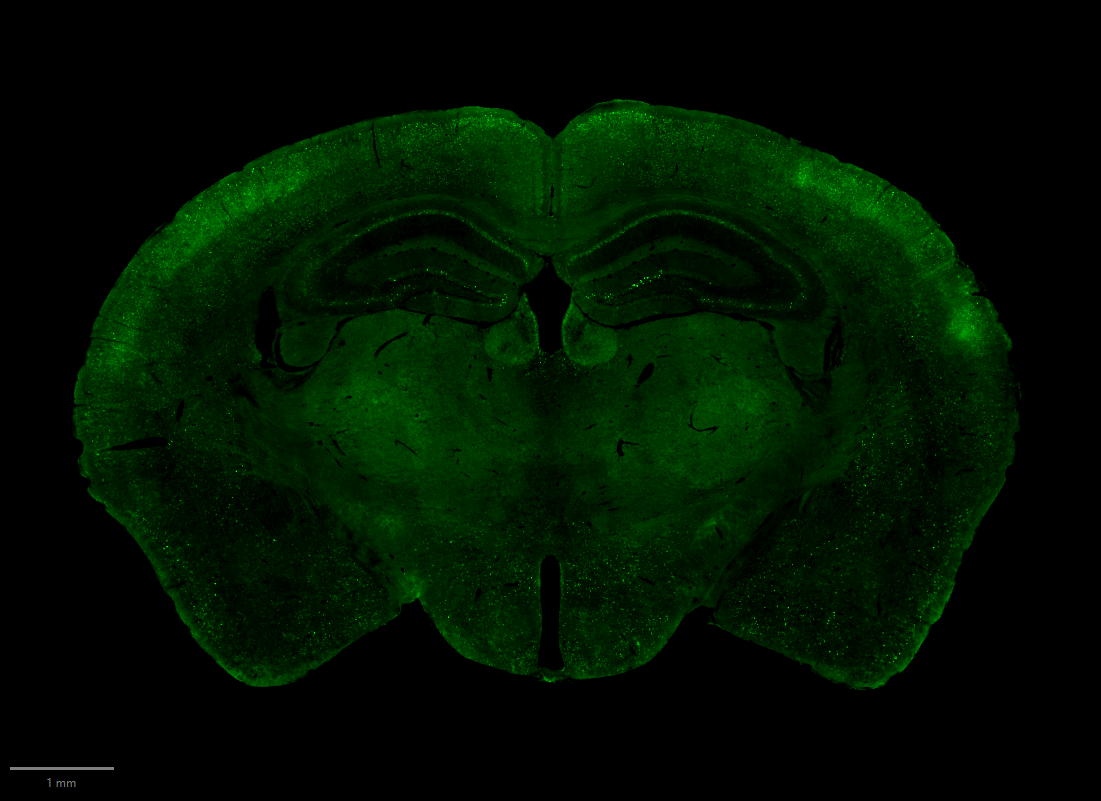

Supplement: Supplementary file 2 — Original pictures of cFos and Cy5 drug appearance shown in Extended Data Fig. 5a–h, including replicates used for quantification. [file 42255_2023_931_MOESM2_ESM.zip › Raw Data Extended Data Figure 5/07-whole brain images/17-M-KO-Veh-HYPO.tif]

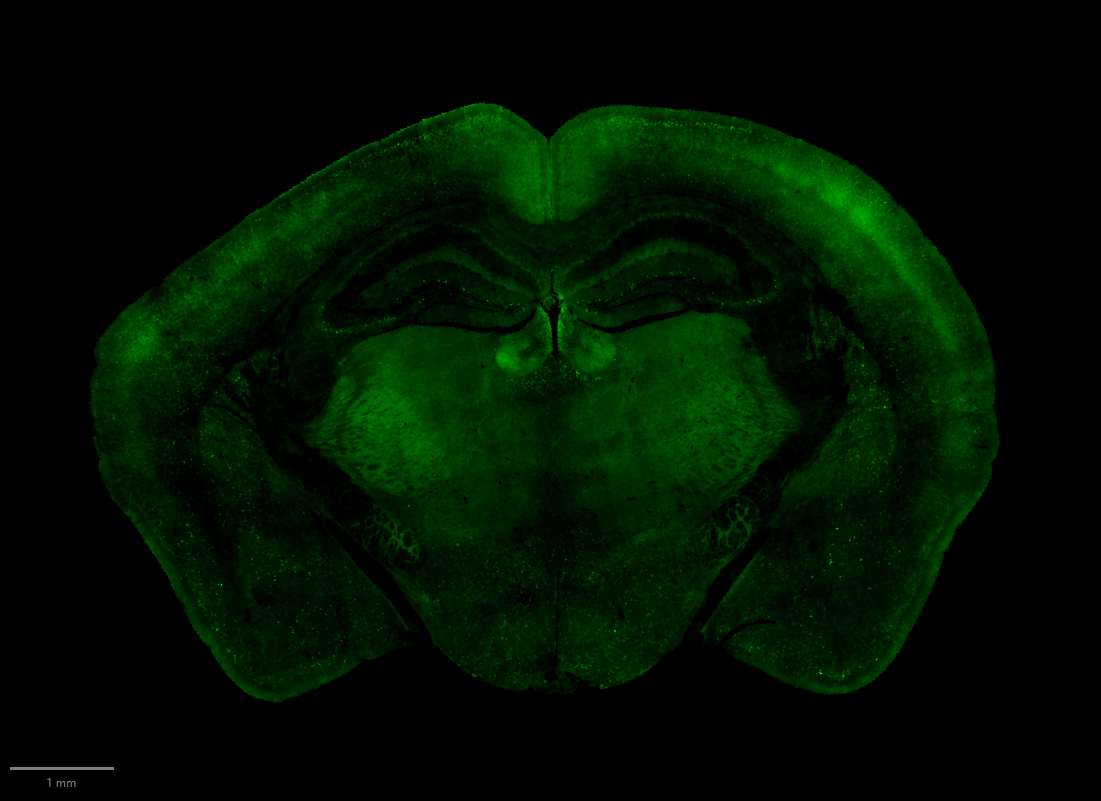

Supplement: Supplementary file 2 — Original pictures of cFos and Cy5 drug appearance shown in Extended Data Fig. 5a–h, including replicates used for quantification. [file 42255_2023_931_MOESM2_ESM.zip › Raw Data Extended Data Figure 5/07-whole brain images/67-M-WT-Veh-HYPO.tif]

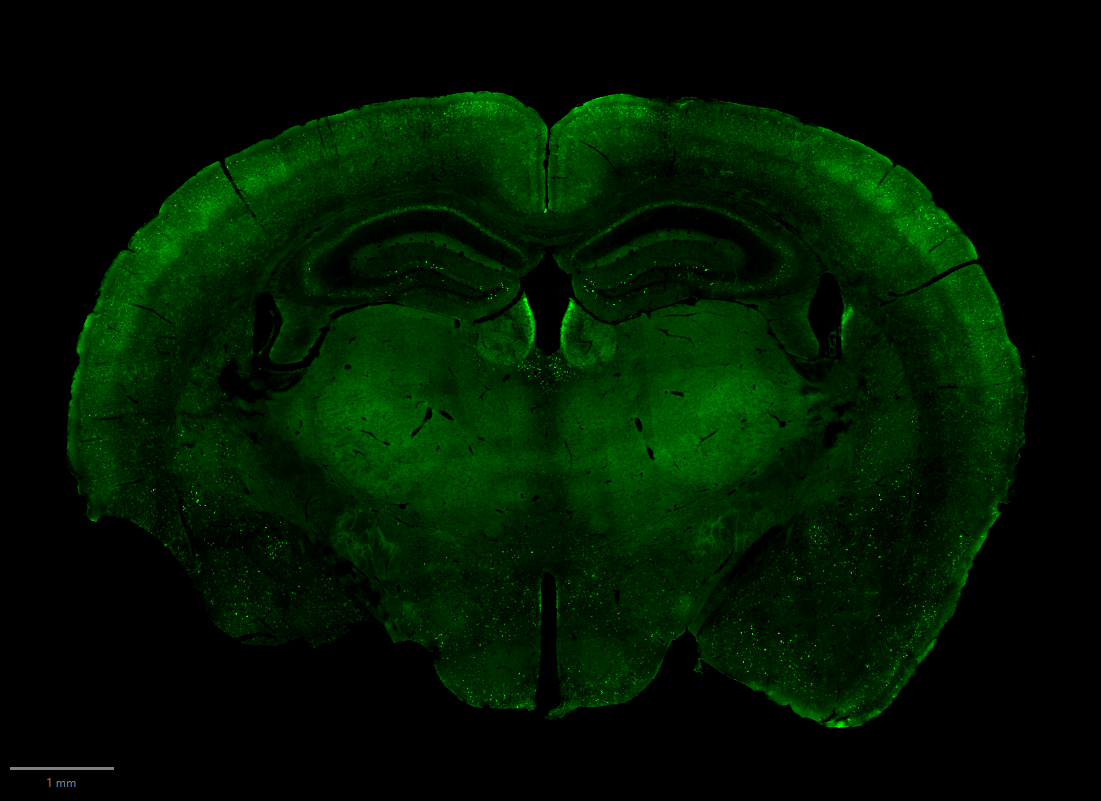

Supplement: Supplementary file 2 — Original pictures of cFos and Cy5 drug appearance shown in Extended Data Fig. 5a–h, including replicates used for quantification. [file 42255_2023_931_MOESM2_ESM.zip › Raw Data Extended Data Figure 5/07-whole brain images/54-M-WT-GIP-HYPO.tif]

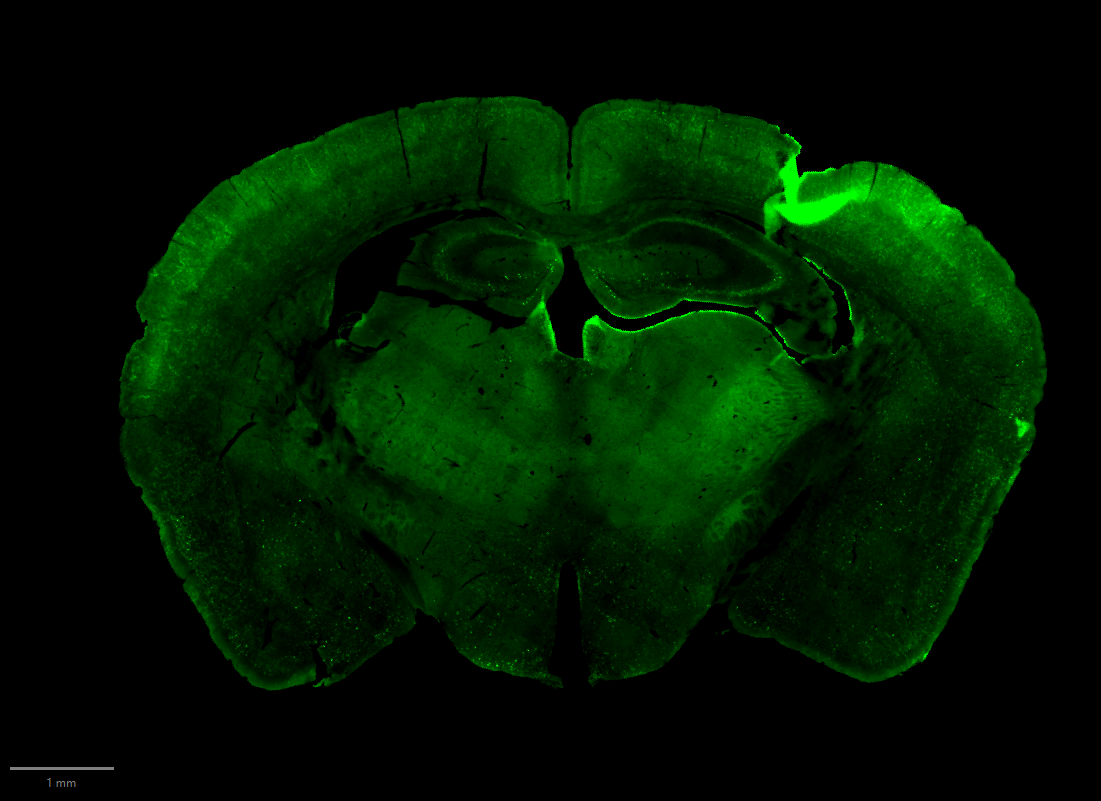

Supplement: Supplementary file 2 — Original pictures of cFos and Cy5 drug appearance shown in Extended Data Fig. 5a–h, including replicates used for quantification. [file 42255_2023_931_MOESM2_ESM.zip › Raw Data Extended Data Figure 5/07-whole brain images/15-M-KO-GIP-HYPO.tif]

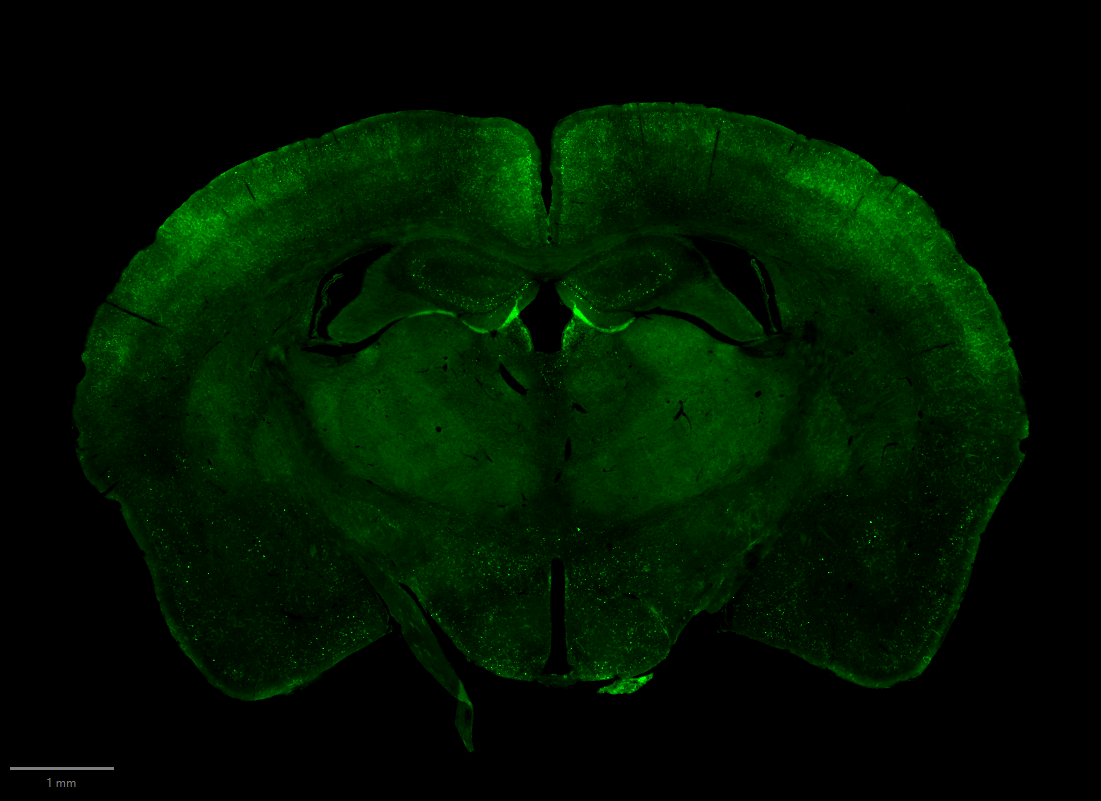

Supplement: Supplementary file 2 — Original pictures of cFos and Cy5 drug appearance shown in Extended Data Fig. 5a–h, including replicates used for quantification. [file 42255_2023_931_MOESM2_ESM.zip › Raw Data Extended Data Figure 5/07-whole brain images/20-M-KO-Veh-HYPO.tif]

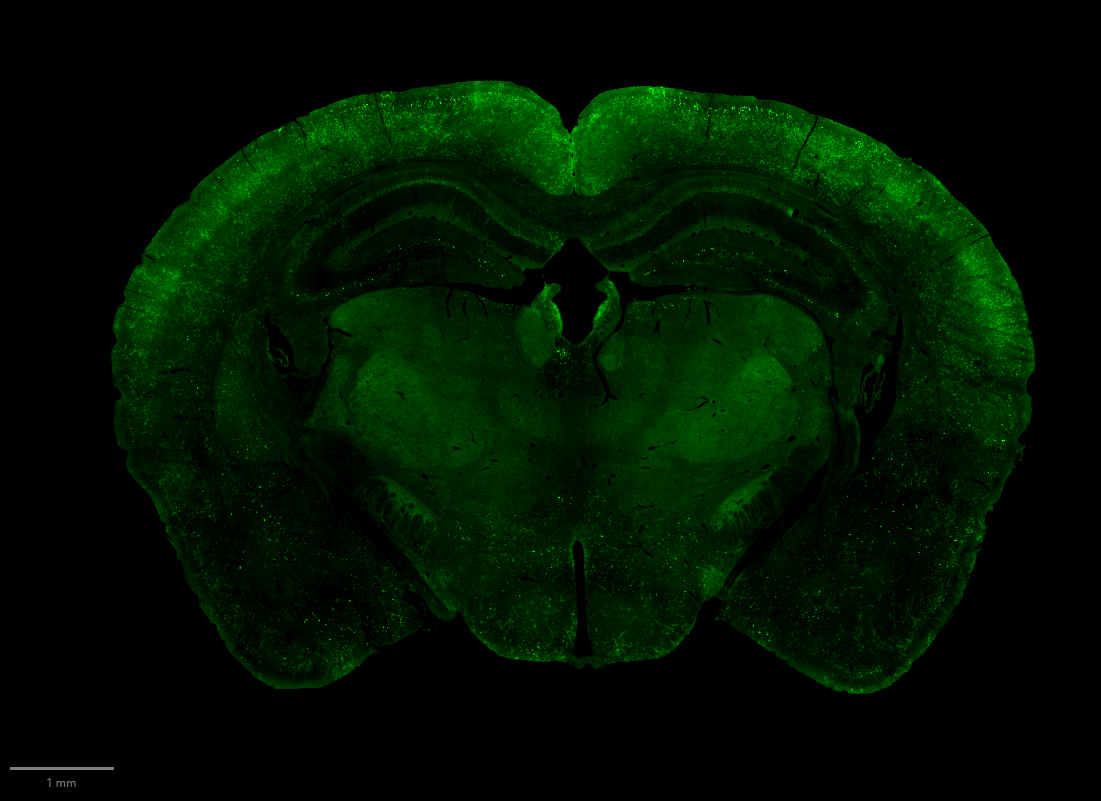

Supplement: Supplementary file 2 — Original pictures of cFos and Cy5 drug appearance shown in Extended Data Fig. 5a–h, including replicates used for quantification. [file 42255_2023_931_MOESM2_ESM.zip › Raw Data Extended Data Figure 5/07-whole brain images/64-M-WT-Veh-HYPO.tif]

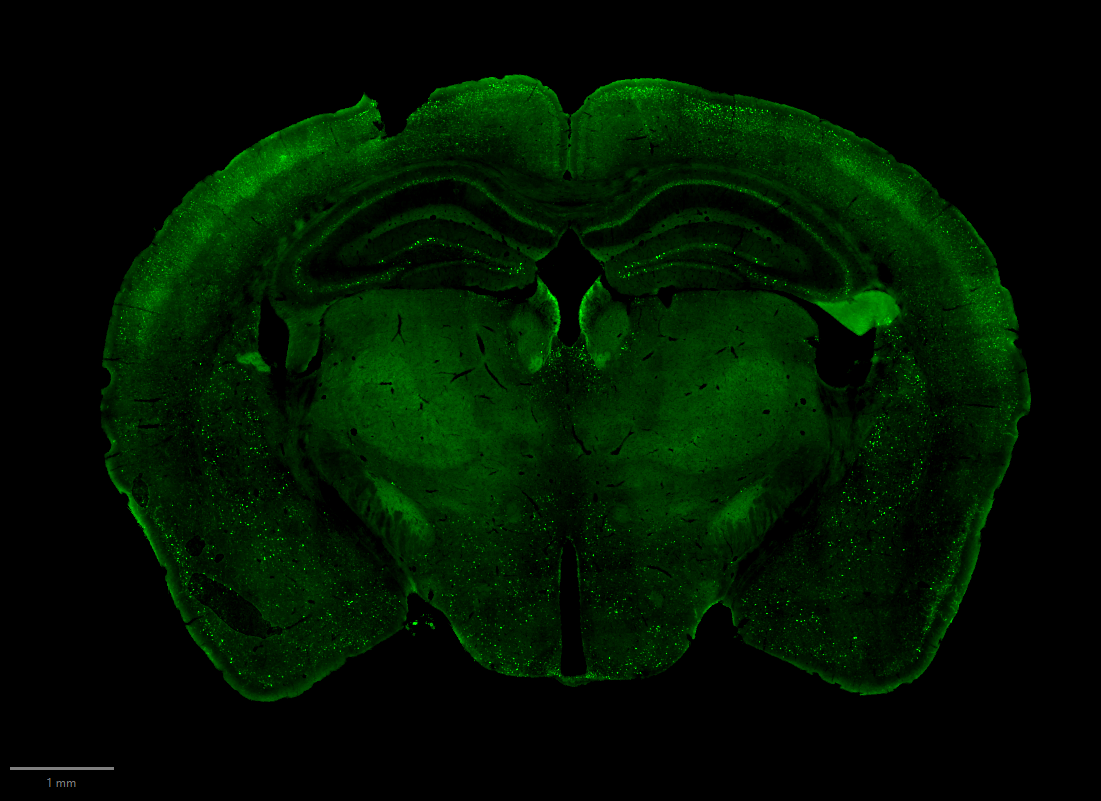

Supplement: Supplementary file 2 — Original pictures of cFos and Cy5 drug appearance shown in Extended Data Fig. 5a–h, including replicates used for quantification. [file 42255_2023_931_MOESM2_ESM.zip › Raw Data Extended Data Figure 5/07-whole brain images/53-M-WT-GIP-HYPO.tif]

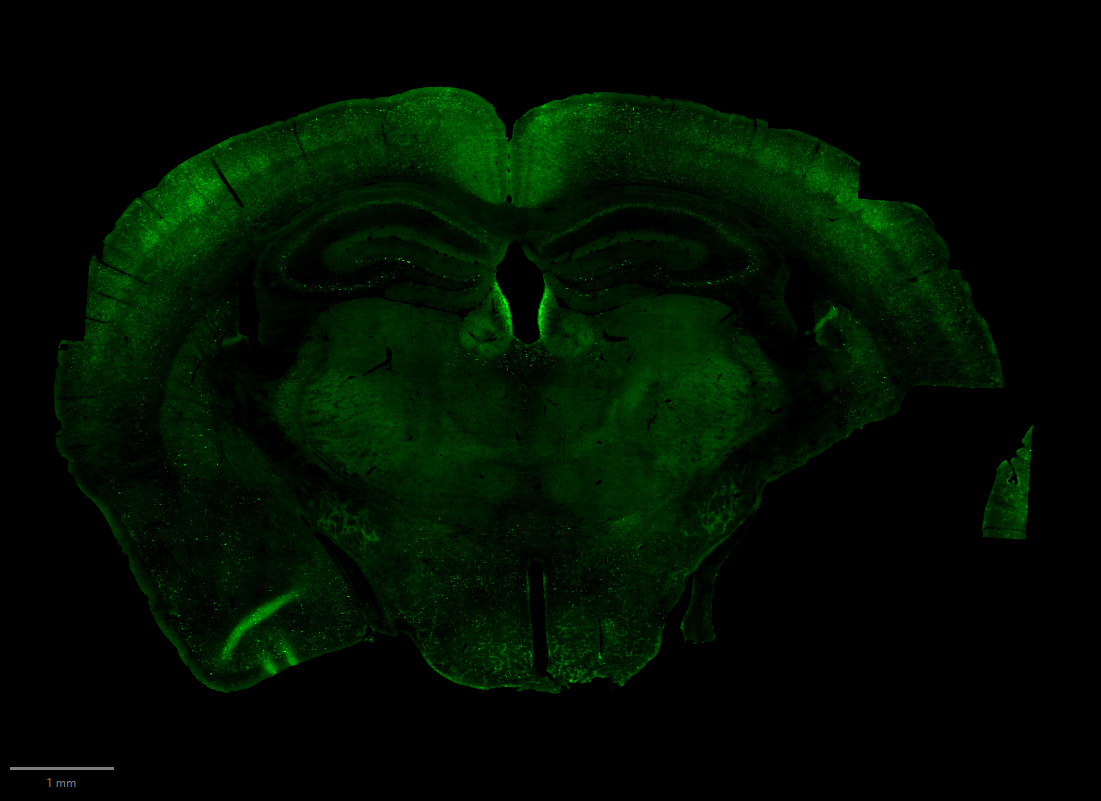

Supplement: Supplementary file 2 — Original pictures of cFos and Cy5 drug appearance shown in Extended Data Fig. 5a–h, including replicates used for quantification. [file 42255_2023_931_MOESM2_ESM.zip › Raw Data Extended Data Figure 5/07-whole brain images/65-M-WT-Veh-HYPO.tif]

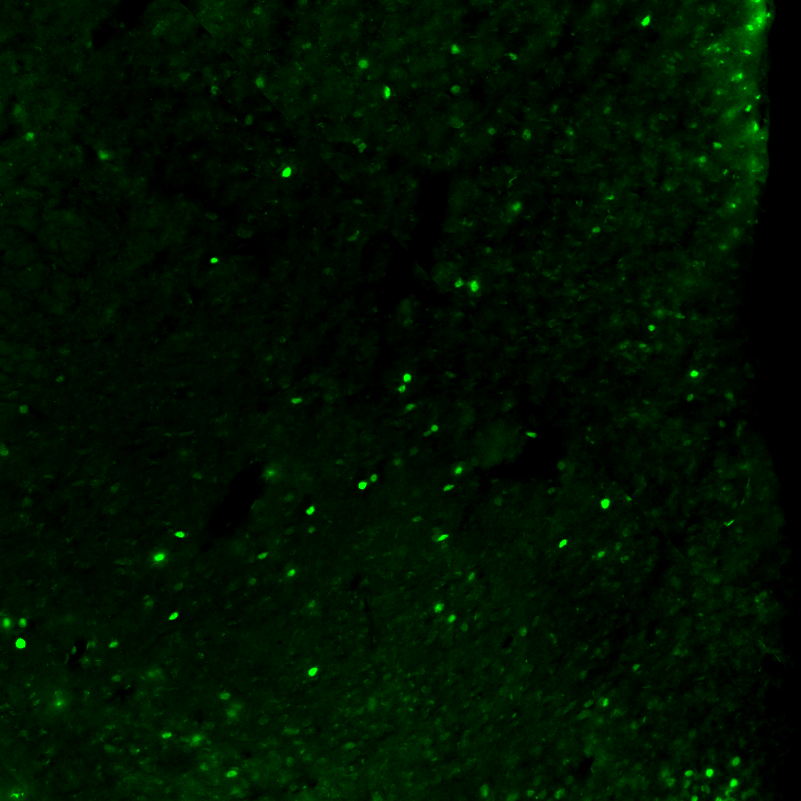

Supplement: Supplementary file 2 — Original pictures of cFos and Cy5 drug appearance shown in Extended Data Fig. 5a–h, including replicates used for quantification. [file 42255_2023_931_MOESM2_ESM.zip › Raw Data Extended Data Figure 5/03-VMH/2-M-KO-GIP-HYPO2.tif]

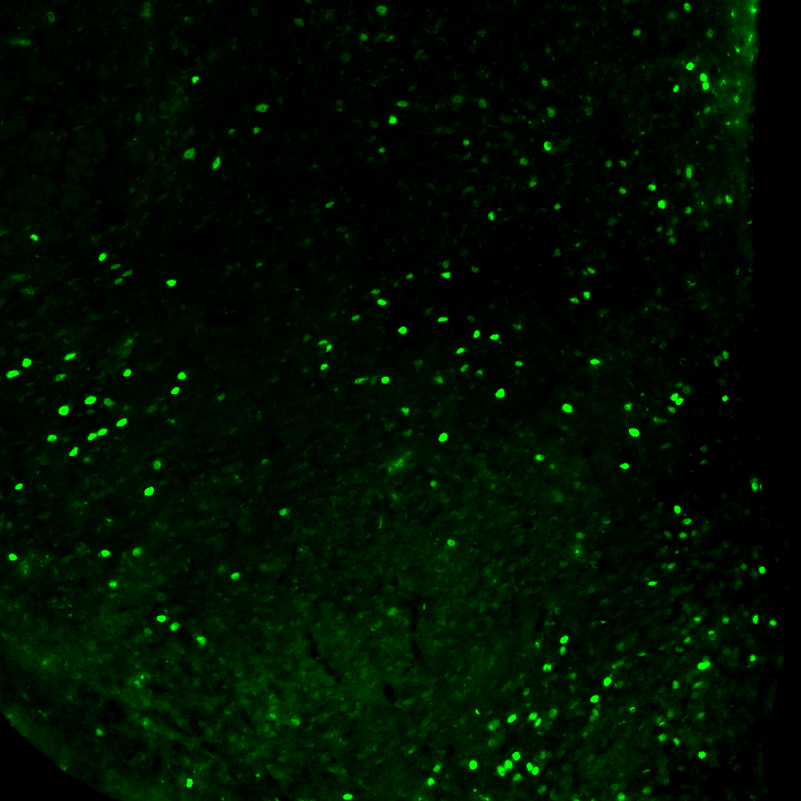

Supplement: Supplementary file 2 — Original pictures of cFos and Cy5 drug appearance shown in Extended Data Fig. 5a–h, including replicates used for quantification. [file 42255_2023_931_MOESM2_ESM.zip › Raw Data Extended Data Figure 5/03-VMH/46-M-WT-GIP-HYPO.tif]

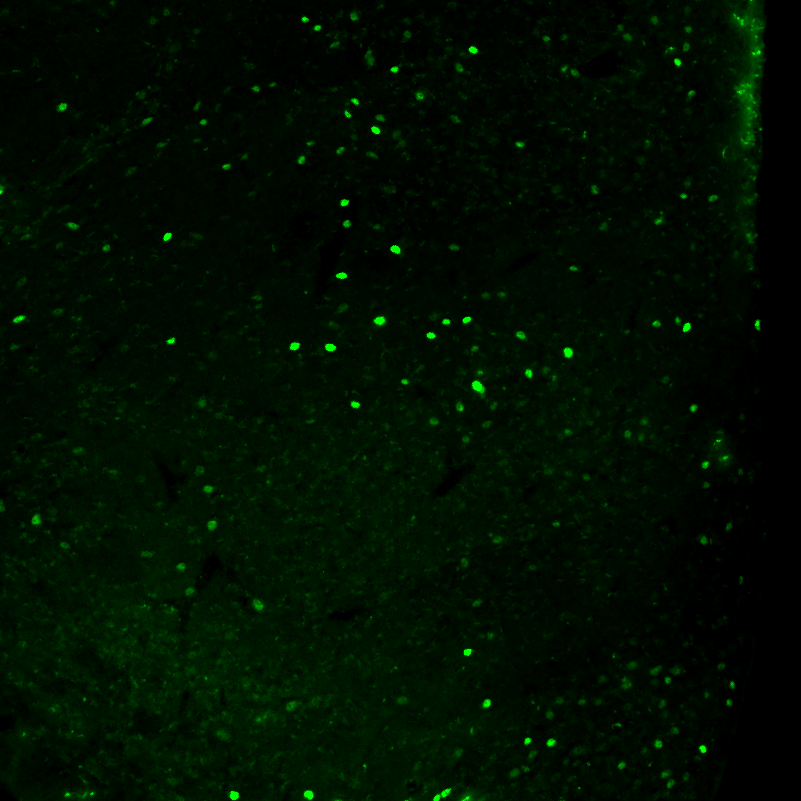

Supplement: Supplementary file 2 — Original pictures of cFos and Cy5 drug appearance shown in Extended Data Fig. 5a–h, including replicates used for quantification. [file 42255_2023_931_MOESM2_ESM.zip › Raw Data Extended Data Figure 5/03-VMH/3-M-KO-GIP-HYPO.tif]

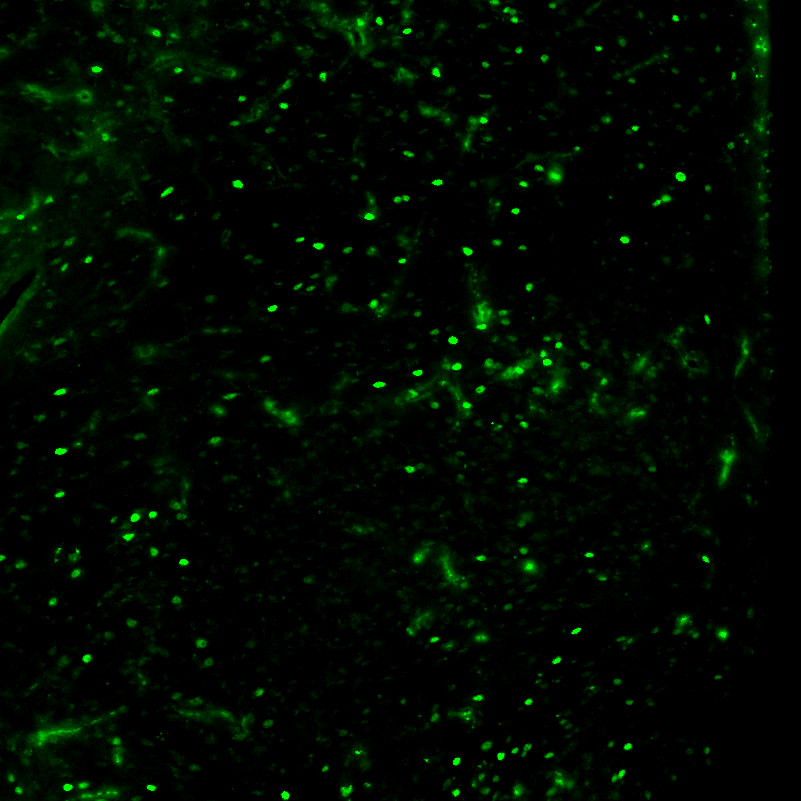

Supplement: Supplementary file 2 — Original pictures of cFos and Cy5 drug appearance shown in Extended Data Fig. 5a–h, including replicates used for quantification. [file 42255_2023_931_MOESM2_ESM.zip › Raw Data Extended Data Figure 5/03-VMH/36-M-KO-Veh-HYPO.tif]

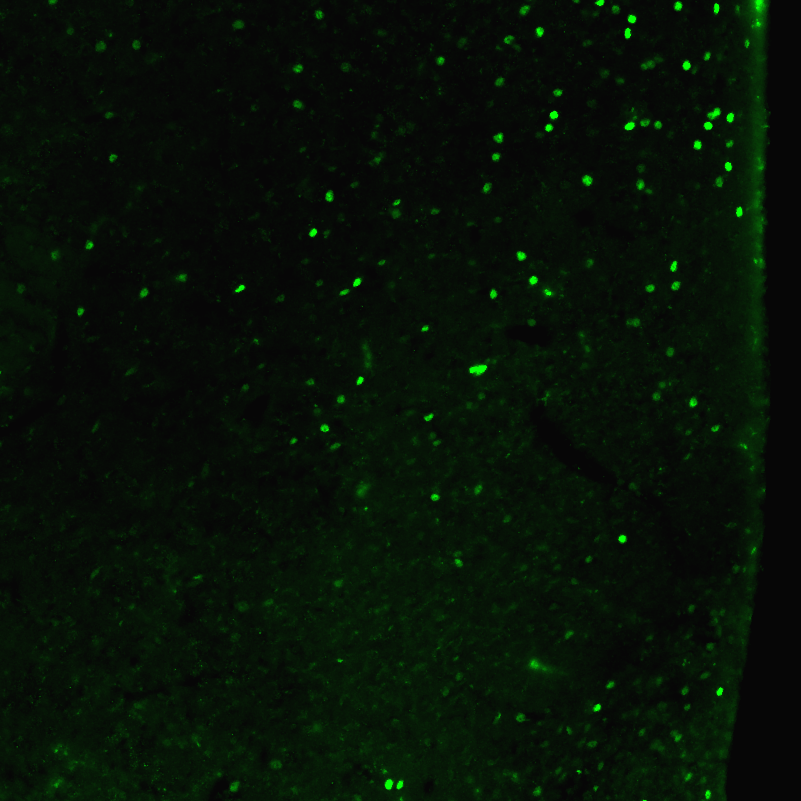

Supplement: Supplementary file 2 — Original pictures of cFos and Cy5 drug appearance shown in Extended Data Fig. 5a–h, including replicates used for quantification. [file 42255_2023_931_MOESM2_ESM.zip › Raw Data Extended Data Figure 5/03-VMH/44-M-WT-GIP-HYPO.tif]

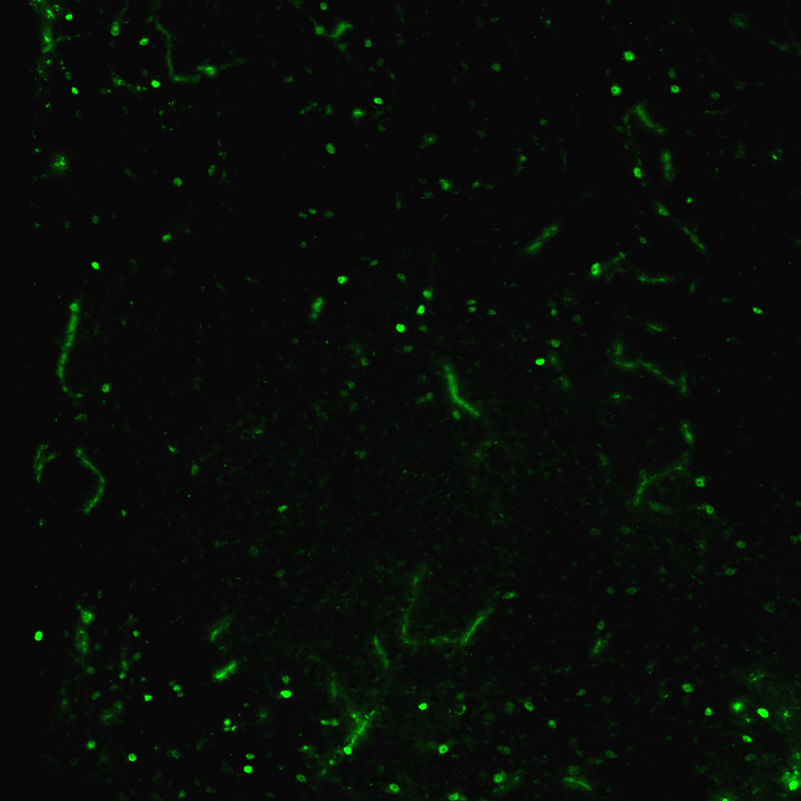

Supplement: Supplementary file 2 — Original pictures of cFos and Cy5 drug appearance shown in Extended Data Fig. 5a–h, including replicates used for quantification. [file 42255_2023_931_MOESM2_ESM.zip › Raw Data Extended Data Figure 5/03-VMH/66-M-WT-Veh-HYPO1.tif]

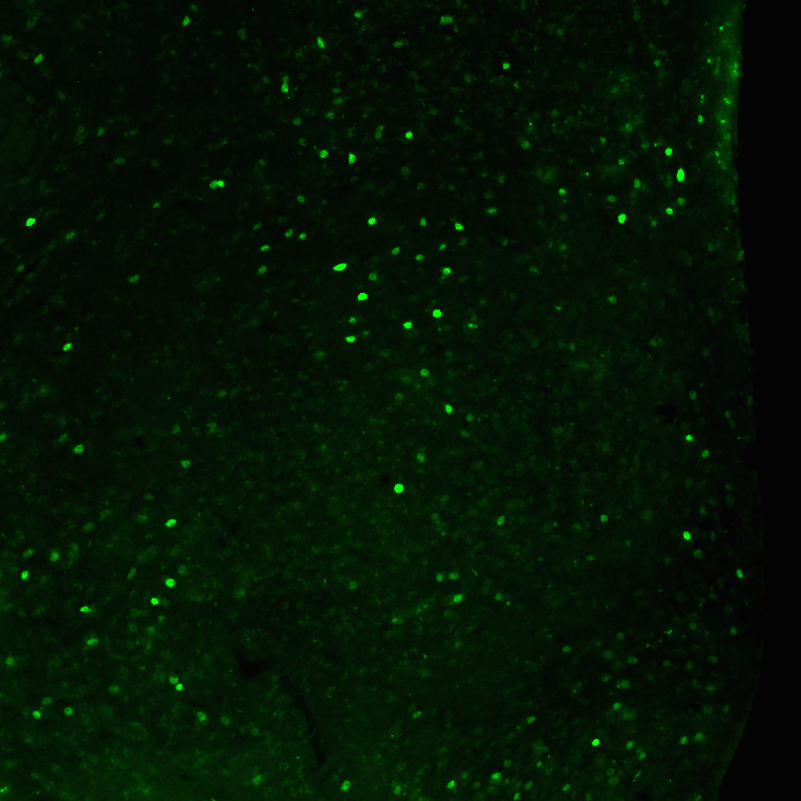

Supplement: Supplementary file 2 — Original pictures of cFos and Cy5 drug appearance shown in Extended Data Fig. 5a–h, including replicates used for quantification. [file 42255_2023_931_MOESM2_ESM.zip › Raw Data Extended Data Figure 5/03-VMH/1-M-KO-GIP-HYPO.tif]

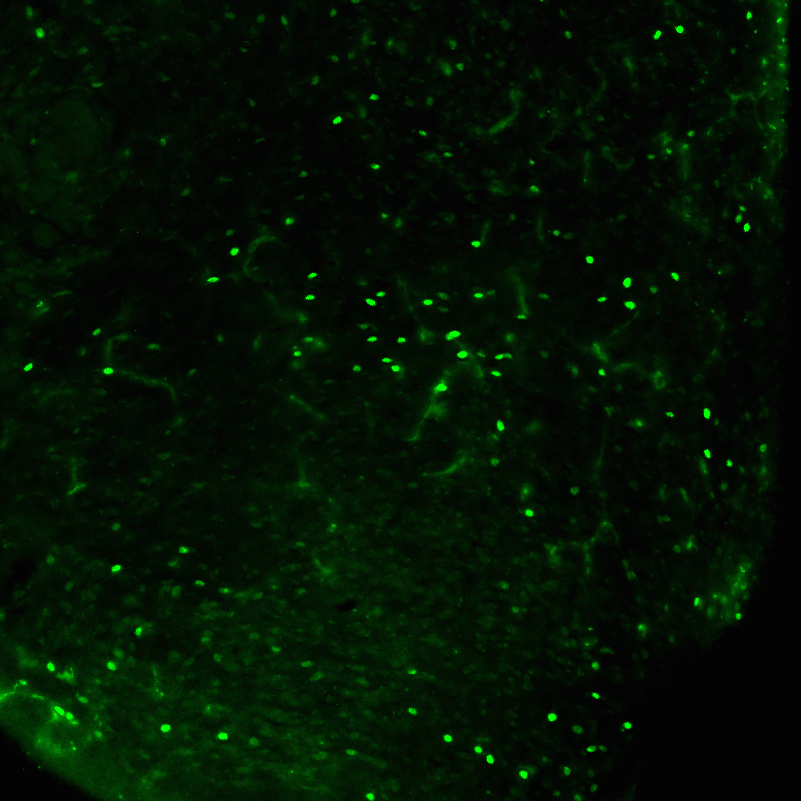

Supplement: Supplementary file 2 — Original pictures of cFos and Cy5 drug appearance shown in Extended Data Fig. 5a–h, including replicates used for quantification. [file 42255_2023_931_MOESM2_ESM.zip › Raw Data Extended Data Figure 5/03-VMH/37-M-KO-Veh-HYPO.tif]

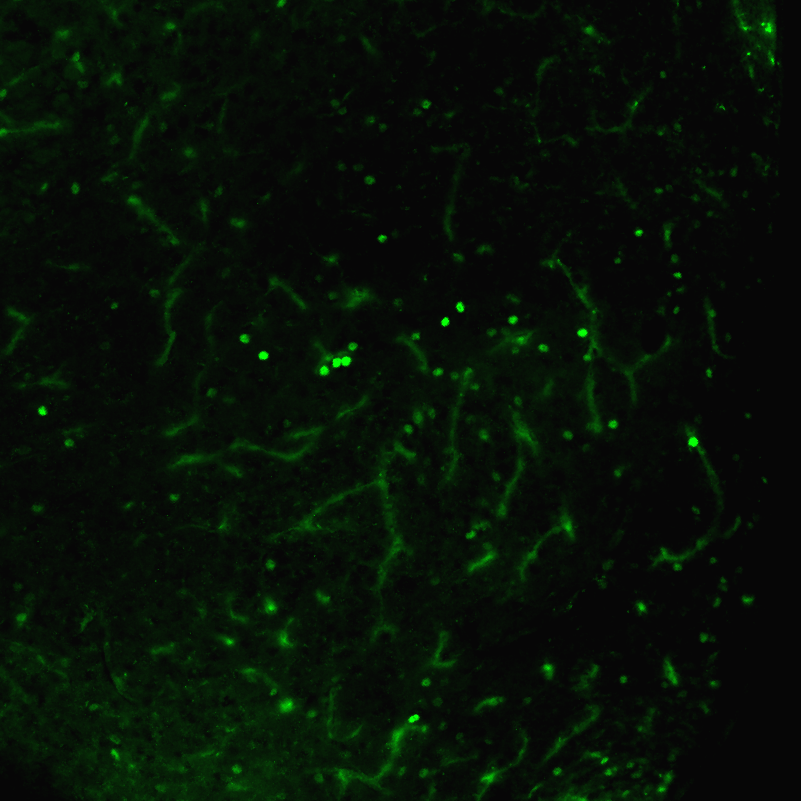

Supplement: Supplementary file 2 — Original pictures of cFos and Cy5 drug appearance shown in Extended Data Fig. 5a–h, including replicates used for quantification. [file 42255_2023_931_MOESM2_ESM.zip › Raw Data Extended Data Figure 5/03-VMH/55-M-WT-GIP-HYPO.tif]

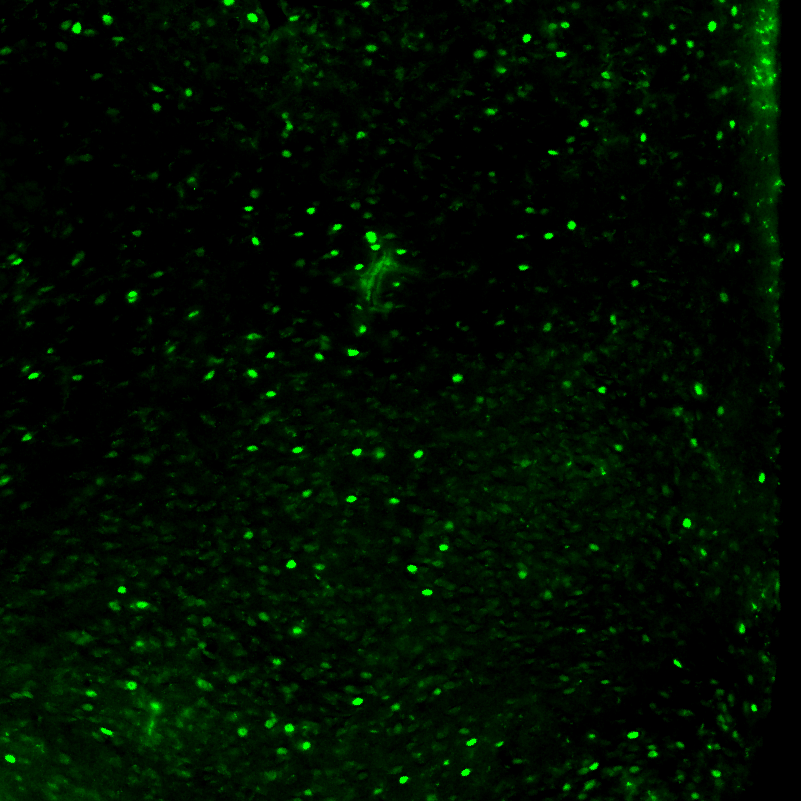

Supplement: Supplementary file 2 — Original pictures of cFos and Cy5 drug appearance shown in Extended Data Fig. 5a–h, including replicates used for quantification. [file 42255_2023_931_MOESM2_ESM.zip › Raw Data Extended Data Figure 5/03-VMH/14-M-KO-GIP-HYPO.tif]

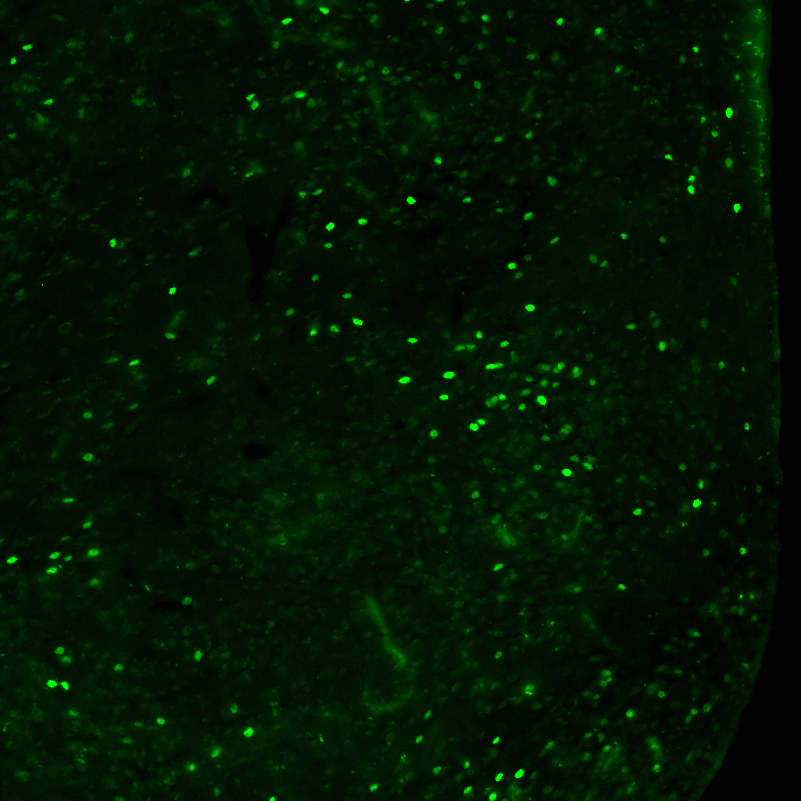

Supplement: Supplementary file 2 — Original pictures of cFos and Cy5 drug appearance shown in Extended Data Fig. 5a–h, including replicates used for quantification. [file 42255_2023_931_MOESM2_ESM.zip › Raw Data Extended Data Figure 5/03-VMH/17-M-KO-Veh-HYPO.tif]

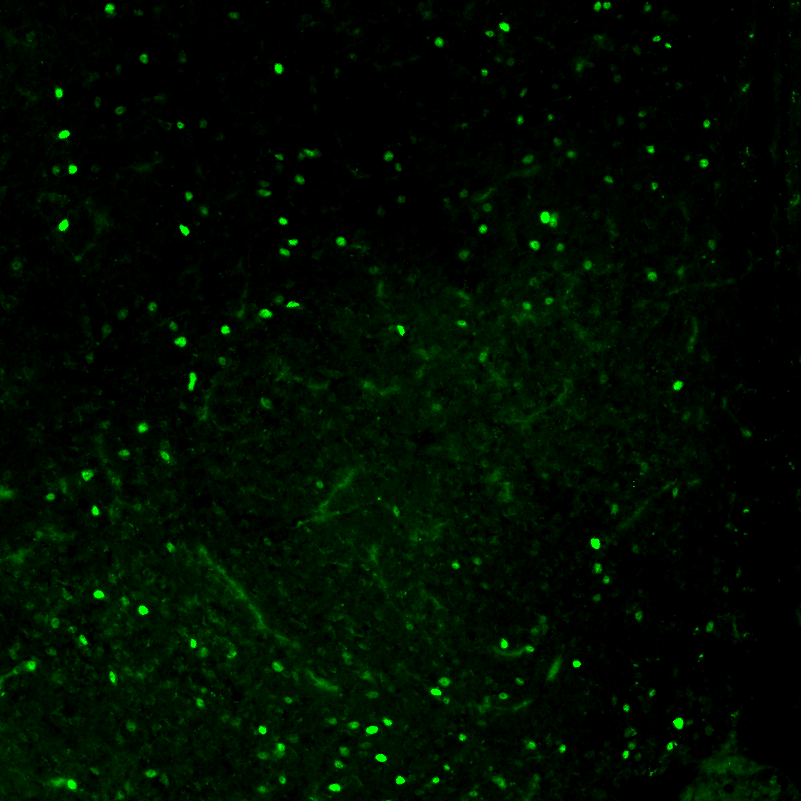

Supplement: Supplementary file 2 — Original pictures of cFos and Cy5 drug appearance shown in Extended Data Fig. 5a–h, including replicates used for quantification. [file 42255_2023_931_MOESM2_ESM.zip › Raw Data Extended Data Figure 5/03-VMH/67-M-WT-Veh-HYPO.tif]

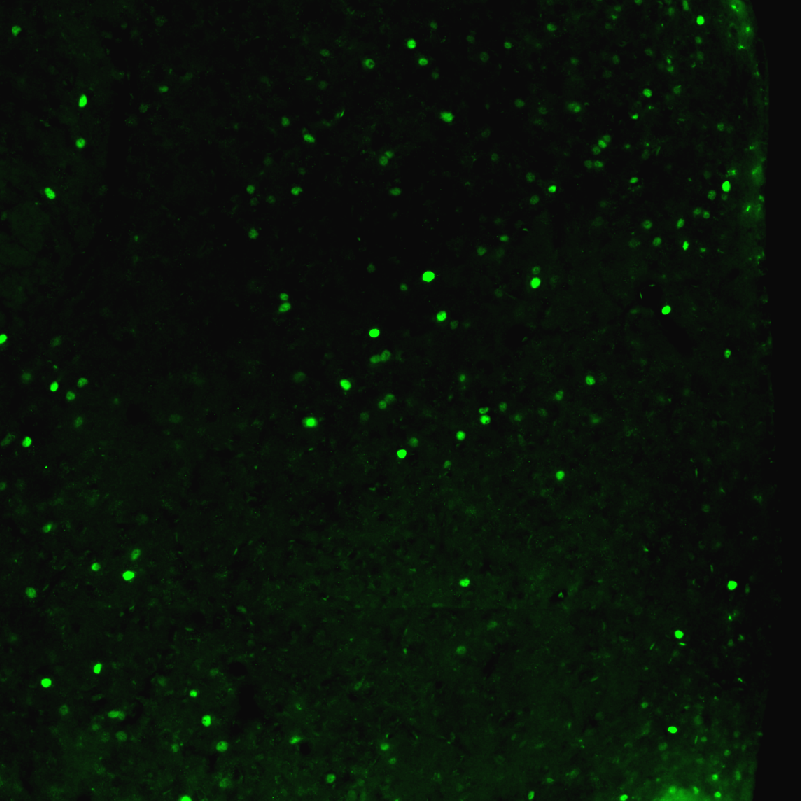

Supplement: Supplementary file 2 — Original pictures of cFos and Cy5 drug appearance shown in Extended Data Fig. 5a–h, including replicates used for quantification. [file 42255_2023_931_MOESM2_ESM.zip › Raw Data Extended Data Figure 5/03-VMH/54-M-WT-GIP-HYPO.tif]

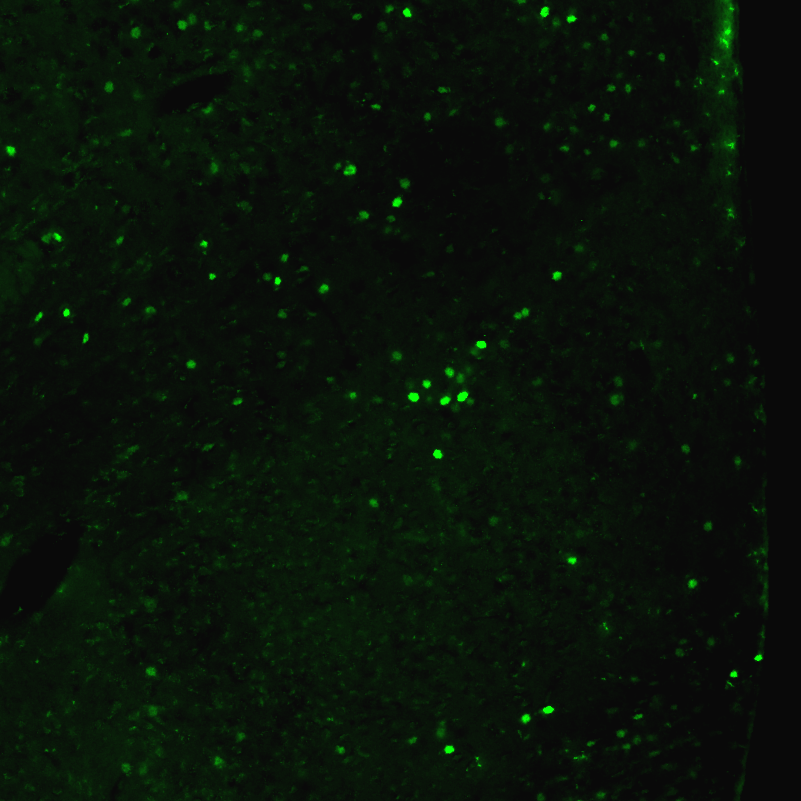

Supplement: Supplementary file 2 — Original pictures of cFos and Cy5 drug appearance shown in Extended Data Fig. 5a–h, including replicates used for quantification. [file 42255_2023_931_MOESM2_ESM.zip › Raw Data Extended Data Figure 5/03-VMH/15-M-KO-GIP-HYPO.tif]

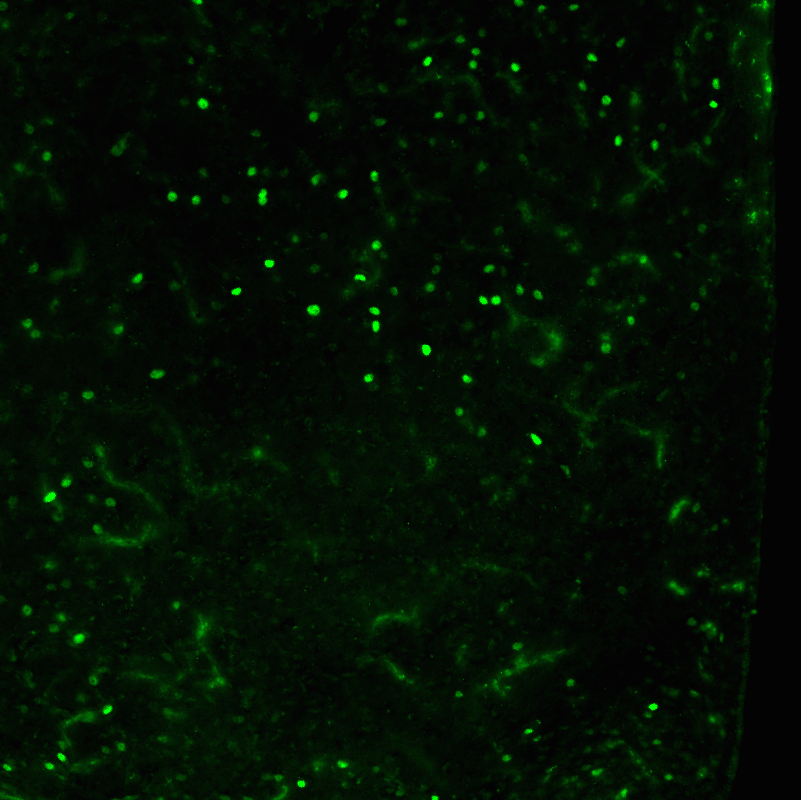

Supplement: Supplementary file 2 — Original pictures of cFos and Cy5 drug appearance shown in Extended Data Fig. 5a–h, including replicates used for quantification. [file 42255_2023_931_MOESM2_ESM.zip › Raw Data Extended Data Figure 5/03-VMH/20-M-KO-Veh-HYPO.tif]

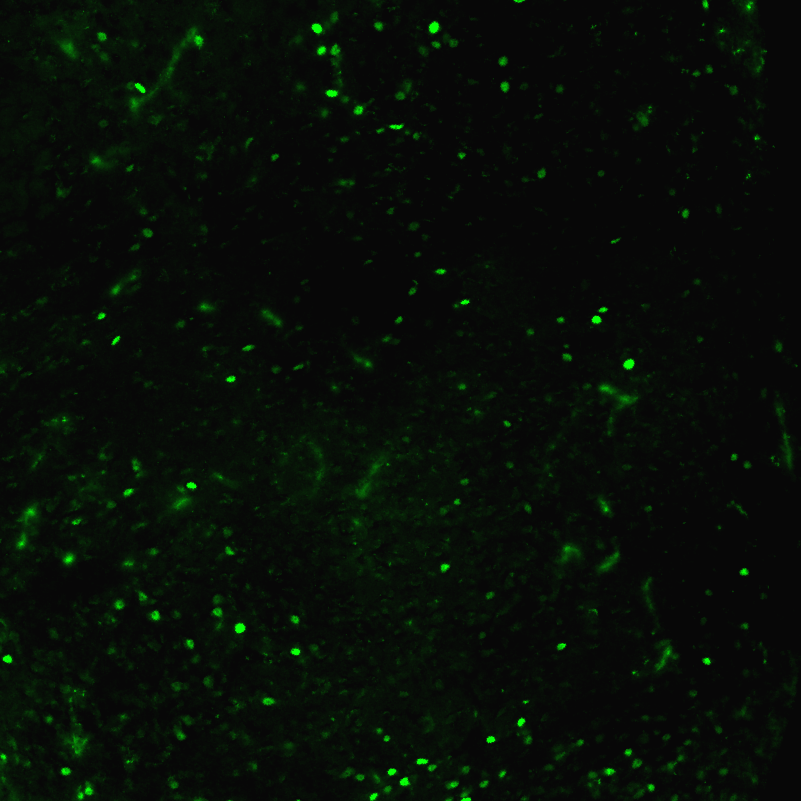

Supplement: Supplementary file 2 — Original pictures of cFos and Cy5 drug appearance shown in Extended Data Fig. 5a–h, including replicates used for quantification. [file 42255_2023_931_MOESM2_ESM.zip › Raw Data Extended Data Figure 5/03-VMH/64-M-WT-Veh-HYPO.tif]

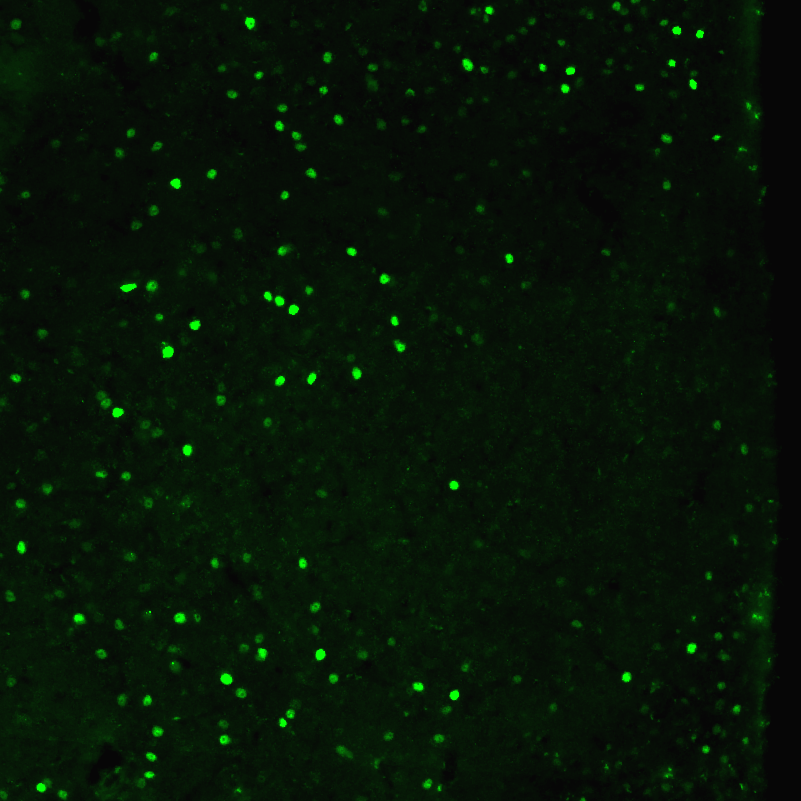

Supplement: Supplementary file 2 — Original pictures of cFos and Cy5 drug appearance shown in Extended Data Fig. 5a–h, including replicates used for quantification. [file 42255_2023_931_MOESM2_ESM.zip › Raw Data Extended Data Figure 5/03-VMH/53-M-WT-GIP-HYPO.tif]

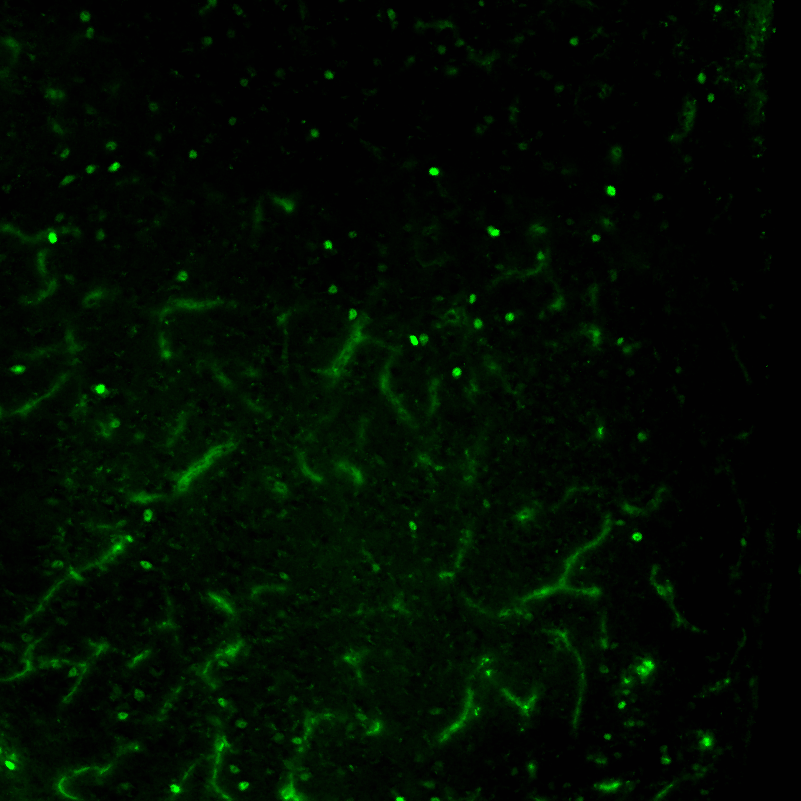

Supplement: Supplementary file 2 — Original pictures of cFos and Cy5 drug appearance shown in Extended Data Fig. 5a–h, including replicates used for quantification. [file 42255_2023_931_MOESM2_ESM.zip › Raw Data Extended Data Figure 5/03-VMH/65-M-WT-Veh-HYPO.tif]

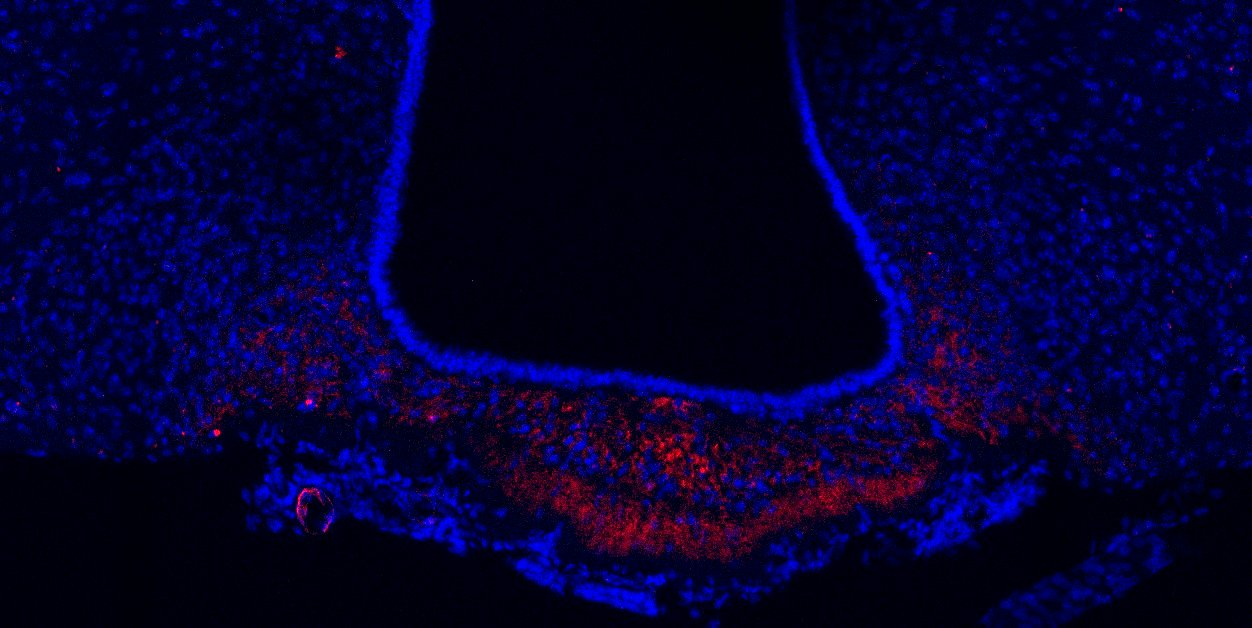

Supplement: Supplementary file 2 — Original pictures of cFos and Cy5 drug appearance shown in Extended Data Fig. 5a–h, including replicates used for quantification. [file 42255_2023_931_MOESM2_ESM.zip › Raw Data Extended Data Figure 5/06-GIPcy5 in ME/1-M-KO-GIP-Hypo2 Merged-1.jpg]

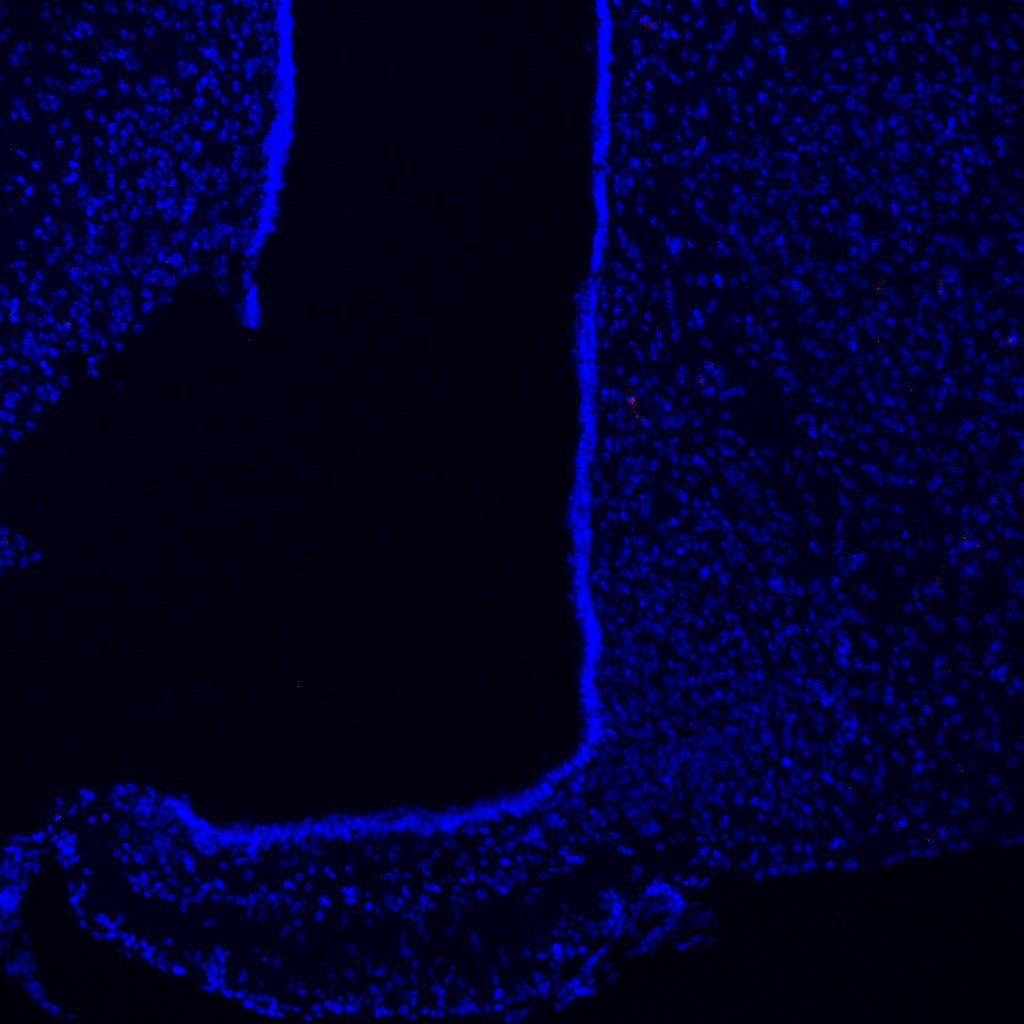

Supplement: Supplementary file 2 — Original pictures of cFos and Cy5 drug appearance shown in Extended Data Fig. 5a–h, including replicates used for quantification. [file 42255_2023_931_MOESM2_ESM.zip › Raw Data Extended Data Figure 5/06-GIPcy5 in ME/37-M-KO-Veh-Hypo.jpg]

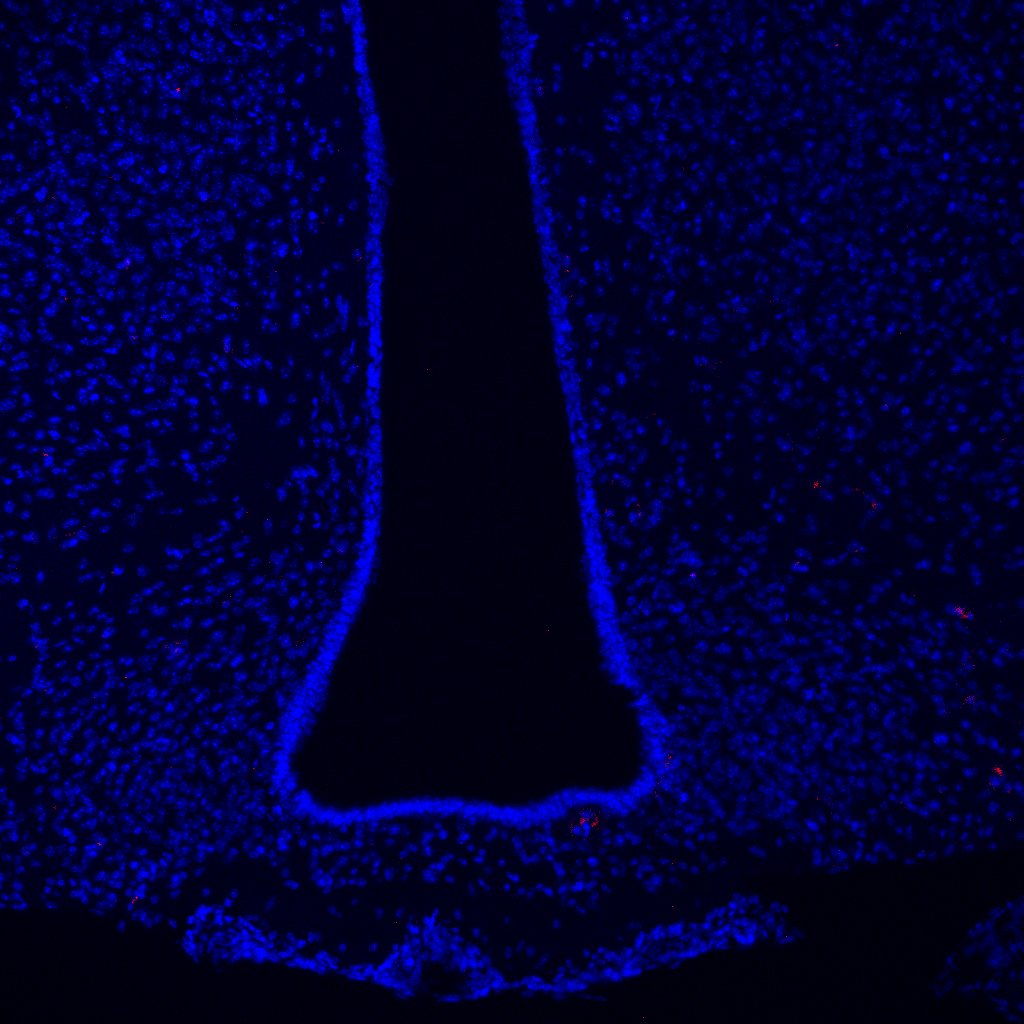

Supplement: Supplementary file 2 — Original pictures of cFos and Cy5 drug appearance shown in Extended Data Fig. 5a–h, including replicates used for quantification. [file 42255_2023_931_MOESM2_ESM.zip › Raw Data Extended Data Figure 5/06-GIPcy5 in ME/20-M-KO-Veh_Hypo.jpg]

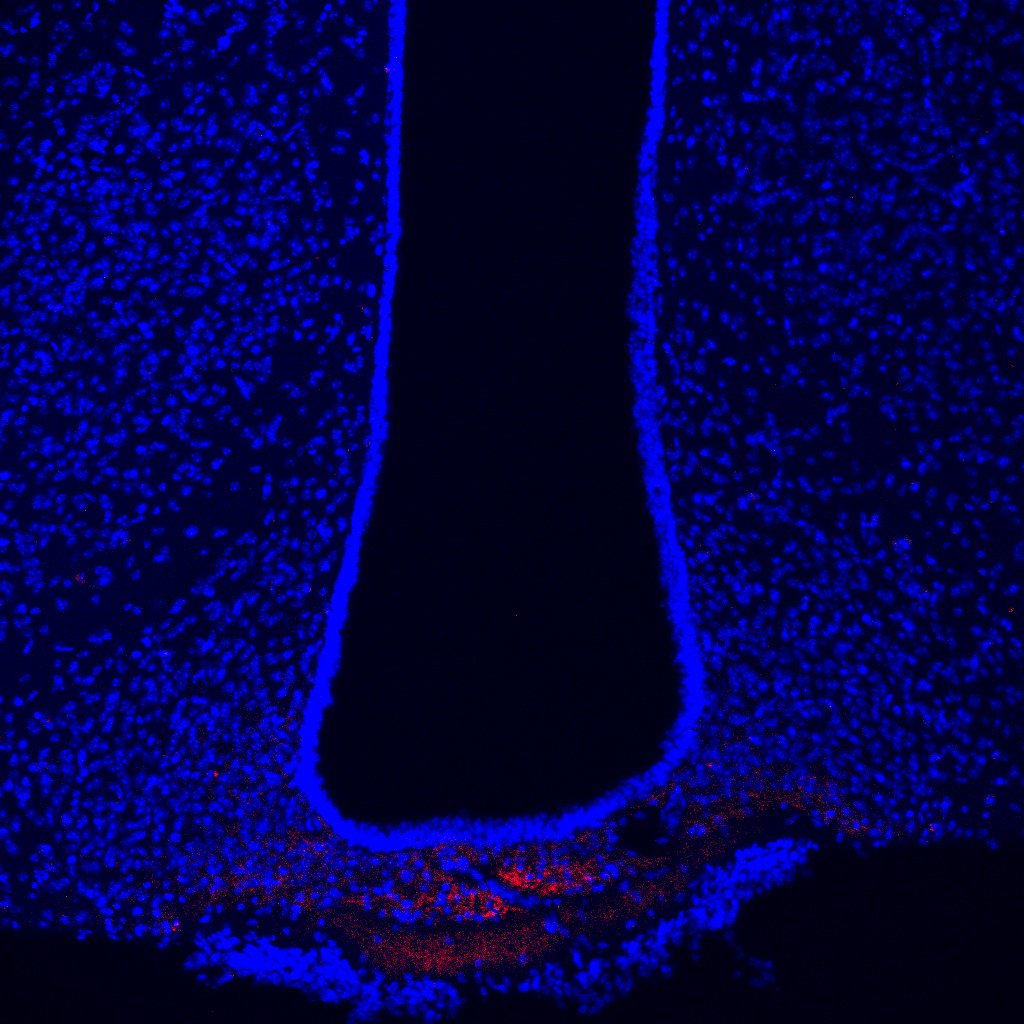

Supplement: Supplementary file 2 — Original pictures of cFos and Cy5 drug appearance shown in Extended Data Fig. 5a–h, including replicates used for quantification. [file 42255_2023_931_MOESM2_ESM.zip › Raw Data Extended Data Figure 5/06-GIPcy5 in ME/44-M-WT-GIP-Hypo.jpg]

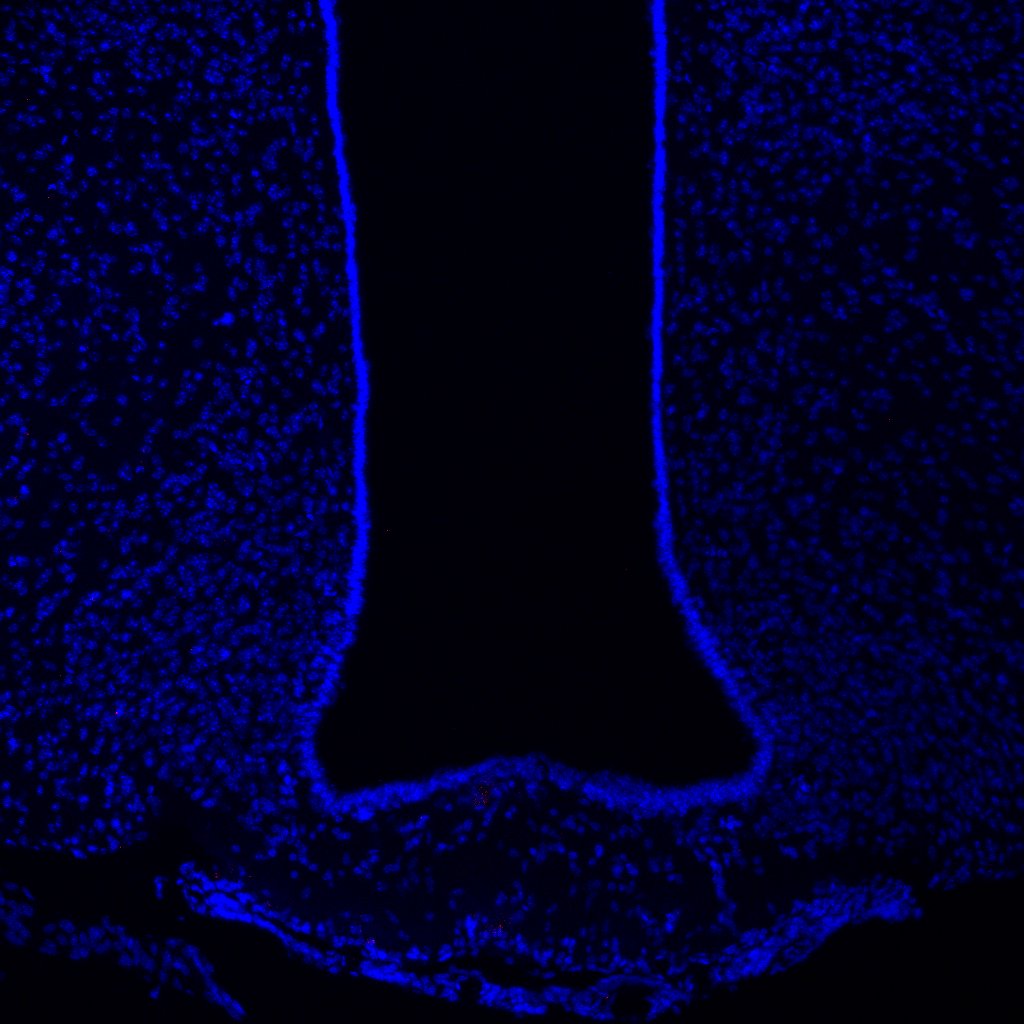

Supplement: Supplementary file 2 — Original pictures of cFos and Cy5 drug appearance shown in Extended Data Fig. 5a–h, including replicates used for quantification. [file 42255_2023_931_MOESM2_ESM.zip › Raw Data Extended Data Figure 5/06-GIPcy5 in ME/17-M-KO-Veh_hypo.jpg]

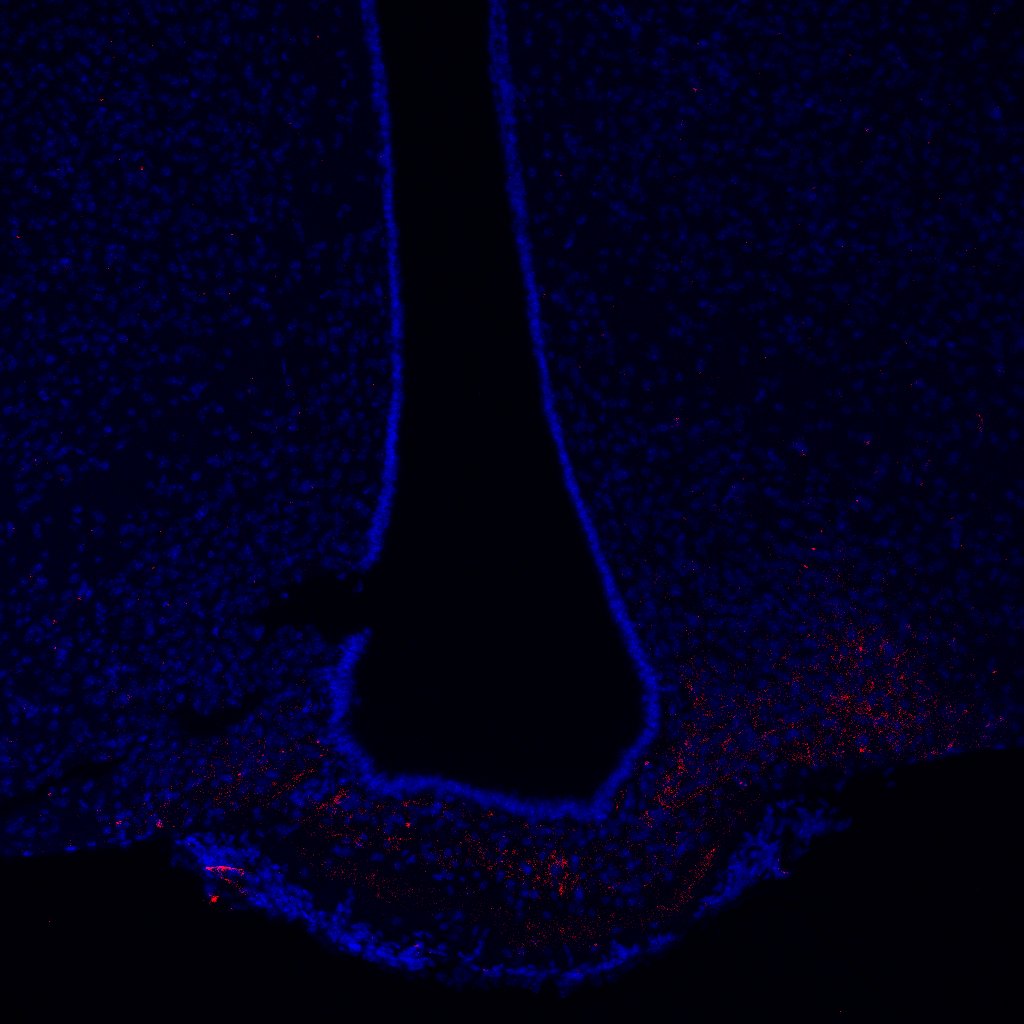

Supplement: Supplementary file 2 — Original pictures of cFos and Cy5 drug appearance shown in Extended Data Fig. 5a–h, including replicates used for quantification. [file 42255_2023_931_MOESM2_ESM.zip › Raw Data Extended Data Figure 5/06-GIPcy5 in ME/3-M-KO-GIP-Hypo.jpg]

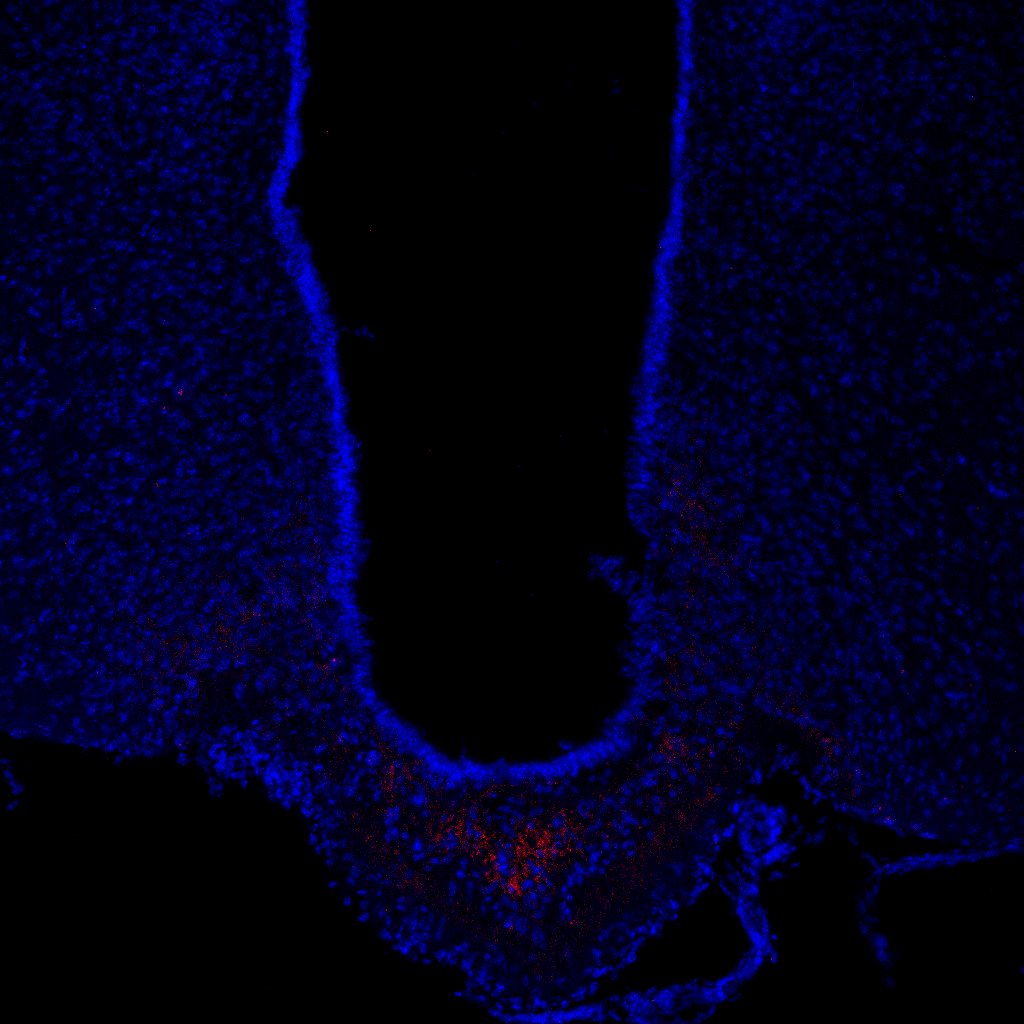

Supplement: Supplementary file 2 — Original pictures of cFos and Cy5 drug appearance shown in Extended Data Fig. 5a–h, including replicates used for quantification. [file 42255_2023_931_MOESM2_ESM.zip › Raw Data Extended Data Figure 5/06-GIPcy5 in ME/2-M-KO-GIP-Hypo2.jpg]

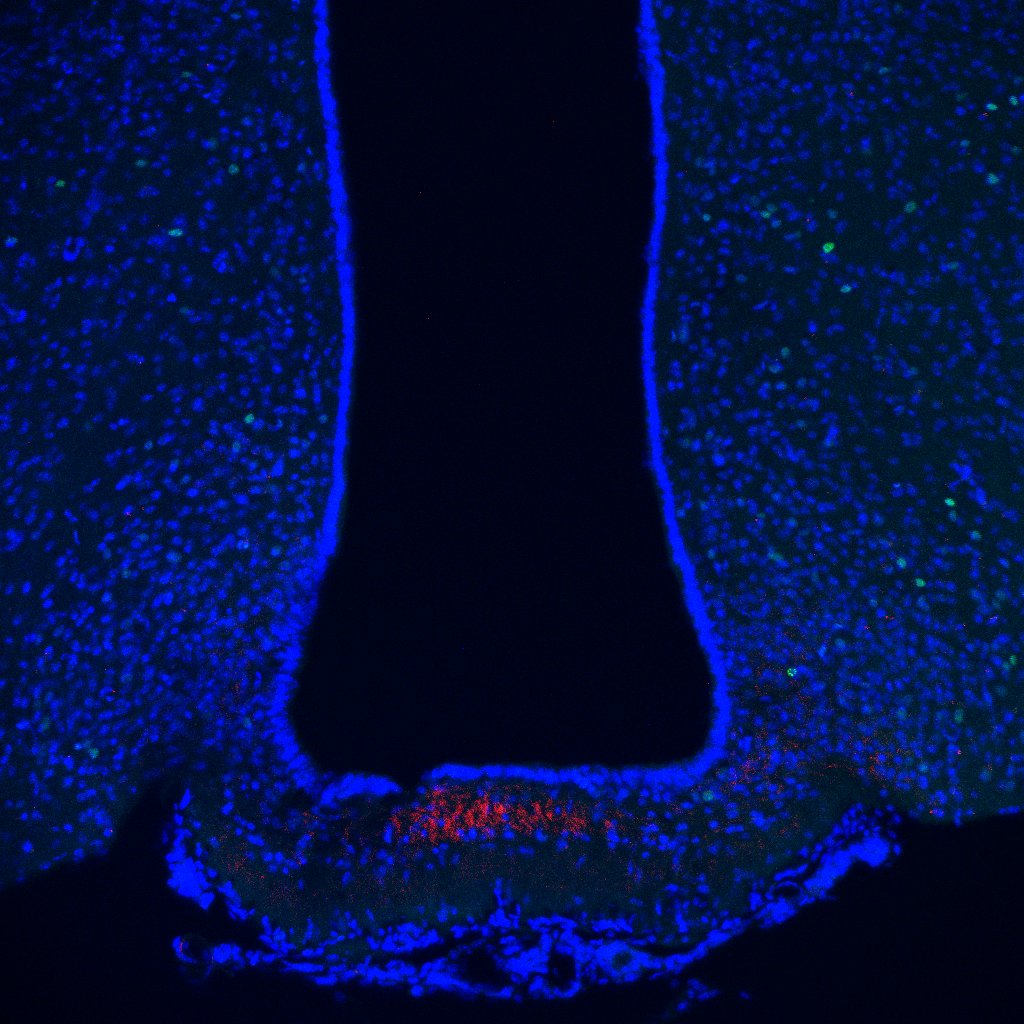

Supplement: Supplementary file 2 — Original pictures of cFos and Cy5 drug appearance shown in Extended Data Fig. 5a–h, including replicates used for quantification. [file 42255_2023_931_MOESM2_ESM.zip › Raw Data Extended Data Figure 5/06-GIPcy5 in ME/46-M-WT-GIP-Hypo.jpg]

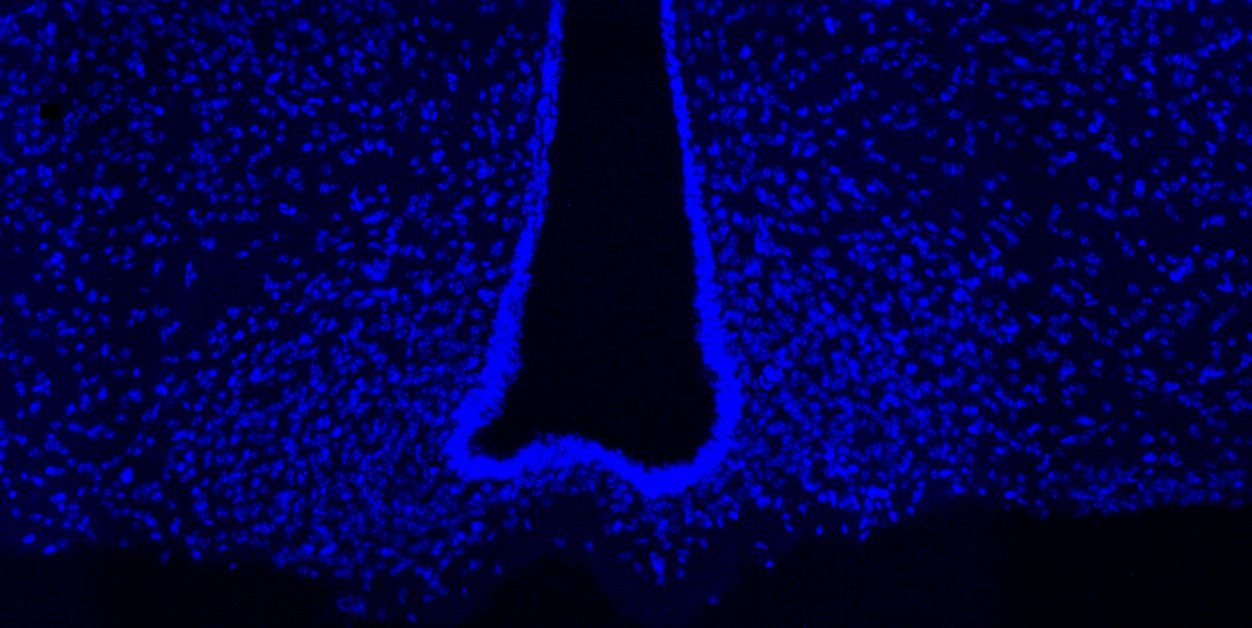

Supplement: Supplementary file 2 — Original pictures of cFos and Cy5 drug appearance shown in Extended Data Fig. 5a–h, including replicates used for quantification. [file 42255_2023_931_MOESM2_ESM.zip › Raw Data Extended Data Figure 5/06-GIPcy5 in ME/64-M-WT-Veh-Hypo Merged-1.jpg]

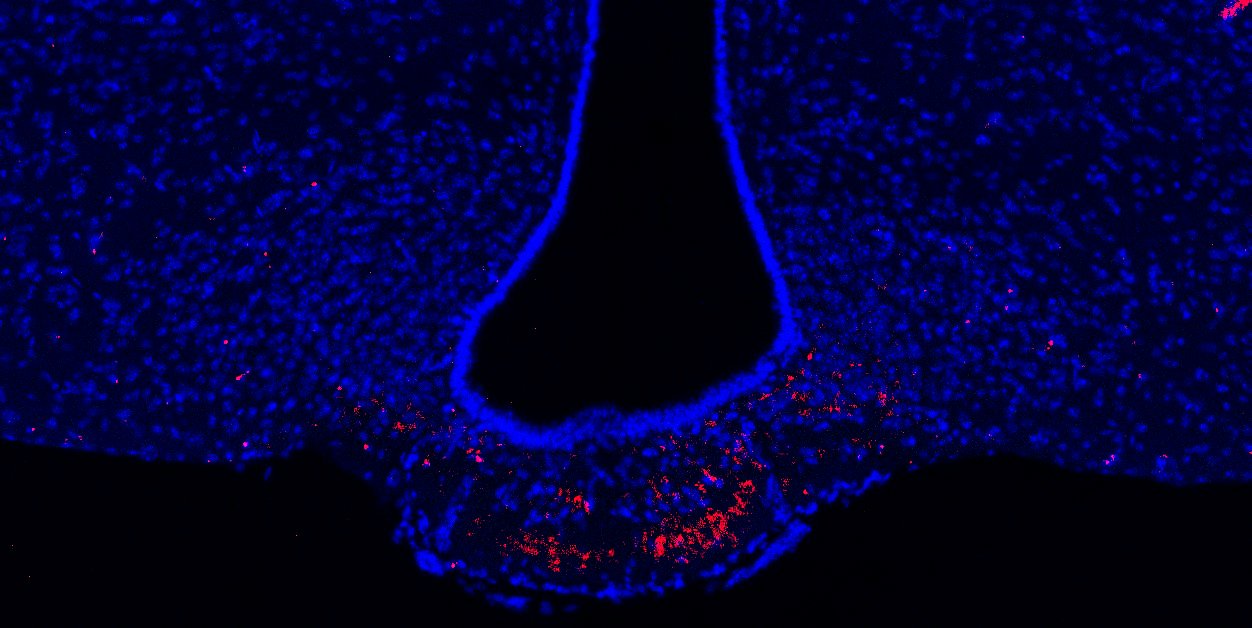

Supplement: Supplementary file 2 — Original pictures of cFos and Cy5 drug appearance shown in Extended Data Fig. 5a–h, including replicates used for quantification. [file 42255_2023_931_MOESM2_ESM.zip › Raw Data Extended Data Figure 5/06-GIPcy5 in ME/14-M-KO-GIP-Hypo Merged-1.jpg]

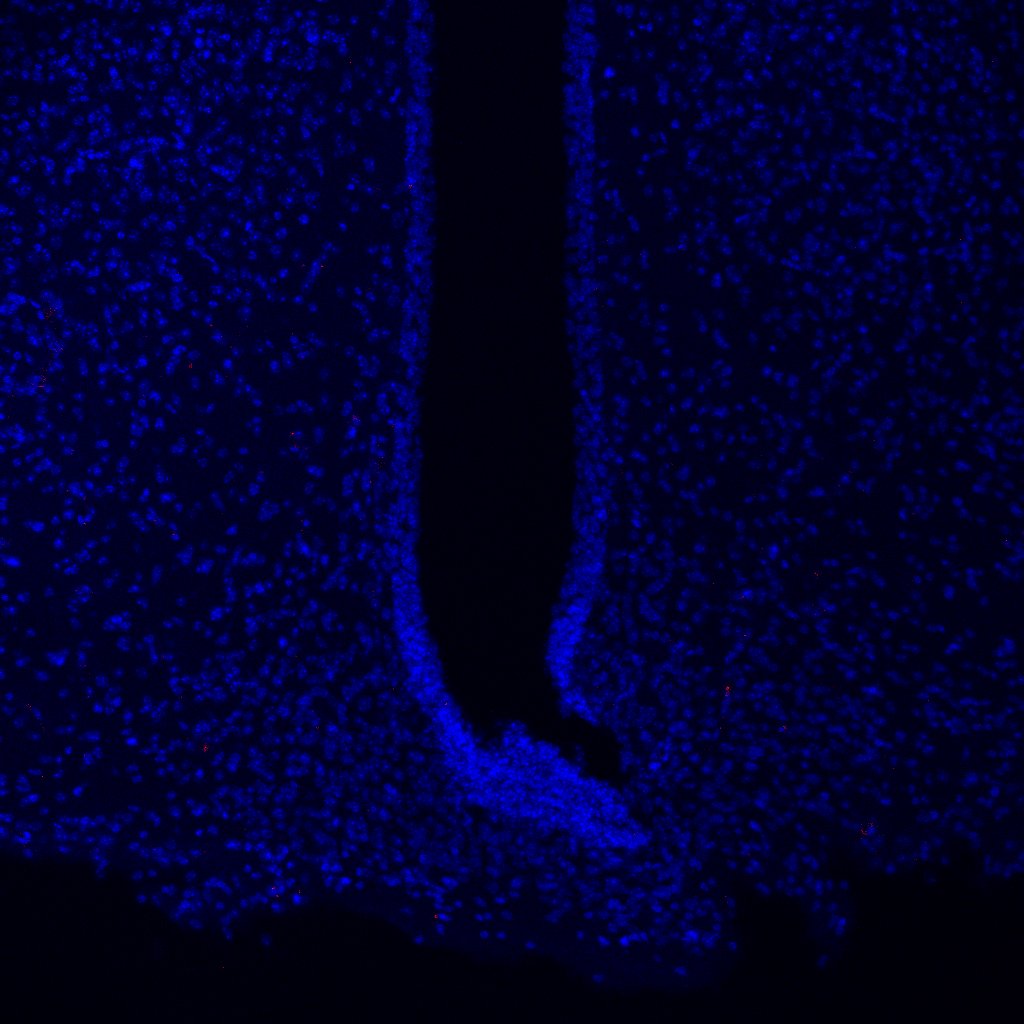

Supplement: Supplementary file 2 — Original pictures of cFos and Cy5 drug appearance shown in Extended Data Fig. 5a–h, including replicates used for quantification. [file 42255_2023_931_MOESM2_ESM.zip › Raw Data Extended Data Figure 5/06-GIPcy5 in ME/65-M-WT-Veh-Hypo.jpg]

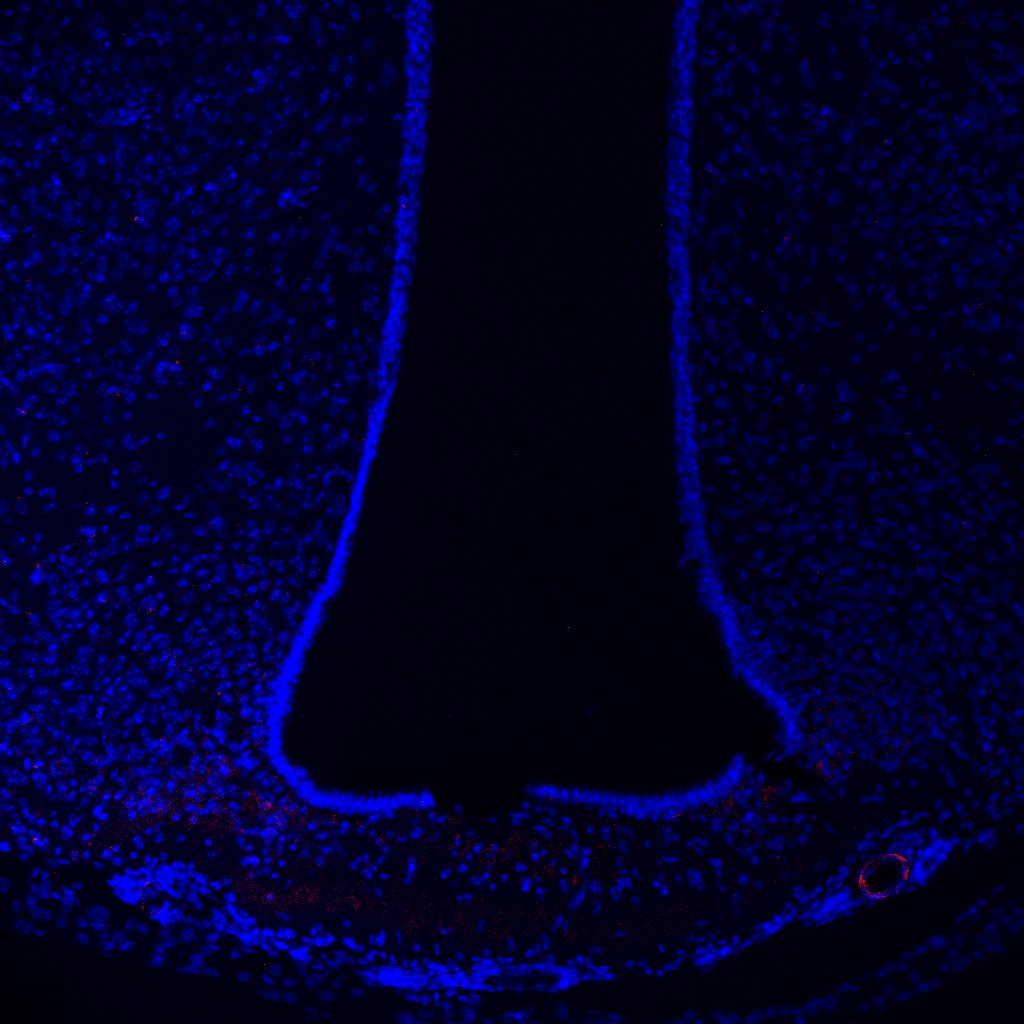

Supplement: Supplementary file 2 — Original pictures of cFos and Cy5 drug appearance shown in Extended Data Fig. 5a–h, including replicates used for quantification. [file 42255_2023_931_MOESM2_ESM.zip › Raw Data Extended Data Figure 5/06-GIPcy5 in ME/15-M-KO-GIP-Hypo.jpg]

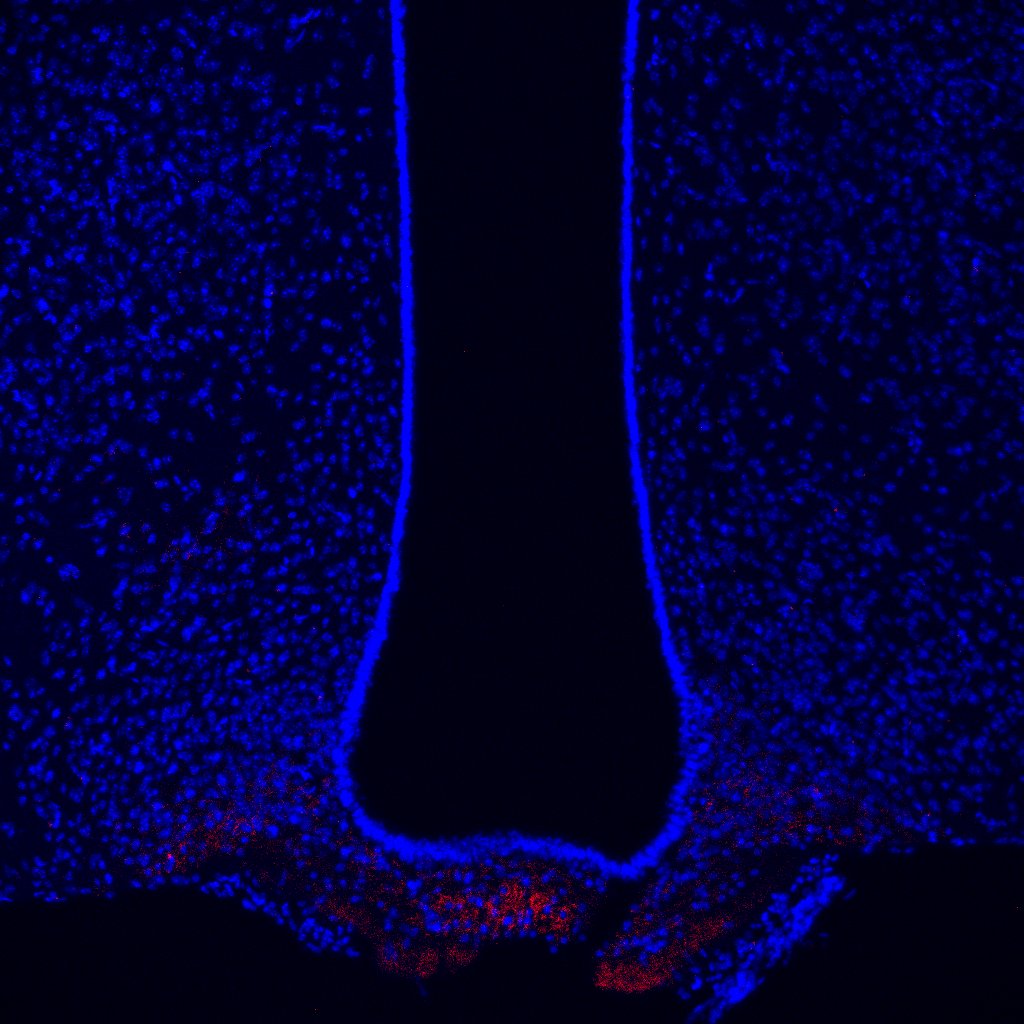

Supplement: Supplementary file 2 — Original pictures of cFos and Cy5 drug appearance shown in Extended Data Fig. 5a–h, including replicates used for quantification. [file 42255_2023_931_MOESM2_ESM.zip › Raw Data Extended Data Figure 5/06-GIPcy5 in ME/54-M-WT-GIP-Hypo.jpg]

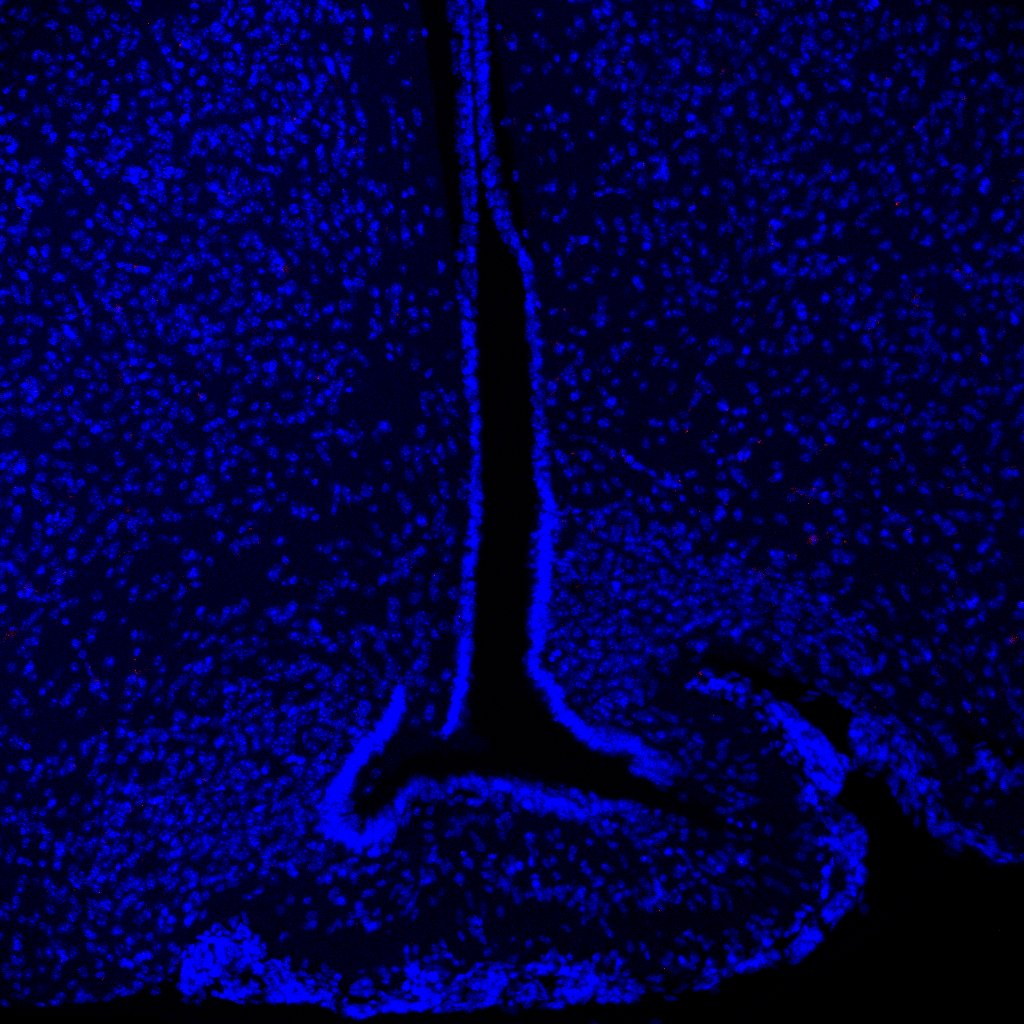

Supplement: Supplementary file 2 — Original pictures of cFos and Cy5 drug appearance shown in Extended Data Fig. 5a–h, including replicates used for quantification. [file 42255_2023_931_MOESM2_ESM.zip › Raw Data Extended Data Figure 5/06-GIPcy5 in ME/67-M-WT-Veh-Hypo.jpg]

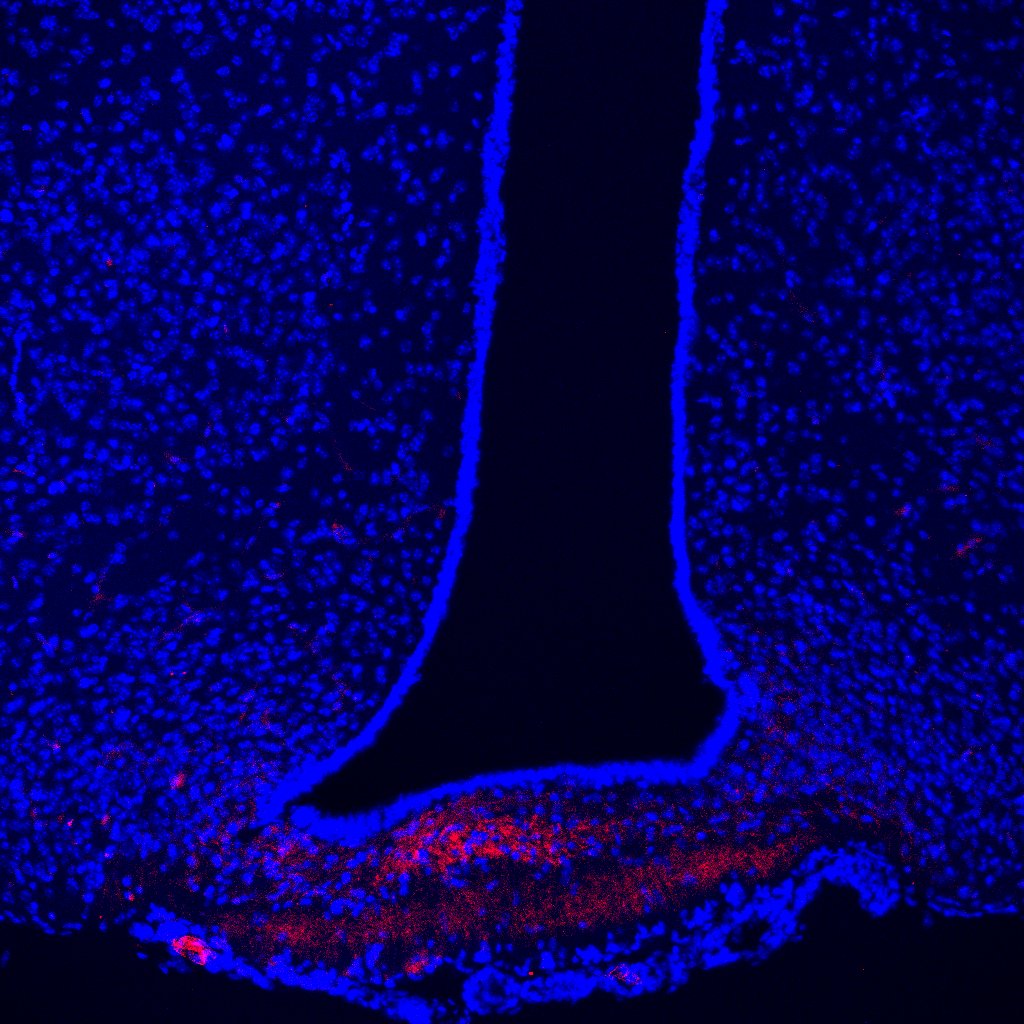

Supplement: Supplementary file 2 — Original pictures of cFos and Cy5 drug appearance shown in Extended Data Fig. 5a–h, including replicates used for quantification. [file 42255_2023_931_MOESM2_ESM.zip › Raw Data Extended Data Figure 5/06-GIPcy5 in ME/55-M-WT-GIP-Hypo.jpg]

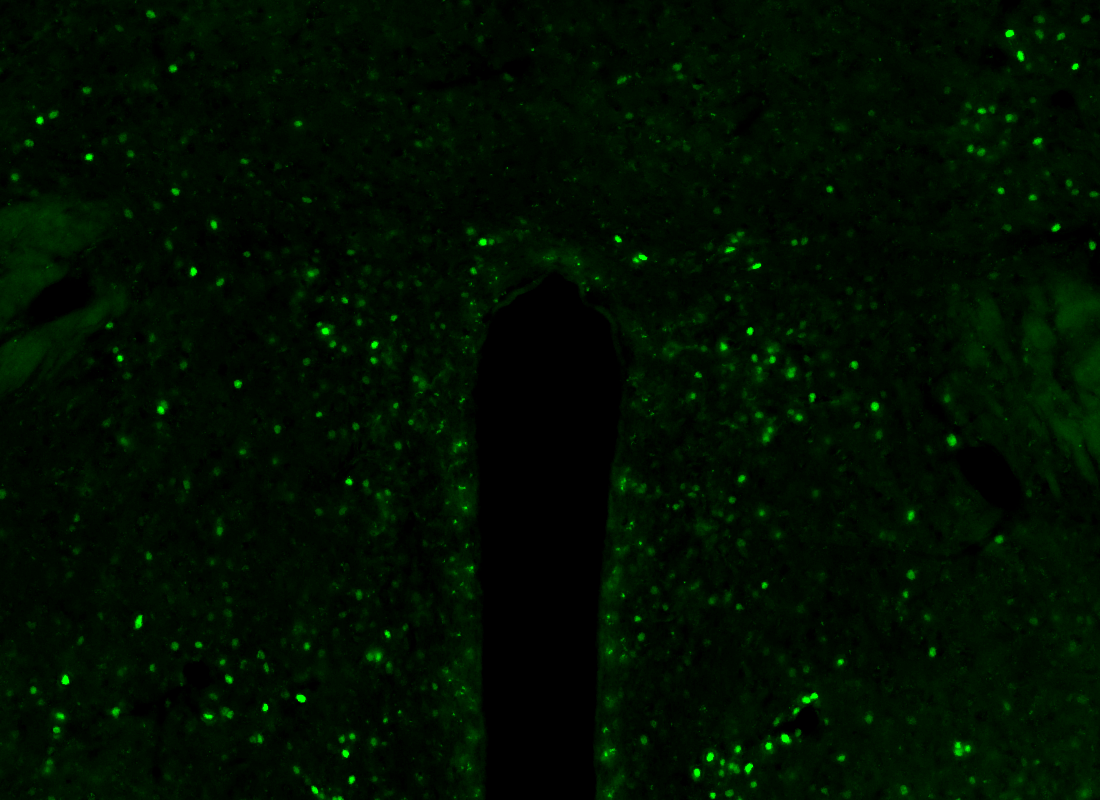

Supplement: Supplementary file 2 — Original pictures of cFos and Cy5 drug appearance shown in Extended Data Fig. 5a–h, including replicates used for quantification. [file 42255_2023_931_MOESM2_ESM.zip › Raw Data Extended Data Figure 5/04-PVN/17-M-KO-Veh-PVN.tif]

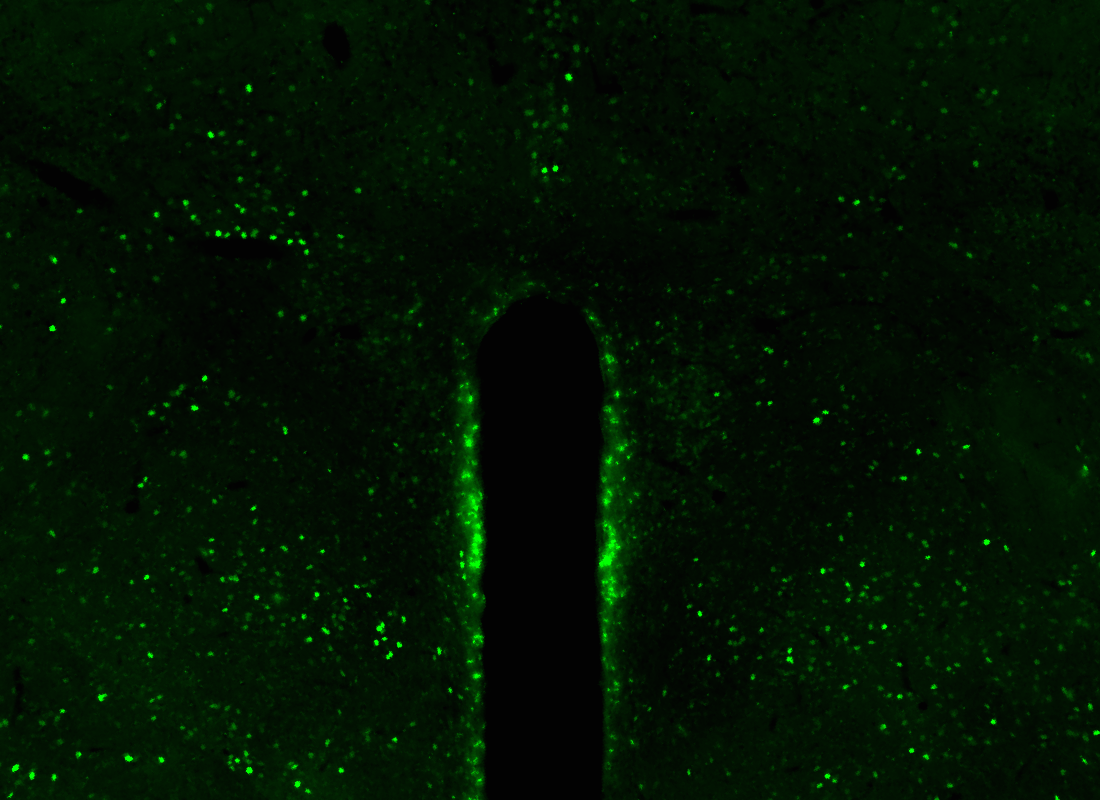

Supplement: Supplementary file 2 — Original pictures of cFos and Cy5 drug appearance shown in Extended Data Fig. 5a–h, including replicates used for quantification. [file 42255_2023_931_MOESM2_ESM.zip › Raw Data Extended Data Figure 5/04-PVN/3-M-KO-GIP-PVN.tif]

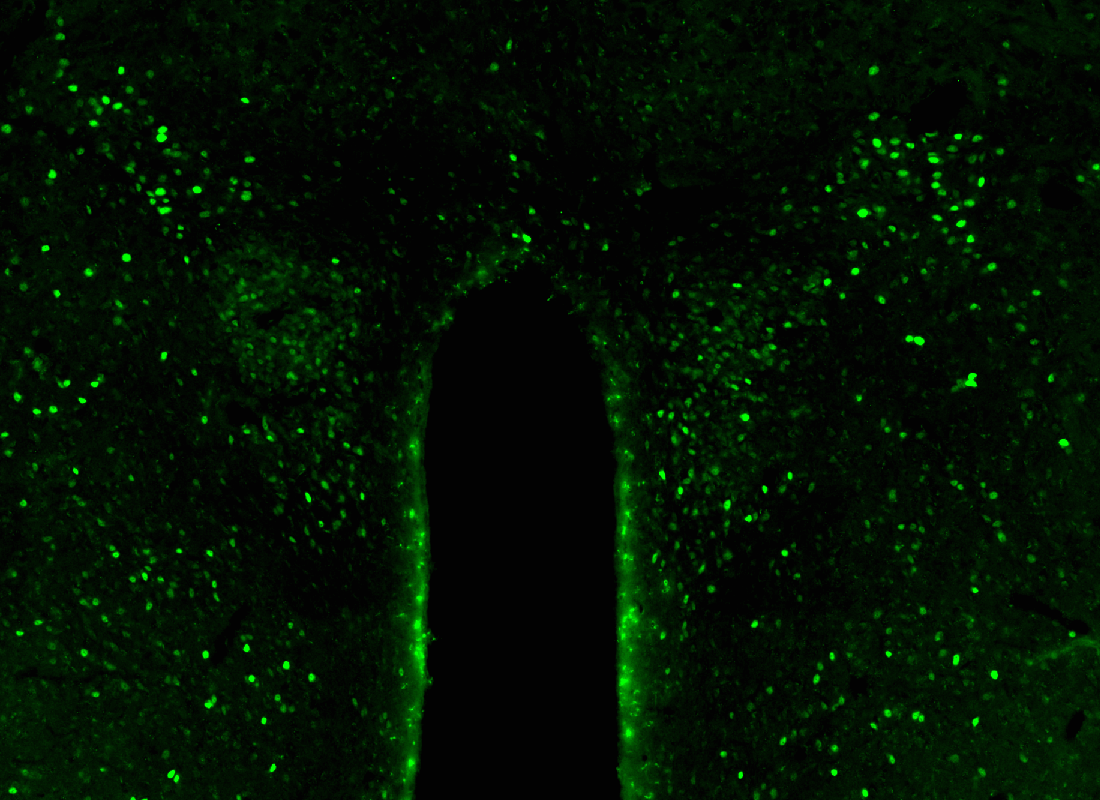

Supplement: Supplementary file 2 — Original pictures of cFos and Cy5 drug appearance shown in Extended Data Fig. 5a–h, including replicates used for quantification. [file 42255_2023_931_MOESM2_ESM.zip › Raw Data Extended Data Figure 5/04-PVN/46-M-WT-GIP-PVN.tif]

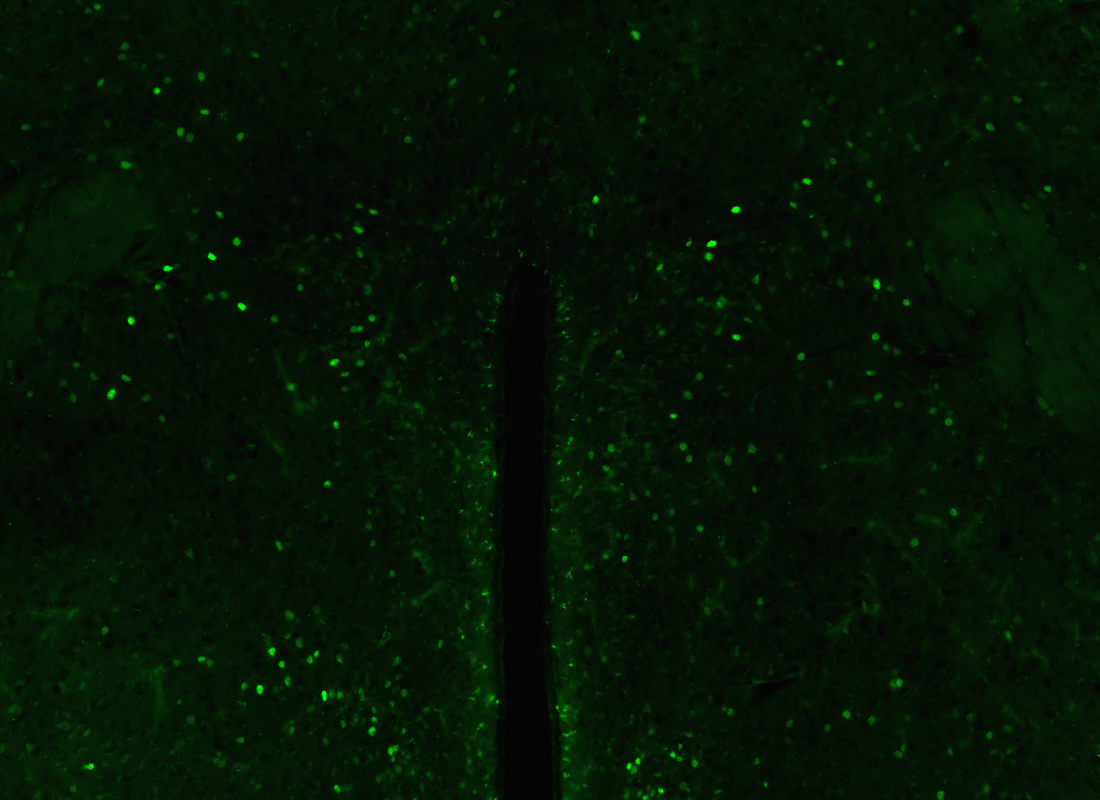

Supplement: Supplementary file 2 — Original pictures of cFos and Cy5 drug appearance shown in Extended Data Fig. 5a–h, including replicates used for quantification. [file 42255_2023_931_MOESM2_ESM.zip › Raw Data Extended Data Figure 5/04-PVN/67-M-WT-Veh-PVN.tif]

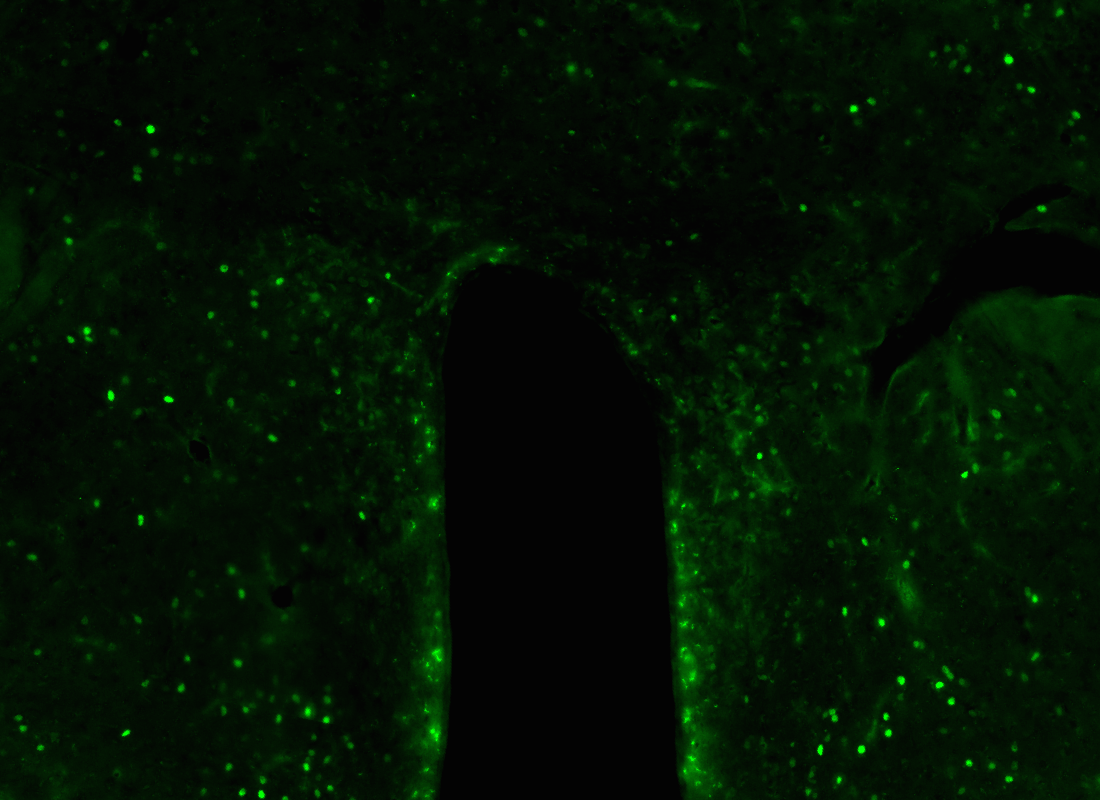

Supplement: Supplementary file 2 — Original pictures of cFos and Cy5 drug appearance shown in Extended Data Fig. 5a–h, including replicates used for quantification. [file 42255_2023_931_MOESM2_ESM.zip › Raw Data Extended Data Figure 5/04-PVN/36-M-KO-Veh-PVN.tif]

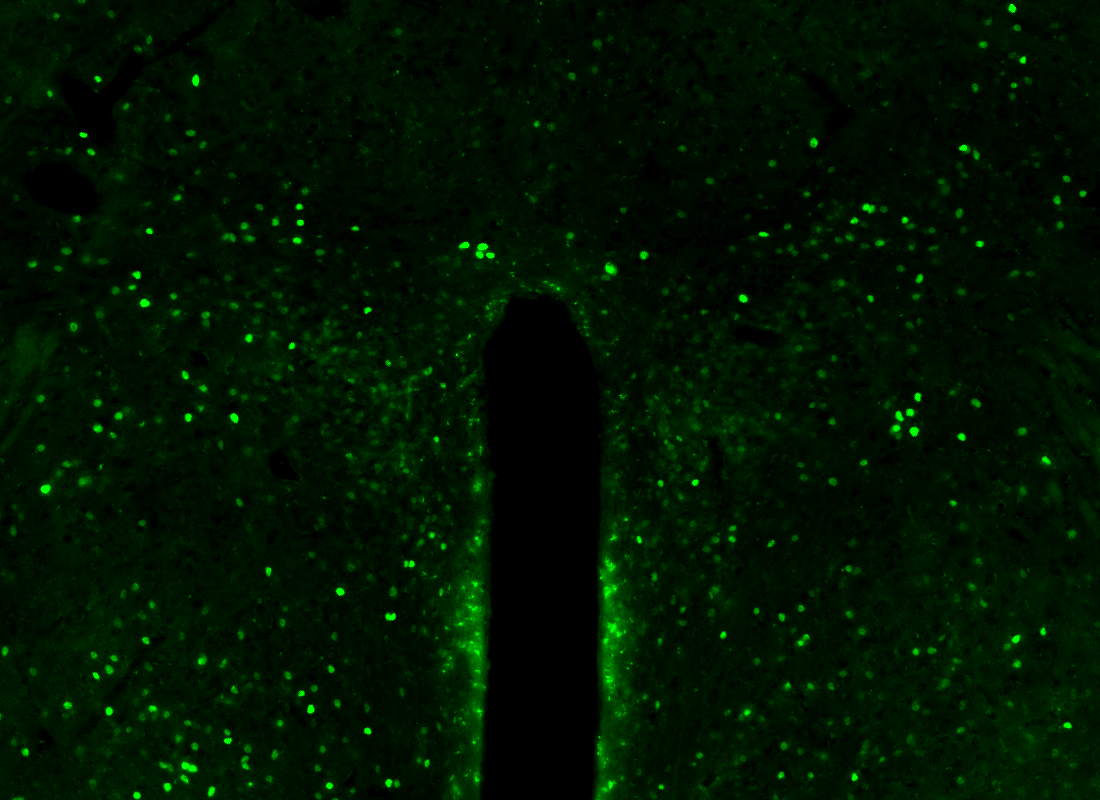

Supplement: Supplementary file 2 — Original pictures of cFos and Cy5 drug appearance shown in Extended Data Fig. 5a–h, including replicates used for quantification. [file 42255_2023_931_MOESM2_ESM.zip › Raw Data Extended Data Figure 5/04-PVN/37-M-KO-Veh-PVN.tif]

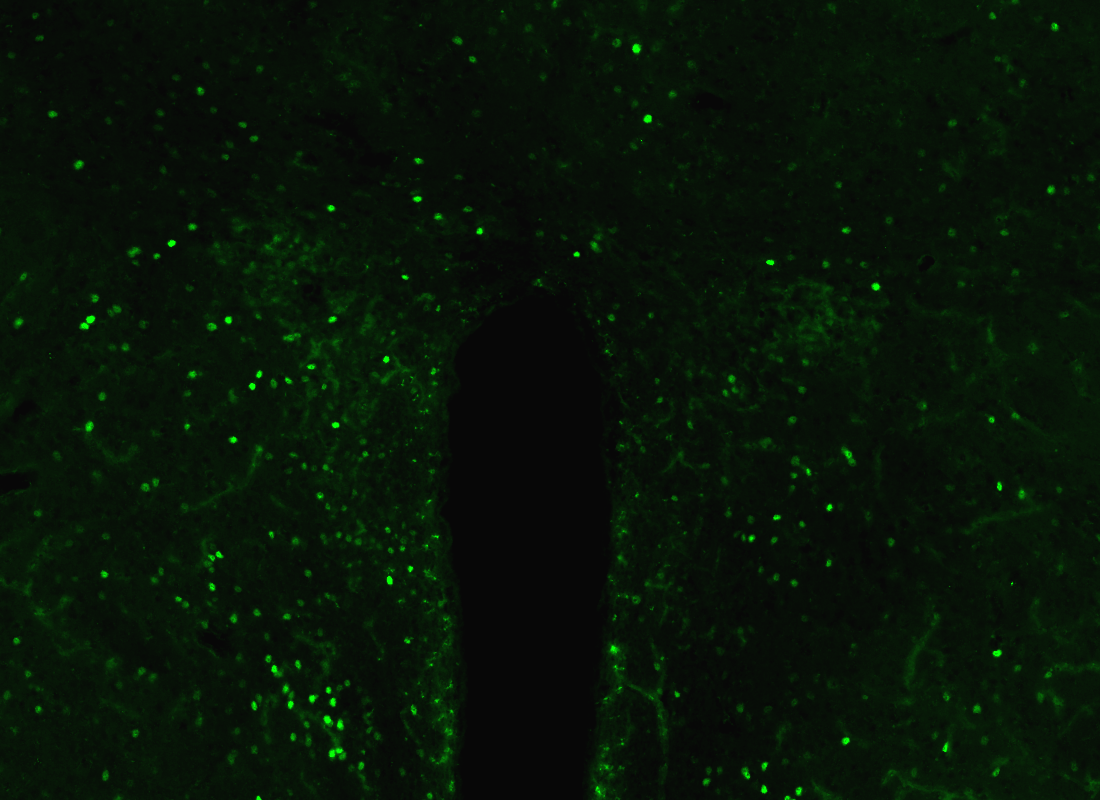

Supplement: Supplementary file 2 — Original pictures of cFos and Cy5 drug appearance shown in Extended Data Fig. 5a–h, including replicates used for quantification. [file 42255_2023_931_MOESM2_ESM.zip › Raw Data Extended Data Figure 5/04-PVN/66-M-WT-Veh-PVN.tif]

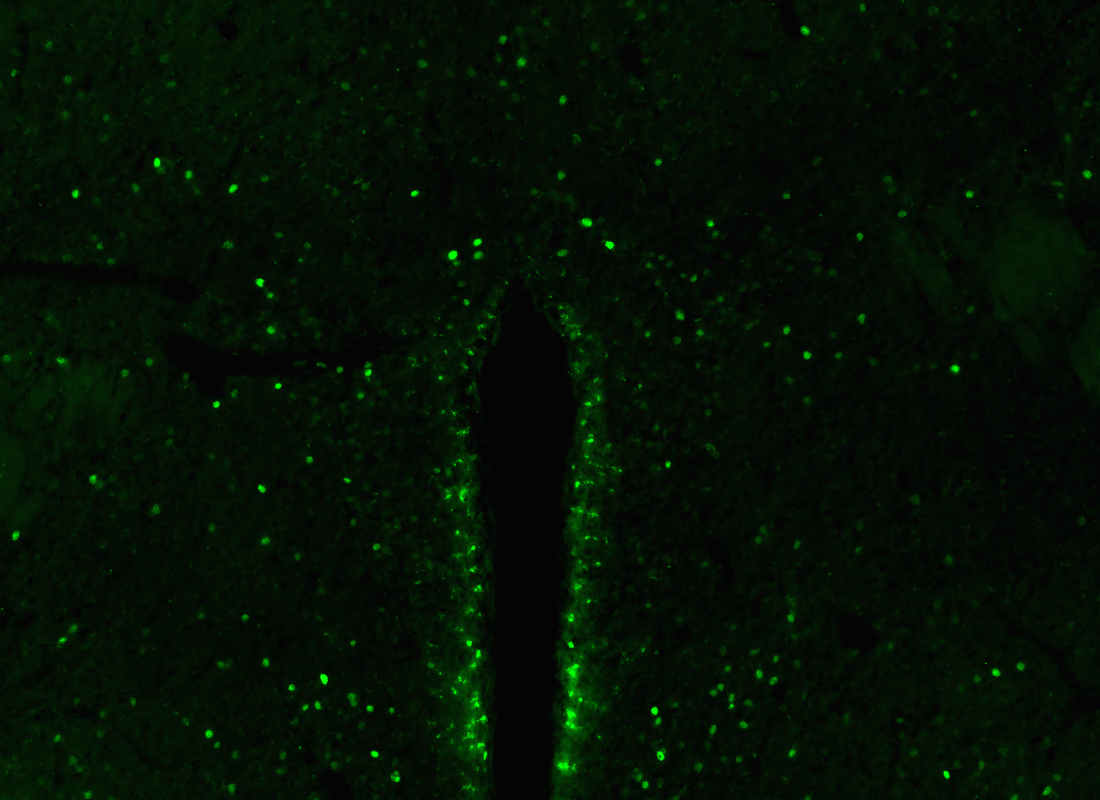

Supplement: Supplementary file 2 — Original pictures of cFos and Cy5 drug appearance shown in Extended Data Fig. 5a–h, including replicates used for quantification. [file 42255_2023_931_MOESM2_ESM.zip › Raw Data Extended Data Figure 5/04-PVN/2-M-KO-GIP-PVN.tif]

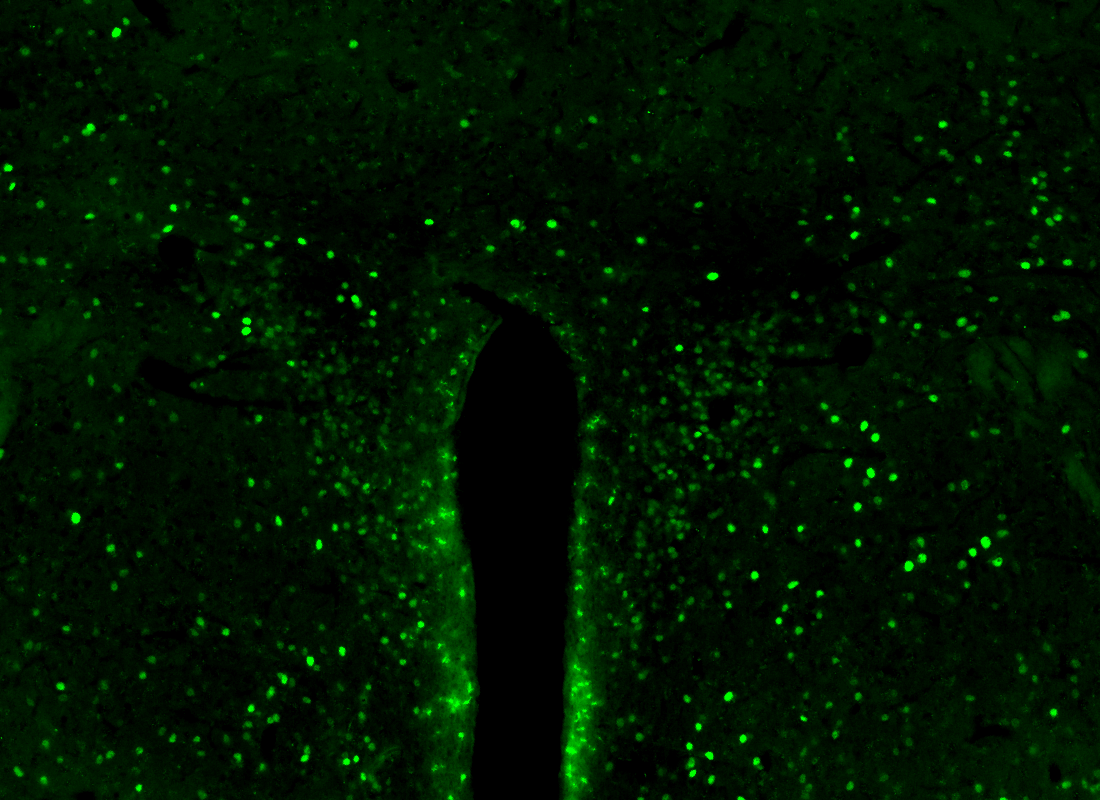

Supplement: Supplementary file 2 — Original pictures of cFos and Cy5 drug appearance shown in Extended Data Fig. 5a–h, including replicates used for quantification. [file 42255_2023_931_MOESM2_ESM.zip › Raw Data Extended Data Figure 5/04-PVN/53-M-WT-GIP-PVN.tif]

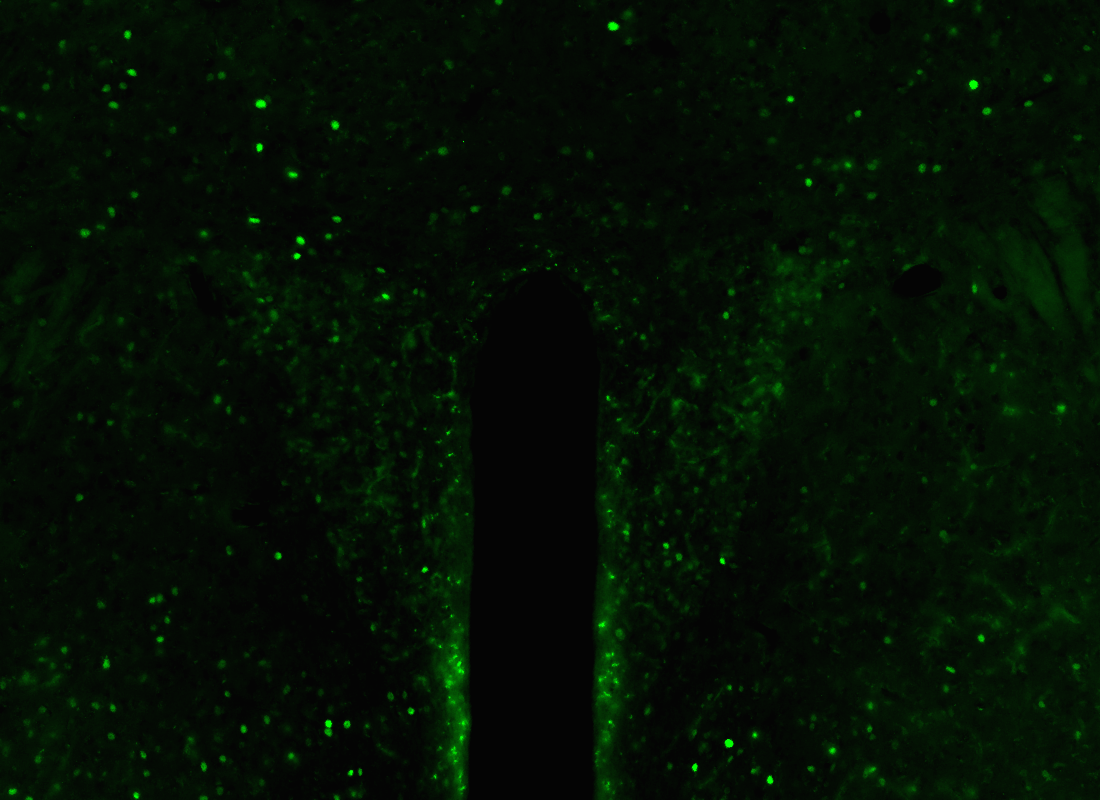

Supplement: Supplementary file 2 — Original pictures of cFos and Cy5 drug appearance shown in Extended Data Fig. 5a–h, including replicates used for quantification. [file 42255_2023_931_MOESM2_ESM.zip › Raw Data Extended Data Figure 5/04-PVN/20-M-KO-Veh-PVN.tif]

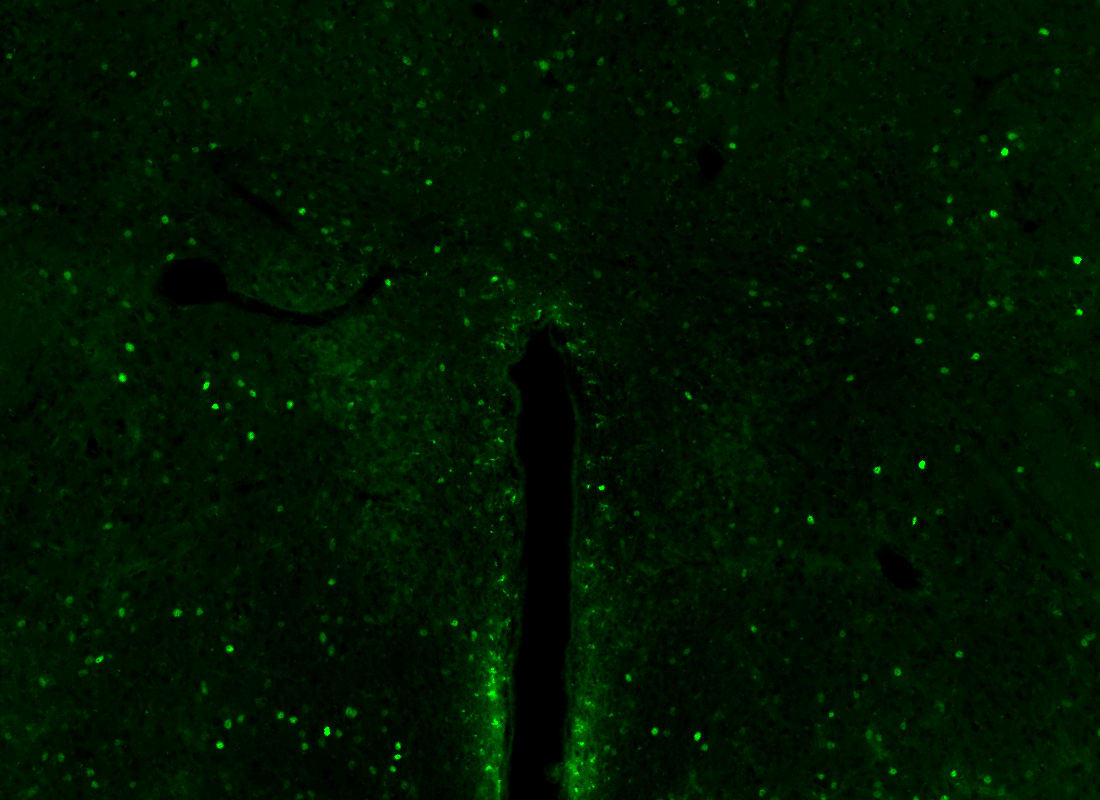

Supplement: Supplementary file 2 — Original pictures of cFos and Cy5 drug appearance shown in Extended Data Fig. 5a–h, including replicates used for quantification. [file 42255_2023_931_MOESM2_ESM.zip › Raw Data Extended Data Figure 5/04-PVN/65-M-WT-Veh-PVN.tif]

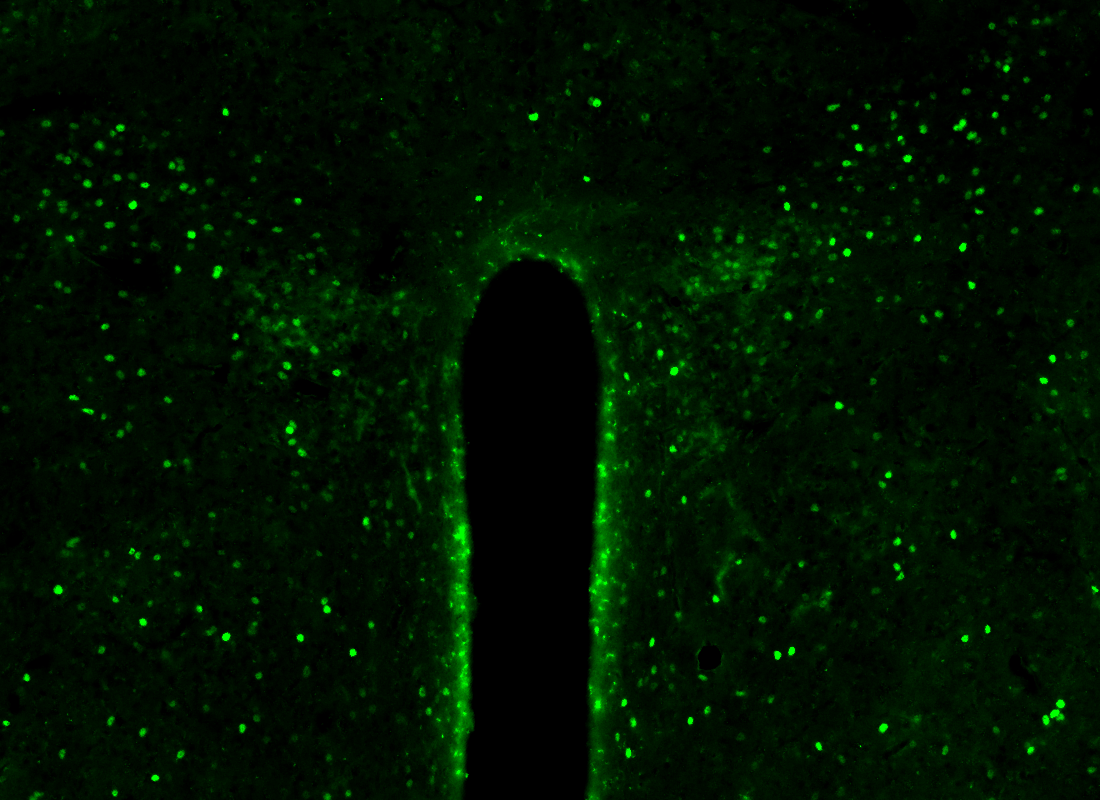

Supplement: Supplementary file 2 — Original pictures of cFos and Cy5 drug appearance shown in Extended Data Fig. 5a–h, including replicates used for quantification. [file 42255_2023_931_MOESM2_ESM.zip › Raw Data Extended Data Figure 5/04-PVN/44-M-WT-GIP-PVN.tif]

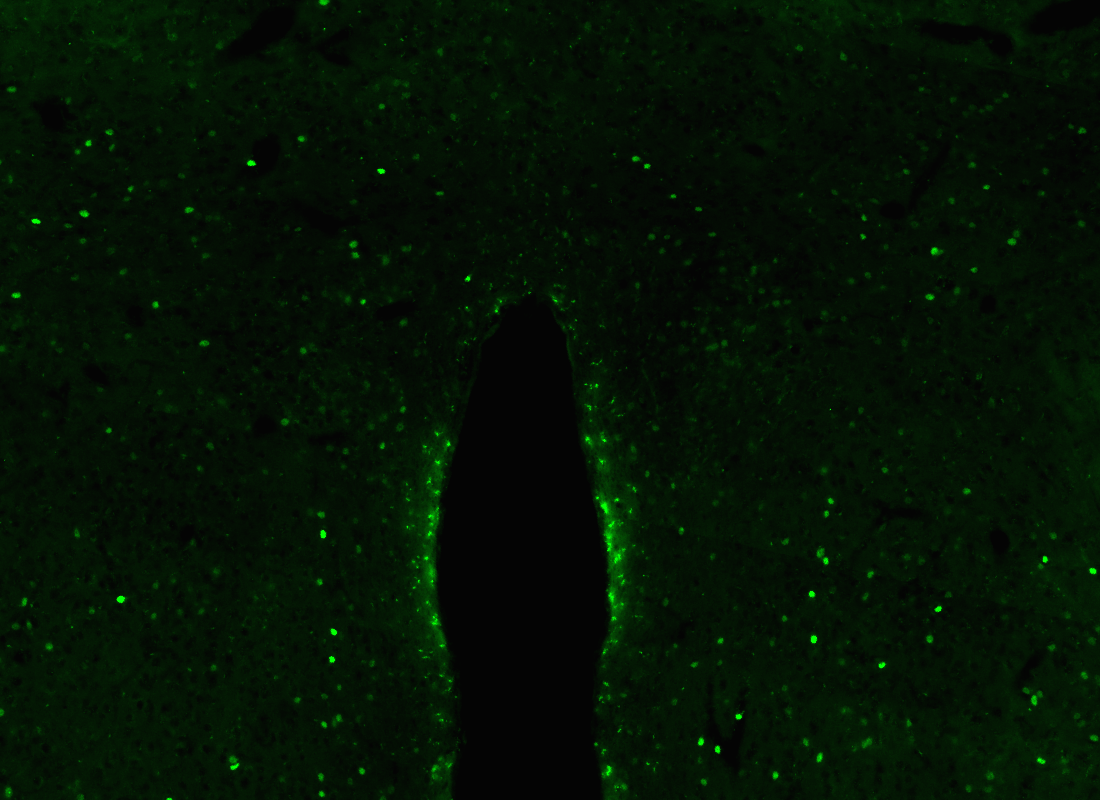

Supplement: Supplementary file 2 — Original pictures of cFos and Cy5 drug appearance shown in Extended Data Fig. 5a–h, including replicates used for quantification. [file 42255_2023_931_MOESM2_ESM.zip › Raw Data Extended Data Figure 5/04-PVN/15-M-KO-GIP-PVN.tif]
